# Heterobimetallic Complexes That Point to When Bond Dissociation Energies Deviate from Computational Expectations

**DOI:** 10.1021/jacs.4c14399

**Published:** 2025-04-15

**Authors:** Raphael Bissig, Raphael Oeschger, Peter Chen

**Affiliations:** Laboratorium für Organische Chemie, ETH Zürich, Zürich CH-8093, Switzerland

## Abstract

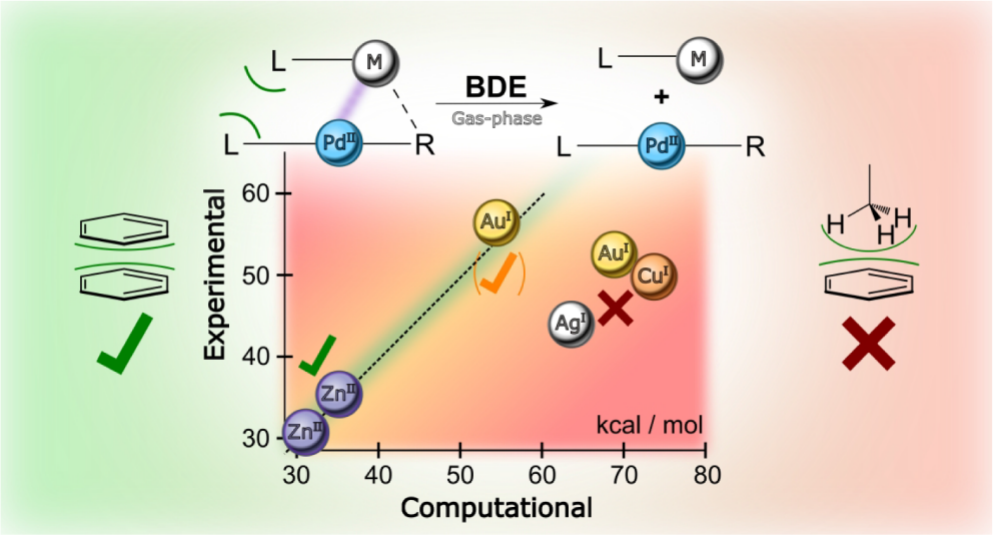

Measurement of the formal, gas-phase, d^8^–d^10^ bond dissociation energy across a series of structurally homologous heterobimetallic complexes of Pd(II) with Cu(I), Ag(I), Au(I), and Zn(II), themselves models for the transition states for transmetalation in Sonogashira and Negishi couplings, finds large discrepancies relative to predictions by a commonly used dispersion-corrected density-functional theory method, DFT-D3(BJ), but *not in all cases*. Control studies on the threshold collision-induced dissociation (T-CID) of electrosprayed molecular ions, as well as the deconvolution of the bond energy from the experimentally measured energy-resolved cross sections, indicate that the experimentally determined bond dissociation energies are most likely correct, which raises the question of why the computational methods, while sometimes agreeing acceptably with experiment, can also sometimes disagree egregiously. While initial attempts to characterize the discrepancy focused on the metal–metal interaction, the most likely origin of the discrepancy appears to be an uneven treatment of nonbonded interactions, among them medium-ranged correlation effects and London dispersion, between the ligands on the two metal centers. The contribution of these effects to the formal bond dissociation energy is large enough to be chemically significant, but it appears to depend on the nature of the interacting groups, specifically the hybridization at carbon, and, more importantly, their relative orientation. Whereas face-to-face aryl–aryl interactions seem to be modeled well by PBE-D3(BJ), a representative DFT-D3 method, alkyl-aryl, and edge-to-face aryl–aryl interactions appear to be overestimated. The consequences for structure and stability in organic and organometallic molecules are discussed, especially with regard to relative energies of conformers and interconverting valence isomers.

## Introduction

Simultaneously from the direction of the development of cross-coupling reactions in organic synthesis,^1^ as well as from the direction of bonding theory in inorganic chemistry, interest has grown in heterobimetallic complexes with direct interaction between d^8^ and d^10^ metal centers.^2−6^ In the former case, d^8^–d^10^ heterobimetallic complexes of Pd(II) and either Cu(I), Zn(II), or other d^10^ metal centers model the transition state for the transmetalation step in Sonogashira and Negishi couplings.^7−15^ In the latter case, the d^8^–d^10^ heterobimetallic complexes present theoretically interesting molecules where the bonding has been variously described as having large contributions from electrostatic,^16−19^ orbital,^20−23^ or even dispersion^24^ interactions. Metal–metal bond have been described as particularly difficult to treat properly,^6,25,26^ or even classify.^27^ Appropriate experimental systems for which physical measurements may provide guidance in the development of theory are most welcome, especially if the test systems closely resemble structures found along the reaction coordinate for relevant catalytic reactions.

**Figure 1:**
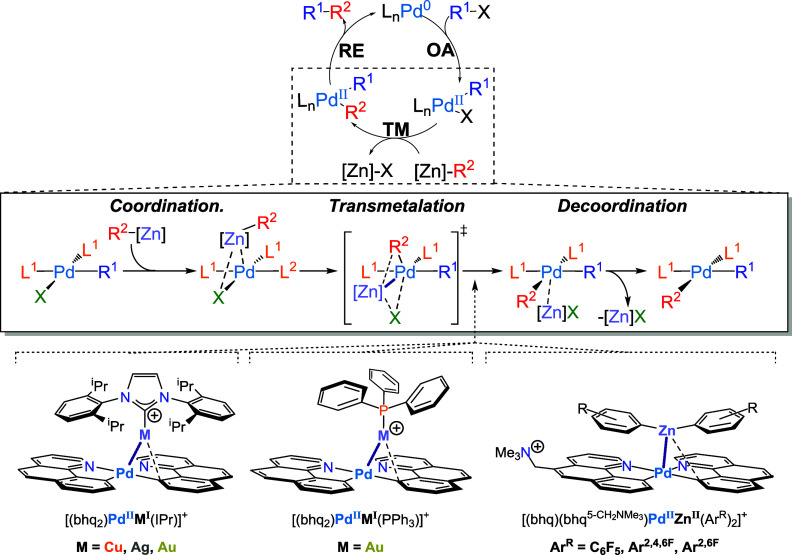
(top) Idealized catalytic cycle for the Negishi cross-coupling reaction, showing oxidative addition (OA), transmetalation (TM) and reductive elimination (RE). (middle) a schematic representation of the potential reaction mechanism for the transmetalation step, including association and dissociation of the organozinc species before and after the transfer of the organyl, respectively. (bottom) Cationic d^8^–d^10^ heterobimetallic complexes, prepared for ESI-MS/MS determination of the gas-phase bond dissociation energy by means of threshold collision-induced dissociation (T-CID).

Recently, we reported Pt^II^–Cu^I^, Pt^II^–Au^I^, Pd^II^–Cu^I^ and Pd^II^–Zn^II^ complexes where the d^10^ metal bridges the d^8^ metal and an *ipso* carbon.^28−33^ All of the complexes show the metals separated by less than the sum of their van der Waals radii; the cases with Pd, which are the ones most relevant for catalysis, were found to have distances between Pd(II), and either Cu(I) or Zn(II), measured from single-crystal X-ray diffraction experiments, of 2.55 or 2.58 Å, respectively. The metals are clearly bonded, from whose point-of-view it is the *ipso* carbon that is bridging. Overlay of the coordinates of the two metals and the bridging carbon on the computed structures for the transition states for the transmetalation step in the Sonogashira and Negishi couplings reveals a high degree of congruence,^30^ from which we claimed that the fully characterized, isolable heterobimetallic complexes should model the electronic structure and thermochemistry of the nonisolable transition states. In particular, the bridged heterobimetallic core, with the three points, i.e., Pd, d^10^ metal, and *ipso* carbon, defining a plane, fixes the spatial relationship between the remaining pendant groups, which will become an important feature in the arguments to come. Figure 1 shows some structures discussed in the present work, and their relation to putative structures in the catalytic cycle for cross-coupling reactions. The spectacular failure, thus far, of dispersion-corrected DFT methods, at a reasonable level of theory, to reproduce experimental bond dissociation energies for some large molecules,^34−38^ in particular for compounds with metal–metal bonds,^33^ calls into doubt the application of these methods to compute species in the catalytic cycle for the related cross-coupling reactions. In the present work, we report the synthesis and characterization of a wider range of isostructural d^8^–d^10^ heterobimetallic complexes of Pd(II), with Cu(I), Ag(I), Au(I) and Zn(II), for which we again find large discrepancies between the gas-phase bond dissociation energies and the corresponding bond energies computed with DFT-D3, but, this time, much to our surprise, *not in all cases*. With discrepancies showing up only in *some* complexes, we gain a tool to validate the experimental method, on the one hand, and seek the underlying physical origin of the difference to theory, on the other. A careful analysis of the cases where the predictions fail by a large margin, and the ones where they agree more acceptably, reveals that, contrary to the original expectations, the metal–metal bonding, *per se*, is treated adequately enough, but that the nonbonded interactions between the ligands on the two metal centers are treated unevenly, depending on the type of substituent, e.g., alkyl, aryl, etc., and their relative orientation. We discuss further the consequences of the uneven treatment for the application of DFT-D3 for complex, flexible molecules with many conformations when there are multiple, competing noncovalent interactions of a similar magnitude.

## Experimental Section

Table 1 shows the synthesis or *in situ* preparation of the heterobimetallic complexes **1**^+^–**6**^+^ and **8**^+^ with either tetrafluoroborate, triflate or BArF as counterions. Complex **1**^+^ was already described and investigated in previous reports,^30^ complexes **2**^+^ and **3**^+^ are closely related, the difference being the second metal center, Ag and Au, respectively. Complexes **4**^+^–**6**^+^ were generated *in situ* and used as prepared in ESI-MS experiments. Complex **4**^+^ is structurally analogous to a previously reported complex [(bhq)_2_Pd–Zn(C_6_F_5_)_2_],^32^ the difference being the presence of a remote charge-tagged moiety permitting ESI-MS investigations. Complexes **5**^+^ and **6**^+^ are variants thereof with slightly varied substitution patterns on the fluoroaryl ligands located on the electrophile, Ar = 2,4,6-C_6_H_2_F_3_ for **5**^+^ and 2,6-C_6_H_3_F_2_ for **6**^+^ respectively. In addition to complex **3**^+^, a variant thereof, **8**^+^, was investigated in which the NHC-based ligand on the electrophile was replaced with a phosphine type ligand.

**Table 1:**
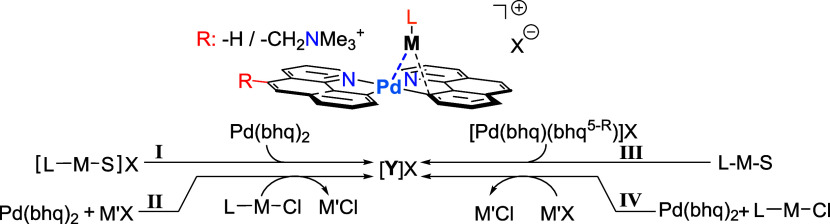
Equimolar Stoichiometries for All Reactions

		Conditions	Compound [Y]X	
Pathway	M’	Solvents/Added Solvent for Crys.	Abbreviation	Numbering
**I**	-	CH_2_Cl_2_	[(bhq)_2_**Pd**^II^–**Cu**^**I**^(IPr)^+^]^−^OTf	[**1**^**+**^]^−^OTf
	-	CH_2_Cl_2/_*n*-hexane	[(bhq)_2_**Pd**^II^–**Au**^**I**^(IPr)^+^] ^–^OTf	[**3**^**+**^]^−^OTf
**II**	Na	CH_2_Cl_2/_*n*-hexane	[(bhq)_2_**Pd**^II^–**Ag**^**I**^(IPr)^+^]BArF^–^	[**2**^**+**^]BArF^–^
**III**	-	CH_2_Cl_2_, THF		[**4**^**+**^]BArF^–^
	-	CH_2_Cl_2_, THF		[**5**^**+**^]BArF^–^
	-	CH_2_Cl_2_, THF		[**6**^**+**^]BArF^–^
**IV**	Ag	CH_2_Cl_2_	[(bhq)_2_**Pd**^II^–**Au**^**I**^(PPh_3_)^+^]BF_4_^–^	[**8**^**+**^]BF_4_^–^

**Figure 2:**
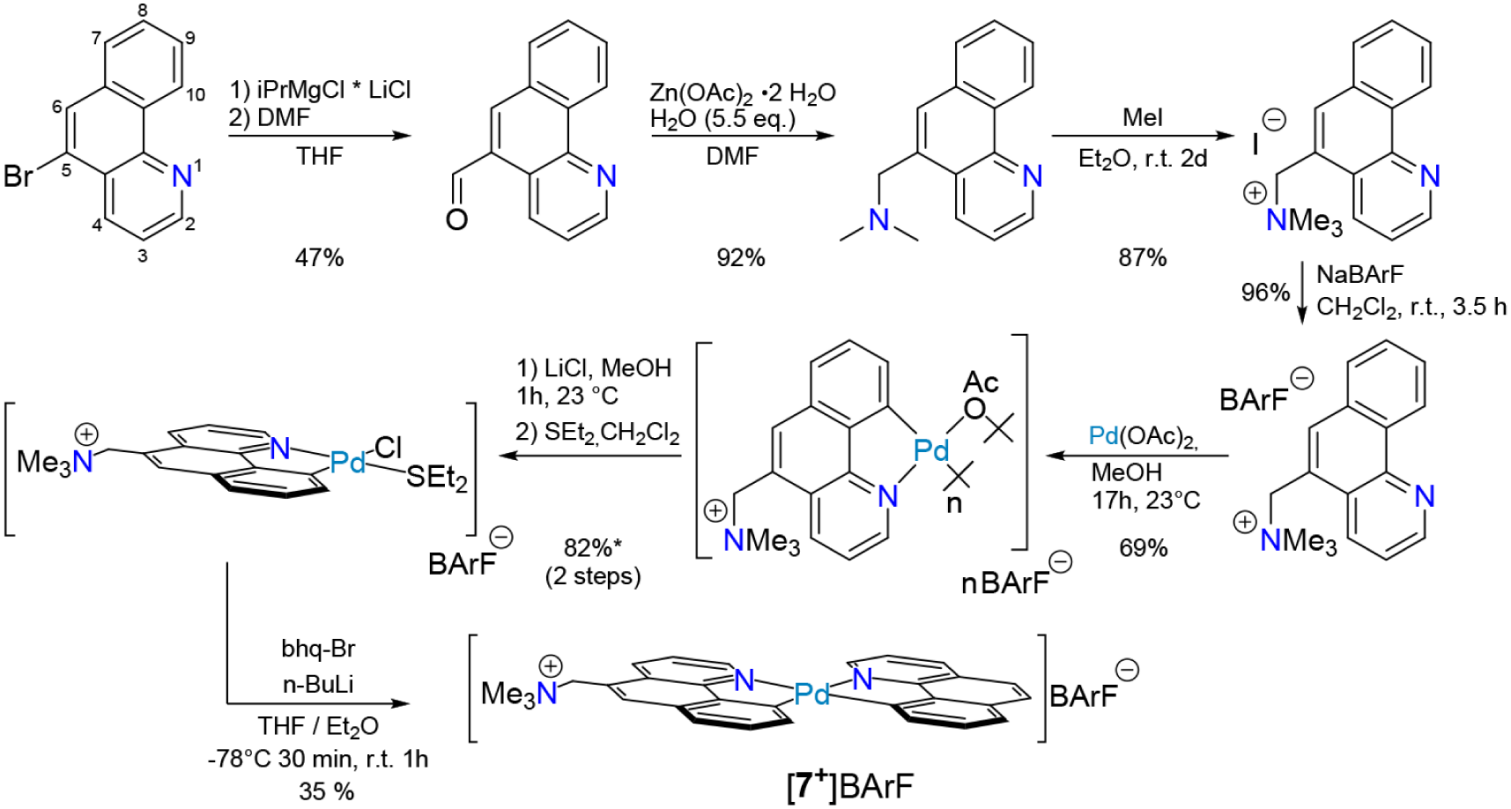
Synthesis of complex , [**7**^**+**^]BArF^–^, with additional information listed: yield in [%].

Figure 2 shows the synthesis of , a heteroleptic *bis*-(1,10-benzo[*h*]quinolato) complex with a particular substitution, a charge tag, which is otherwise analogous to our previously reported cis-*bis*(1,10-benzo[*h*]quinolato)palladium(II) complex.

The more extensive synthetic effort to prepare the heteroleptic complex, , [**7**^**+**^]BArF^–^, was needed for ESI-MS gas-phase studies of Pd–Zn heterobimetallic complexes related to the neutral species we reported earlier.^32^ Whereas **1**^**+**^–**3**^**+**^ are natively charged, making them amenable to transfer from solution to the gas phase by electrospray, **4**^**+**^–**6**^**+**^ are charged by virtue of a prosthetic “charge tag” attached at an (as much as possible) innocuous position of a ligand on the otherwise electrically neutral complex. We reasoned that the charge tag was better attached to a ligand on the Pd complex, so as to permit us to produce heterobimetallic complexes with different electrophiles. The mass spectrometric studies of **4**^**+**^–**6**^**+**^ required preparation of a heteroleptic (bhq)_2_Pd analog, as the homoleptic complex with charge tags on both bhq moieties could potentially further complicate the ion–molecule dynamics because the additional Coulombic repulsion between the two charge tags^39^ might introduce additional, unwanted dissociation channels. Accordingly, the heteroleptic complex, [**7**^**+**^]BArF^–^ was prepared by the route shown in Figure 2.

**Figure 3:**
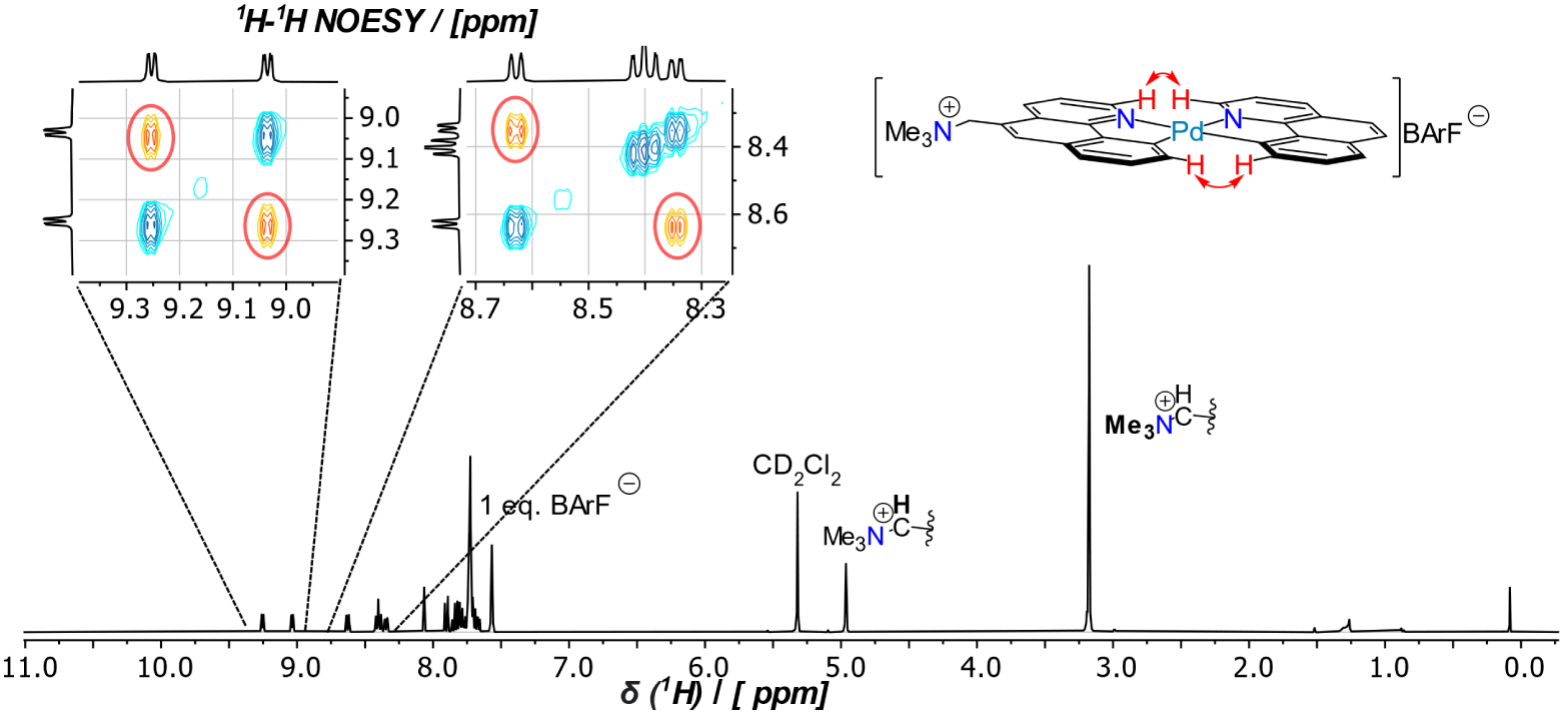
^1^H NMR spectrum of the , [**7**^**+**^]BArF^–^, complex in CD_2_Cl_2_. The insets show selected ^1^H–^1^H-NOESY regions for the assigned hydrogen nuclei shown in the structure on the right.

Whereas the homoleptic complex, (bhq)_2_Pd had been reported, and fully characterized structurally by von Zelewsky in 1996,^40^ the preparation of the heteroleptic complex **7**^**+**^ could proceed, in principle, to either the *cis* or the *trans* isomers. As a model for intermediates and transition states in the cross-coupling catalytic cycle, the *cis* isomer would be strongly preferred. ^1^H–^1^H-NOESY spectroscopy, Figure 3, confirms the *cis* relationship of the bhq and  ligands in **7**^**+**^.

Electrospray ionization tandem mass spectrometry experiments were performed as described in our previous work.^28−30,33^ Threshold collision induced dissociation (ESI-MS/MS, T-CID) experiments were performed on a modified TSQ Quantum Ultra mass spectrometer. NMR spectra were recorded with Bruker Ascend 400 MHz. X-ray crystallographic studies were performed with XtaLAB Synergy, Dualflex, Pilatus 200 K diffractometer at 100 K. DFT calculations were performed with ORCA 5.0.2 and ADF 2016. We note that computational results are reported mostly for PBE-D3(BJ), although we did check a number of other functionals, dispersion corrections with D3zero, and (in part) D4(BJ), and corrections for relativistic effects. See SI, §3.1.3 and §4.5.4. The impact on the computed bond dissociation energies was small, so PBE-D3(BJ) was chosen for use as a representative method.

## Results

### X-Ray Structures

**Figure 4:**
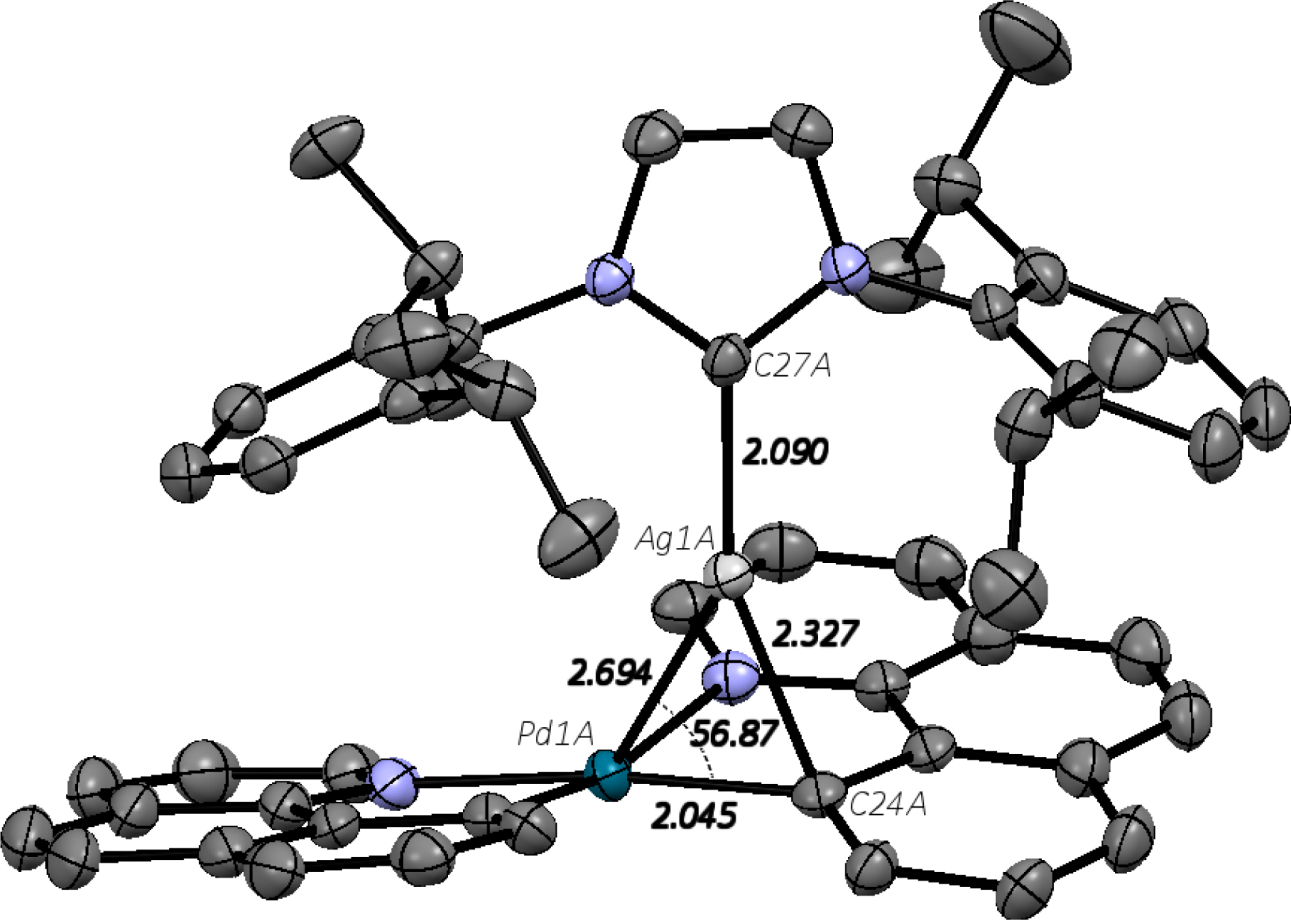
XRD structure of [(bhq)_2_**Pd**^II^–**Ag**^**I**^(IPr)^+^]BArF^–^, [**2**^**+**^]BArF^–^, with thermal ellipsoids, ORTEP 50% and selected bond distances in [Å] and angles in [°]. Two molecules were observed in the unit cell, for sake of clarity only one is shown, also hydrogen atoms and the counterion BArF^–^ are omitted for clarity.

The heterobimetallic complexes **1**^**+**^–**3**^**+**^, as the triflate or BArF salts, were crystallized and analyzed by X-ray diffraction (XRD). Full details of the X-ray structures, with coordinates, are included in the CCDC deposition files. A structure for **2**^**+**^, representative for the series, is shown in Figure 4.

Key structural parameters from the XRD analysis, and a comparison to the computed structures, fully optimized using PBE-D3(BJ)/def2-TZVP, are given in Tables 2 and 3, as well as for the neutral Pd–Zn complex we had previously reported.^32^ All of the structures show metal–metal bonds, i.e. Pd-M distances less than the sum of either the van der Waal or covalent radii of the two metal atoms.^41^ The structures also all show bridging of the d^10^ metal, with the distances from the *d*^10^ metal center to the *ipso* carbon, marked as C1 in the structure, indicating bonding. The extent of bridging is less pronounced for the Pd–Zn system than it is for the Pd–Cu, Pd–Ag, and Pd–Au complexes **1**^**+**^–**3**^**+**^. The computed structures, optimized with PBE-D3(BJ)/def2-TZVP reproduce the XRD structures of **1**^**+**^–**3**^**+**^ extremely well, with differences in the key bond distances well below 0.1 Å and the difference in the key bond angle in the range of 1–4°. For the neutral Pd–Zn complex, the agreement of the computed geometry with the XRD structure is noticeably worse, but still not bad, consisting primarily in even less bridging in the computed structure, which one may see by the significant deviation in the Zn–C1 distance and Zn–Pd–C1 angle (while the Pd–Zn and the Pd–C1 distances agree acceptably.)

**Table 2:**
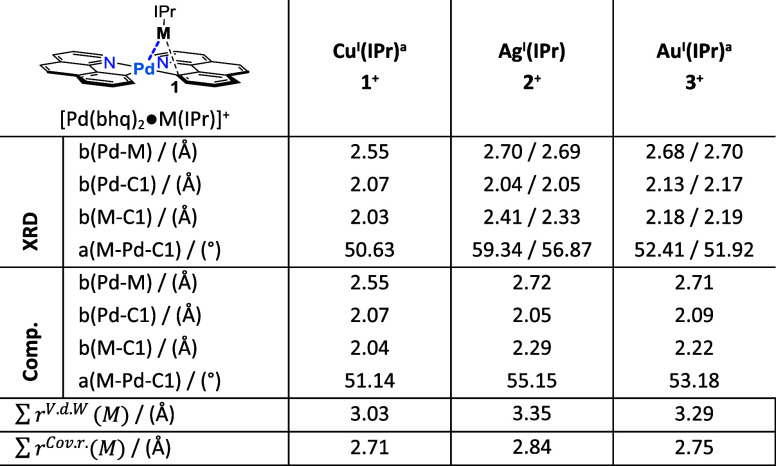
Selected Structural Parameters from Sc-XRD Structures Compared to Computationally Structures Optimized with (PBE-D3(BJ)/def2-TZVP)^b^

a Reported crystal structures **1**^+^,^31^ and **3**^+^.^42^

b Sum of covalent/v.d.W radii of the participating metals are listed.^43^ For complexes 2^+^ and 3^+^ two molecules were observed in the unit cell.

**Table 3:**
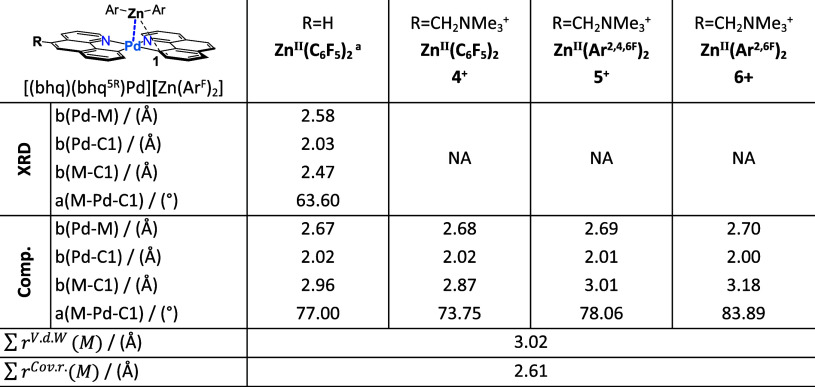
Selected Structural Parameters from the sc-XRD Structure for [(bhq)_2_Pd][Zn(C_6_H_5_)_2_] Compared to Computationally Structures for **4**^**+**^–**6**^**+**^, Optimized with PBE-D3(BJ)/def2-TZVP^b^

a Previously reported crystal structure for [(bhq)_2_Pd][Zn(C_6_H_5_)_2_].^32^

b Sum of covalent/v.d.W radii of the participating metals are listed.

### Bond Dissociation Energies by T-CID Experiments and DFT Calculation

**Figure 5:**
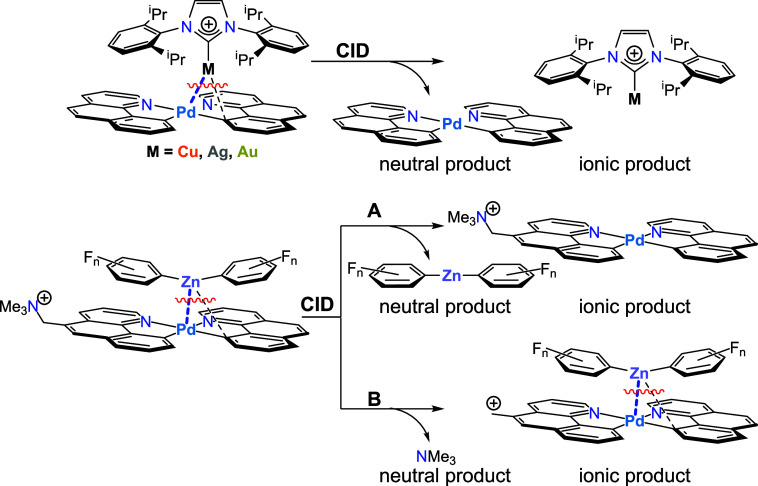
Collision-induced dissociation (CID) of **1**^**+**^–**6**^**+**^, showing the charged product(s) whose cross-section is measured as a function of collision energy.

**Figure 6:**
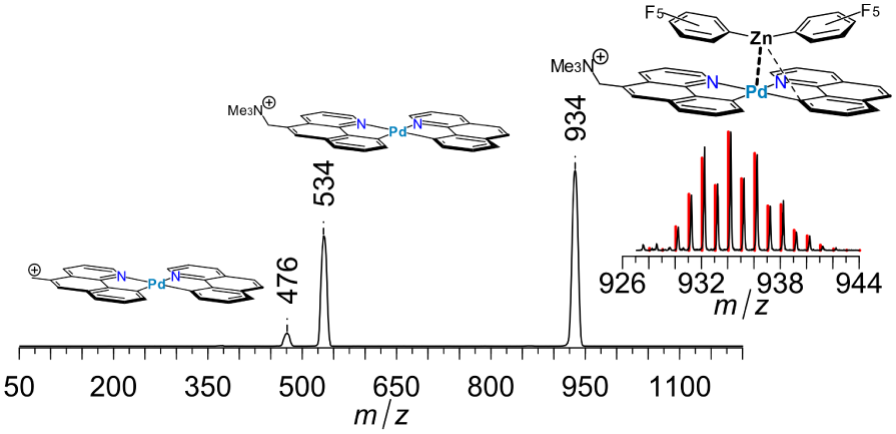
ESI-MS/MS CID spectrum of ion , **4**^**+**^, with 110 μTorr of Ar collision gas in the collision cell at collision offset of 80 V, a high collision energy. Inset: experimental (black) and simulated (red) isotopic pattern of parent ion. Proposed structures for both parent and fragment ions are shown. Marked numbers refer to *m*/*z* of the peak maxima.

**Figure 7:**
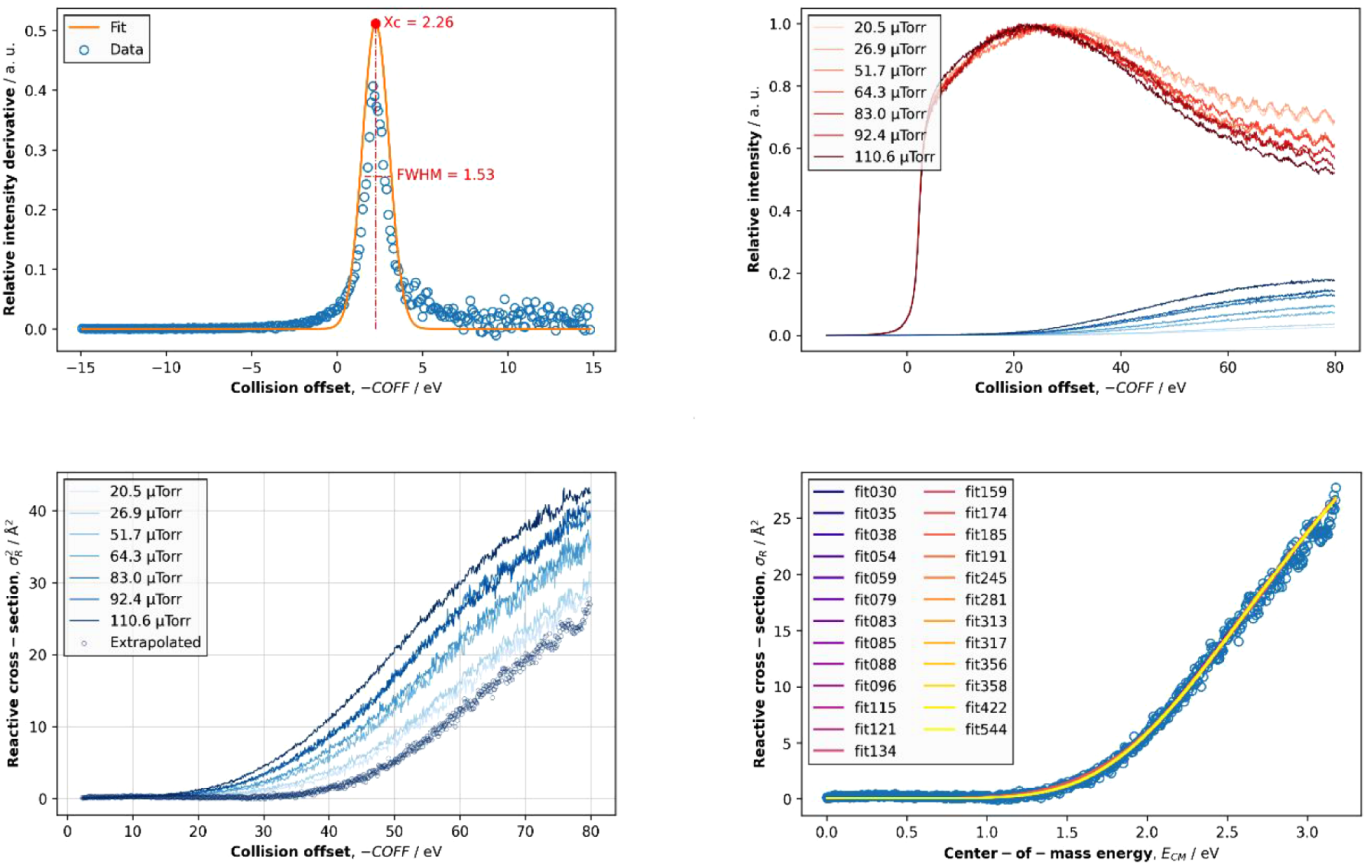
Representative data for the threshold collision-induced dissociation (T-CID) of **4**^**+**^. The panels show the measured kinetic energy distribution of the **4**^**+**^ parent ion after electrospray, desolvation, and thermalization (upper right), the ion intensities of the parent and fragment ions at different pressures of Ar in the octopole collision cell, as a function of collision energy offset in the laboratory frame (upper right), the extrapolation of the fragment ion cross-section to zero-pressure, i.e., single-collision conditions (lower left), and the fit of the cross-section, as a function of collision energy in the center-of-mass frame, with the L-CID program to extract the *E*_0_ value (lower right).

Threshold collision-induced dissociation (T-CID) experiments were executed to measure the bond dissociation energy for the cleavage of **1**^**+**^–**6**^**+**^ into separate monometallic units, shown schematically in Figure 5. In each case, solutions of the triflate or BArF salts were electrosprayed in a modified ESI-MS/MS mass spectrometer, and the appropriate parent ion selected by its *m*/*z* ratio. The threshold for dissociation was measured by monitoring the cross-section for production of the charged fragment ion as a function of collision energy. A representative collision-induced dissociation mass spectrum is shown in Figure 6 for **4**^**+**^. The optimized kinetic energy distribution of the parent ion, the dissociation cross sections as a function of collision energy, the extrapolation of the cross-section to zero pressure, and the L-CID fit to extract the bond dissociation energy, *E*_0_, are all shown in Figure 7 for **4**^**+**^.

The extracted *E*_0_ values for all six complexes, **1**^**+**^–**6**^**+**^ are summarized in Table 4, together with computed bond dissociation energies, the latter taken from PBE-D3(BJ)/def2-CBS(3,4) single point energies at PBE-D3(BJ)/def2-TVZP-optimized geometries. Having confirmed the computed geometries against the XRD structures, we computed the bond dissociation energies with triple- and quadruple-ζ basis sets, which should be large enough to reduce basis set superposition error (BSSE) to about the same magnitude as the level of experimental uncertainty, and extrapolated to the complete basis set limit. Table 4 also lists the discrepancy between the experimental *E*_0_ values and those from DFT. Of note is the consistent trend in which the Pd–Cu, Pd–Ag, and Pd–Au bonds are computed to be 19 ± 1 kcal/mol stronger than that found in experiment, while the three Pd–Zn bonds were all computed to be about 2–3 kcal/mol weaker than experiment. One should note that, while the latter discrepancy is just barely outside the claimed (usual) uncertainty limits of the experimental measurement, both the magnitude and sign of the small discrepancies are consistent.

**Table 4:** Gas-Phase Bond Dissociation Energies for CID Processes Depicted in Figure 5, Extracted by L-CID from T-CID Data, Compared to Bond Dissociation Energies Computed with PBE-D3(BJ)/def-2-CBS(3,4) at PBE-D3(bj)/def2-TVZP-Optimized Geometries^a^

BDE(Pd-M) kcal/mol	1^+^	2^+^	3^+^	8^+^	4^+^	5^+^	6^+^
	M = Cu, L = IPr	M = Ag, L = IPr	M = Au, L = IPr	M = Au, L = PPh_3_	M = Zn, L = C_6_F_5_	M = Zn, L = C_6_H_2_F_3_	M = Zn, L = C_6_H_3_F_2_
Experiment (L-CID)	50.6 ± 1.4	44.2 ± 1.4	51.5 ± 2.8	56.3 ± 2.2	35.2 ± 1.0	29.8 ± 1.0	30.1 ± 0.3
PBE-D3(BJ)/def2-CBS(3,4)	69.1	62.5	71.4	59.5	31.9	28.2	27.4
discrepancy	–19	–18	–20	–3	+3	+2	+3

a Note the sign of the discrepancy as an indicator of whether the measured BDE is higher or lower than the computed one.

## Discussion

In several previous studies, we had investigated the bond dissociation energies in d^8^–d^10^ heterobimetallic complexes with Pd(II) or Pt(II) as the d^8^ component and various Group 11 or Group 12 metals as the d^10^ part.^28,29^ In particular, we had measured the bond dissociation energy of the [*cis*-(bhq)_2_Pd^II^–Cu^I^(IPr)^+^] complex **1**^**+**^, where bhq is the 1,10-benzo[*h*]quinolinato ligand and IPr is the *N*-heterocyclic carbene (NHC) ligand, 1,3-bis(2,6-diisopropylphenyl)imidazol-2-ylidene.^30^ As stable analogs of the transition states for transmetalation step in Pd-catalyzed Sonogashira and Negishi coupling reactions, the thermochemistry of the model heterobimetallic complexes provides, in principle, concrete, experimental values for the d^8^–d^10^ metal–metal interaction that purportedly stabilizes the actual transition states in the catalytic cross-coupling reactions.

Accordingly, we were surprised and concerned by the unacceptably large discrepancy between the experimentally measured bond dissociation energies and the corresponding values computed with the tested DFT methods. A particularly egregious case was presented by **1**^**+**^, for which the fitting of a threshold collision-induced dissociation (T-CID) curve with our deconvolution program, L-CID, gave 50.6 ± 1.4 kcal/mol, which contrasted sharply with dispersion-corrected DFT-computed values between 70 and 80 kcal/mol (depending on exchange-correlation functional, see SI, §3.1).^30^ As one of the ways we validated the derived bond strength extracted from T-CID data by deconvolution with a model built on approximate statistical rate theory,^44−46^ we designed a second experiment with a slightly different heterobimetallic complex, **1a**^**+**^, which differed from **1**^**+**^ only in having a prosthetic side chain, remote from the metal–metal bond, which additionally contained a cleavable C–C bond, whose well-characterized bond dissociation energy of 61.4 ± 1.3 kcal/mol^47^ was bracketed by the previous, “low,” experimental Pd^II^–Cu^I^ bond strength of 50.6 ± 1.4 kcal/mol, and the “high” computational predictions of 70–80 kcal/mol.^33^ The cleavage of the Pd^II^–Cu^I^ bond in complex **1a**^**+**^ could therefore be calibrated against the known C–C bond strength in **1a**^**+**^ in an intramolecular competition which gave a clear result that the Pd^II^–Cu^I^ bond is significantly weaker than the C–C bond, consistent with the “low” value for the Pd^II^–Cu^I^ bond, and ruling out the “high” value from DFT, which, naturally, suggests consequences for the credibility of DFT predictions for the transition states for the transmetalation step in Sonogashira and Negishi coupling reactions.

While the previous work emphasized the reliability (or lack thereof) of the experimental and/or computational methods, and the preponderance of evidence supported the experimental determination, neither the experiment nor the calculations identified unambiguously the physical origin of the unacceptably large discrepancy between measured and computed d^8^–d^10^ metal–metal bond dissociation energies. There is, of course, a significant body of literature documenting the difficulties in computing metal–metal interactions.^25^ While we had indeed considered multiple, possible causes, the most obvious interpretation of the discrepancy started with the metal–metal bond. Our new experimental results, in which some, *but not all* systems, show large discrepancies, call the obvious interpretation into question. In the interest of expanding the scope of the structural and thermochemical data beyond the Pd^II^–Cu^I^ complexes, **1**^**+**^ and **1a**^**+**^, we prepared first the analogs with Pd^II^–Ag^I^ and Pd^II^–Au^I^, designated **2**^**+**^ and **3**^**+**^, respectively, as well as several analogs with Pd^II^–Zn^II^ bonds, designated **4**^**+**^, **5**^**+**^, and **6**^**+**^. All of the complexes share the same d^8^–d^10^ metal–metal interaction, and all complexes **1**^**+**^ to **6**^**+**^ are isostructural, as documented by single-crystal X-ray diffraction, albeit with less bridging in the Pd^II^–Zn^II^ systems. The latter series, with Zn(II), was prepared especially as models for the transition state for transmetalation in the Negishi coupling. In particular, the Zn(II) center carries partially and fully fluorinated aryl groups to make the stable, isolable Pd^II^–Zn^II^ complex as closely structurally analogous as possible to the transition states for the transmetalation reactions investigated by Espinet and Casares.^9^ Whereas we had previously reported the structurally characterized, neutral complex, [*cis*-(bhq)_2_Pd^II^–Zn^II^(C_6_F_5_)_2_], it had proven unsuitable for gas phase studies by electrospray mass spectrometry on account of the lack of a permanent charge. The deficiency was remedied in **4**^**+**^, **5**^**+**^, and **6**^**+**^ by attachment of a prosthetic charged group, a pendant quaternary ammonium center, at the 5-position of one of the bhq ligands on Pd. As documented in the Experimental Section and Results sections, the synthetic effort to prepare the modified ligand, and then prepare the heteroleptic *cis*-(bhq)[5-(trimethylammoniummethyl)bhq]Pd^II^ complex, **7**^**+**^, was significant, but we were rewarded with its ready conversion to the target structures **4**^**+**^, **5**^**+**^, and **6**^**+**^, which proved eminently suitable for electrospray. Threshold collision-induced dissociation (T-CID) of the electrosprayed heterobimetallic complexes yielded the d^8^–d^10^ bond dissociation energies in Table 4. Even without further detailed analysis, a brief perusal of the experimental BDEs shows an unexpected, and striking, dichotomy. For the heterobimetallic complexes **1**^**+**^, **2**^**+**^ and **3**^**+**^, the discrepancy between the experimentally determined d^8^–d^10^ bond BDE, and the dispersion-corrected DFT value, is surprisingly constant and systematic, at −19 ± 1 kcal/mol, regardless of the d^10^ component, Cu(I), Ag (I), or Au(I), the experimental value being lower than the DFT-computed one. In marked contrast, **4**^**+**^, **5**^**+**^, and **6**^**+**^, all with the isoelectronic Zn(II) as the d^10^ component, but differing in the aryl groups on the Zn, do show a systematic discrepancy, but an order of magnitude smaller, and in the opposite direction, amounting to merely +2 to +3 kcal/mol. Given that the technical issues, in particular the kinetic shift,^48,49^ in extracting reliable BDEs from threshold CID curves become increasingly challenging as the molecule becomes larger, i.e., more internal degrees-of-freedom, much of our recent control work had focused on verifying that the approximations made to extract the BDE remain valid for molecular ions as large as the d^8^–d^10^ heterobimetallic complexes.^34,35,45,46^ The large discrepancy for **1**^**+**^–**3**^**+**^, and the, frankly, unexpectedly small discrepancy (coming close to the stated experimental uncertainty) for **4**^**+**^–**6**^**+**^, constitute the fundamental set of results which needs to be explained, presumably (hopefully) telling us something more general about the reliability of experimental and computational determinations of noncovalent interactions in molecules when the molecules become large.

The first question one must ask is: Do we believe the experimental *E*_0_ determinations? If one were to answer the query in the affirmative, it necessarily implies that there is something wrong with the computational method, which sometimes, but not always, produces a large discrepancy. As “sometimes right, sometimes wrong” would be even more disturbing than a systematic, but at least consistent, discrepancy, we sought alternative hypotheses that might explain the data. More than just straw men, the alternative hypotheses would call into question either the interpretation of the experimental data, or even, in some cases, the data themselves. We seek control experiments or arguments with external data that can exclude (or not) each alternative hypothesis.

**Figure 8:**
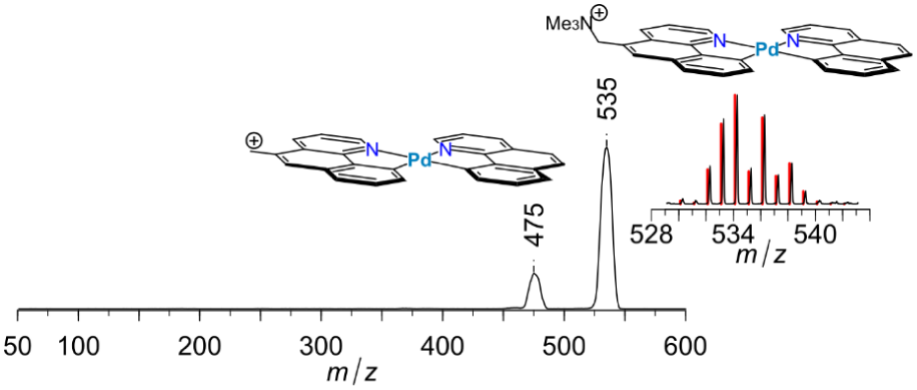
Collision-induced dissociation of **7**^**+**^ produces clean loss of trimethylamine, producing a benzylic fragment ion. ESI-MS/MS CID spectrum of ion , **7**, with 110 μTorr of Ar collision gas in the collision cell at collision offset of 50 V. Inset: experimental (black) and simulated (red) isotopic pattern of parent ion. Proposed structures for both parent and fragment ions are shown. Marked numbers refer to *m*/*z* of the peak maxima. Experimental gas-phase BDE for the C–N cleavage was measured using the T-CID/L-CID method, all fitted parameters are listed in the SI. The lower resolution of the CID spectrum relative to the original mass spectrum is a common feature in mass spectrometry, the second quadrupole in a triple quad instrument being optimized for signal intensity, while the first is optimized for resolution.

Alternative Hypothesis 1. L-CID fails to extract *E*_0_ reliably when the molecules are large. The principal methodological problem with measuring bond dissociation energies for large molecules in the gas phase is the kinetic shift.^44,48,49^ The problem had been acknowledged for decades, with more than one solution having been presented over the years.^50−55^ The present work uses the program L-CID, which approximates the density-of-state function over a wide range in internal energies.^44^ The key approximation in L-CID itself was benchmarked against Beyer–Swinehart explicit state counting.^56^ Furthermore, the approximation has been tested more recently against experimentally determined microcanonical dissociation rates for medium-sized ions, themselves used to compute *E*_0_ with more sophisticated statistical rate methods.^45^ Lastly, the validity, or, at least, the (in)sensitivity of the L-CID treatment of other aspects of the overall dissociation process, e.g., the collision cross-section or the energy transfer in a collision, has been benchmarked against explicit physical simulations.^46^ None of these benchmarking exercises indicated that L-CID would produce discrepancies large enough to be of concern. Of perhaps more immediate relevance is a control experiment we executed on **7**^**+**^, the intermediate in the synthesis of **4**^**+**^–**6**^**+**^. The complex, ^+^, [**7**^**+**^]BArF^–^, electrosprays well to produce cation **7**^**+**^ in the gas phase. Complete data may be found in the SI, §2.4. Collision-induced dissociation of **7**^**+**^ leads to clean cleavage of the C–N covalent bond, producing neutral trimethylamine and a benzylic cation, as may be seen in Figure 8.

Fitting the T-CID curve of **7**^**+**^ with L-CID yields *E*_0_ = 40.8 ± 1.1 kcal/mol for the C–N bond dissociation energy, which closely matches the reported bond dissociation energy of the closely related, and much smaller, benzylammonium cation, *E*_0_ = 39.7 ± 0.7 kcal/mol.^57^ The following logic is analogous to that for the widely used ″thermometer″ ions that calibrate bond strength measurements in mass spectrometric breakdown curves.^50^ According to the logic, there is no reason *a priori* to expect the actual *E*_0_ values for **7**^**+**^ and benzylammonium cation to differ significantly from each other. The similarity in the values extracted by L-CID suggests, again, that the scaling of the T-CID method from small molecules, where the bond energies are indisputable, to a much larger one, where the methodology could have been called into questioned, works properly. Furthermore, in the CID spectrum of **4**^**+**^, Figure 6, a yet larger ion, the principal dissociation channel, loss of (C_6_F_5_)_2_Zn, is accompanied by a minor channel, loss of trimethylamine, when the collision energy is high, i.e., much over threshold, which is the condition under which reactive cross sections for competing product channels tend to equalize. The relative intensities of the major and minor product peaks indicates (qualitatively) that *E*_0_ for the minor channel must be only a few kcal/mol higher than that for the major channel.^51,58^ The L-CID fit for the major channel gave *E*_0_ = 35.2 ± 1.0 kcal/mol for cleavage of the Pd–Zn bond, which is indeed just a few kcal/mol lower than the putative C–N bond dissociation energy of *E*_0_ = 39–41 kcal/mol. Especially since the experimentally determined *E*_0_ values for **4**^**+**^–**6**^**+**^ do agree acceptably, e.g., to within 3 kcal/mol (albeit with the difference always in the same direction,) with the PBE-D3(BJ)/def2-CBS(3,4) predictions, it appears that one would say that there is sufficient evidence to accept that the experimental *E*_0_ values for **4**^**+**^–**6**^**+**^ are, in fact, correct. Taking the argument one step further, we would argue that T-CID data, deconvoluted with L-CID, should produce correct *E*_0_ values for the isostructural complexes, **1**^**+**^–**3**^**+**^ as well. The argument discounts the first alternative hypothesis.

Alternative Hypothesis 2. Dissociation is accompanied by isomerization to lower energy structures of the same *m*/*z* ratio. This second alternative hypothesis would postulate that the fragments from complexes **1**^**+**^–**3**^**+**^ differ from those from **4**^**+**^–**6**^**+**^ in an important way, and that the discrepancy in the *E*_0_ for the former arises because their dissociation does not proceed to the expected products. Aside from the charge tag, the difference between **1**^**+**^–**3**^**+**^ and **4**^**+**^–**6**^**+**^ consists in the d^10^ metal and its ligands, e.g. IPr versus fluoroaryls. Because the parent ion is mass-selected, and the fragment ions are detected by mass spectrometry, a confounding isomerization would have to occur prior to, or during, the dissociation event, and produce fragments of the same *m*/*z* ratio. An isomerization in a distinct step subsequent to the dissociation would not affect the measured *E*_0_. Given the known chemistry of Cu(I), Ag(I), and Au(I), the likely isomerization would be either an intramolecular C–H or C–C activation step occurring as the d^10^ fragment separates from the Pd(II) center. There is, in fact, precedent for rearrangements in the long-lived ion-neutral complexes (INC),^59,60^ out of which dissociation over a loose, orbiting transition state takes place.^61,62^ One may conceive that the formally monovalent, hence coordinatively unsaturated, d^10^ metal center in the dissociating complex **1**^**+**^–**3**^**+**^ might be prone to undergo an oxidative insertion into a C–H or C–C bond of the IPr ligand, analogous to that in the roll-over cyclometalation reported by Schwarz and coworkers,^63^ or even our own early reports of cyclometalation in gas-phase Ir(III) complexes.^64,65^ To confound the *E*_0_ measurement, the intramolecular oxidative addition must occur concurrently with the dissociation of **1**^**+**^–**3**^**+**^, but, more importantly, the isomerized product must be lower in energy than the expected M(IPr)^+^ fragment. As documented in the SI, §3.2, a computational examination of all plausible C–H and C–C intramolecular oxidative addition products finds no structures more stable than the expected M(IPr)^+^ fragments. We also point out that, while it would have been plausible that Cu(I) might be prone to an oxidative addition, the reaction is well-known to be less favorable for Ag(I) and Au(I).^66,67^ While the measured *E*_0_ values do follow the expected Cu ∼ Au > Ag pattern,^68^ the discrepancy between the experimental and the computed bond dissociation energies is close to constant across the series, which argues against a putative process relying on an oxidative addition on the metal center. In any case, the computational study also finds no evidence for isomerization to a more stable product of the same *m*/*z* ratio, which, together with the argument on periodic trends above, discounts the second alternative hypothesis.

Alternative Hypothesis 3. The Pd–Cu, Pd–Ag, and Pd–Au complexes, on the one hand, and the Pd–Zn complexes, on the other, are not themselves structurally homologous. Another way that the two series, **1**^**+**^–**3**^**+**^ and **4**^**+**^–**6**^**+**^, might yet be different is in the nature of the d^8^–d^10^ bond to be broken. To the extent that the single-crystal XRD structures also represent the structures in the gas phase, the key structural parameters in Tables 2 and 3 show some differences between **1**^**+**^–**3**^**+**^ and [(bhq)_2_Pd–Zn(C_6_H_5_)_2_], the neutral antecedent for **4**^**+**^–**6**^**+**^. In particular, the Pd–Zn system displays noticeably less bridging. According to the discussion from Puddephatt, the largest contribution to the interaction looks like a dative bond between either a doubly occupied , or a doubly occupied 4d_*xz*_, orbital on Pd(II), depending on whether the one or the other is the HOMO,^69^ and an empty n*s* orbital, the LUMO, on Cu(I), Ag(I), Au(I), or Zn(II).^70^ The degree to which the  or the 4d_*xz*_ orbital dominates should determine the degree of bridging. The same HOMO–LUMO interaction appears in computational studies of the protonolysis of Pt-alkyls, the d^10^ component in transmetalation being isolobal to a proton, whose unfilled *s*-orbital interacts with the filled d-orbitals on the d^8^ complex to make either a classical or bridging hydride, depending on which d-orbital is the HOMO.^71,72^ Nevertheless, in collision-induced dissociation experiments in a mass spectrometer, the electrostatic charge/dipole or charge/induced dipole interaction means that the dissociation typically occurs from the ion-neutral complex (INC),^59,60^ a relatively long-lived electrostatic complex, often proceeding by what is often called in the literature an orbiting transition state.^61,62^ The nature of the prior bonding should not make any difference as long as it does not lead to a reverse barrier in the exit channel, which would have to be extremely unusual, given the depth of the electrostatic potential well out of which the products must escape.^73,74^ In connection with any claim of a meaningful difference in bonding, we do note that the computed structures for Pd–Zn complexes, **4**^**+**^–**6**^**+**^, optimized with PBE-D3(BJ)/def2-TZVP, have significantly less bridging than is actually observed in the single-crystal XRD structure of [(bhq)_2_Pd–Zn(C_6_H_5_)_2_], the most diagnostic parameter being the M–Pd-C1 angle (Tables 2 and 3), which for **1**^**+**^–**3**^**+**^, varies in the range 51–53°, indicating bridging, and which is computed for **4**^**+**^–**6**^**+**^ to vary from 73 to 84°, indicative, as mentioned above, of much less bridging. The single experimental XRD structure for the Pd–Zn series, however, is for [(bhq)_2_Pd–Zn(C_6_H_5_)_2_], out of which one reads an angle of 63.30°, which is not so far from the angles for **1**^**+**^–**3**^**+**^. Moreover, as mentioned above, the energy decomposition analysis of the bonding in the heterobimetallic systems with the ETS-NOCV method^75^ found broadly similar bonding across all of the complexes we studied. See SI, §3.1.3. The Zn–C bond distance and Zn–Pd–C1 angle are the most informative, physically measurable diagnostics for a bonding interaction, which argue, in fact, for a close similarity of the bonding in **4**^**+**^–**6**^**+**^ to that in **1**^**+**^–**3**^**+**^. We do not believe that the third alternative hypothesis to be plausible.

Accordingly, given the new observations, and given the control studies we have already reported, we believe that it is justified to claim that the extracted BDEs do, in fact, represent accurately the thermochemistry of the heterobimetallic d^8^–d^10^ complexes. We are left with a disturbingly persistent, but, now, not universal, discrepancy between experiment and dispersion-corrected DFT, which necessarily implies that the employed theory has some, as-yet unrecognized, deficiency.

Having excluded the potentially confounding alternative hypotheses, we argue now that we should accept the experimental BDEs at face value. The next logical step would be to consider a number of potential causes for a persistent, but not universal, discrepancy in the predictions from theory. Given that the literature contains relatively frequent claims that metal–metal interactions are problematic for computational chemistry,^25^ we considered three possible issues which could have contributed to the discrepancy: multireference character in the metal–metal bond, relativistic effects, and poorly treated electrostatic effects. For the first, we could quickly ascertain that the d^8^–d^10^ bonds in the entire series from **1**^**+**^ to **6**^**+**^ are dominated by closed-shell interactions. The extent of multireference character can be described by the T1 and T2 metrics for coupled cluster calculations, and B1 and the fractional orbital density for DFT methods.^76^ Not only do the metrics indicate little multireference character, but there is also no indication that Cu(I) should be different from Zn(II), for example, when comparing **1**^**+**^ to **4**^**+**^. Relativistic effects on bond dissociation energies in transition metals systems have long been known to be significant, with the most striking case being the bond in small cations, like AuCH_2_^+^, for which relativistic effects were found to account for a surprisingly large 70% of the metal–carbon bond strength.^77^ Going beyond “small” molecules, one may expect increasing challenges in treating relativistic effects. Nevertheless, the contribution to bond strengths in Pd complexes has been claimed to be modest,^78,79^ and, furthermore, the trend in the BDEs for **1**^**+**^, **2**^**+**^ and **3**^**+**^, i.e., Pd^II^–Cu^I^, Pd^II^–Ag^I^, and Pd^II^–Au^I^, of Cu ∼ Au > Ag,^68^ typically attributed to relativistic effects, is seen in both the experimental numbers as well as the computed ones, albeit with a constant offset. If the offset itself, rather than merely the Cu ∼ Au > Ag ordering of bond energies, were to be attributable to relativity, one might expect that it would become worse as one went from Cu to Ag to Au. The near invariance of the discrepancy in this series **1**^**+**^, **2**^**+**^ and **3**^**+**^ argues against relativistic effects as the cause. To test the last possibility, poorly treated electrostatic effects, we attempted to find correlations of the magnitude of discrepancies with the partial charge on the metal centers. While simple in conception, and inspired by the difference in formal oxidation state, either +1 or +2, of the d^10^ metal, the assignment of partial charges in molecules, in general, remains inconsistent. This particular issue is not unique to the present problem; it has been thoroughly documented in the literature around classical molecular dynamics simulations, for which the partial charges represent essential, atom-centered parameters for intra- and intermolecular interactions.^80^ For example, while there are instances where Mulliken charges, computed with a minimal basis set, provide intuitively realistic values, the values get worse with a larger basis set, which suggests fortuitous error cancelation. The more sophisticated Hirschfeld partitioning, in our hands, provided, however, no clear correlation with the size of the discrepancy. We settled on Bickelhaupt’s extended transition state natural orbitals for chemical valence (ETS-NOCV) approach, a specific instance of more general energy decomposition analysis (EDA),^75^ as a method with which we have had good experience in previous studies. The ETS-NOCV analysis partitions the interaction energy between two fragments into contributions, among which the electrostatic interaction is one. Comparing the ETS-NOCV partitioning of the interaction energies (SI, §3.1.3) in **1**^**+**^–**3**^**+**^ versus **4**^**+**^–**6**^**+**^ finds no identifiable trend correlating to the discrepancy between experimental and computed bond dissociation energies.

**Figure 9:**
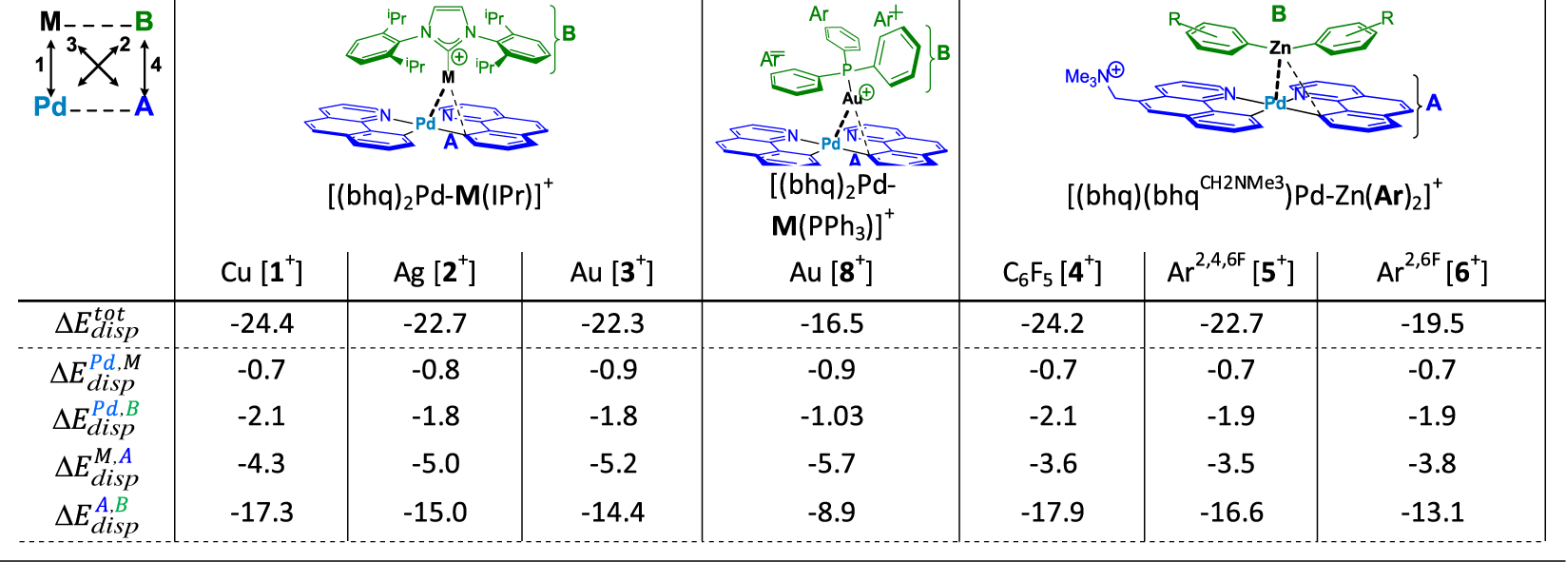
Partition of the atom-pairwise D3 correction in PBE-D3(BJ) calculations of **1**^**+**^ to **6**^**+**^ into contributions attributable to pairwise interactions between subsystems within each of the complexes. All values are in kcal/mol.

A plausible correlation to the observed discrepancy between the computed bond dissociation energies and the values extracted from T-CID experiments comes from an altogether different direction. The usual density functional theory methods, *based on the local density expansion*, cannot describe long-range, inherently nonlocal interactions, of which London dispersion, for example, is one particular instance.^81^ The importance of London dispersion forces in the structure and stability of medium-to-large organic and organometallic molecules, already noted by Zhao and Truhlar,^82^ has been more recently highlighted by Schreiner and Wagner.^83^ Among the many methods proposed to ameliorate the recognized deficiency in DFT for London dispersion, as well as other nonbonded interactions, including medium-range correlation effects, the DFT-D*x* (*x* = 1–4) methods by Grimme et al.^84^ have found particular favor among practitioners of computational chemistry because the *post facto* correction adds essentially no time to the DFT calculation. The method introduces a correction as a sum of atom-pairwise contributions, with C_6_ (and also C_8_) coefficients for each element extracted from the molecular polarizability tensor in diatomic molecules built with each of the elements in the periodic table, and a damping function which becomes important at shorter distances. The ease-of-use of the DFT-Dx methods, and the favorable scaling of DFT, in general, made the DFT-Dx methods often the first choice in treating large molecules with many intra- and intermolecular interactions. The atom-pairwise construction of the correction offers, however, another advantage in the present circumstance. While the extent of the nonbonded interactions captured by any particular exchange-correlation functional may vary, one can nevertheless use the atom-pairwise construction of the correction to partition the overall correction into contributions attributable to pairs of subsystems within a molecule, at least semiquantitatively. The results of just such an exercise appear in Figure 9. We divide complexes **1**^**+**^–**6**^**+**^ and **8**^**+**^, into the d^8^ and the d^10^ halves, and then further divide each of the halves into the metal center and the ligand. We use PBE-D3(BJ), a typical exchange-correlation functional, which neglects a large part of nonbonded interactions before the D3 correction, which means, necessarily, that much, or most, of the nonbonded interaction appears in that D3 correction.^85^ Taking the overall D3 correction apart, which can be done because it built up atom-pairwise, we find that, for **1**^**+**^ to **6**^**+**^, the component of the D3 correction attributable to the Pd(II) center, interacting with the Cu(I), Ag(I), Au(I) or Zn(II) center, remains below 1 kcal/mol for all of the complexes. In fact, the only large, with “large” meaning much greater than 5 kcal/mol, contribution is the component attributable to an interaction between the ligand on Pd(II) and the ligand on Cu(I), Ag(I), Au(I) or Zn(II).

A putative overbinding by DFT-D3, in cases where there is a discrepancy relative to the experimental BDE, means that the computed value of some component in the interaction would have to be too large in absolute magnitude. Contributions that themselves have an absolute magnitude of only 1 kcal/mol in the correction, for example, are therefore too small to explain much of an observed discrepancy of 19 kcal/mol. In making this argument, one should remain cognizant, of course, that not quite all of the nonbonded interaction is captured by the D3 correction alone (the exchange-correlation functional has some of it), but, as long as most of it is in the D3 correction, and the differences are large enough, one may nevertheless proceed with the argument. This argument, if supported by further data, would suggest that a large part, if not most, of the discrepancy between PBE-D3(BJ), and by extension, the other tested DFT methods that gave similar BDEs (see SI, §3.1 and §4.2) and experiment comes from a problem in the description of the interaction between the ligands, because that component is the only one which is large enough in absolute magnitude to matter. We do not exclude a residual error in the DFT-D3 description of the metal–metal bond itself, but the case of **4**^**+**^–**6**^**+**^ indicates that any residual discrepancy appears likely to be small enough in magnitude that our data, with the stated experimental uncertainty, cannot yet make a definite conclusion.

This new hypothesis for the discrepancy requires a recognizable difference between the interligand interactions in **1**^**+**^–**3**^**+**^ versus **4**^**+**^–**6**^**+**^. Perusal of the structures does indeed find an important difference. In **4**^**+**^–**6**^**+**^, the aryl groups on the Zn(II) center and the 1,10-benzo[*h*]quinolinato ligand on Pd(II) are not so far from face-to-face parallel, and, furthermore, not so close as to be in the repulsive part of the potential. The centroid-to-centroid distances between the two aryls on Zn(II) and the 1,10-benzo[*h*]quinolinato ligand(s) facing them in **4**^**+**^ are 3.6 and 3.7 Å, which can be compared to an optimal face-to-face π-stacking distance of just about 4 Å, for example, in [4.4]-paracyclophane, the first of the paracyclophanes whose sufficiently long and sufficiently flexible linkers allow the phenyl rings to remain planar and assume their preferred distance at the bottom of the attractive potential.^86^ For complexes **1**^**+**^–**3**^**+**^, in contrast, the principal interaction is between the methyl groups on the isopropyl side chains of the 1,3-bis(2,6-diisopropylphenyl)imidazol-2-ylidene ligand on Cu(I), Ag(I), or Au(I) with the face of the aromatic 1,10-benzo[*h*]quinolinato ligand. With the isopropyl groups necessarily turned orthogonal to the plane of the appended aromatic ring, a total of four methyl groups are thrust straight down into close proximity with the aromatic below, as may be seen in the X-ray structures of **1**^**+**^–**3**^**+**^. In **1**^**+**^, for example, the closest distances from one of the hydrogens on the methyl groups to the bhq plane are approximately 2.5 Å in the X-ray structure. In addition, the core of the IPr ligand on Cu(I), Ag(I), or Au(I) in **1**^**+**^–**3**^**+**^ is comprised of an aromatic heterocyclic moiety oriented approximately perpendicular to the plane of the 1,10-benzo[*h*]quinolinato ligands on Pd(II), whose ″edge″ is albeit farther away than the alkyl groups, but still close enough for a noncovalent interaction. For example, in **1**^**+**^, the aryl–aryl, edge-to-face distance is approximately 5.7 Å. The latter may yet be important, considering the predicted bond dissociation energies for [(bhq)_2_Pd–Cu(imidazol-2-ylidene)]^+^ complexes for which the isopropyl groups of IPr are replaced with hydrogens in the computed structures.^76^

**Figure 10:**
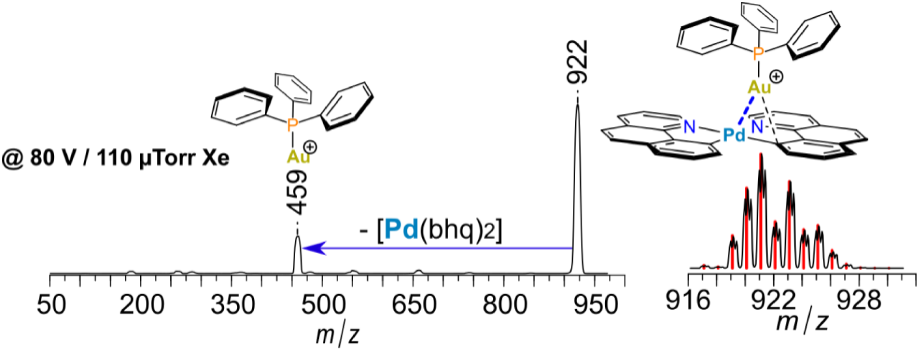
Collision-induced dissociation (CID) of the [(bhq)_2_Pd][Au(PPh_3_)^+^] complex, **9**^**+**^, from which the T-CID experiment gives *E*_0_= 56.3 ± 2.2 kcal/mol, which can be compared to the PBE-D3(BJ)/def2-CBS(3,4) prediction of 59.5 kcal/mol.

Motivated to check the hypothesis experimentally, we prepared an additional complex, [(bhq)_2_Pd–Au(PPh_3_)]^+^, **8**^**+**^, whose central Au–Pd–C1 geometry closely resembles that for **3**^**+**^ (with a computed structure for **8**^**+**^ in the SI, §3.1), but whose ligand, PPh_3_, has neither alkyl groups nor the heteroaromatic core of IPr. **8**^**+**^ is missing the isopropyl groups on **3**^**+**^, and the three phenyl groups are constrained sterically to take a propeller-like geometry, introducing one partial (tilted) edge-to-face phenyl, one face-to-face phenyl, and one phenyl pointing out between the two bhq ligands, with the proviso that we expect the interactions to be weaker because the phenyls are one bond further away from the bhq ligand(s) and tilted “up” and away, as compared to the case in **3**^**+**^. Subjecting **8**^**+**^ to the T-CID experiment, and extracting the bond dissociation energy with L-CID, yields *E*_0_ = 56.3 ± 2.2 kcal/mol, Figure 10, which is only slightly larger than the value for **3**^**+**^ in Table 4. Importantly, the computed bond dissociation energy for **8**^**+**^, again with PBE-D3(BJ)/def2-CBS(3,4)//PBE-D3(BJ)/def2-TZVP, is 59.5 kcal/mol, which deviates from the experimental value by only −3 kcal/mol rather than the −20 kcal/mol discrepancy exhibited by **3**^**+**^. For the Pd–Zn systems, **4**^**+**^–**6**^**+**^, if we regard the performance of PBE-D3(BJ) to indicate a slight underestimate of the face-to-face interactions, the small difference for **8**^**+**^, in the opposite direction, suggests that perhaps the one edge-to-face interaction may yet be overestimated, but not by so much as to cause a gross discrepancy. We do note, as a side comment, that the reasonably small discrepancy between experiment and computation for **8**^**+**^ means that, even for the d^8^–d^10^ heterobimetallic complexes with Pd(II) as the d^8^ component and a Group 11 metal as the d^10^ component, Au in this case, the description of the metal–metal interaction itself by DFT-D3 appears to be not too bad.

**Figure 11:**
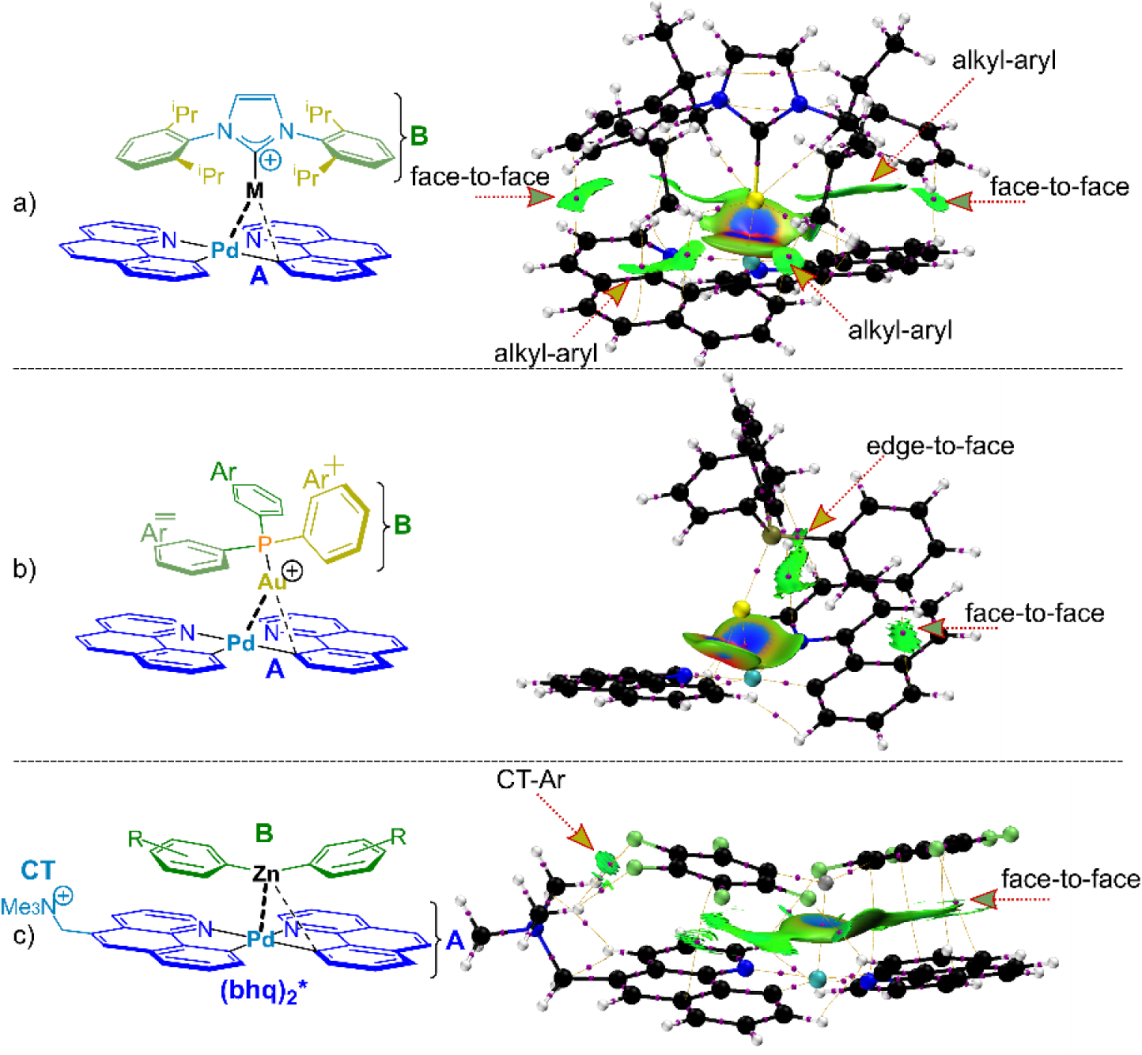
IGMH analysis performed using Multiwfn Version 3.8(dev), and densities obtained with DKH2-M06-D30/DKH-def2-TZVP//PBE-D3(BJ)/def2-TZVP (Section S3.1.3, Tables SI-3-1 and Table SI-3-2). Sign(λ_2_)ρ colored isosurfaces of δ*g*^inter^ = 0.0025 au corresponding to IGMH analyses for shown ions (a) **3**^**+**^, (b) **8**^**+**^, (c) **4**^**+**^. Specified Fragments A and B shown in structures (left). The coloring method of sign(λ_2_)ρ is as follows: (blue) prominent attractive weak interaction, (green) van der Waals interaction, red) prominent repulsive interaction (steric effect in ring etc.). Different interactions are marked with arrows. (for input file for Multiwfn analysis see Supporting Information). Additionally the bond critical points, according to AIM theory, and the bond-paths are shown.

A significant ligand–ligand interaction is also supported, in fact, by an Independent Gradient Model (IGMH) analysis based on Hirschfeld partitioning of the charges.^87^ The method visualizes noncovalent interactions, and produces plots similar to those from NCI.^88^Figure 11 shows color-coded isosurfaces with weak noncovalent interactions represented in green.

**Figure 12:**
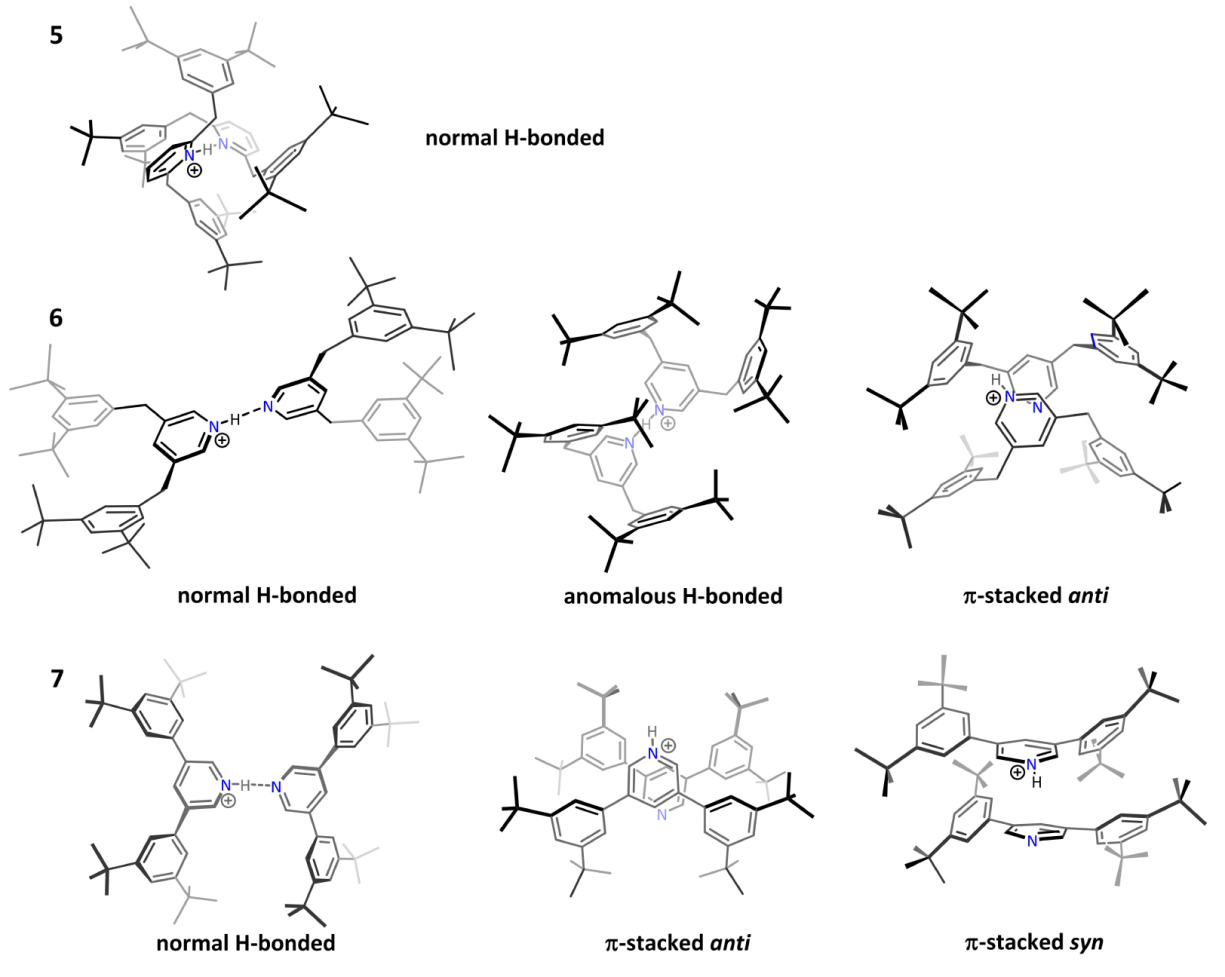
Structures of proton-bound dimers, reproduced from Figure 2 of ref. (35), copyright 2020 American Chemical Society. The principal noncovalent contributions to the dissociation energy in the normal H-bonded structures for the ortho dimer (top row) are edge-to-face aryl–aryl and alkyl-to-aryl face, whereas the they are face-to-face aryl–aryl for the anomalous H-bonded and π-stacked meta dimers.

In our original report of a discrepancy between experimentally determined BDEs and those calculated with B97D3, the primary interactions were edge-to-face aryl–aryl and alkyl-to-aryl face.^34^ We encountered comparably large discrepancies, with the BDE computed with B97D3 exceeding the experimental value by about 10 kcal/mol. In that report, an intramolecular competition experiment analogous to that with **1a**^**+**^ ruled out the higher B97D3 value, as well. In subsequent work, which we undertook broadly as a control for the prior work, we reported gas-phase BDEs for proton-bound dimers of pyridines with the substituents moved from the *ortho* positions, from which they could interact, to the *meta* and *para* positions, from which they designed to be out-of-reach for interaction if the proton-bonded dimers were to remain isostructural.^35^ The principal conclusion of the control experiments, taken from cases with the substituents on the *para* position, was that the extraction of BDEs from T-CID experiments worked reliably—the experimental numbers were good. In the course of the control experiments, we did determine that some of the *meta*-substituted proton-bound dimers did not appear to be isostructural. Figure 2 in that publication, reproduced here as Figure 12, showed the “normal H-bonded,” “anomalous H-bonded,” and “π-stacked” structures. The expected “normal H-bonded” structure was the lowest energy structure for the *ortho* dimers used in the original BDE studies, but the “anomalous H-bonded” or “π-stacked” structures were computed to be lower in energy for the *meta* dimers. Importantly, for one of the *meta* dimers, we confirmed the “π-stacked” structure spectroscopically in the gas phase. Because the structures were no longer isostructural with the *ortho* dimer, we could not make comparisons in the series with the *meta* dimers, but we did report the BDEs.^35^ Relevant for the present suggestion—in fact, inspiring it—is the observation that the experimentally measured dissociation energies of the *meta* dimers, with face-to-face aryl–aryl interactions, agreed quite satisfactorily with the B97D3 predictions, in contrast to the large discrepancy for the *ortho* dimer.

**Figure 13:**
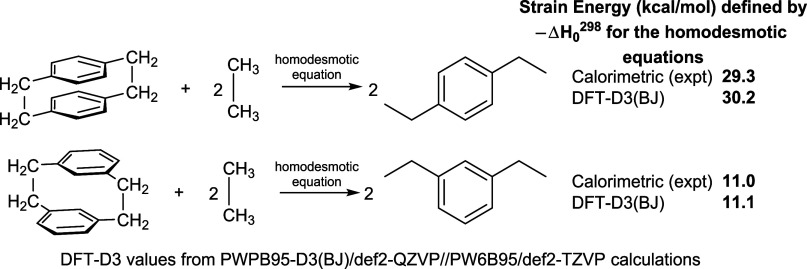
Comparison of strain energies, defined by homodesmotic reactions, for [2.2]-paracyclophane and [2.2]-metacyclophane, obtained with PWPB95-D3(BJ)/def2-QZVP//PW6B95/def2-TZVP calculations, against the strain energies obtained from gas-phase heats of formation from experimental calorimetry.

If we now presume that the d^8^–d^10^ metal–metal interaction is described by PBE-D3(BJ) adequately enough, then the dichotomy between **1**^**+**^–**3**^**+**^ versus **4**^**+**^–**6**^**+**^ completely parallels that between the *ortho* versus *meta* dimers in the previous work. In both cases, it appears that DFT-D3 overestimates the alkyl-to-aryl face and/or the aryl–aryl, edge-to-face interactions, but that it describes aryl–aryl, face-to-face interactions well enough. An indication that the latter part is correct may be found in the benchmarking study using the strain energy in various cyclophanes.^89^ Strain energy is defined as the difference in enthalpy between a cyclophane and appropriate reference molecules defined in a homodesmotic reaction.^90^ Whereas the original benchmarking study compared the DFT-D3-derived strain energies, with several functionals, to strain energies computed with high-level wave function methods, it did not take advantage of experimentally determined calorimetric data that were available.^91−94^ The comparison to calorimetric data is shown in Figure 13. The particular comparison is unique in that the quality of the DFT-D3(BJ) calculation—for the cited numbers, PWPB95-D3(BJ)/def2-QZVP//PW6B95/def2-TZVP but similar for other functionals—of aryl–aryl, face-to-face interactions can be compared to wholly experimental, gas-phase thermochemistry obtained without any computational deconvolution of solvation free energies.^95^ The latter deconvolution, typically done with generalized Born models, i.e., treatment of solvent as a polarizable continuum, introduces large, and not completely predictable, uncertainties into the benchmarking studies for DFT-D3 on large molecules, and, especially, large charged molecules. To illustrate the problem, consider that the absolute free energies of solvation for ions are 1–2 orders-of-magnitude larger than those for comparably sized neutral molecules, with ionic solvation energies reaching ∼100 kcal/mol for small ions in polar solvent.^96^ Even the original Born equation predicts these orders-of-magnitude, if one considers that the partial charge on a center goes into the solvation energy quadratically.^97^ Accordingly, an error in the calculation of solvation free energy, for example, 25%, does not necessarily compromise the accuracy of the computational prediction when the absolute magnitude of the solvation energy is only 5 kcal/mol, as one finds for many neutral molecules. The same 25% error for an ionic solvation energy of 100 kcal/mol would be egregious. Accordingly, benchmarking of DFT-D3 for gas-phase interaction energies by deconvoluting solution binding constants computationally is uncertain, particularly when the species are charged.^98^ We highlight the benchmark with the cyclophanes because the experimental data for the comparison consist of heats of combustion and heats of vaporization (sublimation), which allow the derivation of wholly experimental heats of formation for [2.2]-paracyclophane and [2.2]-metacyclophane.^92−94^ with well-defined interaction geometries. We find the striking agreement for the cyclophanes to be credible, and it indicates that, for the aryl–aryl, face-to-face interactions in the *para*-cyclophanes, DFT-D3 performed very well, indeed. In principle, DFT-D3 could also be benchmarked with the experimentally determined binding energy of planar aromatic hydrocarbons to a graphite surface, measured to be 52 meV per carbon by thermal desorption spectrometry.^99^ As noted in that report, various semiempirical and density functional methods had produced estimates between 8 and 170 meV per carbon atom, which highlights a great sensitivity of the computational result to the method employed.

Our previous report had included an in-depth discussion of possible origins for the dichotomous behavior of the D3 correction,^35^ from which the most likely ones are the dependence on hybridization, and the anisotropy of the C_6_ coefficients.^100^ As we had briefly summarized above, the atom-pairwise treatment of the correction in DFT-D3 with the molecular polarizability tensor taken from diatomics cannot treat hybridization of a carbon center properly, and necessarily forces the C_6_ coefficients to be isotropic. Experience from chemical practice, as well as precedent from other related physical phenomena, e.g., refractive index, indicate that, especially for aryl groups, the C_6_ coefficient describing actual, physical London dispersion interactions is most certainly strongly anisotropic.^101^ Accordingly, there may well be specific interaction geometries for which DFT-D3 represents the interaction well, and others where it performs much less satisfactorily.

An uneven treatment of nonbonded interactions, with a strong dependence on the type of substituent and its orientation, presents a particular difficult challenge to methods for the calculation of complex organic and organometallic (flexible) molecules, as different conformations, or different valence isomers convertible over low barriers, whose relative energies are determined by a competition of multiple, noncovalent interactions of similar magnitude, would be treated differently, leading to a strong skewing of potential surfaces, depending, for example, on the rotation of an aryl group. In the worst case, the computational study could produce a wholly incorrect structure for the global minimum on the ground state surface.^102,103^ The case where an equilibrium structure of a molecule is determined by the balance of multiple, different, noncovalent interactions is common in the ground states and transition states of organic and organometallic reactions, especially where stereoselectivity is sought, which means that we have reservations as to the reliability of the DFT-D3 methods for these applications. In other words, given the magnitude of discrepancies observed in this, and other, studies, we believe that the utility of the DFT-D3 method may be seriously compromised for molecules of the size and complexity that one typically finds in, for example, asymmetric catalysis.

Having focused on DFT-D3 methods, we do note that there have been disturbing reports that even canonical CCSD(T) calculations, and some approximations thereof,^104^ may display systematic overbinding when applied to noncovalent complexes as they become larger.^105^ The recent study by Schäfer finds that the overbinding is likely caused by the truncation of the many-body perturbation series expansion. It appears that both computationally accessible wave function-based and density functional methods for noncovalent interactions in large molecules remain challenging.

In progress is an experimental study, which exceeds the scope of the present report, in which we will use the same T-CID methods to measure dissociation energies in large molecular ions—metal-free—for which the interaction geometry between aryl moieties is verifiably face-to-face. We expect the degree of agreement with DFT-D3 to confirm (or not) the hypothesis arising from the present data on the series of heterobimetallic complexes.

## Conclusion

We report a combined experimental-computational study of two series of heterobimetallic complexes with a d^8^–d^10^ bond between Pd(II) as the d^8^ component and either Cu(I), Ag(I), Au(I), or Zn(II) as the d^10^ component. Measurement of the bond dissociation energy by means of threshold collision-induced dissociation (T-CID), whose data are then deconvoluted by the program L-CID, produces the experimental *E*_0_ values for the gas phase reaction, which may be compared directly to bond dissociation energies computed with PBE-D3(BJ)/def2-CBS(3,4). We find a large, systematic discrepancy between experiment and theory for the Pd–Cu, Pd–Ag, and Pd–Au complexes, **1**^**+**^–**3**^**+**^, but a much smaller one for the Pd–Zn complexes, **4**^**+**^–**6**^**+**^ and the one Pd–Au complex, **8**^**+**^. We report a series of control experiments, reference calculations, and arguments with external, auxiliary data that indicate strongly that the experimentally determined *E*_0_ values are correct. A careful analysis of possible explanations identifies the most likely explanation for a large part of the discrepancy which shows up for one set of d^8^–d^10^ complexes, but not another. We find that a large part, if not most, of the discrepancy arises very probably from inadequacies in the description of the ligand–ligand noncovalent interactions. More specifically, while aryl–aryl, face-to-face interactions are treated well enough, alkyl-aryl and aryl–aryl, edge-to-face interactions appear to be overestimated seriously by the tested computational methods. With regard to the original motivation of a study of heterobimetallic d^8^–d^10^ complexes as models for the transmetalation step in Sonogashira and Negishi coupling reactions, the result means that model studies with DFT methods on simple models of the intermediates and transition states can probably give chemically realistic results, which is a positive result, but that the same DFT methods may fail with fully elaborated structures for which there are many nonbonded contacts between peripheral alkyl and aryl groups. Of particular concern would be a skewing of the conformational preferences, given the uneven treatment of interactions involving aryl groups, in particular. Nevertheless, by identifying the origin of various inconsistencies that have been reported in the literature, we hope that computational methodology can be upgraded to accommodate the findings.

## Supporting Information Available

General experimental details, synthetic procedures, MS and T-CID measurement details, NMR spectra; computational details, supplementary results, mathematical derivations, and discussion, generalized input files for all performed types of calculations (conformational search, optimization, single point calculation); R script for analyzing structural characteristics (PDF)
XMol xyz coordinates files for all computed structures; X-ray crystal data for all experimentally obtained crystal structures (ZIP)
