# Supplementary material for: Heterobimetallic Complexes That Point to When Bond Dissociation Energies Deviate from Computational Expectations
Source: J Am Chem Soc. 2025 Apr 15;147(17):14105–21. doi: 10.1021/jacs.4c14399 (PMC12046563; doi:10.1021/jacs.4c14399)
Supplement: Supplementary file 1 — ja4c14399_si_001.pdf [file ja4c14399_si_001.pdf]

Supporting Information

Heterobimetallic Complexes that point to when Bond Dissociation Energies deviate from Computational Expectations

Raphael Bissig, Raphael Oeschger, Peter Chen*

Laboratorium für Organische Chemie, ETH Zürich, Vladimir-Prelog-Weg 2, 8093 Zürich, Switzerland

*peter.chen@org.chem.ethz.ch

Contents

1. Numbered Structures ... 3
2. Experimental ... 5
  2.1. Synthesis – Heteroleptic charged Palladium Complex ... 6
    2.1.1. Synthesis of $bhq^{5-Br}H$, 7.1 ... 8
    2.1.2. Synthesis of $bhq^{5-CHO}H$, 7.2 ... 8
    2.1.3. Synthesis of $bhq^{5-CH2NMe2}H$, 7.3 ... 8
    2.1.4. Synthesis of $[bhq^{5-CH2NMe3}H^+]^-I$, $[7.4^+]^-I$ ... 9
    2.1.5. Synthesis of $[bhq^{5-CH2NMe3}H^+]^-BArF$, $[7.5^+]^-BArF$ ... 9
    2.1.6. Synthesis of $[Pd(bhq^{5-CH2NMe3})(OAc)]_n$ n BArF, $[7.6^{n+}]n^-BArF$ ... 9
    2.1.7. Synthesis of $[Pd(bhq^{5-CH2NMe3})(Cl)(SEt_2)]BArF$, $[7.7^+]^-BArF$ ... 10
    2.1.8. Synthesis of $[Pd(bhq)(bhq^{5-CH2NMe3})]BArF$, $[7^+]^-BArF$ ... 10
  2.2. Synthesis - Organozinc Compounds ... 11
    2.2.1. Synthesis of Bis(4-fluorophenyl)zinc, $Zn(Ar^{4F})_2$ ... 11
    2.2.2. Synthesis of Bis(2,6-difluorophenyl)zinc, $Zn(Ar^{2,6F})_2$ ... 11
    2.2.3. Synthesis of Bis(2,4,6-trifluorophenyl)zinc, $Zn(Ar^{2,4,6F})_2$ ... 12
  2.3. Synthesis - The Heterobimetallic Complexes ... 12
    2.3.1. Synthesis of $[(bhq)_2Pd^{II}Ag^{I}(IPr)]BArF$, $[2^+]^-BArF$: ... 12
    2.3.2. Synthesis of $[(bhq)_2Pd^{II}Au^{I}(IPr)]BArF$, $[3^+]^-BArF$: ... 13
    2.3.3. XRD-structures - Overview ... 14
  2.4. ESI-MS/MS - Gas-Phase Investigations ... 15
    2.4.1. ESI-MS/MS T-CID Measurements of $[(bhq)_2Pd\text{-}M(IPr)]^+$, $[2\text{-}3^+]$ ... 16
    2.4.2. ESI-MS/MS T-CID Measurements of $[(bhq)(bhq^{5-CH2NMe3})Pd\text{-}Zn(Ar)_2]^+$, $[4\text{-}6^+]$: ... 17
    2.4.3. ESI-MS/MS T-CID Measurements of $[(bhq)_2Pd\text{-}Au(PPh_3)]^+$ $[8^+]$: ... 18
    2.4.4. ESI-MS/MS T-CID Measurements of $[Pd(bhq)(bhq^{5-CH2-NMe3})]^+$, $[7^+]$: ... 18
    2.4.5. Summaries and Observations of the quantitative ESI-MS investigations ... 19
3. Computational ... 22
  3.1. Bond Dissociation Energies in Heterobimetallic complexes ... 22
    3.1.1. Procedures and Methods ... 22
    3.1.2. Atoms in Molecules Analysis ... 23
    3.1.3. Dissociation Characteristics – BDE ... 25

3.1.4. The Effect of the Charge 31
3.2. Investigations Into Possible Transformations prior Dissociation 32
4. Additional Experimental Spectra and Tables 38
4.1. NMR-Spectra of Products Towards Charged Pd-Complex Derivative 38
4.1.1. NMR Spectra of $bhq^{5\text{-}Br}H$,7.1 38
4.1.2. NMR Spectra of $bhq^{5\text{-}CHO}H$, 7.2 39
4.1.3. NMR Spectra of $bhq^{5\text{-}CH2NMe2}H$, 7.3 41
4.1.4. NMR Spectra of $[bhq^{5\text{-}CH2NMe3}H^+]^-I$, $[7.4^+]^-I$ 43
4.1.5. NMR Spectra of $[bhq^{5\text{-}CH2NMe3}H^+]^-BArF$, $[7.5^+]^-BArF$ 45
4.1.6. NMR Spectra of $[Pd(bhq^{5\text{-}CH2NMe3})(OAc)]_n$ n BArF, $[7.6^{n+}]n^-BArF$ 47
4.1.7. NMR Spectra of $[Pd(bhq^{5\text{-}CH2NMe3})(Cl)(SEt_2)]BArF$, $[7.7^+]^-BArF$ 50
4.1.8. NMR Spectra of $[Pd(bhq)(bhq^{5\text{-}CH2NMe3})]BArF$, $[7^+]^-BArF$ 52
4.2. NMR-Spectra of Organozinc Compounds 55
4.2.1. NMR Spectra of $Zn(Ar^{4F})_2$ 56
4.2.2. NMR Spectra of $Zn(Ar^{2,6F})_2$ 58
4.2.3. NMR Spectra of $Zn(Ar^{2,4,6F})_2$ 60
4.3. NMR-Spectra of Heterobimetallic Complexes 63
4.3.1. NMR Spectra of $[(bhq)_2Pd^{II}Ag^{I}(IPr)]BArF$, $[2^+]^-BArF$: 63
4.3.2. NMR Spectra of $[(bhq)_2Pd^{II}Au^{I}(IPr)]BArF$, $[3^+]^-BArF$: 66
4.4. Processing ESI-MS/MS - T-CID / L-CID 69
4.4.1. T-CID / L-CID data for the Measurements of $[(bhq)_2Pd\text{-}Ag(IPr)]^+$, $[2^+]$ 71
4.4.2. T-CID / L-CID data for the Measurements of $[(bhq)_2Pd\text{-}Au(IPr)]^+$, $[3^+]$ 72
4.4.3. T-CID / L-CID data for the Measurements of $[(bhq)(bhq^{5\text{-}CH2NMe3})Pd\text{-}Zn(C_6F_5)_2]^+$, $[4^+]$ 73
4.4.4. T-CID / L-CID data for the Measurements of $[(bhq)(bhq^{5\text{-}CH2NMe3})Pd\text{-}Zn(Ar^{2,4,6F})_2]^+$, $[5^+]$ 74
4.4.5. T-CID / L-CID data for the Measurements of $[(bhq)(bhq^{5\text{-}CH2NMe3})Pd\text{-}Zn(Ar^{2,6F})_2]^+$, $[6^+]$ 75
4.4.6. T-CID / L-CID data for the Measurements of $[(bhq)_2Pd\text{-}Au(PPh_3)]^+$ $[8^+]$ 77
4.4.7. T-CID / L-CID data for the Measurements of $[Pd(bhq)(bhq^{5\text{-}CH2\text{-}NMe3+})]$, $[7^+]$ 78
4.5. Computational Input files and Scripts 79
4.5.1. CREST Calculations 79
4.5.2. ORCA Input Templates 79
4.5.3. ADF Input Templates 81
4.5.4. Single point Energies 85
4.5.5. AIM-Analysis 87
4.5.6. AIM-IGMH-Analysis 88
4.5.7. DFT-D3 Analysis 98
4.5.8. Scripts 98
5. References 100

1. Numbered Structures

Structure	Chemical Name	Abbreviation	Numbering
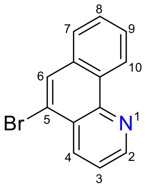	5-Bromobenzo[*h*]quinoline,	$bhq^{5\text{-}Br}H$	**7.1**
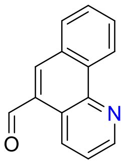	Benzo[*h*]quinoline-5-carbaldehyde	$bhq^{5\text{-}CHO}H$	**7.2**
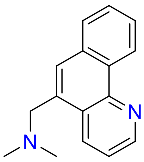	1-(Benzo[*h*]quinolin-5-yl)-N,N-dimethylmethanamine	$bhq^{5\text{-}CH_2NMe_2}H$	**7.3**
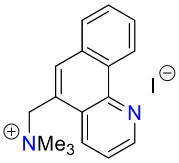	1-(Benzo[h]quinolin-5-yl)-N,N,N-trimethylmethanaminiumiodide	$[bhq^{5\text{-}CH_2NMe_3}H^+]^-I$	$[\mathbf{7.4}^+]^-I$
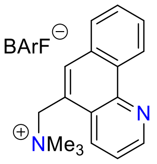	1-(Benzo[h]quinolin-5-yl)-N,N,N-trimethylmethanaminiumtetrakis(3,5-bis(trifluoromethyl)phenyl)borate	$[bhq^{5\text{-}CH_2NMe_3}H^+]^-$BArF	$[\mathbf{7.5}^+]^-$BArF
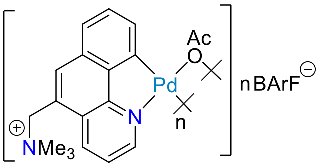	NA	$[Pd(bhq^{5\text{-}CH_2NMe_3})(OAc)]_n$ n BArF	$[\mathbf{7.6}^{n+}]n^-$BArF
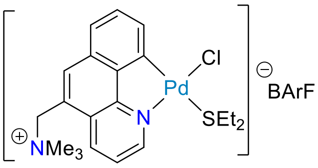	NA	$[Pd(bhq^{5\text{-}CH_2NMe_3})(Cl)(SEt_2)]$BArF	$[\mathbf{7.7}^+]^-$BArF
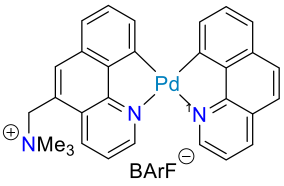	NA	$[Pd(bhq)(bhq^{5\text{-}CH_2NMe_3})]$BArF	$[\mathbf{7}^+]^-$BArF
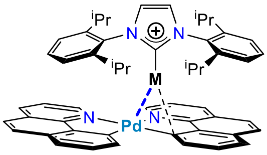	NA	$[(bhq)_2Pd^{II}\text{-}M^{I}(IPr)]$BArF	M^I=<br>Cu →$[\mathbf{1}^+]$<br>Ag →$[\mathbf{2}^+]$<br>Au →$[\mathbf{3}^+]$
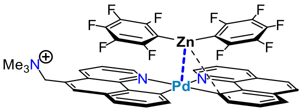	NA	$[(bhq)(bhq^{5\text{-}CH_2NMe_3})Pd\text{-}Zn(C_6F_5)_2]^+$	$[\mathbf{4}^+]$

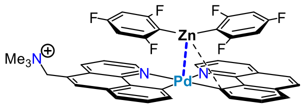	NA	[(bhq)(bhq$^{5\text{-}CH_2NMe_3}$)Pd-Zn(Ar^{2,4,6F})$_2$]$^+$	[**5**$^+$]
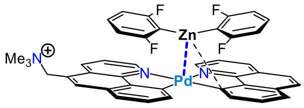	NA	[(bhq)(bhq$^{5\text{-}CH_2NMe_3}$)Pd-Zn(Ar^{2,6F})$_2$]$^+$	[**6**$^+$]
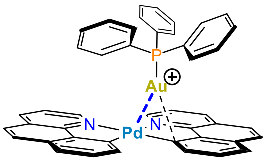	NA	[(bhq)$_2$Pd^{II}-Au^{I}(PPh3)]	[**8**$^+$]

2. Experimental

General Information Synthesis

General: Unless otherwise stated, all reactions were carried out under an atmosphere of argon using standard Schlenk techniques or in a nitrogen filled glovebox. Reactions where Ag(I) was present were conducted with minimal sources of light present.

Dry Solvents: The following solvents were dried and distilled prior to use : dichloromethane (DCM) / acetonitrile (MeCN) / 1,2-difluorobenzene (DFB) over CaH_2; *n*-hexane, *n*-pentane, diethylether (Et_2O), tetrahydrofuran (THF) over Na/benzophenone with tetraethyleneglycoldimethylether (TEGDME); toluene over Na; MeOH over Mg. The solvents were stored over molecular sieves in a N_2-filled glovebox. Commercially available ethyl acetate (EtOAc, Acros Organics with Acroseal), dimethylformamide (DMF) and methanol (MeOH) were stored over molecular sieves prior to use. Deuterated solvents were dried by storing over molecular sieves for at least 3 days prior to use.

Commercial consumables: Syringe filters (PTFE, pore size 0.2 µm, diameter. 13 mm, Whatman or PTFE, pore size 0.45 µm, diameter 25 mm, CHROMAFIL) were dried using a vacuum oven at 60 °C for at least 3 days prior to use.

Commercially available compounds: The following chemicals were purchased and used without further purifaction: (*organics*) benzo[*h*]quinoline (bhqH, TCI), *N*-bromosuccinimide (NBS, Aldrich), methyliodide (MeI, Sigma Aldrich), 4-fluoroiodobenzene (Sigma Aldrich), 1-bromo-2,6-difluorobenzene (TCI), 1-bromo-2,4,6-trifluorobenzene (TCI), SEt_2 (TCI), (*organometallics*) isopropylmagnesium chloride - lithium chloride complex solution ((*i*Pr)MgCl•LiCl, Acros), chloro[1,3-bis(2,6-diisopropylphenyl)imidazol-2-ylidene]copper(I) ([(IPr)CuCl], TCI), chloro[1,3-bis(2,6-diisopropylphenyl)imidazol-2-ylidene]silver(I) ([(IPr)AgCl], TCI), chloro[1,3-bis(2,6-diisopropylphenyl)imidazol-2-ylidene]gold(I) ([(IPr)AuCl], ABCR), bis(pentafluorphenyl)zinc ($Zn(C_6F_5)_2$, Sigma Aldrich), palladium(II) acetate ($Pd(OAc)_2$, Fluorochem), zinc(II) acetate dihydrate ($Zn(OAc)_2$•$2H_2O$, Fluka), zinc(II) chloride ($ZnCl_2$, Acros), (*salts*) sodium tetrakis[3,5-bis(trifluoromethyl)phenyl]borate (NaBArF, Apollo), silver tetrafluoroborate ($AgBF_4$, ABCR), silver trifluoromethanesulfonate (AgOTf, ABCR). *n*-Butyllithium in *n*-hexane solution (*n*-BuLi, 1.6 M in *n*-hexane, Sigma Aldrich) was titrated with *sec*-Butanol using 2,2'-bipyridine as an indicator prior to use.

Reported compounds: The following compounds were synthesized according to literature: *cis*-Bis(benzo[*h*]quinoline)palladium(II) ($Pd(bhq)_2$)[1], (acetonitrile)[1,3-bis(2,6-diisopropylphenyl)imidazol-2-ylidene]gold(I) tetrakis[3,5-bis(trifluoromethyl)phenyl]borate ([(IPr)Au(MeCN)]BArF)[2], 5-bromo-benzo[*h*]quinoline ($bhq^{5\text{-}Br}H$)[3].

Analytical Instrumentation:

NMR spectra were recorded on a Bruker Ascend 400 MHz and were analyzed using the MestReNova (v14.1.0-24037) software suite. The spectra were referenced to the specific residual solvent peak[4]: In ^{1}H-NMR spectra the peak integrals were normalized to a well separated and identifiable peak or so that the sum of compound peak integrals added up to the expected number of H nuclei.

XRD measurements were performed by the in-house service. Temperature sensitive compounds were transported in a pre-cooled (-35°C) dewar and immediately transferred onto a µChill coldhead[5] device using a pipette. The crystals were kept at low temperatures with a constant flow of cold inert gas during the preparation of the XRD measurement. Single crystals of the compound were selected and mounted on a XtaLAB Synergy, Dualflex, Pilatus 300K diffractometer (or on a Bruker Apex2 Duo (Mo) diffractometer). The crystals were kept at 100.0(1) K or 200(1) K during data collection. Using Olex2[6], the structure was solved with either the SHELXT[7](or the XT[7]) structure solution program using Intrinsic Phasing and refined with the SHELXL[8] (or the XL[9]) refinement package using Least Squares minimization. The obtained data were analyzed using the Mercury software suite (Version 3.10.3).

Elemental Analysis (EA), the quantitative measurement of the elemental composition of a compound with respect to specific elements was performed by the in-house service. For C, H, N, a LECO TruSpec Micro instrument was used, wherein the gaseous combustion products of C (CO2) and H (H2O) are quantified by means of infrared spectroscopy. Nitrogen is measured as N_2 with a thermal conductivity detector. Elemental compositions of the halides Cl and F were determined by digesting the sample using the Schöniger method, followed by capturing combustion products in an absorbing

solution with subsequent quantification by ion chromatography. Compounds were dried a few days before performing Elemental analysis.

Electrospray Ionization (ESI) high resolution mass spectrometry (HR-MS) measurements for analytical purposes were performed by the in-house service on a Bruker Daltonics maXis ESI-QTOF Instrument.

Qualitative Electrospray Ionization Mass Spectrometry (ESI-MS/MS) experiments were performed on a Thermo Finnigan TSQ Quantum triple quadrupole mass spectrometer. Gas-tight syringes were used for spray solution injection.

Quantitative Electrospray Ionization Mass Spectroscopy – Threshold Collision Induced Dissociation (ESI-MS / T-CID) experiments were performed on a modified TSQ Quantum Ultra mass spectrometer. For all experiments thermalization gas was Ar and the pressure was set to approx. 30 mTorr. Other key settings used for the different measurements are mentioned in the corresponding experimental procedures.

Programs:

Isotopic pattern simulations were performed using the in-house program IsoPat Version 0.2 for Windows, written by Dr. E.P.A Couzijn 2011

T-CID / L-CID: The T-CID data were processed using the in-house phyton program workup_data.py. The L-CID fits were performed using the lcid-0.9.3 version, recompiled 2023. The L-CID data were then processed using the in-house python programs workup_fits.py and workup_2fits.py.

2.1. Synthesis – Heteroleptic charged Palladium Complex

Introduction

Scheme SI-2-1 provides a schematic overview of the retrosynthetic strategy employed for the synthesis of the here discussed heterobimetallic complexes $[(bhq)_2Pd^{II}M(L_n)]^{z+}$. Intra-molecular interactions, between the d^8 and d^{10} fragments, were studied by investigating dissociation characteristics, e.g. Bond dissociation energies (BDE) in gas-phase, both experimentally and computationally. The bond dissociation energies (BDEs) were experimentally determined using electrospray ionization mass spectrometry (ESI-MS). This involved measuring gas-phase rate data through threshold collision-induced dissociation (T-CID) experiments. The resulting data were then deconvoluted using the ligand collision-induced dissociation (L-CID) method.[10] This method is limited to measurements of charged species,[11] e.g. the previously reported study of $[(bhq)_2Pd\text{-}Zn(C_6F_5)_2]$ was restricted to the homogeneous solution phase.[12] To overcome this limitation a prosthetic charged substituent was introduced.

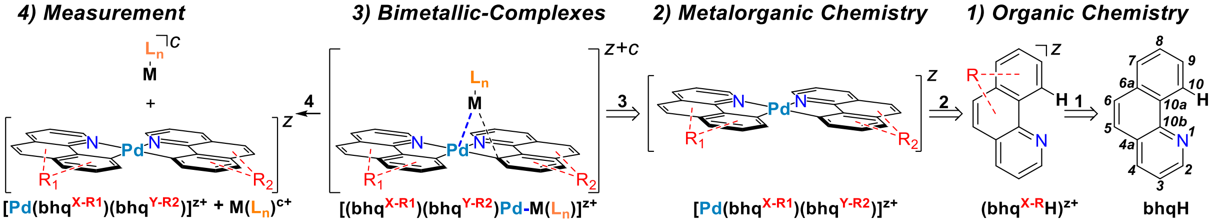

Scheme SI-2-1: Schematic representation of the derivatization strategy in a retrosynthetic notation (*1-3*). The dissociation process is also shown to the left *4*.

The choice of the substituent and its location in the molecule was mainly influenced by considerations regarding factors such as its structural and electronic effects on the overall interaction and its synthetic feasibility. In principle the substituent can be introduced on either fragment, here the substituent is introduced on the d^{10} fragment.

The synthesis of bis-cyclometalated Pd(II) complexes involves distinct synthetic methodologies, for bis-homoleptic $[Pd(bhq^R)_2]$ and bis-heteroleptic $[Pd(bhq^{R1})(bhq^{R2})]$ complexes respectively.[13] For bis-homoleptic complexes, a single preactivated precursors species, commonly the brominated ligand activated by lithiation, is combined with *trans*-$[Pd(SEt_2)_2Cl_2]$ yielding the *cis*-configuration complex.[13] In the more arduous synthesis of heteroleptic complexes, the ligands are introduced sequentially as the intermediate complex $[LPd(SEt_2)Cl]$ is first isolated.[13] Reportedly these monocyclometalated complexes can be prepared via mild C-H activation of the ligand precursor with Pd complex precursors. For example, by mixing the ligand precursor directly with $Pd(OAc)_2$ to form a monocyclometalated palladium-acetate dimer,[14,15] which is usually converted into a chlorine-bridged dimer and cleaved by the addition of SEt_2, leading to the formation of the monocyclometalated complex $[LPd(SEt_2)Cl]$.[1,16] Although various benzo[*h*]quinoline derivatives (ligand-precursors) can be synthesized,[14,15,17–23] only the

formations of [Pd(bhq$^{7\text{-Me}}$)$_2$] and [Pd(bhq$^{7\text{-OMe}}$)$_2$], with electron-donating substituents, were achieved prior this work.[24]

Based on synthetic feasibility, experimental experience, and computational assessments, a heteroleptic complex with a -$CH_2NMe_3^+$ substituent at location 5 (see Scheme SI-2-1) is proposed. The CH_2 linker is expected to moderate the electron-withdrawing effect of NMe_3^+ and also provide greater configurational restraint compared to longer tethers. A single positive charge is preferred to model gas-phase kinetics and reduce the risk of corona discharge. The objective was to place this substituent in a remote region of the system to avoid interference with the molecule's core and its interactions. While both positions 5 and 6 are classified as remote, their suitability for the intended application remains uncertain, however position 5 provides greater synthetical accessibility. A drawback of the system is the potential homolytic cleavage of the C-N bond in -CH_2-NMe_3^+, yielding a benzylic cation and dissociated NMe_3. Figure SI-2-1 shows the synthetic route to complex [Pd(bhq)(bhq$^{5\text{-}CH_2NMe_3}$)$^+$]BArF, [**7**$^+$]$^-$BArF, full details and characterizations of [**7**$^+$]$^-$BArF, with BArF as the counterion are provided in the subsection 2.1.8.

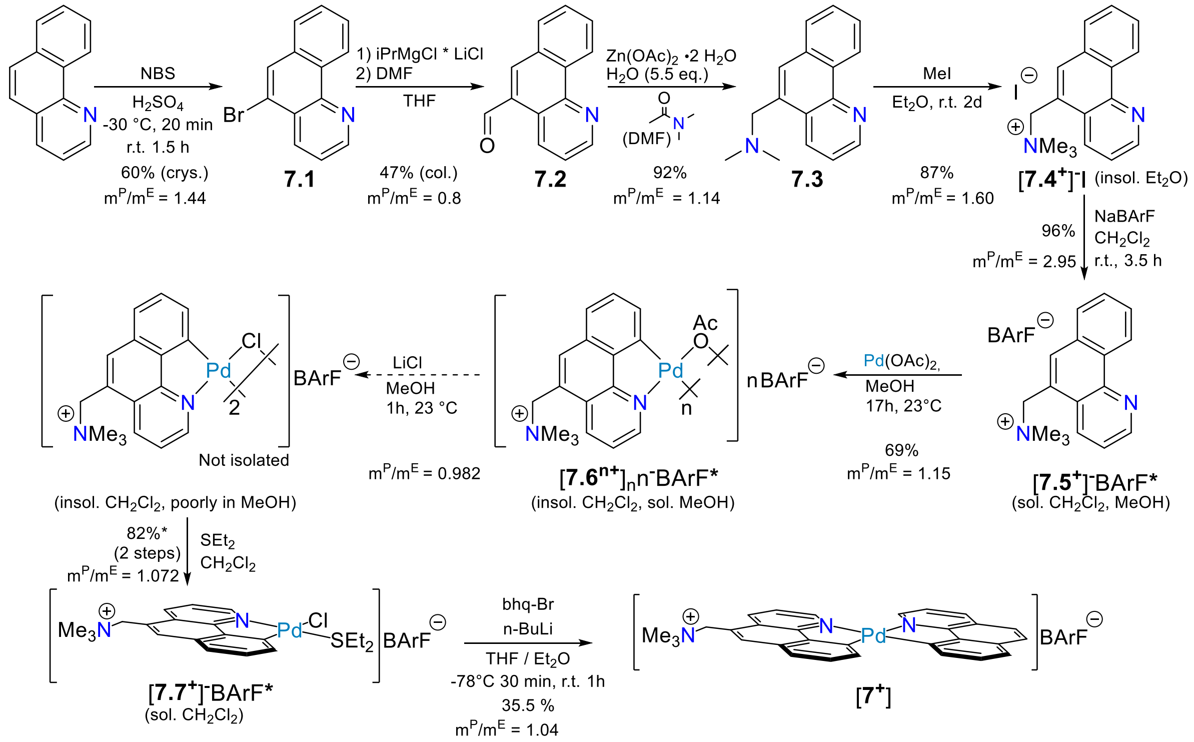

Figure SI-2-1: Synthesis of complex cis-[Pd(bhq)(bhq$^{5\text{-}CH_2NMe_3}$)$^+$]$^-$BArF [**7**$^+$]BArF, additional information are listed: yield in [%] together with purification type, factor for gain in molecular weight, in some cases solubility properties.

Despite the potential electron withdrawing characteristics of the charged substituent, the C-H activation **7.5**$^+$ to **7.6**$^{n+}$ worked. Regarding the monocyclometalated palladium acetate species **7.6**$^{n+}$, it is noteworthy that, for the non-charged analog, a dimer with stabilizing π-π interactions was observed by x-ray crystallography.[14] A further crucial step is the last step, where the second ligand is introduced via a lithiation. Despite the harsh conditions, a mediocre yield was obtained. The obtained complex [**7**$^+$]$^-$BArF, was characterized by NMR spectroscopy and ESI-MS/MS investigations. Both the isotope pattern as well as integral assessment in the ^{1}H-NMR spectra, suggested the right composition, also a single BArF counterion was indicated from NMR evaluation. In addition, ^{1}H-^{1}H-NOESY NMR spectroscopy was used to assert the configuration. Two cross-peaks, for two sets of hydrogens (hydrogens on position 2 and 9 on either side and in close proximity), at two different shift ranges, are indicative for a cis-configuration (see publication).

2.1.1. Synthesis of bhq$^{5\text{-}Br}$H, 7.1

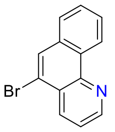

This synthesis was adapted from literature:[3,23] Benzo[*h*]quinoline (15 g, 83.7 mmol, 1 eq, TCI) was dissolved in conc. H_2SO_4 (150 mL) at 0°C. This mixture was cooled down to -30 °C (using a MeCN /dry ice bath), due to the increased viscosity the mixture was stirred using an overhead stirrer. To the reaction mixture *N*-bromosuccinimide (15 g, 84.6mmol, 1 eq., Aldrich) was added portion-wise. The mixture was stirred for 20 min and then let warm up and stirred for another 1.5h at r.t.. The mixture was poured on ice and basified to pH 14 aqueous solution of NaOH. The precipitate was filtered off and the water-phase was extracted with Et_2O (approx. 0.5 L). The precipitate was suspended in Et_2O. The combined organic phases were washed with H_2O and dried using $MgSO_4$. The obtained organic phase was reduced in volume by rotary evaporation until saturation was reached, and subsequently put into the freezer and kept at -20°C. Pale-pink crystals were obtained, the crystallization process was continued, and two more crops were obtained. The residual pale-pink solid was recrystallized from Et_2O one more time where again multiple crops were obtained. After drying using high vacuum a pale-pink solid was obtained. (13 g, 50.2 mmol, Yield: 60 %)

^{1}H NMR ($CDCl_3$, 400 MHz, ref res-sol. peak 7.26 ppm): δ (ppm) = 9.31 – 9.22 (1H, m), 9.01 (1H, dd, *J*=4.4, 1.7 Hz), 8.61 (1H, dd, *J*=8.3, 1.7 Hz), 8.15 (1H, s), 7.83 (1H, dd, *J*=7.7, 1.6 Hz), 7.81 – 7.64 (2H, m), 7.62 (1H, dd, *J*=8.3, 4.4 Hz).

(for NMR spectra see 4.1.1)

2.1.2. Synthesis of bhq$^{5\text{-}CHO}$H, 7.2

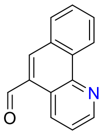

In a Schlenk tube under Argon, 5-bromobenzo[*h*]quinoline (6 g, 23.25 mmol, 1 eq., **7.1**) was dissolved in THF (44 mL, dry). To this solution Isopropylmagnesium chloride - lithium chloride complex solution ((*i*Pr)MgCl•LiCl, 1.3 M, 36 mL, 46.8 mmol, 2 eq., Acros) was added at r.t., the color changes from slightly pink, via yellow, to dark-green. After the reaction mixture was stirred at r.t. for 1h, it was transferred to another Schlenk flask containing DMF (24 mL, extra dry) portionwise over 30 min using cannula transfer. A reflux condenser was mounted, and the reaction mixture was stirred at 60°C for 90 min. The reaction was quenched using aqueous HCl (2M, 45 mL), then aqueous NaOH (100 mL, 1M) was added until the mixture was basic, approx. pH > 11. The aqueous phase was extracted with CH_2Cl_2 multiple times (approx. 4 x 250 mL). The obtained organic phase was washed with water, brine and dried with $MgSO_4$, then filtered. Subsequently, the volatiles were removed using rotary evaporation. The residual orange solid was separated using column chromatography (Rf= approx. 0.08). Column chromatography was conducted using a gradient eluent (eluent 1:1 (*v/v*) *n*-hexane: CH_2Cl_2 to pure CH_2Cl_2) and yielded a yellow solid. (1844.27mg, 8.9 mmol, Yield: 38.3 %)

^{1}H NMR ($CDCl_3$, 400 MHz, ref res-sol. peak 7.26 ppm): δ (ppm) = 10.36 (1H, s), 9.68 (1H, dd, *J*=8.4, 1.8 Hz), 9.33 (1H, ddt, *J*=8.3, 1.3, 0.7 Hz), 9.06 (1H, dd, *J*=4.3, 1.8 Hz), 8.38 – 8.33 (1H, m), 8.07 (1H, ddt, *J*=7.9, 1.3, 0.6 Hz), 7.91 (1H, ddd, *J*=8.4, 7.1, 1.3 Hz), 7.79 (1H, ddd, *J*=7.9, 7.1, 1.3 Hz), 7.64 (1H, dd, *J*=8.4, 4.3 Hz)

^{13}C{^{1}H} NMR ($CDCl_3$, 101 MHz, ref res-sol. peak 77.16 ppm): δ (ppm) = 193.45, 149.87, 146.72, 141.76, 134.18, 134.14, 131.71, 130.86, 129.98, 129.64, 129.10, 125.21, 123.35, 123.25

Elemental Analysis: Calculated for $C_{14}H_8NO$: C: 81.14%, H: 4.38%, N: 6.76%, Found C:79.67 %, H:4.33 %, N:6.66 %

(for NMR spectra see 4.1.2)

2.1.3. Synthesis of bhq$^{5\text{-}CH_2NMe_2}$H, 7.3

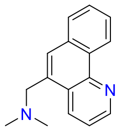

This synthesis was adapted from literature:[25] In a vial, bhq$^{5\text{-}CHO}$H (1.4 g, 6.76 mmol, 1 eq., **7.2**) was combined with $Zn(OAc)_2$•$2H_2O$ (150.48 mg, 0.69 mmol, 0.1 eq, Fluka), DMF (17 mL, 32 eq.) and H_2O (deion. 0.67 mL (5.5 eq.). The reaction mixture was heated to 150 °C and stirred overnight. Then deion. H_2O and Brine were added to the reaction mixture and this aqueous phase was extracted with Et_2O (3 x 150 mL). The org. phase was washed with brine and dried with Mg_2SO_4. The volatiles were removed using rotary evaporation. Residual Oil dried up and yielded an orange solid. (1.47 mg, 6.22 mmol, 92 % Yield). This product was used without further purification.

^{1}H NMR ($CDCl_3$, 400 MHz, ref res-sol. peak 7.26 ppm): δ (ppm) = 9.30 – 9.24 (1H, m), 9.00 (1H, dd, *J*=4.3, 1.8 Hz), 8.68 (1H, dd, *J*=8.3, 1.8 Hz), 7.87 (1H, ddd, *J*=7.2, 1.7, 0.9 Hz), 7.74 – 7.69 (1H, m), 7.70 (1H, s), 7.71 – 7.66 (1H, m), 7.55 (1H, dd, *J*=8.3, 4.3 Hz), 3.85 (2H, d, *J*=0.8 Hz), 2.32 (6H, s) ppm

^{13}C{^{1}H} NMR ($CDCl_3$, 101 MHz, ref res-sol. peak 77.16 ppm): δ (ppm) = 148.66, 147.08, 133.44, 133.09, 132.52, 131.66, 128.42, 128.36, 127.76, 127.07, 126.41, 124.58, 121.77, 63.00, 45.71

Elemental Analysis: Calculated for $C_{16}H_{16}N_2$: C: 81.32 %, H: 6.82 %, N: 11.85 %, Found C: 79.78 %, H: 6.68 %, N: 11.42 %

(for NMR spectra see 4.1.3)

2.1.4. Synthesis of [bhq$^{5\text{-}CH_2NMe_3}$H$^+$]$^-$I, [7.4$^+$]$^-$I

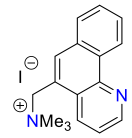

In a round bottomed flask bhq$^{5\text{-}CH_2NMe_2}$ (1 g, 4.23 mmol, 1 eq., **7.3**) was dissolved in Et_2O (16 mL, dry), under an inert gas atmosphere. To this solution MeI (2.1 mL, 33.73 mmol, 8 eq., Sigma Aldrich) was added, and stirred at r.t. over the weekend. The white-beige precipitations were collected by filtration and washed with Et_2O (2 x 20 mL). (1.398 mg, 3.7 mmol, 87.3 %). This product was used without further purification.

^{1}H NMR (MeOD-d^4, 400 MHz, ref res-sol. peak 3.31 ppm): δ (ppm) = 9.34 – 9.27 (1H, m), 9.07 (1H, dd, *J*=4.3, 1.6 Hz), 8.86 (1H, dd, *J*=8.5, 1.6 Hz), 8.28 (1H, s), 8.12 (1H, dd, *J*=7.5, 1.6 Hz), 7.92 – 7.81 (2H, m), 7.79 (1H, dd, *J*=8.5, 4.3 Hz), 5.16 (2H, s), 3.23 (9H, s)

^{13}C{^{1}H} NMR (MeOD-d^4, 101 MHz, ref res-sol. peak 53.84 ppm): δ (ppm) = 150.59, 137.58, 133.69, 130.26, 129.90, 125.53, 123.76, 66.10, 53.80

ESI-HRMS Positive mode: calculated for $C_{17}H_{19}N_2^+$ [M$^+$] 251.1543 m/z; found 251.1547 m/z. Negative mode: calculated for I$^-$ [M$^-$] 126.9050 m/z; found 126.9047 m/z

(for NMR spectra see 4.1.4)

2.1.5. Synthesis of [bhq$^{5\text{-}CH_2NMe_3}$H$^+$]$^-$BArF, [7.5$^+$]$^-$BArF

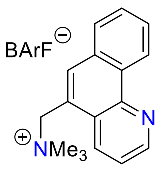

In a round bottom flask [bhq$^{5\text{-}CH_2NMe_3}$]I (1 g, 2.64 mmol, 1 eq., [**7.4**$^+$]$^-$I) was combined with NaBArF (2.33 g, 2.63 mmol, 1 eq., Apollo). The mixture was suspended in CH_2Cl_2 (500 mL). The suspension was stirred for approx. 3 h at r.t.. The obtained reaction mixture was filtered, and the volatiles were removed using rotary evaporation. A yellowish solid was obtained and used without further purification (2.84 g, 2.55 mmol, 96 %).

^{1}H NMR (CD_2Cl_2, 400 MHz, ref res-sol. peak 5.32 ppm): δ (ppm) = 9.39 (1H, ddt, *J*=8.3, 1.4, 0.7 Hz), 9.15 (1H, dd, *J*=4.3, 1.6 Hz), 8.32 (1H, dd, *J*=8.4, 1.6 Hz), 8.03 (1H, s), 8.03 (1H, ddd, *J*=7.9, 1.3, 0.6 Hz), 7.94 (1H, ddd, *J*=8.4, 7.1, 1.4 Hz), 7.86 (1H, ddd, *J*=7.9, 7.1, 1.3 Hz), 7.75 – 7.71 (8H, m), 7.72 (1H, dd, *J*=8.4, 4.3 Hz), 7.58 – 7.55 (4H, m), 4.92 (2H, s), 3.18 (9H, s)

^{13}C{^{1}H} NMR (CD_2Cl_2, 101 MHz, ref res-sol. peak 53.84 ppm): δ (ppm) = 162.92, 162.42, 161.93, 161.43, 150.66, 136.96, 135.22, 133.56, 131.72, 130.68, 130.63, 130.10, 129.43, 129.17, 129.08, 128.97, 126.38, 125.34, 123.67, 123.17, 119.45, 117.91, 117.87, 67.65, 54.78, 54.74, 54.7

^{19}F{^{1}H} NMR (CD_2Cl_2 , 377 MHz): δ = -62.80

^{11}B NMR (CD_2Cl_2, 128 MHz): δ = -6.61 (d, *J*=4.6 Hz)

Elemental Analysis: Calculated for $C_{49}H_{31}BF_{24}N_2$: C: 52.80 %, H: 2.80 %, N: 2.51 %,Found C: 51.36 %, H: 2.94 %, N: 2.61 %

ESI-HRMS Positive mode: calculated for $C_{17}H_{19}N_2^+$ [M+] 251.1543 m/z; found 251.1539 m/z. Negative mode: calculated for $C_{32}H_{12}BF_{24}^-$ [M]$^-$ 863.0654 m/z; found 863.0632 m/z

(for NMR spectra see 4.1.5)

2.1.6. Synthesis of [Pd(bhq$^{5\text{-}CH_2NMe_3}$)(OAc)]$_n$ n BArF, [7.6^{n+}]n$^-$BArF

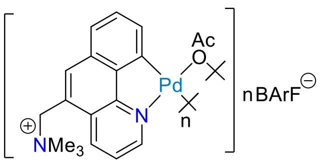

$Pd(OAc)_2$ (302 mg, 1.34 mmol, 1 eq, Fluorochem) and [bhq$^{5\text{-}CH_2NMe_3}$]BArF (1.5 g, 1.35 mmol, 1 eq, [**7.5**$^+$]$^-$BArF) were combined and suspended in MeOH (95 mL, extra dry). This mixture was stirred at r.t. for 23 h. The obtained greenish-yellow suspension was filtered over a pad of celite, which was washed with MeOH (80 mL). The volatiles were removed using rotary evaporation. The obtained residual was suspended with CH_2Cl_2 and the precipitation was filtered off. The volatiles were removed using rotary evaporation. The obtained solid was resuspended in CH_2Cl_2 (80 mL) and filtered using a glass frit (Por 4) and washed with CH_2Cl_2 (40 mL), a light yellow solid was obtained (1.19 g , 0.93 mmol, approx. 69 % assuming purity). This product was used without further purification.

^{1}H NMR (MeOD-d^4, 400 MHz, ref res-sol. peak 3.31 ppm): δ (ppm) = 8.54 – 8.08 (Int:2, m broad), 7.84 – 7.55 (Int:14, m broad), 7.43 – 7.03 (Int:3, m broad), 3.27 – 3.15 (Int:1, m broad), 3.14 – 2.94 (Int:8, m broad), 2.33 (Int:3, s)

^{13}C{^{1}H} NMR (MeOD-d^4, 101 MHz, ref res-sol. peak 49.00 ppm): δ (ppm) = 135.81, 127.13, 124.43, 118.48, 53.60

^{19}F{^{1}H} NMR (MeOD-d^4 , 377 MHz): δ (ppm) = -64.30

^{11}B NMR (MeOD-d^4, 128 MHz): δ (ppm) = -6.76

(for NMR spectra see 4.1.6)

2.1.7. Synthesis of [Pd(bhq$^{5\text{-}CH_2NMe_3}$)(Cl)(SEt$_2$)]BArF, [7.7$^+$]$^-$BArF

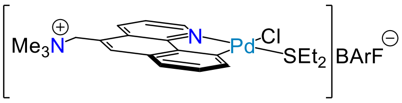

The synthesis was performed in an ambient air atmosphere, but dry solvents were used. [Pd(bhq$^{5\text{-}CH_2NMe_3}$)(OAc)]$_n$nBArF (1.1 g, 0.86 mmol, 1 eq., [**7.6**$^{n+}$]nBArF$^-$) was dissolved in MeOH (20 mL, extra dry). Then LiCl (729 mg, 17.2 mmol, 20 eq.) was dissolved in MeOH (20 mL, extra dry) and added to the Pd-complex. Decolorization and precipitation of a white solid was observed. The volatiles were removed using rotary evaporation, a grey slurry-solid was obtained and was used without further purification. This solid mixture is expected to contain [Pd(bhq$^{5\text{-}CH_2NMe_3}$)(Cl)]$_2$ 2BArF. The grey slurry mixture was suspended in CH_2Cl_2 (60 mL) and sonicated. To this suspension SEt$_2$ (0.8 mL, 7.14 mmol, 8.3 eq., TCI) was added. The reaction mixture was stirred at r.t. for 40 min and subsequently filtered through a glass filter frit (Por 4). The volatiles were removed from the clear yellow solution using rotary evaporation. The obtained solid was further dried using high vacuum. The solid was dissolved in CH_2Cl_2 and filtered using a syringe filter (PTFE, pore size 0.45 µm, diameter 25 mm, CHROMAFIL). Volatiles were removed using rotary evaporation with subsequent high vacuum and a yellow solid was obtained (818 mg, 0.61 mmol, 71 %).

^{1}H NMR (CD_2Cl_2, 400 MHz, ref res-sol. peak 5.32 ppm): δ (ppm) = 10.13 – 9.39 (Int:1, s broad), 8.40 (1H, d, *J*=8.3 Hz), 8.02 (1H, s), 7.99 – 7.89 (Int:1, s broad), 7.84 – 7.77 (Int:1, m broad), 7.76 – 7.69 (Int:9.5, m broad), 7.66 (t, 1H, *J*=7.7 Hz), 7.54 (d, 4H, *J*=2.2 Hz), 4.87 (2H, s), 3.53 – 2.67 (Int:12, m broad), 1.63 – 1.32 (Int:6, s broad)

^{13}C{^{1}H} NMR (CD_2Cl_2, 101 MHz, ref res-sol. peak 53.84 ppm): δ (ppm) = 162.90, 161.90, 161.41, 135.21, 134.45, 129.42, 129.12, 129.06, 126.36, 123.65, 120.94, 117.90, 117.86, 13.96

^{19}F{^{1}H} NMR (CD_2Cl_2 , 377 MHz): δ = -62.76

^{11}B NMR (CD_2Cl_2, 128 MHz): δ = -4.10 – -9.80 (m)

(for NMR spectra see 4.1.7)

2.1.8. Synthesis of [Pd(bhq)(bhq$^{5\text{-}CH_2NMe_3}$)]BArF, [7$^+$]$^-$BArF

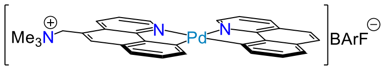

In a Schlenk tube under Argon, bhq-Br (57.6 mg, 0.22 mmol, 1 eq., **7.1**) was dissolved in THF (5 mL, dry). To this solution, *n*-BuLi (0.14 mL, 0.22 mmol, 1 eq., 1.6 M in n-hexane, Aldrich) was added slowly at -78°C and stirred for 1h while keeping cold. Then the reaction mixture was transferred portionwise to a solution of [Pd(bhq$^{5\text{-}CH_2NMe_3}$)(Cl)(SEt$_2$)]BArF (300.4 mg, 0.22 mmol, 1 eq., **7-8**) in THF (5 mL, dry), via cannula transfer. The reaction mixture was let warm to r.t. and kept stirring for 2.5 h, subsequently the mixture was transferred to a round bottom flask and THF (60 mL, not dry) was added. Volatiles were removed using rotary evaporation to obtain an orange solid. The solid was suspended in $CHCl_3$ (50 mL) and filtered over a glass filter frit (por 4) and washed with $CHCl_3$ (40 mL). The solid was dried using vacuum. This solid (200 mg) was suspended in MeOH (4 mL) and filtered using a syringe filter. $CHCl_3$ (8 mL) was added carefully, and the mixture was kept in the freezer (-20 °C) for a few days. The observed crystals were isolated, and the product was obtained as an orange solid. Multiple crops were taken (92.21 mg, 0.066 mmol, 35.5 %).

^{1}H NMR (CD_2Cl_2, 400 MHz, ref res-sol. peak 5.32 ppm): δ (ppm) = 9.25 (dd, J = 5.0, 1.3 Hz, 1H), 9.04 (dd, J = 5.1, 1.5 Hz, 1H), 8.63 (dt, J = 7.0, 1.1 Hz, 1H), 8.41 (dd, J = 7.9, 1.4 Hz, 1H), 8.39 (dd, J = 8.3, 1.2 Hz, 1H), 8.34 (dd, J = 6.6, 1.6 Hz, 1H), 8.07 (s, 1H), 7.90 (d, J = 8.7 Hz, 1H), 7.86 – 7.80 (m, 2H), 7.78 (dd, J = 8.3, 5.1 Hz, 1H), 7.76 – 7.70 (m, 11H), 7.67 (dd, J = 8.0, 5.1 Hz, 1H), 7.57 (s, 4H), 4.96 (s, 2H), 3.18 (s, 9H).

^{13}C{^{1}H} NMR (CD_2Cl_2, 101 MHz, ref res-sol. peak 53.84 ppm): δ (ppm) = 149.17, 147.45, 135.23, 130.65, 130.15, 129.47, 123.73, 123.45, 121.75

^{19}F{^{1}H} NMR (CD_2Cl_2 , 377 MHz): δ = -62.82

^{11}B NMR (CD_2Cl_2, 128 MHz): δ = -5.97 – -8.13 (m)

Elemental Analysis: Calculated for$C_{62}H_{38}BF_{24}N_3Pd$: C: 53.26 %, H: 2.74 %, N: 3.01 %,Found C: 52.26 %, H: 2.88 %, N: 2.89 %

ESI-HRMS Positive mode: calculated for $C_{30}H_{26}N_3Pd^+$ [M$^+$] 534.1156 m/z; found 534.1152 m/z. Negative mode: calculated for $C_{32}H_{12}BF_{24}^-$ [M$^-$] 863.0654 m/z; found 863.0663m/z

(for NMR spectra see 4.1.8)

2.2. Synthesis - Organozinc Compounds

Introduction:

In *Scheme SI-2-2* an overview of the synthesis towards the organozinc compounds$^-$ is shown. For further characterizations such as NMR spectra see section 4.2.

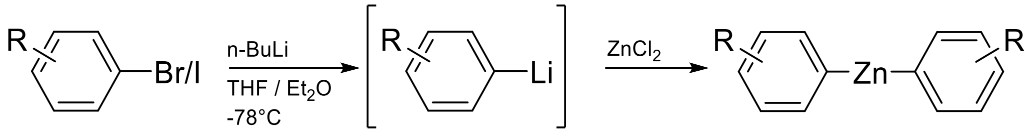

Scheme SI-2-2: Schematic depiction of the synthesis of organozinc compounds.

2.2.1. Synthesis of Bis(4-fluorophenyl)zinc, $Zn(Ar^{4F})_2$

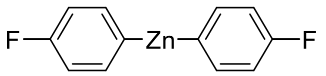

In a Schlenk flask (100 mL), 4-fluoroiodobenzene (1.15 mL, 9.9 mmol, 2 eq., Sigma Aldrich 99%) was dissolved in Et_2O (40 mL, dry). The solution was cooled down to -78°C and *n*-BuLi (6.25 mL, 10 mmol, 2 eq., 1.6 M in n-hexane, Aldrich) was added portionwise. This mixture was stirred at -78°C for 1h. In a vial, $ZnCl_2$ (680.5 mg, 4.99 mmol, 1.eq, Acros 99.99%) was dissolved stepwise in Et_2O (total 13 mL, dry) and transferred to the reaction mixture portionwise. The reaction mixture was stirred at -78°C for 1h, and then let warm to r.t. The volatiles were removed by evaporation using an N_2-trap. By treating directly the residual product, a white solid product was obtained by sublimation at 130°C with reduced pressure (approx. 1.5 *10^{-1} mbar). A white powder was obtained (647.9 mg, 2.54 mmol, 50% Yield).

^{1}H NMR (THF-d^8, 400 MHz, ref res-sol. peak 3.58 ppm): δ (ppm) = 7.58 – 7.50 (2H, Ar(o^{Zn}-**H**), m), 6.90 – 6.84 (2H, Ar(m^{Zn}-**H**), m)

^{13}C{^{1}H} NMR (THF-d^8, 101 MHz, ref res-sol. peak 67.21 ppm): δ (ppm) = 162.97 (**C**$^{quart.}$F, d, $J_{C\text{-}F}$=241.6 Hz), 151.30 (**C**$^{quart.}$Zn, d, $J_{C\text{-}F}$=4.8 Hz), 140.38 (Ar(o^{Zn}-**C**), d, $J_{C\text{-}F}$=5.1 Hz), 113.74 (Ar(m^{Zn}-**C**), d, $J_{C\text{-}F}$=16.9 Hz) ppm

^{19}F NMR (THF-d^8 , 377 MHz, ref $C_6H_3(CF_3)_3$ -63.77 ppm): δ = -118.49 (tt, $J_{F\text{-}H}{}^o$=10.2, $J_{F\text{-}H}{}^m$=7.4 Hz)

Elemental Analysis: Calculated for $C_{12}H_8F_2Zn$: C: 56.40 %, H: 3.16 % Found C: 54.04 %, H: 2.85%[a]

(for NMR spectra see 4.2.1)

2.2.2. Synthesis of Bis(2,6-difluorophenyl)zinc, $Zn(Ar^{2,6F})_2$

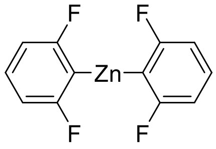

The reaction was conducted under an atmosphere of argon using standard Schlenk techniques. In a Schlenk flask, 1-bromo-2,6-difluorobenzene (1 ml, 8.68 mmol, 2 eq., TCI) was dissolved in Et_2O (10 ml, dry). The solution was cooled down to -78°C and n-BuLi (5.8 ml, 1.6 M in n-hexane, 9.28 mmol, 2.15 eq., Aldrich) was added dropwise (over 10 min) while vigorous stirring. The reaction mixture was kept at -78°C for 30 min and a solution of $ZnCl_2$ (589.2 mg, 4.32 mmol, 1 eq., Acros 99.99%) in Et_2O (7 mL, dry) was added dropwise during 10 min at -78°C. The cooling was removed after 20 min. The volatiles were removed by vacuum, a white-beige chunky solid was obtained and put under high vacuum over night. The crude was sublimed twice at 80°C up to 150°C at 5.5 *10^{-1} mbar. The obtained solid was dissolved in THF (2 mL, dry), diluted with Toluene (8 mL, dry), filtered using a syringe filter (PTFE) and kept at -35°C over night. The observed crystals were isolated by decanting the mother liquor and washing with n-hexane (2 * 2mL). The obtained white powder solid was resublimed once more. (369.4 mg, 1.3 mmol, 29 %).

^{1}H NMR (THF-d^8, 400 MHz, ref res-sol. peak 3.58 ppm): δ (ppm) = 7.15 – 7.02 (2H, Ar(p^{Zn}-**H**), m), 6.65 (4H, Ar(m^{Zn}-**H**), dd, *J*=8.0, 5.1 Hz)

^{13}C{^{1}H} NMR (THF-d^8, 101 MHz, ref res-sol. peak 67.21 ppm): δ (ppm) = 170.39 (dd, $J_{C\text{-}F}$=230.7, 26.9 Hz), 129.38 (t, $J_{C\text{-}F}$=8.8 Hz), 124.40 (t, $J_{C\text{-}F}$=66.2 Hz), 109.77 (dd, $J_{C\text{-}F}$=31.9, 2.9 Hz)

^{19}F{^{1}H} NMR (THF-d^8 , 377 MHz, ref $C_6H_3(CF_3)_3$ -63.77 ppm): δ = -88.83

Elemental Analysis: Calculated for $C_{12}H_6F_4Zn$: C: 49.44 %, H: 2.07 % Found C: 48.6 %, H: 1.6 %[a]

(for NMR spectra see 4.2.2)

[a] Differences between experimental and simulated values may occure due to the moisture sensitivity of the product compound.

2.2.3. Synthesis of Bis(2,4,6-trifluorophenyl)zinc, $Zn(Ar^{2,4,6F})_2$

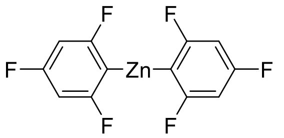

The reaction was conducted under an atmosphere of argon using standard Schlenk techniques. In a Schlenk flask, *n*-BuLi (6.25 mL, 10 mmol, 2 eq., 1.6M in *n*-hexane, Sigma Aldrich) was added dropwise to a solution of 1-bromo-2,4,6-trifluorobenzene (1.2 mL, 9.98 mmol, 2 eq., TCI) in Et_2O (38 mL, dry) at -78°C. The mixture was stirred for 1h at -78°C, then a suspension of $ZnCl_2$ (680 mg, 5 mmol, 1 eq. Acros 99.99%) in Et_2O (12 mL, dry) was added portionwise. The flask containing the $ZnCl_2$ solution was washed with Et_2O (10 mL, dry), which was also added to the reaction mixture. The reaction mixture was stirred at -78°C for 1h and let warm to r.t. for another hour. Then the solvent was removed by evaporation using an N_2-trap. The obtained residual was sublimed under reduced pressure ($1.6*10^{-1}$ mbar) at 170 °C, the visible white solid was pushed further up along the flask wall using a heat gun. The white solid was collected and resublimed once more with the same method to obtain the product as a white solid (268.2 mg, 0.82 mmol, 16 %).

^{1}H NMR (THF-d^8, 400 MHz, ref res-sol. peak 3.58 ppm): δ (ppm) = 6.54 – 6.46 (4H, m)

^{13}C{^{1}H} NMR (THF-d^8, 101 MHz, ref res-sol. peak 67.21 ppm): δ (ppm) = 170.13 (Ar(o^{Zn}-**C**$^{quart.}$), ddd, $J_{C\text{-}F}$=230.9, 31.7, 14.6 Hz), 163.93 (Ar(p^{Zn}-**C**$^{quart.}$), dt, $J_{C\text{-}F}$=242.2, 14.8 Hz), 119.22 (Ar(ipso^{Zn}-**C**$^{quart.}$), td, $J_{C\text{-}F}$=67.7, 3.2 Hz), 98.44 (**C**-H, ddd, $J_{C\text{-}F}$=37.6, 23.7, 4.8 Hz)

^{19}F NMR (THF-d^8 , 377 MHz, ref $C_6H_3(CF_3)_3$ -63.77 ppm): δ = -86.73 (4F, tt, *J*=7.0, 2.3 Hz), -114.92 (2F, dtd, *J*=17.5, 7.9, 1.4 Hz)

Elemental Analysis: Calculated for $C_{12}H_4F_6Zn$: C: 44.01%, H: 1.23 % Found C: 43.5 %, H: 0.92 %[a]

(for NMR spectra see 4.2.3)

2.3. Synthesis - The Heterobimetallic Complexes

Introduction

The heterobimetallic complexes [**1**$^+$]$^-$OTf, [**3**$^+$]$^-$OTf were already reported and characterized elsewhere.[11,24] The heterobimetallic complex $[(bhq)_2Pd\text{-}Au(PPh_3)]BF_4$ [**8**$^+$]$^-$$BF_4$ and complexes of general formula [(bhq)(bhq$^{5\text{-}CH_2NMe_3}$)Pd-Zn(Ar)$_2$]BArF with Ar = C_6F_5 [**4**$^+$]$^-$BArF, Ar^{2,4,6F} [**5**$^+$]$^-$BArF and Ar^{2,6F} [**6**$^+$]$^-$BArF were generated *in-situ* for the purpose of gas-phase experiments (see section 2.4). Detailed synthetic procedures, including the synthesis of [**7**$^+$]$^-$BArF (see section 2.1) and the organozinc compounds (see section 2.2) can be found above, further characterizations such as NMR-spectra (see subsection 2.34.2) or XRD-data can be found below.

2.3.1. Synthesis of $[(bhq)_2Pd^{II}Ag^{I}(IPr)]$BArF, [2$^+$]$^-$BArF:

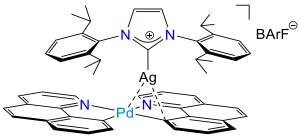

$Pd(bhq)_2$ (29.8 mg, 0.06 mmol, 1 eq.)[1] and NaBArF (57.3 mg, 0.06 mmol, 1 eq., Apollo) were combined and suspended in CH_2Cl_2 (5 mL, dry). (IPr)AgCl (33.9 mg, 0.06 mmol, 0.98 eq., TCI) was dissolved in CH_2Cl_2 (4 mL, dry) and added dropwise (over 5 min) to the [Pd]-solution while stirring. This reaction mixture was stirred for 1.5 h. The reaction mixture was filtered through a syringe filter (PTFE, pore size 0.2 µm, diameter. 13 mm, Whatman), and then layered with *n*-hexane (dry). This mixture was kept at -35°C for weeks, until crystals were observed. The crystals were isolated, washed with *n*-hexane (ca. 14 mL), and dried using high-vacuum. The product was obtained as a yellow powder. (71.7 mg, 61.1 %)

^{1}H NMR (CD_2Cl_2, 400 MHz, ref res-sol. peak 5.32 ppm): δ (ppm) = 8.67 (d, *J* = 5.2 Hz, 1H), 8.40 (dt, *J* = 8.0, 1.4 Hz, 2H), 7.86 (d, *J* = 7.2 Hz, 2H), 7.83 (dd, *J* = 8.7, 1.6 Hz, 2H), 7.76 – 7.64 (m, 19H), 7.57 (q, *J* = 1.5 Hz, 5H), 7.30 (td, *J* = 7.6, 1.4 Hz, 2H), 7.22 (t, *J* = 7.7 Hz, 2H), 6.99 (dd, *J* = 2.0, 0.7 Hz, 2H), 6.86 (d, *J* = 7.7 Hz, 4H), 1.99 (hept, *J* = 6.9 Hz, 4H), 0.93 (d, *J* = 6.9 Hz, 14H), 0.45 (dd, *J* = 7.0, 1.2 Hz, 13H)

^{13}C{^{1}H} NMR (CD_2Cl_2, 101 MHz, ref res-sol. peak 53.84 ppm): δ (ppm) = 162.42, 161.43, 152.90, 147.78, 145.47, 144.08, 139.86, 139.65, 138.84, 136.58, 135.22, 134.87, 134.27, 130.89, 130.13, 129.41, 129.14, 129.08, 127.65, 126.76, 126.37, 124.54, 123.94, 123.90, 123.82, 123.66, 122.57, 120.96, 117.89, 117.84, 28.59, 24.25, 23.69

^{19}F{^{1}H} NMR (CD_2Cl_2 , 377 MHz): δ = -62.85

^{11}B NMR (CD_2Cl_2, 128 MHz): δ = -6.59

Elemental Analysis: Calculated for Product-Abbrev: C: 56.02 %, H: 3.54 %, N: 3.07 %, Found C: 56.26 %, H: 3.57 %, N: 2.96 %

ESI-HRMS Positive mode: calculated for $C_{53}H_{52}AgN_4Pd^+$ [M$^+$] 957.2272 m/z; found 957.2276 m/z. Negative mode: calculated for $C_{32}H_{12}BF_{24}^-$ [M$^-$] 863.0654 m/z; found 863.0667 m/z

Crystal Data for $C_{88}H_{70}AgBCl_6F_{24}N_4Pd$ (*M* =2077.26 g/mol): triclinic, space group P-1 (no. 2), *a* = 16.31264(11) Å, *b* = 19.19348(13) Å, *c* = 30.14452(14) Å, *α* = 95.5855(5)°, *β* = 94.2147(5)°, *γ* = 111.1471(6)°, *V* =

8700.57(10) Å^3, Z = 4, T = 100.0(1) K, μ(Cu Kα) = 6.045 mm^{-1}, $Dcalc$ = 1.586 g/cm^3, 252705 reflections measured (6.79° ≤ 2Θ ≤ 146.812°), 33425 unique (R_{int} = 0.0416, R_{sigma} = 0.0245) which were used in all calculations. The final R_1 was 0.0647 (I > 2σ(I)) and wR_2 was 0.1839 (all data).

(for NMR spectra see 4.3.1)

Comment on Crystal Data: The synthesis and crystallization procedure is described in experimental subsection 2.3.1. A second domain was observed with 5% contribution. An A alert was signaled which is most likely coming from the second domain contribution. Disordered structures, 2 complex cations, 2 BArF units, 6 CH_2Cl_2.

2.3.2. Synthesis of [(bhq)$_2$Pd^{II}Au^{I}(IPr)]BArF, [3$^+$]$^-$BArF:

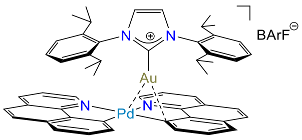

Pd(bhq)$_2$ (15.2 mg, 0.03 mmol, 1 eq.)[1] was suspended in CH_2Cl_2 (3 mL, dry) and transferred to a vial containing [(IPr)Au(MeCN)]BArF (50.8 mg, 0.04 mmol, 1.2 eq.)[2]. The reaction mixture was filtered through a syringe filter (PTFE, pore size 0.2 µm, diameter. 13 mm, Whatman). The solution was layered with *n*-hexane (3 mL, dry) and kept at -35°C for weeks, until crystals were observed. The crystals were isolated, washed with *n*-hexane (2 mL) and dried using high vacuum. The product was obtained as a yellow powder solid (37.8 mg, 60.2 %).

^{1}H NMR (CD_2Cl_2, 400 MHz, ref res-sol. peak 5.32 ppm): δ (ppm) = 8.48 (dd, 1H, J=5.1, 1.4 Hz), 8.42 (dd, 1H, J=8.1, 1.4 Hz), 7.86 (d, 1H, J=8.7 Hz), 7.77 – 7.71 (m, 8H), 7.69 (dd, 1H, J=8.0, 5.1 Hz), 7.56 (p, 2H, J=1.1 Hz), 7.41 (t, 1H, J=7.6 Hz), 7.37 (t, 1H, J=7.8 Hz), 7.11 (s, 1H), 7.02 (d, 2H, J=7.8 Hz), 2.18 (hept, 2H, J=6.8 Hz), 1.02 (d, 6H, J=6.9 Hz), 0.72 (d, 6H, J=6.9 Hz)

^{13}C{^{1}H} NMR (CD_2Cl_2, 101 MHz, ref. residual-sol. peak 53.84 ppm): δ (ppm) = 152.96, 147.72, 145.80, 144.76, 142.44, 141.25, 138.78, 135.20, 134.32, 133.89, 131.13, 130.10, 129.32, 129.07, 127.76, 127.08, 126.36, 124.27, 124.21, 123.71, 123.65, 122.67, 117.86, 54.31, 28.93, 24.08, 23.89

^{19}F{^{1}H} NMR (CD_2Cl_2, 377 MHz): δ = -62.88

^{11}B NMR (CD_2Cl_2, 128 MHz): δ =-6.60

Elemental Analysis: Calculated for $C_{85}H_{64}AgBF_{24}N_4Pd$: C: 53.41%, H: 3.37%, N: 2.93 %, Found C: 53.46 %, H: 3.47 %, N: 3.27 %

(for NMR spectra see 4.3.2)

2.3.3. XRD-structures - Overview

	Group 11			*Group 12*	
Measurements:	M= Cu, X= OTf$^-$	M= Ag, X= BArF$^-$	M= Au, X= NTf$^-$	M= Zn, R=C6F5	Measurements:
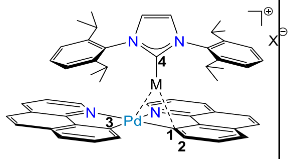	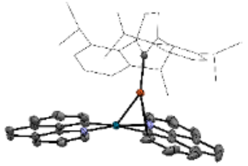	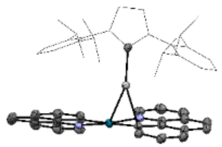	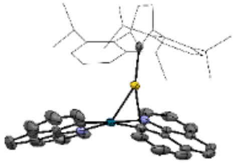	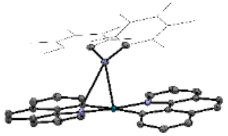	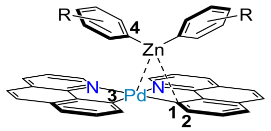
b(Pd-M) / (Å)	2.553	2.696 / 2.694	2.683 / 2.703	2.579	b(Pd-M) / *(Å)*
b(Pd-C1) / (Å)	2.071	2.041 / 2.045	2.133 / 2.168	2.031	b(Pd-C1) / *(Å)*
b(M-C1) / (Å)	2.025	2.412 / 2.327	2.183 / 2.186	2.473	b(M-C1) / *(Å)*
b(M-C2) / (Å)	2.610	2.935 / 2.939	2.911 / 2.887	2.765	b(M-C2) / *(Å)*
b(Pd-C3) / (Å)	2.018	2.025 / 2.029	1.998 / 1.993	1.997	b(Pd-C3) / *(Å)*
b(M-C4) / (Å)	1.901	2.108 / 2.090	1.997 / 2.006	1.983 / 1.999	b(M-C4) / *(Å)*
a(M-Pd-C1) / (°)	50.63	59.34 / 56.87	52.41 / 51.92	63.60	a(M-Pd-C1) / *(°)*

Table SI-2-1: Summary table of XRD-structures of the bimetallic complexes. Values (b: bond length, a: angle, d: dihedral angle) without error were read out using the Mercury software suite (Version 3.10.3). a) Pictures of the XRD structure (ORTEP 50% probability ellipsoids), selected structural features are drawn as wireframe for clarity. Anions, hydrogens and co-crystallizing molecules omitted for clarity.

2.4. ESI-MS/MS - Gas-Phase Investigations

Introduction

General sample preparation: Samples were either prepared by direct dissolution of isolated compounds as for [**2-3**$^+$] or by mixing equimolar amounts of the two corresponding metal complexes [**4-6**$^+$] *in-situ*. For the preparation of [**8**$^+$], additionally a halide abstraction was necessary. All spray solutions were freshly prepared in a glovebox prior to the measurement. Gas-tight syringes were used for all experiments.

Experimental details: Analogous to the reported study on $[(bhq)_2Pd\text{-}Cu(IPr)]^+$ [**1**$^+$],[11] gas-phase BDEs were obtained for the compounds [**2-8**$^+$] using ESI-MS/MS T-CID / L-CID methodology.[10] The compositions of all measured ions were confirmed by matching isotopic patterns. Due to the large molecular size and the high BDEs, the complexes [**2-3**$^+$] and [**8**$^+$] were measured using Xe as the collision gas. In contrast, the significantly lower BDEs for the Zn complexes allowed measurement using Ar (in one case also Ne was used) as the collision gas. In all experiments, only a single channel was measured.

Processing details: Structures, required for assessment of the number of rotors, were obtained using computational methods (see section 3.1) or XRD analysis. The structures of [**2-6**$^+$] were indirectly supported by crystal structures reported for the closely related complexes $[(bhq)_2Pd\text{-}Cu(IPr)]^+$ [**1**$^+$] and $[(bhq)_2Pd\text{-}Zn(C_6F_5)_2]$.[11,12] The number of rotors were chosen based on counting free rotors by structural inspection (see the corresponding schemes), e.g. for ions [**2-3**$^+$] a rotor number of 8 was chosen in accordance with the literature.[11] All experimental gas-phase data were analyzed assuming loose dissociation mechanisms. Additionally, the data for complexes [**2-3**$^+$] were evaluated using a tight dissociation mechanism. The analysis of experimental data, performed using L-CID, yields the fitting parameters E_0, v_{eff}, α' together with the corresponding standard deviations.
The obtained experimental results (E_0, v_{eff}, α') for the separate datasets, the degrees of freedom (d.o.f), the molecular weight of the measured ion (MW), the number of atoms, the number of rotors, the used collision gas and the maximum measured collision offset (-*COFF*) are specified in the corresponding tables (see below). The combined estimates, the averages $\bar{x}$ and the corresponding standard deviations σ of the parameters (E_0, v_{eff}, α'), were obtained using reciprocal-variance weighted averaging, see the formulas (1) and (2):[26,27]

$$\bar{x} = \frac{\sum_i \frac{n_i \bar{x}_i}{s_i^2}}{\sum_i \frac{n_i}{s_i^2}} \qquad (1)$$

$$\sigma = \sqrt{\frac{\sum_i n_i + \sum_i \frac{n_i \bar{x}_i^2}{s_i^2}}{\sum_i \frac{n_i}{s_i^2}} - \bar{x}^2} \qquad (2)$$

Equations (1) and (2): The results of the individual experiments are presented as $\bar{x}_i \pm s_i$, where $\bar{x}_i$ represents the fitting parameter obtained for experiment *i*, s_i is the corresponding standard deviation, and n_i denotes the number of fits. The combined estimates are calculated using Equations (1) for the weighted mean $\bar{x}$ and Equation (2) for the combined standard deviation σ.

2.4.1. ESI-MS/MS T-CID Measurements of $[(bhq)_2Pd\text{-}M(IPr)]^+$, $[2\text{-}3^+]$

Sample preparation

The complexes $[(bhq)_2Pd\text{-}M(IPr)]X$ was dissolved in CH_2Cl_2 (or also Fluorobenzene). This solution (c^{Pd} = ca. 5-50 µM) was used immediately and sprayed using a gas-tight syringe.

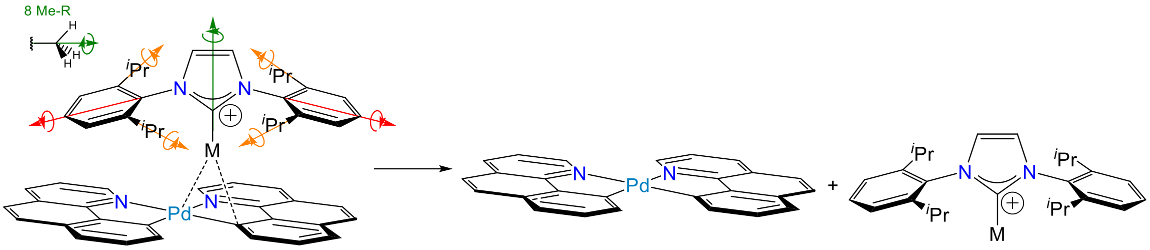

Scheme SI-2-3: Schematic representation of the observed dissociation process, proposed structures of the parent ion and the fragments. The number of rotors for the here measured ions was 8, in accordance with literature.[11]

L-CID Analysis of $[(bhq)_2Pd\text{-}Ag(IPr)]^+$, $[2^+]$

$[(bhq)_2Pd\text{-}Ag(IPr)]^+$, $[2^+]$		MW / (g /mol)	Nr. Atoms	D.o.f	Nr. rotors
		959.32	111	327	8
Dataset	E_0 / (kcal/mol)	ν_{eff} / (cm^{-1})	a' / (cm^{-1})	Col. Gas	-COff^{max} / (eV)
1	44.3 ± 1.4	589 ± 57	5912 ± 399	Xe	100
2	44.0 ± 1.4	561 ± 32	5909 ± 510	Xe	100
Combined	44.2 ± 1.4	568 ± 41	5911 ± 444		

Table SI-2-2: List of individual experimental datasets with the corresponding results for the fit parameters. Selected settings for L-CID, assuming a "loose" dissociation mechanism, are also provided. Additionally, the combined results, including reciprocal-variance weighted averages and their standard deviations, are presented.

$[(bhq)_2Pd\text{-}Ag(IPr)]^+$, $[2^+]$		MW / (g /mol)	Nr. Atoms	D.o.f	Nr. rotors
		959.32	111	327	8
Dataset	E_0 / (kcal/mol)	ν_{eff} / (cm^{-1})	a' / (cm^{-1})	Col. Gas	-COff^{max} / (eV)
1	29.9 ± 1.0	666 ± 56	321 ± 117	Xe	100
2	29.3 ± 1.4	629 ± 68	287 ± 141	Xe	100
Combined	29.7 ± 1.2	651 ± 64	307 ± 128		

Table SI-2-3: List of individual experimental datasets with the corresponding results for the fit parameters. Selected settings for L-CID, assuming a "tight" dissociation mechanism, are also provided. Additionally, the combined results, including reciprocal-variance weighted averages and their standard deviations, are presented.

L-CID Analysis of $[(bhq)_2Pd\text{-}Au(IPr)]^+$, $[3^+]$

$[(bhq)_2Pd\text{-}Au(IPr)]^+$, $[3^+]$		MW / (g /mol)	Nr. Atoms	D.o.f	Nr. rotors
		1048.41	111	327	8
Dataset	E_0 / (kcal/mol)	ν_{eff} / (cm^{-1})	a' / (cm^{-1})	Col. Gas	-COff^{max} / (eV)
1	53.8 ± 1.6	938 ± 62	5722 ± 414	Xe	100
2	49.2 ± 1.6	750 ± 88	5830 ± 421	Xe	100
Combined	51.5 ± 2.8	876 ± 114	5775 ± 421		

Table SI-2-4: List of individual experimental datasets with the corresponding results for the fit parameters. The selected settings for L-CID, assuming a "loose" dissociation mechanism, are also provided. Additionally, the combined results, including reciprocal-variance weighted averages and their standard deviations, are presented.

$[(bhq)_2Pd\text{-}Au(IPr)]^+$, $[3^+]$		MW / (g /mol)	Nr. Atoms	D.o.f	Nr. rotors
		1048.41	111	327	8
Dataset	E_0 / (kcal/mol)	ν_{eff} / (cm^{-1})	a' / (cm^{-1})	Col. Gas	-COff^{max} / (eV)
1	39.1 ± 0.9	964 ± 47	284 ± 116	Xe	100
2	35.1 ± 1.2	835 ± 67	228 ± 116	Xe	100
Combined	37.7 ± 2.2	921 ± 81	256 ± 119		

Table SI-2-5: List of individual experimental datasets with the corresponding results for the fit parameters. Selected settings for L-CID, assuming a "tight" dissociation mechanism, are also provided. Additionally, the combined results, including reciprocal-variance weighted averages and their standard deviations, are presented.

2.4.2. ESI-MS/MS T-CID Measurements of $[(bhq)(bhq^{5-CH_2NMe_3})Pd\text{-}Zn(Ar)_2]^+$, [4-6+]:

Sample preparation:

$[Pd(bhq)(bhq^{5-CH_2NMe_3})^+]^-$BArF (5 mg, 3.6 umol, 1 eq., $[7^+]^-$BArF) was dissolved in CH_2Cl_2 (2 mL), this was used as a stock solution (c = 1.8 mM). A stock solution of $Zn(Ar)_2$ (0.24 mM in THF) was made. The Pd-stock solution (0.5 mL) and the Zn-stock solution (0.1 mL) were combined and diluted with CH_2Cl_2 (2-3mL). This solution (c^{Pd} = 50-70 μM) was used immediately.

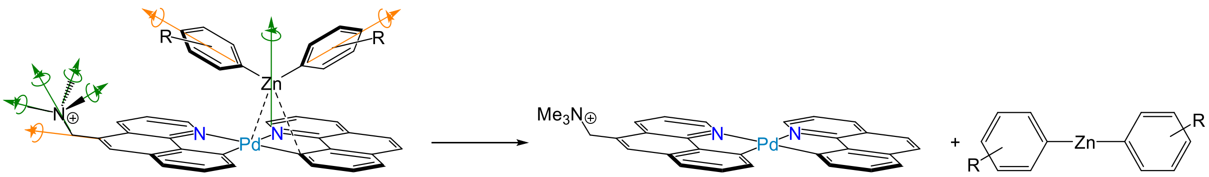

Scheme SI-2-4: Schematic representation of the observed dissociation process, proposed structures of the parent ion and the fragments.

L-CID Analysis of $[(bhq)(bhq^{5-CH_2NMe_3})Pd\text{-}Zn(C_6F_5)_2]^+$, [4+]

$[(bhq)(bhq^{5-CH2NMe3})PdZn(C_6F_5)_2]^+$		MW / (g /mol) 934.47	Nr. Atoms 83	D.o.f 243	Nr. rotors 8
Dataset	E_0 / (kcal/mol)	ν_{eff} / (cm^{-1})	a' / (cm^{-1})	Col. Gas	-COff^{max} / (eV)
1	35.4 ± 1.1	860 ± 81	5783 ± 482	Ar	79.9
2	35 ± 0.9	831 ± 87	5618 ± 424	Ar	59.9
Combined	35.2 ± 1.0	847 ± 85	5690 ± 458		

Table SI-2-6: List of individual experimental datasets with the corresponding results for the fit parameters. Selected settings for L-CID, assuming a "loose" dissociation mechanism, are also provided. Additionally, the combined results, including reciprocal-variance weighted averages and their standard deviations, are presented.

L-CID Analysis of $[(bhq)(bhq^{5-CH_2NMe_3})Pd\text{-}Zn(Ar^{2,4,6F})_2]^+$, [5+]

$[(bhq)(bhq^{5-CH2NMe3})PdZn(Ar^{2,4,6F})_2]^+$		MW / (g /mol) 862.51	Nr. Atoms 83	D.o.f 243	Nr. rotors 8
Dataset	E_0 / (kcal/mol)	ν_{eff} / (cm^{-1})	a' / (cm^{-1})	Col. Gas	-COff^{max} / (eV)
1	30.1 ± 1.0	663 ± 41	5683 ± 415	Ar	59.9
2	29.7 ± 0.9	814 ± 45	5817 ± 464	Ar	59.9
3	29.5 ± 1.1	870 ± 67	5934 ± 497	Ar	59.9
Combined	29.8 ± 1.0	755 ± 98	5743 ± 442		

Table SI-2-7: List of individual experimental datasets with the corresponding results for the fit parameters. Selected settings for L-CID, assuming a "loose" dissociation mechanism, are also provided. Additionally, the combined results, including reciprocal-variance weighted averages and their standard deviations, are presented.

L-CID Analysis of $[(bhq)(bhq^{5-CH_2NMe_3})Pd\text{-}Zn(Ar^{2,6F})_2]^+$, [6+]

$[(bhq)(bhq^{5-CH2NMe3})PdZn(Ar^{2,6F})_2]^+$		MW / (g /mol) 826.53	Nr. Atoms 83	D.o.f 243	Nr. rotors 8
Dataset	E_0 / (kcal/mol)	ν_{eff} / (cm^{-1})	a' / (cm^{-1})	Col. Gas	-COff^{max} / (eV)
1	30.1 ± 0.2	861 ± 32	6296 ± 128	Ne	50
2	30.6 ± 0.8	667 ± 39	6105 ± 376	Ar	44.9
Combined	30.1 ± 0.3	783 ± 101	6276 ± 181		

Table SI-2-8: List of individual experimental datasets with the corresponding results for the fit parameters. Selected settings for L-CID, assuming a "loose" dissociation mechanism, are also provided. Additionally, the combined results, including reciprocal-variance weighted averages and their standard deviations, are presented.

2.4.3. ESI-MS/MS T-CID Measurements of $[(bhq)_2Pd\text{-}Au(PPh_3)]^+$ $[8^+]$:[2]

Sample preparation

$Pd(bhq)_2$ (4.3 mg, 0.01 mmol, 1 eq.) and $[(PPh_3)AuCl]$ (4.3 mg, 0.01 mmol, 0.94 eq.) were combined in a vial, and dissolved in CH_2Cl_2 (3 mL). This mixture is then slowly added to a suspension of $AgBF_4$ (2.1 mg, 0.01 mmol, 0.9 eq.). This reaction mixture was stirred for a few min and then filtered through a syringe filter (PTFE, pore size 0.2 µm, diameter. 13 mm, Whatman). The obtained solution was diluted with CH_2Cl_2 until the concentration of the [Pd] reached approx. 60 µM, and sprayed directly using a gas-tight syringe.

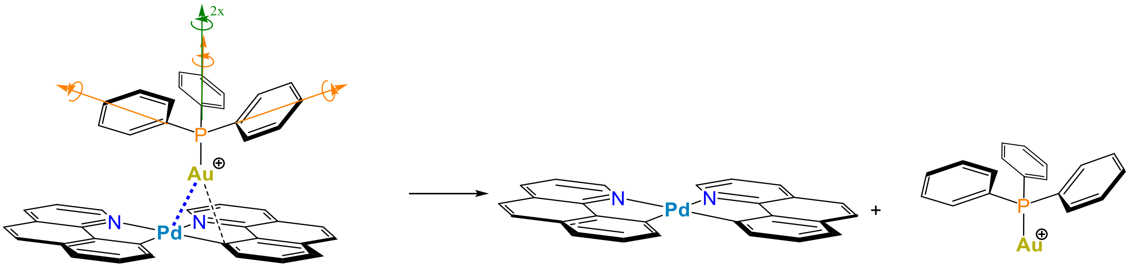

Scheme SI-2-5: Schematic representation of the observed dissociation process, proposed structures of the parent ion and the fragments.

L-CID Analysis of $[(bhq)_2Pd\text{-}Au(PPh_3)]^+$, $[8^+]$

$[(bhq)_2Pd\text{-}AuPPh_3]^+$		MW / (g /mol)	Nr. Atoms	D.o.f	Nr. rotors
		922.11	80	234	5
Dataset	E_0 / (kcal/mol)	v_{eff} / (cm^{-1})	a' / (cm^{-1})	Col. Gas	-COff^{max} / (eV)
1	57.9 ± 1.7	969 ± 46	6030 ± 487	Xe	79.9
2	54.9 ± 1.6	969 ± 36	5882 ± 522	Xe	79.9
Combined	56.3 ± 2.2	969 ± 40	5961 ± 509		

Table SI-2-9: List of individual experimental datasets with the corresponding results for the fit parameters. Selected settings for L-CID, assuming a "loose" dissociation mechanism, are also provided. Additionally, the combined results, including reciprocal-variance weighted averages and their standard deviations, are presented.

2.4.4. ESI-MS/MS T-CID Measurements of $[Pd(bhq)(bhq^{5\text{-}CH_2\text{-}NMe_3})]^+$, $[7^+]$:

Sample preparation:

Measurements of this complex were performed from the same spraying solutions as $[(bhq)(bhq^{CH2NMe3})Pd\text{-}Zn(Ar)_2]^+$.

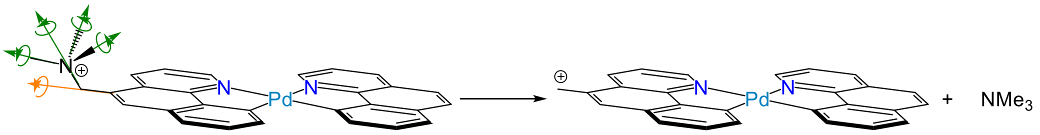

Scheme SI-2-6: Schematic representation of the observed dissociation process, proposed structures of the parent ion and the fragments.

L-CID analysis of $[Pd(bhq)(bhq^{CH_2\text{-}NMe_3})]^+$, $[7^+]$

$[Pd(bhq)(bhq5\text{-}^{CH2NMe3})]^+$		MW / (g /mol)	Nr. Atoms	D.o.f	Nr. rotors
		534.98	60	174	5
Dataset	E_0 / (kcal/mol)	v_{eff} / (cm^{-1})	a' / (cm^{-1})	Col. Gas	-COff^{max} / (eV)
1	40.4 ± 0.9	754 ± 55	5820 ± 438	Ar	59.9
2	41.3 ± 1.1	874 ± 83	5606 ± 455	Ar	59.9
Combined	40.8 ± 1.1	791 ± 85	5717 ± 459		

Table SI-2-10: List of individual experimental datasets with the corresponding results for the fit parameters. Selected settings for L-CID, assuming a "loose" dissociation mechanism, are also provided. Additionally, the combined results, including reciprocal-variance weighted averages and their standard deviations, are presented.

[2] Prepared and measured by Dr. Alexandra Tsybizova and Angela Spadea.

2.4.5. Summaries and Observations of the quantitative ESI-MS investigations

Overview Investigations on Coinage Metal Based Heterobimetallic Complexes.

In Figure SI-2-2, ESI-MS/MS spectra for the complex cations [**1-3**$^+$] and [**8**$^+$] are shown, which were recorded at the indicated CID settings (the collision offset and gas pressure in the collision cell). For ions [**1-3**$^+$] additional fragmentation products (>2% relative intensity) were observed at these conditions. The rationalization of the origin of these fragmentation products, and potential influences on the interpretations, necessitated a computational investigation (see section 3.2). In order to suppress effects of neglecting these additional fragmentation channels, the energy-resolved CID cross sections were cut for higher collision offsets (for the chosen settings see tables in section 2.4).

The experimentally obtained gas-phase BDEs, assuming "loose" dissociation mechanism, for the ions [**1-3**$^+$] and [**8**$^+$] are summarized in Table SI-2-11 (for more detailed information see subsections 2.4.1 and 2.4.3). The values for [**1**$^+$] are taken from literature,[11] and are demonstrated for comparison.[3]

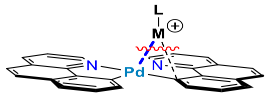 [(bhq)$_2$Pd-**M(L)**]$^+$	Cu(IPr)[a)] [1$^+$]	Ag(IPr) [2$^+$]	Au(IPr) [3$^+$]	Au(PPh$_3$) [8$^+$]
E_0 / [kcal /mol]	50.6 ± 1.5	44.2 ± 1.4	51.5 ± 2.8	56.3 ± 2.2
ν_{eff} / [cm^{-1}]	880 ± 58	568 ± 41	876 ± 114	969 ± 40
α' / [cm^{-1}]	5838 ± 366	5911 ± 444	5775 ± 421	5961 ± 509
D.o.f. and Nr. rotors	327 8	327 8	327 8	234 5

Table SI-2-11: Reciprocal-variance weighted averages of the experimentally obtained fitting parameters and their corresponding standard deviations. Selected settings for the L-CID analysis are shown. All experiments were conducted using Xe as the collision gas, assuming a "loose" dissociation mechanism.[a)] Data obtained from literature.[11]

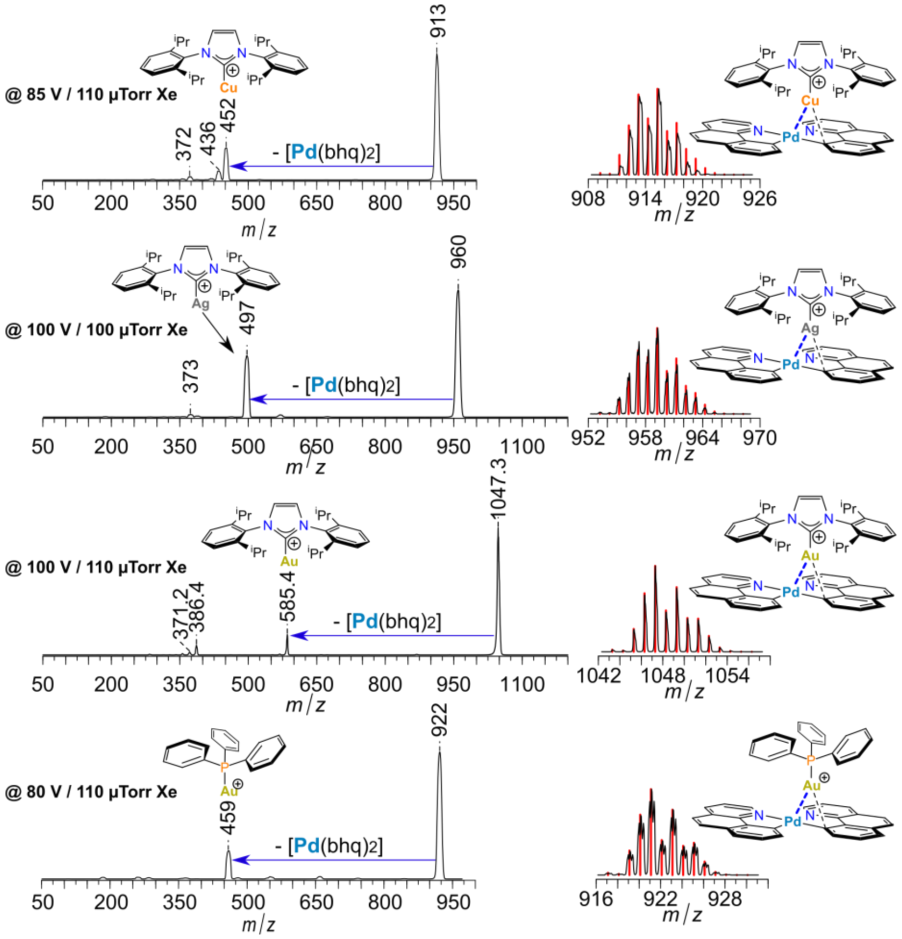

Figure SI-2-2: ESI-MS/MS CID spectra of ions [**1-3**$^+$], and [**8**$^+$] at indicated collision offset (- *COFF*) and gas-pressure of Xe in the collision cell. Inset: experimental (black) and simulated (red) isotopic pattern. Proposed structures for both parent and fragment ions are shown. Marked numbers refer to m/z of the peak maxima, only peaks with >2% of rel. intensity are labelled.

---

[3] The reported BDE for the original complex were measured on another device.

Overview Investigations on Pd-Zn Heterobimetallic Complexes

For the ions [**4-6**$^+$], clean dissociations were observed at the highest pressures (approx. 110 µTorr, Ar gas) and collision offset (-*COFF* of 60 V) settings. Notable, when going to higher collision offsets, an additional fragment peak appears assumed to show NMe_3 cleavage to form the benzylic cation (see Figure SI-2-3). The obtained gas-phase rate data were fitted at energies below the appearance of secondary channels. The experimental obtained gas-phase BDEs, assuming "loose" dissociation mechanism, for the ions [**4-6**$^+$] are summarized in Table SI-2-12.

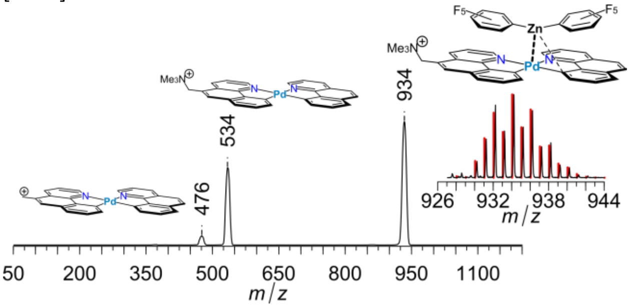

Figure SI-2-3: ESI-MS/MS CID spectrum of ion [(bhq)(bhq$^{5\text{-}CH_2NMe_3}$)Pd-Zn(C_6F_5)]$^+$ with 110 µTorr of Ar collision gas in the collision cell at collision offset of 80 V. Inset: experimental (black) and simulated (red) isotopic pattern of parent ion. Proposed structures for both parent and fragment ions are shown. Marked numbers refer to m/z of the peak maxima.

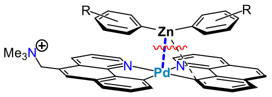 [(bhq)(bhq^{CH2NMe3})Pd-Zn(**Ar**)$_2$]$^+$	C_6F_5 [4$^+$]		$Ar^{2,4,6F}$ [5$^+$]		$Ar^{2,6F}$ [6$^+$]	
E_0 / [kcal /mol]	35.2 ± 1.0		29.8 ± 1.0		30.1 ± 0.3	
$ν_{eff}$ / [cm^{-1}]	847 ± 85		755 ± 98		783 ± 101	
α' / [cm^{-1}]	5690 ± 458		5743 ± 442		6276 ± 181	
D.o.f. and Nr. rotors	243	8	243	8	243	8

Table SI-2-12: Reciprocal-variance weighted averages of the experimentally obtained fitting parameters and their corresponding standard deviations. Selected settings for the L-CID analysis are shown. All experiments were conducted using either Ar or Ne as the collision gas, assuming a "loose" dissociation mechanism. For more details see subsection 2.4.2.

Overview Investigations on C-N cleavage

The complex cation [**7**$^+$] was further characterized by measuring the BDE for the proposed C-N cleavage (see Figure SI-2-4), using ESI-MS/MS T-CID / L-CID.[10] A BDE of 40.9 ± 1 kcal/mol was measured. This is reminiscent to reported studies on so called "thermometer ions", where a series of benzylammonium ions were subjected to gas-phase CID experiments to obtain the C-N BDEs (Scheme SI-2-7).[28] For the dissociation of benzylammonium, forming a benzylic cation and an ammonia, the experimental BDE was reported to be 39.7 ± 0.9 kcal /mol. However it was also shown that these BDEs are very sensitive to the electronics in the aromatics, which makes the comparison to the here reported system less informative. Nevertheless, the similar BDEs are encouraging.[28]

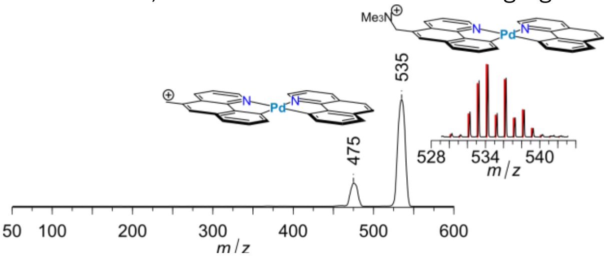

E_0 / [kcal /mol]	40.8 ± 1.1	
$ν_{eff}$ / [cm^{-1}]	791 ± 85	
α' / [cm^{-1}]	5717 ± 459	
D.o.f. and Nr. rotors	174	5

Figure SI-2-4: (left) ESI-MS/MS CID spectrum of [Pd(bhq)(bhq$^{5\text{-}CH2NMe3}$)]$^+$,[7$^+$], with 110 µTorr of Ar as the collision gas at a 50 V collision offset. Inset: experimental (black) and simulated (red) isotopic patterns for the parent ion. Proposed structures for parent and fragment ions are shown, with numbers indicating m/z peak maxima. The gas-phase BDE for C–N cleavage was determined using the T-CID/L-CID method. Results include reciprocal-variance weighted averages of fitting parameters, standard deviations, and L-CID settings. Here, a "loose" dissociation mechanism was assumed.

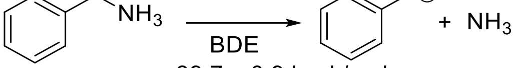

Scheme SI-2-7: Schematic representation of the C-N dissociation of benzylammonium to form a benzyl cation and ammonia. The reported BDE is indicated, calculated from a thermodynamic cycle [28]

Additional Observations:

- *Observation 1:* Analogous to the characterizations of [$(bhq)_2$Pd-Au(PPh_3)]$^+$ [**8$^+$**], qualitative ESI-MS/MS investigations were conducted for all complexes with the general formula [$(bhq)_2$PdM(PR_3)]$^+$ with all following combinations M= Cu(I), Ag(I), Au(I) and PR_3 = PPh_3, P(*t*-Bu)$_3$. All of them, except for [$(bhq)_2$Pd-Au(PPh_3)]$^+$ [**8$^+$**],showed other fragmentation patterns, e.g. the dissociation of the phosphine ligand or fragmentations leading to the cleavage of a *t*-Bu group. Reminiscent observations were reported for related molecules, wherein the PPh_3 dissociation was not visible for Au but for Cu and Ag.[29]
- *Observation 2:* In an attempt to measure Pd^{II}-Zn^{II} with organozincate adducts, formally [$ZnAr_xCl_y$]$^-$ (where x+y=3) was mixed with [Pd$(bhq)_2$]. Although many organozincate species were observed in ESI-MS, no heterobimetallic complexes were found.
- *Observation 3:* In addition to ions [**4-6$^+$**] also the complex [(bhq)(bhq$^{5\text{-}CH_2NMe_3}$)Pd-Zn$(Ar)_2$]$^+$ with Ar=*p*-C_6H_4F was investigated. No BDE could be obtained.

3. Computational

3.1. Bond Dissociation Energies in Heterobimetallic complexes

This section outlines the computational investigation of the heterobimetallic complexes [**1-6**$^+$] and [**8**$^+$], including a focused study into the central interaction between the two metal-complex fragments [M] and [Pd]. Computational and experimental investigations have already been reported for the complex [(bhq)$_2$Pd-Cu(IPr)]$^+$ [**1**$^+$], as well as [(bhq)$_2$Pd-Zn(C$_6$F$_5$)$_2$], a charge-neutral analogue of ion [**4**$^+$].[11,12,30] The computational investigations thereof were utilized, in part as a guide, for the work described here.[30] Due to the slightly different deployed methodologies some structures were recalculated for comparison. Notably, the mentioned publication also laid the foundation for investigating the accuracy of computational methods, for example, by demonstrating the absence of multi-reference character in both WFT and DFT based methods.[30] The CREST sampling and the optimization methodology are central differences as compared to the mentioned publication, although it did not change the earlier results.[30] The medium-to-large size of the molecules and the presence of transition metals, rendered more sophisticated ab initio methods like CI or CC unpractical, hence the computational investigations were conducted using DFT methodologies.[31]

3.1.1. Procedures and Methods

Conformational Search

Conformational searches were conducted using the Conformer-Rotamer Ensemble Sampling Tool (CREST v.2.7.1)[32,33] based on the GFN-xtB method. The heterobimetallic complexes [**1**$^+$] and [**4-6**$^+$], as well as hypothesized dissociation products [Pd(bhq)$_2$], [(IPr)Cu]$^+$ and [**7**$^+$] were investigated by CREST. The starting structures for the ions [**2-3**$^+$],[**8**$^+$] and the dissociation products Zn(Ar)$_2$ (where Ar = C$_6$F$_5$, Ar^{2,4,6F}, Ar^{2,6F}) and [(PPh$_3$)Au]$^+$ were derived from analogous structures obtained through CREST analysis. Additionally, the dissociations of the secondary channels for [**4-6**$^+$] and [**7**$^+$], involving the cleavage of the trimethylamine molecule, were investigated. The required structures [Pd(bhq)(bhq$^{5\text{-}CH_2}$)]$^+$, [(bhq)(bhq$^{5\text{-}CH_2}$)Pd-Zn(Ar)$_2$]$^+$ (where Ar = C$_6$F$_5$, Ar^{2,4,6F}, Ar^{2,6F}) and NMe$_3$ were derived from analogous structures obtained through CREST analysis.

The geometries of the obtained conformers and rotamers were further optimized using ORCA 5.0.2. Geometry optimizations were performed using the PBE functional together with the def2-SVP[34] basis set; for Pd,Ag and Au Def2-ECP[35–42]was used, and Grimme's D3 dispersion correction was used (with Becke-Johnson damping).[43,44] For all calculations defgrid2 DFT integration grid was used. SCF convergency criteria were set to TightSCF and optimization convergency criteria were set to TightOpt (TolE = 1e-6, TolRMSG = 3e-5, TolMaxG = 1e-4, TolRMSD = 6e-4, TolMaxD = 1e-3). For all calculations, Coulomb fitting was employed with general Weigend J auxiliary basis set.[45] The minimal energy structures were identified and selected for further processing. Additional information and input templates are provided in subsection 4.5.1.

Geometry Optimizations

The geometries for all structures were re-optimized using the same method as for the post-CREST geometry optimizations mentioned above, but the basis set was set to def2-TZVP[34], and optimization convergency criteria were set to VeryTightOpt (TolE = 1e-7, TolRMSG = 3e-6, TolMaxG = 5e-6, TolRMSD = 5e-5, TolMaxD = 1e-4). In order to validate the ground state nature of the structures, and also to obtain zero-point energies, frequency calculations were performed on the same level of theory as the optimization (PBE-D3BJ/Def2-TZVP). PBE-D3(BJ) was used for optimizations and frequency calculations, because of its usual low computational cost, its computational stability and its reported proficiency for describing closely related molecules.[30] Additional information and input templates are provided in subsection 4.5.2.

Single Point Calculations

The used functionals were PBE (GGA), M06L (meta-GGA) and M06 (hybrid meta-GGA). The M06-L functional, developed for transition metal complexes, was selected for its commonly reliable performance and cost-effectiveness, even though it may exhibit lower computational stability.[46] Furthermore, the presence of heavy metals required the incorporation of relativistic treatments, here relativistic pseudopotentials or relativistic Hamiltonians in combination with all-electron basis sets were used. For the more sophisticated and computational demanding calculations, no complete basis set

extrapolation was performed (Table SI-3-1 and Table SI-3-2, entry 4-10). Hence, potential errors must be expected, e.g. basis set superposition error (BSSE). The obtained BDEs are summarized in Table SI-3-1 and Table SI-3-2.

Single Point Calculations: All single point calculations were based on the optimized structures (PBE-D3BJ/def2-TZVP) as described above. Single point calculations were performed using various DFT-methods / basis-sets and programs, using ORCA 5.0.2 as well as the ADF suite (ADF 2016). The DFT-calculations were performed using the PBE and the M06-L functional together with the def2-NVP[34] (N=S,TZ,QZ) basis sets, for Pd, Ag and Au Def2-ECP[35–42] was used. For the PBE calculations, D3 dispersion correction was used with Becke-Johnson damping[43,44], and for M06L D3zero was used[43,47]. Complete basis set extrapolation (triple / quadruple) was performed using the ORCA command “Extrapolate(3/4,def2)”[34,45,48–50]. In some cases Grimme’s D4[51,52] was used.
Relativistic calculations: were performed using the PBE, M06L and M06 functionals. Calculations using the Second order Douglas-Kroll-Hess (DKH2) Hamiltonian were used together with a DKH-def2-NVP[34] basis set with element-specific modifications for Pd,Ag,Au SARC-DKH-NVP[53–57], the auxiliary basis set AuxJ was SARC/J.[45,54–57] Additional Auxiliary basis set information (AuxC,AuxJK) and approximations were included using the “AutoAux” command in ORCA.[58] Calculations with the scalar relativistic ZORA (zeroth-order regular approximation) Hamiltonian were used together with a ZORA-def2-NVP basis set with element-specific modifications for Pd,Ag,Au SARC-ZORA-NVP[53–57], otherwise the same settings as for the DKH2 calculations.
ADF[59]: Energies were computed using the PBE functional and a ZORA/QZ4P basis-set. Grimmes’s D3 dispersion correction was used (with Becke-Johnson damping)[43,44]. Relativistics were treated via the Zero Order Regular Approximation (ZORA) formalism.

Additional information and input templates are provided in subsections 4.5.2 (ORCA) and 4.5.3 (ADF).

3.1.2. Atoms in Molecules Analysis

The central part constituting of the Pd-M-C motif was investigated by Bader’s theory, Atoms In Molecules (AIM).[60–62] Single point calculations on the DKH2-M06-D30 / DKH-def2-TZVP // PBE-D3(BJ) / def2-TZVP (Table SI-3-1 and Table SI-3-2, entry 10) level of theory were conducted and used for the AIM analysis, which was conducted using the Multiwfn 3.8(dev) program.[63] Additional information and input templates files are provided in subsection 4.5.5.

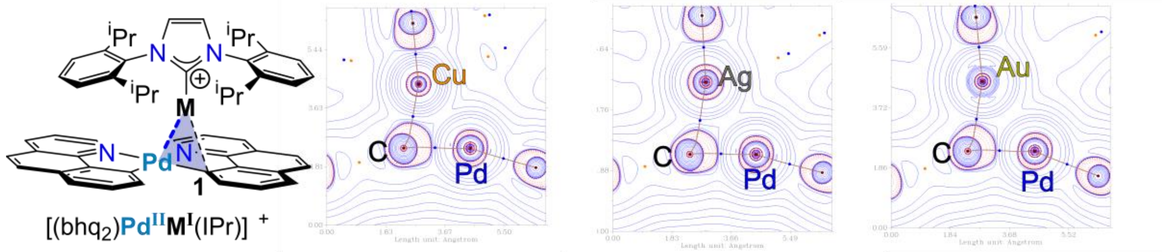

Figure SI-3-1: Laplacian contour plots for [**1-3**$^+$] in the plane of Pd-C-M as indicated in the structure on the left. BCP are shown in blue. Electron density at the DKH2-M06-D30 / DKH-def2-TZVP // PBE-D3(BJ) / def2-TZVP level of theory.

As can be seen in Figure SI-3-1, no BCP was found between the two metal centers in the heterobimetallic complexes [**1-3**$^+$]. This was first observed for the heterobimetallic complex [(bhq)$_2$Pd-Cu(IPr)]$^+$ [**1**$^+$] ,for which computational investigations showed that the BCP is masked by the electron density of C_1.[11,30]

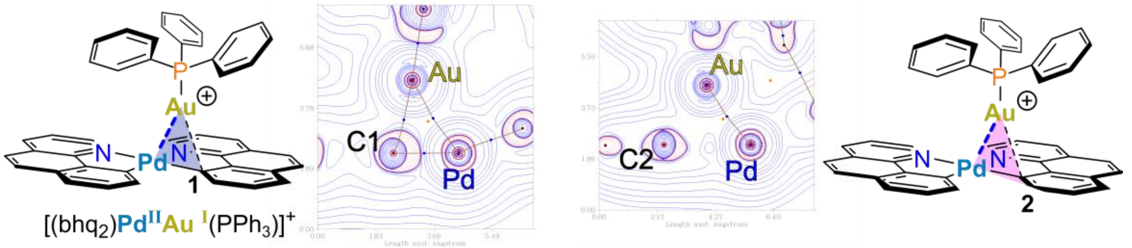

Figure SI-3-2: Laplacian contour plots for [**8**$^+$] in the plane of Pd-C1-Au and Pd-C2-Au as indicated in the structures on the sides. Electron density at the DKH2-M06-D30 / DKH-def2-TZVP // PBE-D3(BJ) / def2-TZVP level of theory.

For the complex [**8**$^+$], BCPs were observed between the metal centers Pd-Au and also between Au-C1.

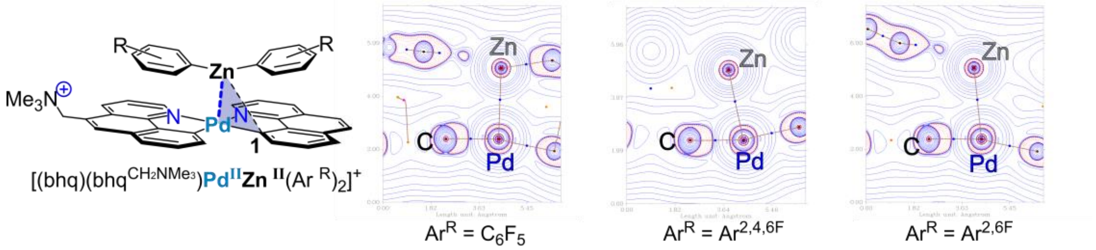

Figure SI-3-3: Laplacian contour plots for [**4-6**⁺] in the plane of Pd-C-Zn as indicated in the structure on the left. Electron density at the DKH2-M06-D30 / DKH-def2-TZVP // PBE-D3(BJ) / def2-TZVP level of theory.

In the complexes [**4-6⁺**], all show a BCP between Pd and Zn, but none between Zn and C_1.

3.1.3. Dissociation Characteristics – BDE

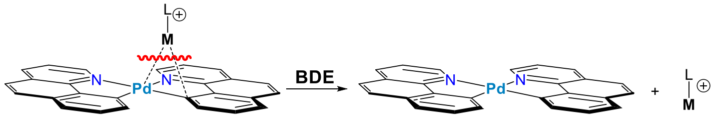

[(bhq)$_2$Pd-M(L)]$^+$

		Cu(IPr) [1$^+$]			Ag(IPr) [2$^+$]			Au(IPr) [3$^+$]			Au(PPh$_3$) [8$^+$]		
		BDE	ΔBDE	Rel. [%][b]	BDE	ΔBDE	Rel. [%][b]	BDE	ΔBDE	Rel. [%][b]	BDE	ΔBDE	Rel. [%][b]
1	PBE / def2-TZVP	49.6	-1.0	(2)	42.9	-1.3	(3)	52.2	0.7	(1)	45.5	10.9	(19)
	ZPE		2.4			1.8			1.7			0.9	
	D3(BJ) / D4 for PBE	23.7	/	22.0	22.1	/	20.5	22.0	/	20.4	15.6	/	14.9
	D30 / D4 for M06-L	6.1	/	7.1	5.8	/	6.7	5.7	/	6.6	3.6	/	4.5
2	PBE-D3(BJ) / def2-CBS	69.1	18.5	(37)	62.5	18.3	(41)	71.4	19.9	(39)	59.5	3.2	(6)
3	M06L-D30 / def2-CBS	73.6	23.0	(45)	63.4	19.2	(44)	70.0	18.5	(36)	54.4	-1.9	(3)
4	DKH2-PBE-D3(BJ) / DKH-def2-TZVP	72.5	21.9	(43)	63.0	18.8	(43)	74.2	22.7	(44)	61.8	5.5	(10)
5	ZORA-PBE-D3(BJ) / ZORA-def2-TZVP	73.0	22.4	(44)	63.5	19.3	(44)	74.3	22.8	(44)			
6	PBE-D3(BJ) / ZORA-QZ4P	72.2	21.6	(43)	63.5	19.3	(44)	73.4	21.9	(43)	61.3	5.0	(9)
7	DKH2-M06L-D30 / DKH-def2-TZVP	76.7	26.1	(52)	64.9	20.7	(47)	75.1	23.6	(46)	59.7	3.4	(6)
8	ZORA-M06L-D30 / ZORA-def2-TZVP	77.2	26.6	(53)	65.4	21.2	(48)	75.3	23.8	(46)	59.8	3.5	(6)
9	M06-D30 / def2-CBS	77.7	27.1	(53)	67.7	23.5	(53)	75.8	24.3	(47)	60.5	4.2	(7)
10	DKH2-M06-D30 / DKH-def2-TZVP	81.9	31.3	(62)	70.1	25.9	(59)	80.5	29.0	(56)			
Experimental (T-CID / L-CID)		50.6			44.2			51.5			56.3		

Table SI-3-1: BDEs for [**1**-**3**$^+$] and [**8**$^+$] The experimentally obtained values are presented at the bottom in [kcal/mol]. a) Difference comp. vs exp., b) relative error [%] with respect to exp. with colors ranging from red for large discrepancies to blue for small discrepancies

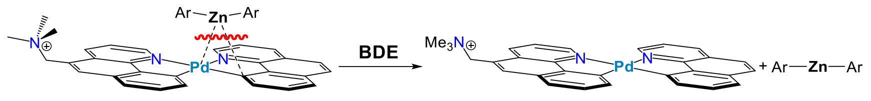

[(bhq)(bhq$^{5\text{-}CH_2NMe_3}$)Pd-Zn(Ar)$_2$]$^+$

		C_6F_5 [4$^+$]			Ar^{2,4,6F} [5$^+$]			Ar^{2,6F} [6$^+$]		
		BDE	ΔBDE[a]	Rel. [%][b]	BDE	ΔBDE	Rel. [%][b]	BDE	ΔBDE	Rel. [%][b]
1	PBE / def2-TZVP	11.8	-23.4	(66)	8.4	-21.4	(72)	10.3	-20.1	(66)
	ZPE		1.2			1.0			0.7	
	D3(BJ) / D4 for PBE	23.9	/	24.1	22.7	/	22.6	19.7	/	19.6
	D30 / D4 for M06-L	4.2	/	6.0	4.1	/	5.8	4.2	/	5.4
2	PBE-D3(BJ) / def2-CBS	31.9	-3.3	(9)	28.2	-1.6	(5)	27.4	-2.7	(9)
3	M06L-D30 / def2-CBS	34.9	-0.3	(1)	30.8	1.0	(3)	30.3	0.2	(1)
4	DKH2-PBE-D3(BJ) / DKH-def2-TZVP	33.2	-2.0	(6)	29.0	-0.8	(3)	27.9	-2.2	(7)
5	ZORA-PBE-D3(BJ) / ZORA-def2-TZVP	33.4	-1.8	(5)	29.2	-0.6	(2)	28.1	-2.0	(7)
6	PBE-D3(BJ) / ZORA-QZ4P	32.6	-2.6	(7)	28.7	-1.1	(4)	27.6	-2.5	(8)
7	DKH2-M06L-D30 / DKH-def2-TZVP	35.7	0.5	(1)	31.1	1.3	(4)	30.5	0.4	(1)
8	ZORA-M06L-D30 / ZORA-def2-TZVP	35.9	0.7	(2)	31.3	1.5	(5)	30.8	0.7	(2)
9	M06-D30 / def2-CBS	37.6	2.4	(7)	33.6	3.8	(13)	32.5	2.4	(8)
10	DKH2-M06-D30 / DKH-def2-TZVP	39.1	3.9	(11)	34.6	4.8	(16)	33.2	3.1	(10)
Experimental (T-CID / L-CID)		35.2			29.8			30.1		

Table SI-3-2: BDEs for [**4**-**6**$^+$]. The experimentally obtained values are presented at the bottom in [kcal/mol]. a) Difference comp. vs exp., b) relative error [%] with respect to exp. with colors ranging from red for large discrepancies to blue for small discrepancies

Taking the relative discrepancy, with respect to the experimentally obtained values, as the metric for accuracy, then the method M06L-D30 / def2-CBS // PBE-D3(BJ) / def-TZVP in (Table SI-3-1 and Table SI-3-2, entry 3) showed most accurate results in general.
The computational BDEs for [**1-3**$^+$] are substantially higher than the experimentally obtained values, as was originally observed for the Pd-Cu complex [**1**$^+$]. The computational BDEs for [**4-6**$^+$] not only show a generally matching trend of the computations with experiments but also with significantly less discrepancy. Such a trend has already been predicted computationally for closely related molecular systems.[30] A direct comparison is difficult as a slight perturbation of the trend was noticed and ascribed to the charged substituent (see subsection 3.1.4).[30]

Energy Decomposition Analysis

The interaction energy between the two fragment complexes [M] and [Pd] was further investigated by Energy decomposition analysis (EDA), using the Extended Transition State (ETS) method in conjunction with Natural Orbitals for Chemical Valence (NOCV), as implemented in ADF.[64–66] The analysis was performed on the ZORA-PBE-D3(BJ) / QZ4P // PBE-D3(BJ) / def2-TZVP level of theory (Table SI-3-1 and Table SI-3-2, entry 6). The total binding energy (E_{bind}) of the two specified fragments in this study can be decomposed into five distinct components as per formula (3); these components include:

$$E_{bind} = \underbrace{\overbrace{\Delta E_{pauli} + \Delta E_{elstat}}^{\Delta E_{steric}} + \Delta E_{orb}}_{\Delta E_{int}} + \Delta E_{disp} + \Delta E_{prep} \qquad (3)$$

ΔE_{pauli}: This represents a repulsive interaction, Pauli repulsion energy contribution.

ΔE_{elstat} (ΔV_{elstat}): This term accounts for the combination of attractive and repulsive electrostatic interactions.

ΔE_{orb}: Energy contribution due to orbital overlap. This component reflects the energy change when the fragment orbitals are allowed to interact and relax into combined molecular orbitals. It encompasses charge transfer and polarization effects.

ΔE_{disp}: This term describes the interaction due to dispersion forces.

ΔE_{prep}: This is the sum of the energy differences of the fragments in their complex geometry relative to when the fragments are separated and in relaxed geometries.

Additionally, within the same theoretical framework, certain terms are often summarized for convenience, such as ΔE_{steric} for steric contributions, or ΔE_{int} for internal energy. In Table SI-3-3 the energy contributions are listed for all investigated complexes.

Structure	[(bhq)$_2$Pd-**M**(IPr)]$^+$ 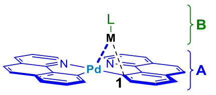			[(bhq)$_2$Pd-**Au**(PPh$_3$)]$^+$	[(bhq)(bhq^{CH2NMe3})Pd-Zn(**Ar**)$_2$]$^+$ 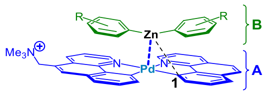		
	Cu [**1**$^+$]	Ag [**2**$^+$]	Au [**3**$^+$]	Au [**8**$^+$]	C_6F_5 [**4**$^+$]	Ar^{2,4,6F} [**5**$^+$]	Ar^{2,6F} [**6**$^+$]
-BDE^{comp}	-74.4	-65.2	-74.3	-61.9	-33.4	-29.9	-28.4
$\mathbf{\Delta E_{prep}}$	7.7	2.5	6.2	4.8	8.9	7.0	10.5
ΔE^{A}_{prep}	2.8	2.0	4.8	2.2	1.4	1.1	1.4
ΔE^{B}_{prep}	4.8	0.5	1.4	2.5	7.6	5.9	9.0
$\mathbf{\Delta E_{steric}}$	9.5	6.7	17.0	17.3	16.9	15.4	10.6
ΔE_{pauli}	97.5	84.2	126.6	108	62.5	55.5	55.5
ΔE_{elstat}	-88.0 [49]	-77.5 [51]	-109.7 [53]	-90.7 [52]	-45.7 [44]	-40.1 [43]	-45.0 [48]
$\mathbf{\Delta E_{orb}}$	-67.7 [38]	-52.3 [34]	-75.8 [37]	-68.2 [39]	-35.1 [33]	-29.6 [32]	-30.1 [32]
$\mathbf{\Delta E_{disp}}$	-23.8 [13]	-22.0 [15]	-21.7 [10]	-15.8 [9]	-24.1 [23]	-22.7 [25]	-19.4 [21]
ΔBDE	21.6	19.3	21.9	5.0	-2.6	-1.1	-2.5

Table SI-3-3: EDA analysis at ZORA-PBE-D3(BJ) / QZ4P // PBE-D3(BJ) / def2-TZVP level of theory (Table SI-3-1 and Table SI-3-2 entry 6) with energy contributions ΔE in [kcal/mol]. ΔBDE describes the discrepancy between computational and experimental BDEs for the specified method (including ZPE). BDE^{comp} denotes the computational BDE without ZPE .

In the triad [**1-3**$^+$], the ΔE_{prep} contribution is more pronounced for ions [**1**$^+$] and [**3**$^+$]. Reasoned by visual inspection of the dissociated structures, in [**1**$^+$] an interaction of the metal center with an *i*Pr moiety is visible, whereas in Au a more pronounced deformation of the [Pd(bhq)$_2$] moiety is required. As for the complexes [**4-6**$^+$], the preparation energy is of comparable magnitude. Deviations may be explained by structural deformations of Zn(Ar)$_2$. For ΔE_{steric} it becomes evident that the magnitude of this term is comparable across all structures relative to the larger differences in the contributions from orbital interactions ΔE_{orb}.This becomes especially prominent when [**3**$^+$], and [**8**$^+$], are compared, while the steric term is similar, the biggest differences are observed for dispersion and orbital contributions. Consequently the two contributions ΔE_{orb} and ΔE_{disp} were analysed more in-depth, which is shown in the next two subsections.

Decomposing Orbital Interaction

To scrutinize the orbital interaction in more detail, it was decomposed into contributions from the different NOCV contributions. [64–66] In ETS-NOCV, the fragment orbitals are linear combined to form a symmetry adapted basis in the irreducible representation ($\Delta E_{orb} = \Sigma_i \Delta E^i_{orb}$ where i is index for the NOCV's) of the molecular symmetry group. The deformation density is then decomposed into this basis. This commonly allows the interpretation of bonding interactions with respect to intuitively accessible terms such as σ donation or π-backdonation. [64–66] Due to the lack of symmetry in the complex structures discussed here, no assignments as π or σ orbital contributions were possible. Hence the here presented analysis relies on a combination of visual inspection of the NOCV interactions as well as the investigation into the charge transfer pathways, specifically emphasizing whether an orbital interaction is electron donating or accepting.[67] Table SI-3-4 presents the energy contributions for all the complexes under investigation. It includes the total charge transfer (Hirshfeld) and the charge transfer for the two major NOCV interactions. Additionally, the table provides insights into the partial charges for the two metal centers and C$_{ipso}$, utilizing both the Hirshfeld method and AIM charges. Moreover, the HOMO-LUMO gap is detailed for comprehensive analysis.

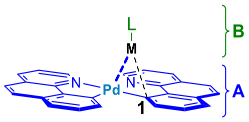

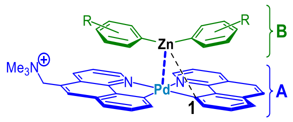

	[Pd(bhq)$_2$-**M**(IPr)]$^+$						[Pd(bhq)$_2$-**M**(PPh$_3$)]$^+$		[Pd(bhq)(bhq^{CH2NMe3})-Zn(**Ar**)$_2$]$^+$					
	Cu [**1**$^+$]		Ag [**2**$^+$]		Au [**3**$^+$]		Au [**8**$^+$]		C$_6$F$_5$ [**4**$^+$]		Ar^{2,4,6F} [**5**$^+$]		Ar^{2,6F} [**6**$^+$]	
$\mathbf{\Delta E_{orb}}$	-67.7	[38]	-52.3	[34]	-75.8	[37]	-68.2	[39]	-35.1	[33]	-29.6	[32]	-30.1	[32]
ΔE^1_{orb}	-29.3	(43)	-23.7	(45)	-40.7	(54)	-36.2	(53)	-16.9	(48)	-14.6	(49)	-15.5	(52)
ΔE^2_{orb}	-10.4	(15)	-7.1	(14)	-9.8	(13)	-7.2	(11)	-2.9	(8)	-2.2	(7)	-1.9	(6)
ΔE^{rest}_{orb}	-28.0	(41)	-21.5	(41)	-25.3	(33)	-24.8	(36)	-15.3	(44)	-12.8	(43)	-12.7	(42)
$\Sigma_i \Delta E^i_{orb}()$														
$\Delta\delta^i_A < 0$	-42.9	(63)	-35.9	(69)	-52.4	(69)	-50.4	(74)	-24.5	(70)	-19.4	(65)	-19.9	(66)
$\Delta\delta^i_A > 0$	-24.8	(37)	-16.1	(31)	-23.3	(31)	-17.6	(26)	-10.2	(29)	-9.8	(33)	-9.8	(33)
$\Delta\delta^{tot}_A$	-0.21		-0.23		-0.29		-0.36		-0.16		-0.13		-0.13	
$\Delta\delta^1_A$	-0.18		-0.21		-0.30		-0.31		-0.19		-0.16		-0.18	
$\Delta\delta^2_A$	0.03		0.02		0.06		-0.07		-0.02		0.02		0.02	
$\Delta\delta^{rest}_A$	-0.06		-0.04		-0.05		0.02		0.04		0.02		0.03	
δ(Pd) [a] | [b]	0.41	0.49	0.40	0.50	0.41	0.53	0.40	0.52	0.41	0.47	0.41	0.45	0.41	0.44
δ(M) [a] | [b]	0.21	0.47	0.24	0.37	0.19	0.17	0.15	0.02	0.34	0.90	0.33	0.87	0.33	0.89
δ(C) [a] | [b]	-0.14	-0.22	-0.13	-0.19	-0.13	-0.17	-0.13	-0.20	-0.12	-0.17	-0.11	-0.13	-0.11	-0.11
ΔE^{A-B}	-2.07		-1.88		-2.53		-2.68		3.64		4.30		4.32	
ΔE^{B-A}	6.17		6.13		6.18		6.90		1.08		0.98		0.66	

Table SI-3-4: Computational results of the ETS-NOCV calculations at the ZORA-PBE-D3(BJ) / QZ4P // PBE-D3(BJ) / def2-TZVP level of theory theory (Table SI-3-1 and Table SI-3-2 entry 6). (1st block) Energy decomposition into major 2 orbital interaction contributions or alternatively into sum of orbital contributions with charge donating or accepting characteristics respectively. Energies in [kcal/mol]. (2nd block) Charge transfer Hirshfeld assigned to major 2 orbital interactions, Δδ in [a.u.]. (3rd block) Partial charges on selected atoms according to Hirshfeld[a] and AIM[b] methodologies. (4th block) HOMO-LUMO gap , $\boldsymbol{\Delta E}$ in [eV].

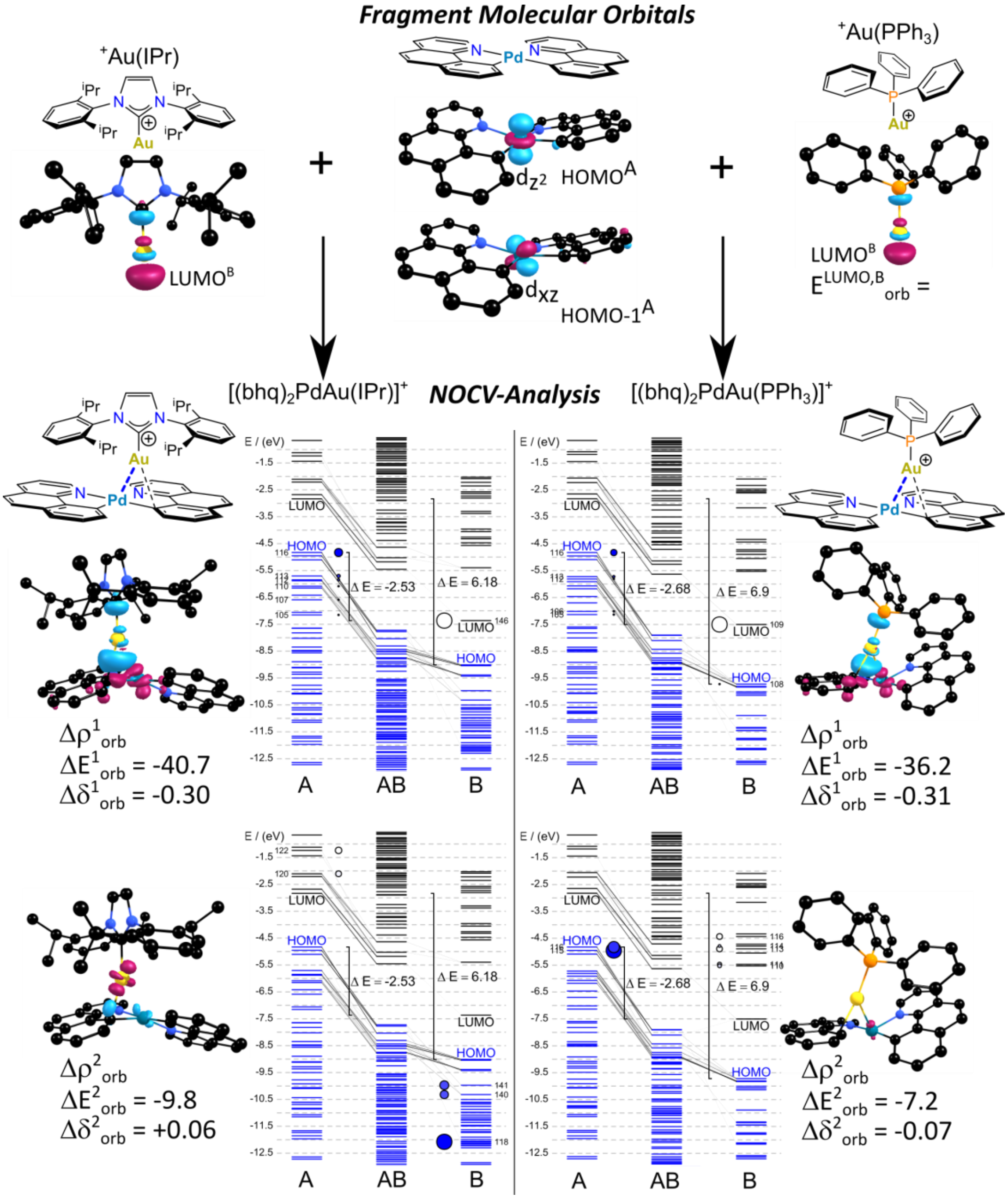

Figure SI-3-4: Schematic representation of primary and secondary orbital interactions in ions [**3**+] and [**8**+], at the ZORA-PBE-D3(BJ) / QZ4P // PBE-D3(BJ) / def2-TZVP level of theory theory (Table SI-3-1 and Table SI-3-2 entry 6). (top) Molecular orbitals of the separate complexes A and B. (bottom- sides) NOCVs of major two orbital interactions and their corresponding energy contributions as well as the charge transfer. (bottom, middle) MO diagram, the lines are showing the participation of the fragment orbitals in the construction of the molecular orbital.The circles are proportional to the contributions to the NOCVs.

Fragment Separated Dispersion Correction Contributions

In the energy decomposition analysis (see Table SI-3-4), it was shown that the dispersion interaction accounts for roughly 9-15 % of the attractive interactions in systems [**1-3**+],[**8**+] and approximately 21-25 % for complexes [**4-6**+]. In terms of the absolute magnitude, the dispersion correction terms were found to be relatively similar.

The dispersion correction obtained from the EDA analysis is basically the D3 correction term as PBE itself incorporates very little dispersion correction at middle to long range.[68] The D3 correction is based on an underlying additivity scheme where the dispersion correction is a sum of mainly pairwise interactions.[43] This additivity permits a more detailed dissection of the dispersion correction, where dispersion corrections between selected groups of atoms can be studied separately. $\Delta E_{disp} = \Sigma_{\mathrm{i}} \Delta E^{i}_{disp}$ where i is index for fragment combinations

This functionality is built into Grimme's program DFTD3 (standalone, version DFTD3 V3.1 Rev 1).[43,44] and was used as follows.[d] The program requires specification of the structure, the functional (here PBE) and a list of fragments for which the inter-fragment D3 correction are then to be calculated. The here studied structures were initially dissected into 4 fragments: the Pd atom, the M(d^{10}) metal atom, the ligands on the Pd metal center (depicted as A), and the ligands on the M(d^{10}) metal center (depicted as B). Intramolecular (or here intra-fragment) dispersion terms Pd-A, M-B, A-A, B-B, are largely cancelled out when calculating bond dissociation energies and are therefore neglected.

By far the biggest contribution to the dispersion correction is coming from the ligand-ligand interaction A-B. Although the dispersion correction term for the Pd-M interaction is minor in the shown decomposition scheme (Table SI-3-5), in a per-interaction manner the contribution is by far the biggest. The dispersion correction terms can be further decomposed into sub-fragments as described in Table SI-3-5. This allows to investigate the different interaction patterns in detail.

Structural Analysis

In order to analyse and compare the different structures, a set of distance measures is used. In the following subsection such metrices (see Figure SI-3-5) are defined, the corresponding results are shown in Table SI-3-5. The following analysis was conducted on xyz files (obtained from the DFT calculations as described above, PBE-D3(bj) / Def2-TZVP) using the program R. The R-script is provided in subsection 4.5.8.

$$i \in [X] \quad j \in [Y] \qquad (4)$$

$$d_{ij} = \left\| \overrightarrow{d_{ij}}^{XY} \right\| = \sqrt{\left(\vec{r}_j^{\,Y} - \vec{r}_i^{\,X}\right)^2} \qquad (5)$$

Equations (4) and (5): Indexes *i* and *j* represent atoms located in fragments *X* and *Y*, respectively. The vector $\vec{r}_i^{\,X}$ ($\vec{r}_j^{\,Y}$) denotes the coordinates of atom *i (j)* in fragment *X (Y)*, and d_{ij} is the distance (*Å)* between atoms *i* and *j* located in fragments *X* and *Y*, respectively.

<u>*Averaged inter-fragment distances:*</u> The average of pairwise inter-fragment distances (*Å)* can be calculated using the following equation (6).

$$\bar{d}^{XY} = \frac{1}{N_X N_Y} \sum_{i_X} \sum_{j_Y} d_{ij} \qquad (6)$$

Equation (6): The quantity $\bar{d}^{XY}$ represents the average inter-fragment distance (*Å)* between fragments *X* and *Y*. Here, N_X (N_Y) is the number of atoms in fragment *X (Y).*

<u>*Weighted inter-fragment distances:*</u> The weighted inter-fragment distance (*Å)* is calculated using Formula (8), with the weighting factor determined by Formula (7). This approach gives greater emphasis to smaller inter-fragment distances, similar to the D3 dispersion correction method, although all atoms are treated equally in this calculation.

$$w_{ij}^{XY} = \frac{d_{ij}^{-6}}{\sum_{i_X} \sum_{j_Y} d_{ij}^{-6}} \qquad (7)$$

$$\bar{d}_w^{XY} = \sum_{i_X} \sum_{j_Y} w_{ij}^{XY} d_{ij} \qquad (8)$$

Equations (7)** and **(8): d_{ij} represents the distance between atom *i* in fragment *X* and atom *j* in fragment *Y*. w_{ij}^{XY} is the corresponding weighting factor. The weighted inter-fragment distance (*Å)* between fragments *X* and *Y* is denoted by $\bar{d}_w^{XY}$.

<u>*Centroid-centroid distances:*</u> Another metric is the centroid-centroid distance between fragments (see Figure SI-3-5). In equation (10) a definition is provided.

$$\vec{c}^{\,X} = \frac{\sum_i^{N_X} \vec{r}_i^{\,X}}{N_X} \qquad (9)$$

$$d_c^{XY} = \left\| \vec{d}_c^{\,XY} \right\| = \sqrt{\left(\left(\vec{c}^{\,Y} - \vec{c}^{\,X}\right)\right)^2} \qquad (10)$$

Equations (9)** and **(10): $\vec{c}^{\,X}$ ($\vec{c}^{\,Y}$) denotes the vector to the centroid of fragment *X (Y)*. d_c^{XY} represents the distance (*Å)* between the centroids of fragments *X* and *Y*.

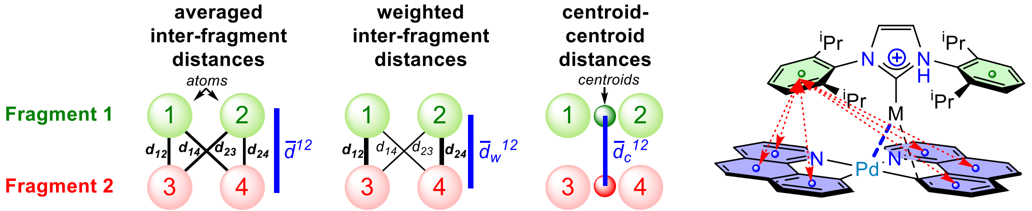

Figure SI-3-5: (left) Schematic representations of the different metrices used to analyse structural characteristics. (right) Model complex with selected centroid-centroid distances highlighted.

[d] D4 does not provide this functionality at the time.

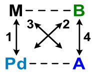	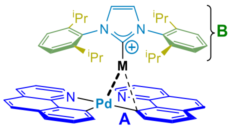 $[Pd(bhq)_2\text{-}\mathbf{M}(IPr)]^+$			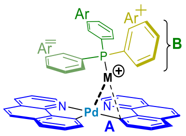 $[Pd(bhq)_2\text{-}\mathbf{M}(PPh_3)]^+$	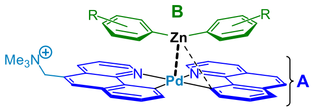 $[Pd(bhq)(bhq^{CH2NMe3})\text{-}Zn(\mathbf{Ar})_2]^+$		
	Cu	Ag	Au	Au	C_6F_5	$Ar^{2,4,6F}$	$Ar^{2,6F}$
$\Delta\mathbf{E}_{disp}$	-23.8 [13]	-22.0 [15]	-21.7 [10]	-15.8 [9]	-24.1 [23]	-22.7 [25]	-19.4 [21]
$\Delta E_{disp}^{Pd,M}$	-0.7	-0.8	-0.9	-0.9	-0.7	-0.7	-0.7
$\Delta E_{disp}^{Pd,B}$	-2.1[e]	-1.8[e]	-1.8[e]	-1.03[e]	-2.1	-1.9	-1.9
$\Delta E_{disp}^{M,A}$	-4.3	-5.0	-5.2	-5.7	-3.6[f]	-3.5[f]	-3.8[f]
$\Delta E_{disp}^{A,B}$	-17.3	-15.0	-14.4	-8.9[g]	-17.9	-16.6	-13.1
	A -Ar			A -Ar$^=$	(bhq$_2$)*-B		
	-6.4	-5.6	-5.5	-4.4	-16.1	-14.0	-10.0
$\Delta E_{disp}^{A*,B*}$	A -^{i}Pr			A -Ar$^+$	CT-B		
	-8.9	-7.9	-7.4	-2.72	-1.8	-2.6	-3.2
	A -NHC			A -Ar^{remote}			
	-2.1	-1.5	-1.5	-0.66			
ΔE_{disp}^{tot}	-24.4	-22.7	-22.3	-16.5	-24.2	-22.7	-19.5
$d^{Pd,M}$	2.55	2.72	2.71	2.71	2.68	2.69	2.70
$\bar{d}^{Pd,B}$	6.18	6.35	6.31	6.74	5.16	5.24	5.32
$\bar{d}^{M,A}$	5.12	5.20	5.28	4.98	5.93	5.89	5.52
$\bar{d}^{A,B}$	7.62	7.74	7.84	7.74	7.12	7.16	6.89
$\bar{d}_w^{Pd,B}$	3.90	4.27	4.35	5.02	4.19	4.25	4.18
$\bar{d}_w^{M,A}$	2.61	2.95	2.87	3.03	3.71	3.84	3.79
$\bar{d}_w^{A,B}$	4.04	4.17	4.17	4.36	3.74	3.84	3.85
$min(d_c^{A',B'})$	3.94	3.94	3.95	4.10	3.60	3.65	4.34
$min(d^{A,B})$	2.49	2.58	2.54	2.87[Y]	2.27[X]	2.42	2.35[Z]

Table SI-3-5: Decomposition scheme for the dispersion correction. All energy values in [kcal/mol], distances d^{XY}, $\bar{d}^{XY}$, $\bar{d}_w^{XY}$ and d_c^{XY} in [Å]. The relative contribution to attractive interactions to the total binding energy are given in square brackets. (top) Structures and the definitions of the fragments Pd, M, A, B, and subdivisions of these fragments, as for the IPr ligands B is further decomposed into Ar, ^{i}Pr and NHC, for Pd^{CT} further decomposing into bhq$_2$* and CT. Superscripts describe the following: x)H-H(CT) interaction, y) H(Ph)-N(bhq) interaction and z) F-H(CT) interaction.

Non covalent interactions

In order to visualize non-covalent interactions, an IGMH analysis was performed. IGMH refers to "Independent gradient model based on Hirshfeld partition" and is similar to NCI.[69] In Figure SI-3-6 an isosurface of δg^{inter} is shown, with the coloring Sign(λ_2)ρ, which is the product of the electron density and the second largest eigenvalue of the hessian matrix.[69] Weak non-covalent interactions such as van der Waals interaction are represented as green. Although IGMH and the shown dissection of dispersion interaction are conceptually different, the representation by IGMH helps visualizing the different sub-fragment interactions.

---

[e] not further decomposed, predominantly composed of the Pd-*i*Pr interaction.

[f] not further decomposed into M-bhq$_2$* and M-CT, the dispersion contribution originating form the later is insignificant, hence $\Delta E_{disp}^{M-A}(Zn-A) \approx \Delta E_{disp}^{M-A}(Zn-(\mathrm{bhq})_2{}^*)$.

[g] The energy contribution also includes the interaction between the A-P which accounts for -1.1 kcal/mol, which is not listed.

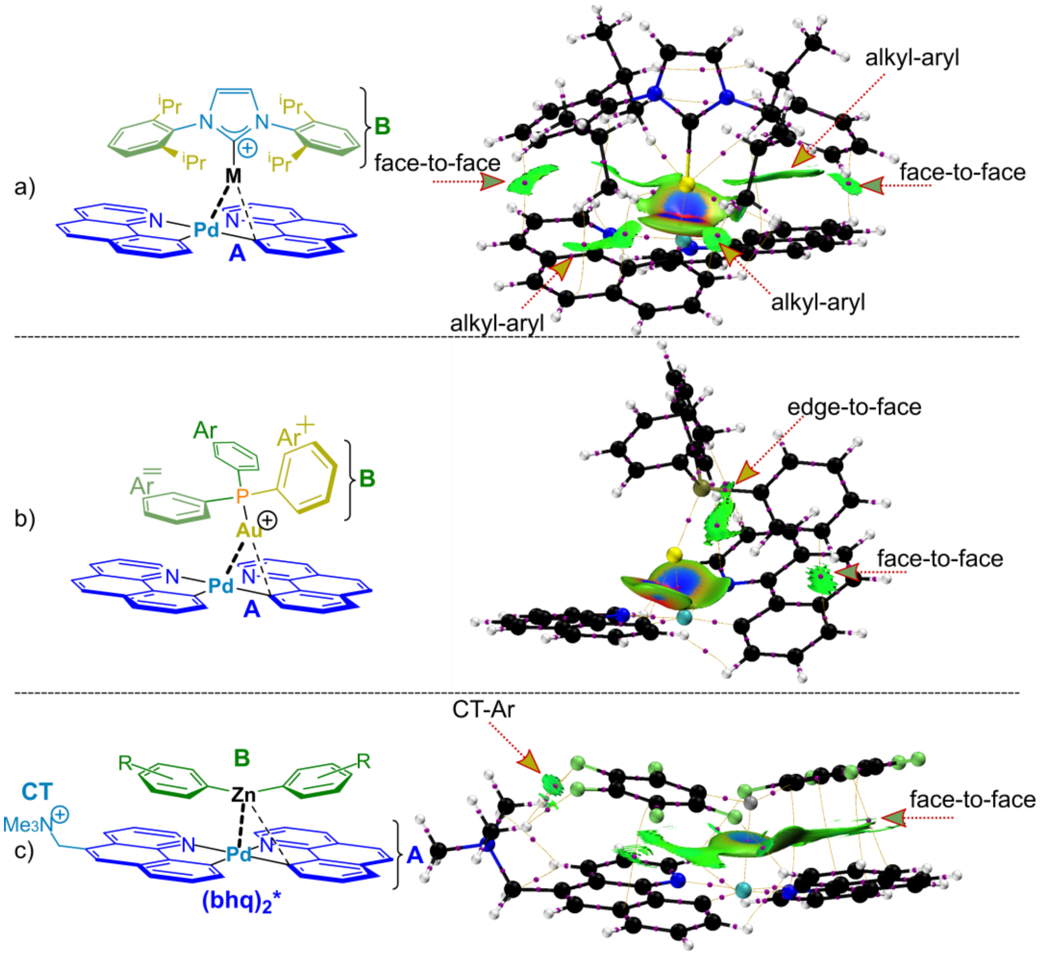

Figure SI-3-6: IGMH analysis[69] performed using Multiwfn Version 3.8(dev)[63], densities obtained at the DKH2-M06-D30 / DKH-def2-TZVP // PBE-D3(BJ) / def2-TZVP level of theory (Table SI-3-1 and Table SI-3-2, entry 10). *Sign(λ₂)ρ* colored isosurfaces of δg^{inter} = 0.0025 a.u. corresponding to IGMH analyses for shown ions a) [**3**⁺], b) [**8**⁺], c) [**4**⁺]. Specified Fragments A and B shown in structures (left). The coloring method of *sign(λ₂)ρ* is as follows: blue) prominent attractive weak interaction, green) van der waals interaction, red) prominent repulsive interaction (steric effect in ring etc.). Different interactions are marked with arrows. Additionally the bond critical points, according to AIM theory,[61] and the bond-paths are shown.

3.1.4. The Effect of the Charge

To compare experimentally obtained BDEs with reported values, which were calculated for charge-neutral systems, an investigation into potential effects of the charge-tag was necessary.[30] The comparison of the computational BDEs of the charged heterobimetallic complexes [**4-6**⁺] with their uncharged analogues, revealed a different trend with respect to the electronic modifications on the aryl moiety. As more electron rich aryl rings were present, a rotation of one of the aryls was observed.

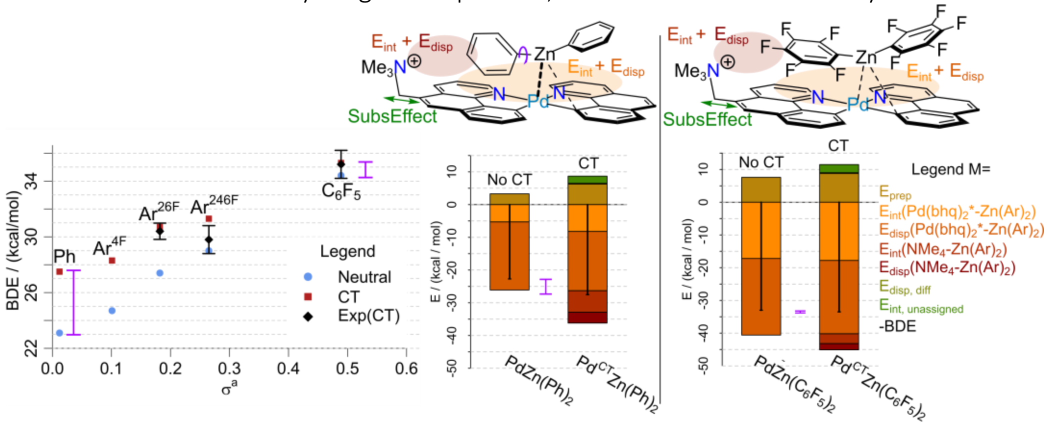

Figure SI-3-7: (left) Correlation of comp. BDEs with corresponding Hammet constants obtained from a Web Tool,[70] for both neutral and charged Pd-Zn heterobimetallic complexes (M06-L-D30/def2-CBS // PBE-D3BJ / Def2-TZVP (ZPE)). Experimentally obtained values are described. (right) Energy decomposition to highlight charge effects. The methodology is further described in the text.

As a consequence of introducing a charge-tag, three potential effects were formulated (depicted as ovals in Figure SI-3-7):

1) an electronic substituent effect on the overall BDE,
2) the establishment of electrostatic interactions between the charged moiety and the aryl groups,
3) accompanied by structural changes with consequental effects on the electronic structure, including dispersion interactions.

In a case study, the binding energy between a tetramethylammonium ion (NMe_4^+) and an organozinc compound $Zn(Ar)_2$ in a structural configuration equivalent to the heterobimetallic complex NMe_4^+-$Zn(Ar)_2$ with Ar = Ph, C_6F_5 , was calculated. The structures were derived from the corresponding heterobimetallic complexes by replacing the $[Pd(bhq)_2]$ for an H to form NMe_4^+.

The obtained binding energies for these control structures were found to be significantly different, when Ph and C_6F_5 were compared, and could serve as an explanation for the observed charge-tag effect. For both the heterobimetallic systems $[(bhq)(bhq^{5\text{-}CH2NMe3})Pd\text{-}Zn(Ar)_2]^+$ with Ar = Ph, C_6F_5, and the corresponding NMe_4^+- $Zn(Ar)_2$ structures, binding energies were computed. To isolate the residual changes introduced to the complex upon the introduction of the charge-tag, the binding energies were subtracted from each other.

In both cases, a small residual energy contribution was obtained, for Ar = Ph 2.2 kcal/mol and for Ar = C_6F_5 2.5 kcal/mol. The ammonium moiety is not directly bound to the bhq, but via a methylene bridge, which means some residual electron withdrawing capabilities can be assumed. Unfortunately no Hammett constants were found for $-CH_2NMe_3^+$, the closest resembling substituent for which such constants were available was $-CH_2NH_3^+$. They were reported to be σ^m / σ^p = 0.59 / 0.53 showing electron withdrawing capabilities comparable to $-CF_3$ σ^m/σ^p = 0.43/0.54 or NO_2 σ^m/σ^p = 0.71/0.78.[71] On the other hand the differences in BDE for $[(bhq^{7\text{-}R})_2Pd\text{-}(Zn(C_6F_5)_2]^+$ with R=CF_3, NO_2 relative to R = H were reported to be 1.5 and 3.7 kcal/mol respectively.[30] By this analysis, the mentioned residual energy term is in the energetic range of electronically comparable (by means of Hammet constants) substituents.

3.2. Investigations Into Possible Transformations prior Dissociation

As shown in a previous section (see section 2.4) the ESI-MS/MS CID spectra for ions [**1-3**$^+$] show additional fragmentation products (see Figure SI-2-2). The “cleanest” dissociation was observed for cation [**2**$^+$] among the triad.

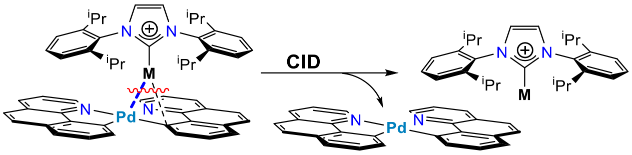

Scheme SI-3-1: Expected dissociation for ions [**1-3**$^+$], resulting in a charge neutral $[Pd(bhq)_2]$ complex and a charged $[(IPr)M]^+$ ion complex.

In Figure SI-3-8, an ESI-MS/MS CID spectrum for ion [**1**$^+$] is shown, exemplifying a more detailed view on the fragmentation products. The isotopic pattern for the observed fragments were simulated for specific compositional differences, e.g. relative to the daughter ion $[(IPr)Cu]^+$, and led to the proposition that fragmentations may occur that ultimately result in methane formation and the cleavage of CuH or CuMe respectively.

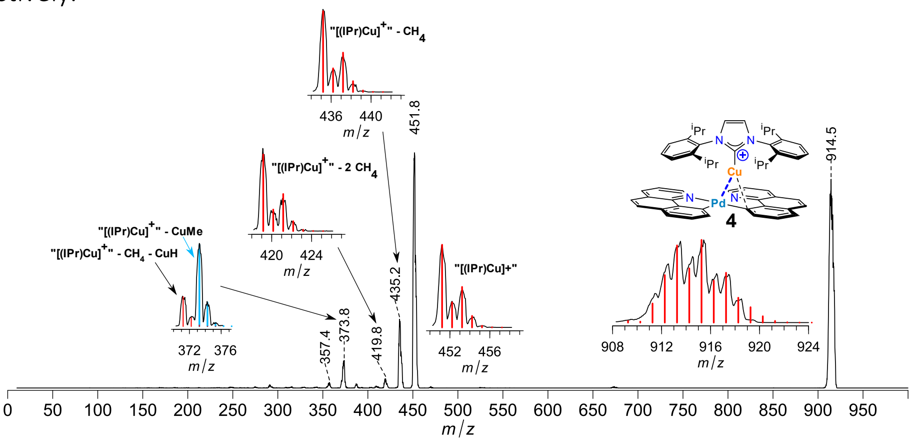

Figure SI-3-8: ESI-MS/MS CID spectrum of complex ion [**1**$^+$] with approx. 0.4 mTorr of Ar collision gas in the collision cell at collision offset (*-COFF*) 60 V. Proposed structure for the parent ion is shown. Inset: experimental (black, CID-mode) and

simulated (red) isotopic pattern. Marked numbers refer to m/z of the peak maxima, only peaks with >2% of relative intensities are labelled. The set conditions are "harsher" then the once in a common T-CID experiment.

The obtained rather high experimental BDEs ("loose" dissociation mechanism), the appearance of other fragments when harsher CID conditions are chosen and the observed discrepancies between experimental and computational BDEs, motivated a more in-depth analysis of the dissociation processes.

The main focus of this section is to legitimize the interpretations of the experimentally observed dissociation events. The primary focus is to determine whether the additional fragmentation products arise from sequential transformations occurring after dissociation or from transformations that precede the dissociation event.

Particularly important are potential transformations prior to the dissociation, as they could potentially affect the gas-phase kinetics and hence the interpretation thereof. To investigate potential alternative dissociation pathways, auxiliary data were obtained computationally. The additional fragmentation products observed experimentally serve a dual purpose – guiding the delineation of potential transformations and providing an experimental fingerprint for the dissociation events.

Computational : A schematic representation of a set of relevant transformations is shown in Figure SI-3-9, the corresponding computational results are shown in Figure SI-3-10 (for more detailed information and all calculated transformations see Figure SI-3-11, Figure SI-3-12, Figure SI-3-13, Figure SI-3-14). All structures with the same base label (**A**,**B**,**C**) have the same m/z ratio and the super-scripts are descriptive of the transformations that led to the specific structure.
The investigations were based on the structures for [**1-3**]$^+$ obtained from the conformational search (see subsection 3.1.1). Two potentially relevant types of reactivities were investigated; the reductive elimination of the bhq$_2$ on the [Pd] moiety and the oxidative addition of the M(I)(d^{10}) metal center into either a C-H or a C-C bond located on the spatially close ^{i}Pr groups.

The former process, the reductive elimination to form bhq$_2$ together with a subsequent dissociation, was found to be unfeasible as the asymptote dissociation energy was found to be significantly higher (see Figure SI-3-11 and Figure SI-3-12) than the corresponding BDEs for the expected Pd-M cleavage. Hence, this process is not discussed here explicitly. However, the transformations proceeding via an oxidative addition activation step did reveal some potentially relevant dissociation mechanisms. Reminiscent oxidative addition processes have been reported for the C-H activation of an ^{i}Pr group with an Ir(I) metal center.[72,73] While the transition states for these oxidative addition activation steps were not obtained, subsequent intermediates with proposed M(III) metal centers coordinated to Pd(bhq)$_2$ were identified. The energetics of these intermediates are presumed to establish a lower limit for the activation step's energy.

A specific set of consecutive reaction steps, encompassing reductive eliminations, the generation of noncovalently bound adducts, and dissociations, were computationally examined. The reductive elimination of NHC ligands with Me has been documented for Pd(II).[74] In the subsequent section, only pathways with dissociation asymptotes lower or marginally higher than the initially conceived dissociation processes are addressed.

Optimizations : The calculations were performed using ORCA 5.0.2. Geometry optimizations were performed using the PBE functional, together with the def2-TZVP[34] basis set; for Pd,Ag and Au Def2-ECP[35–42] was used. Grimme's D3 dispersion correction was used together with the Becke-Johnson damping.[43,44] For all calculations defgrid2 DFT integration grid was used. SCF convergency criteria were set to TightSCF and optimization convergency criteria were set to TightOpt (TolE = 1e-6, TolRMSG = 3e-5, TolMaxG = 1e-4, TolRMSD = 6e-4, TolMaxD = 1e-3). For all calculations, Coulomb fitting was employed with general Weigend J auxiliary basis set.[45] In order to validate the ground state nature of the structure, and also to obtain zero-point energies, frequency calculations were performed on the same level of theory as the optimization (PBE-D3(BJ)/Def2-TZVP) .

Discussion: No intermediates were found that were more stable than the starting structures **A**. Most transformations appear to have energetically demanding OA activation steps (**ii**$^{a\text{-}c}$) when compared to

the commonly assumed energy transfer in the preparation phase of roughly 20-25 kcal/mol, hence no preparative rearrangements were expected.[h]

The energetically lowest lying dissociation asymptotes were found for the II^{b} dissociations. These dissociation pathways do not explain the observation of the main daughter peak but may be the origin of some observed additional fragmentation products (e.g. for $\mathbf{II}^{abb}$ and $\mathbf{II}^{bbb}$: 387.28 m/ z and $\mathbf{II}^{cbb}$: 373.26 m/ z). Taking gas-phase rate kinetics into account the preceding activation steps may render these dissociation processes to be “tight” and hence to be less pronounced when compared to pathway I dissociations. Due to the unknown energetics of the transition states a rationalization of the differences in fragmentation patterns is not permitted.

One potentially relevant dissociation mechanism was observed, $\mathbf{A}$-$\mathbf{ii}^{c}$-$\mathbf{A}^{c}$-$\mathbf{ii}^{ca}$-$\mathbf{A}^{ca}$—>$\mathbf{II}^{ca}$, which would mimic the experimental observations. For Cu and Ag this process is unlikely because the initial activation step would lead to diminished dissociation observation compared to the original dissociation I (as mentioned above). Also for Au this is unlikely, but when a very low transition state, energetically barely above $\mathbf{A}^{c}$ would be assumed, then this dissociation process could be modelled as a loose dissociation process. However a discrepancy would remain as the final dissociation asymptote is still roughly 10 kcal/mol above the experimentally measured value assuming “loose” dissociation.

Another explanation for the additional fragmentation products, and frankly more intuitive, is the sequential rearrangement of free $[(IPr)M]^{+}$ ions by additional collision events, which one might expect because, after the dissociation, the metals are coordinatively unsaturated, which opens low energy pathways, e.g. fragmentations. This is supported by the observation that the same fragmentation patterns are observed when performing CID analysis on the ions $[(IPr)M(NCMe)]^{+}$ separately.

In conclusion, it is assumed that the main daughter peak originates from the expected dissociation process I, and cannot be explained by other mechanisms. The fragmentation products are either a consequence of undetectable competitive transformations prior dissociation or a result from secondary fragmentations after the dissociation. Effects from the latter, can be suppressed by cutting at certain collision offsets where no additional fragmentation is observed.

[h] Preparative, meaning transformations that may happen during sample preparation, electrospray or thermalization.

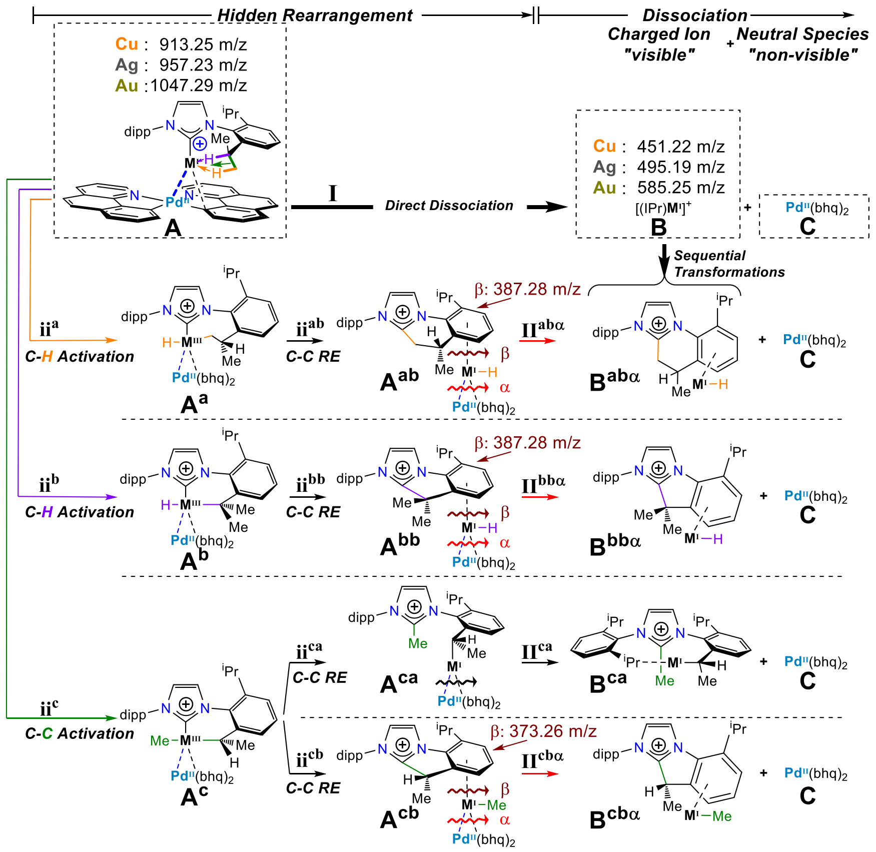

Figure SI-3-9: Schematic representation of potential dissociation processes occurring in the ESI-MS/MS CID experiment when measuring complex ions of general formula $[(bhq)_2Pd\text{-}M(IPr)]^+$. The labelling of the structures is defined locally and is only used in this sub-chapter, structures with the same base-label (A,B,C) are having the same m/z ratio and are indifferentiable in common MS. Dissociations are marked with roman letters I/II. The abbreviation, dipp, denotes o,o'-diisopropylphenyl.

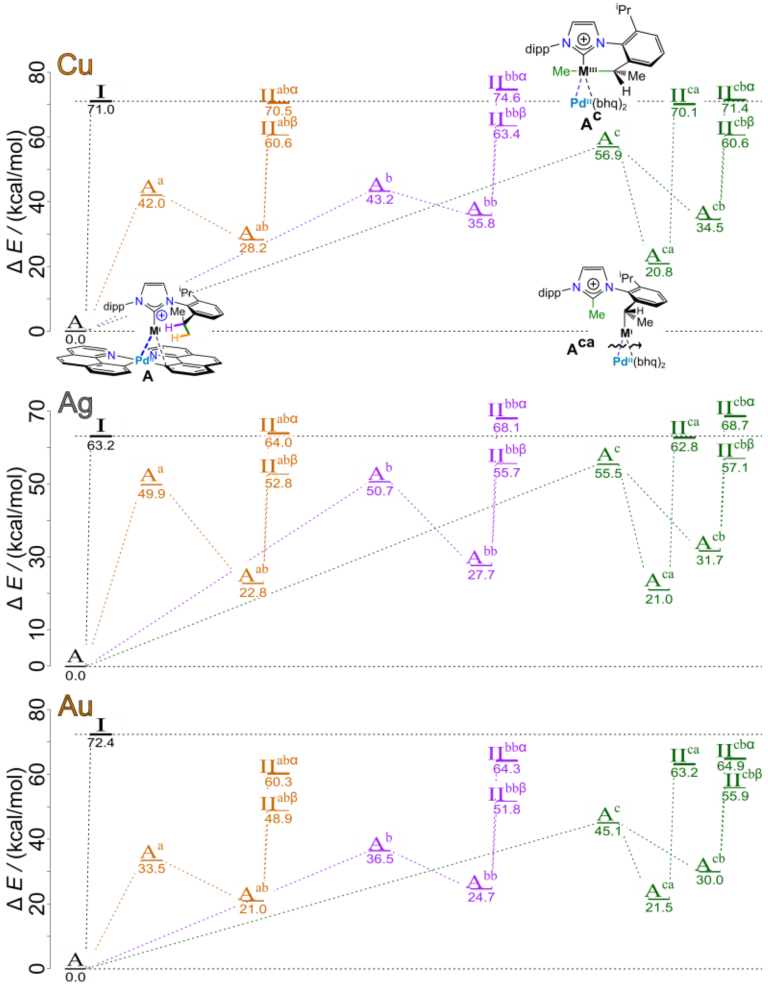

Figure SI-3-10: PES for the rearrangements shown in Figure SI-3-9. Energies obtained and corrected for zero-point energy at the PBE-D3bj/def2-TZVP level of theory.

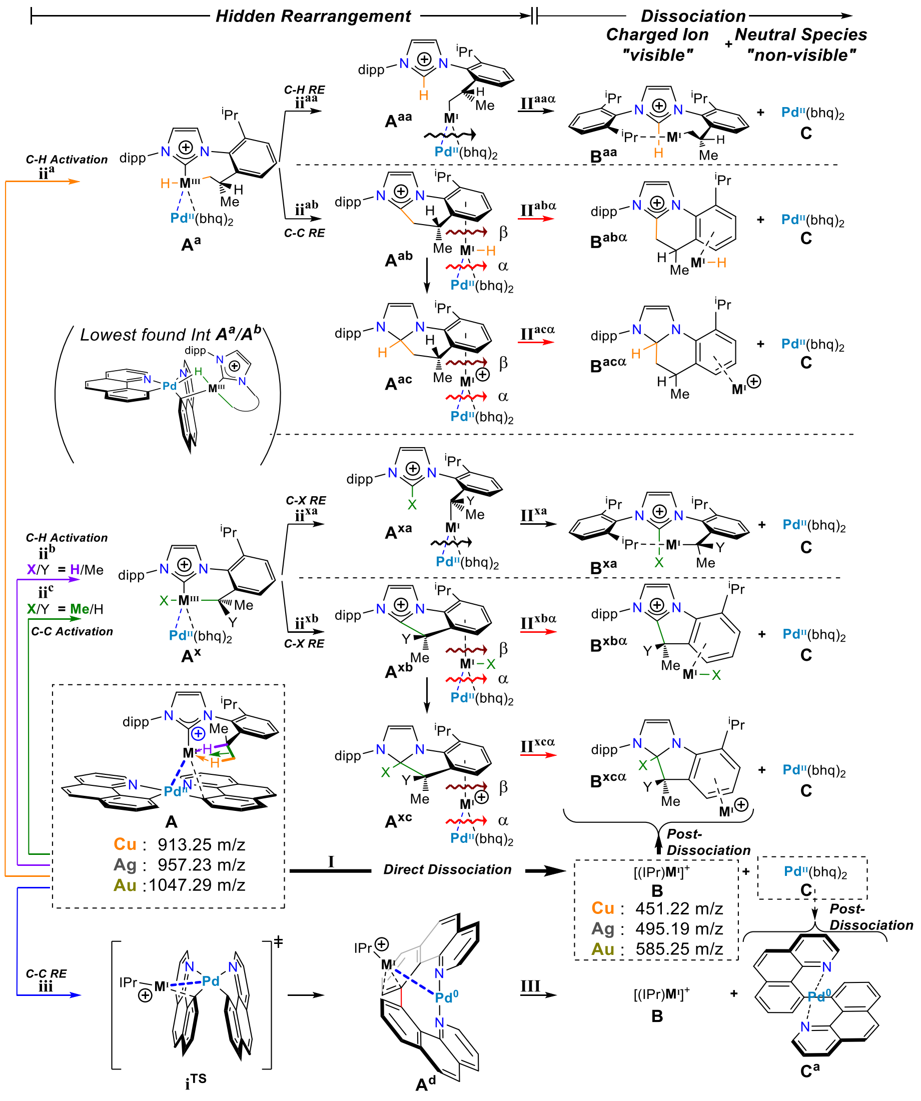

Figure SI-3-11: Schematic representation of all considered dissociation pathways.

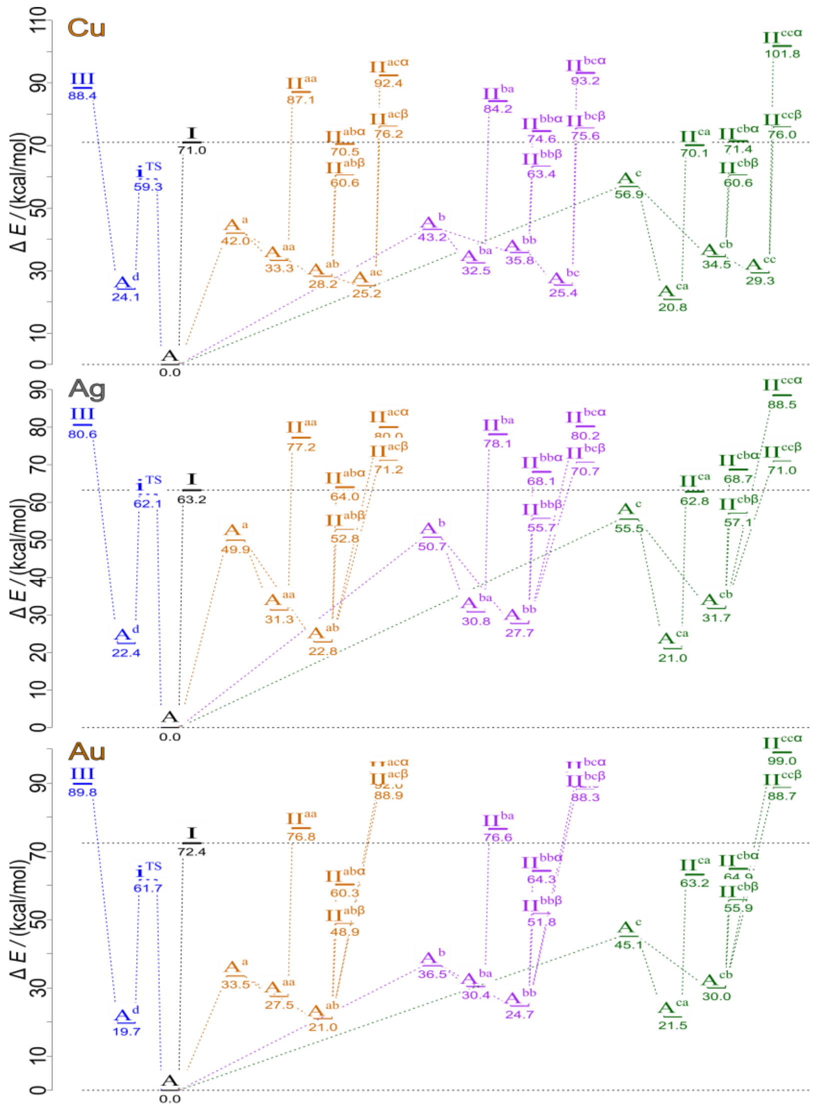

Figure SI-3-12:: Potential Energy Surfaces for the rearrangements shown in Figure SI-3-11.

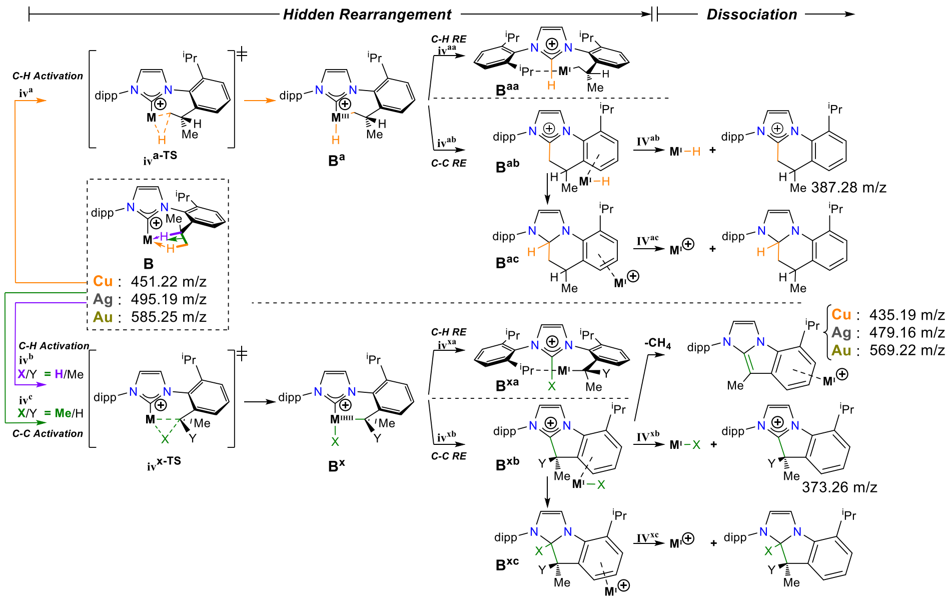

Figure SI-3-13: Schematic representation of all considered dissociation pathways.

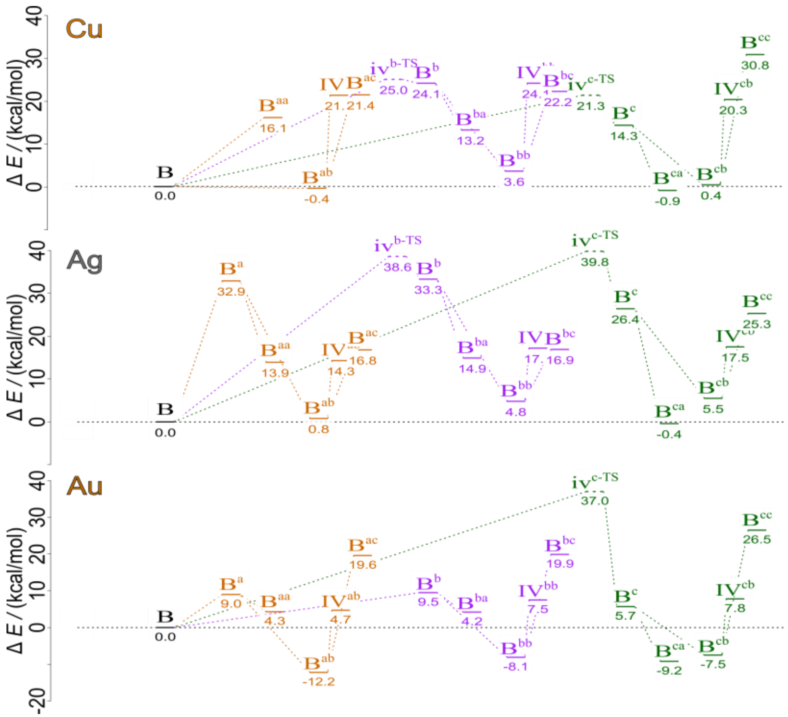

Figure SI-3-14: Potential Energy Surfaces for the rearrangements shown in Figure SI-3-13.

4. Additional Experimental Spectra and Tables

4.1. NMR-Spectra of Products Towards Charged Pd-Complex Derivative

4.1.1. NMR Spectra of bhq$^{5\text{-}Br}$H,7.1

For the synthetic procedures see subsection 2.1.1.

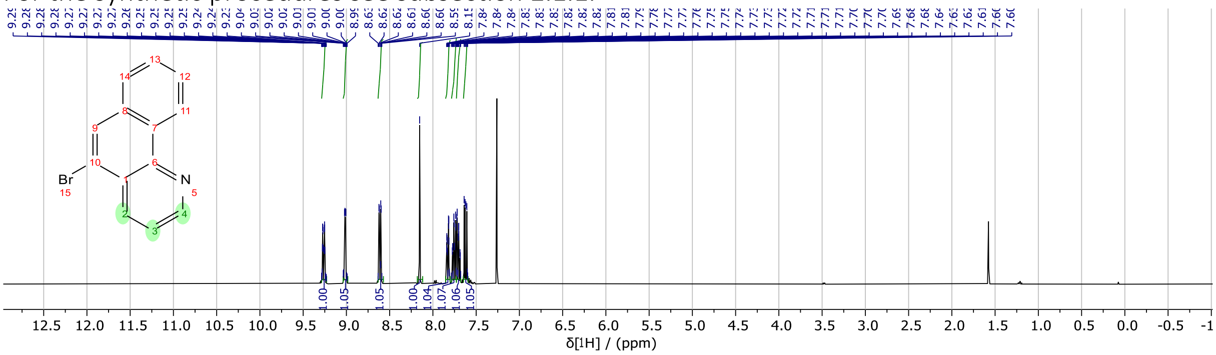

Figure SI-4-1: ^{1}H-NMR (400 MHz) spectrum of compound bhq$^{5\text{-}Br}$H, **7.1**, in $CDCl_3$ measured at r.t. The spectrum was referenced to the residual solvent peak: ^{1}H-NMR ($CDCl_3$) δ = 7.26 ppm.

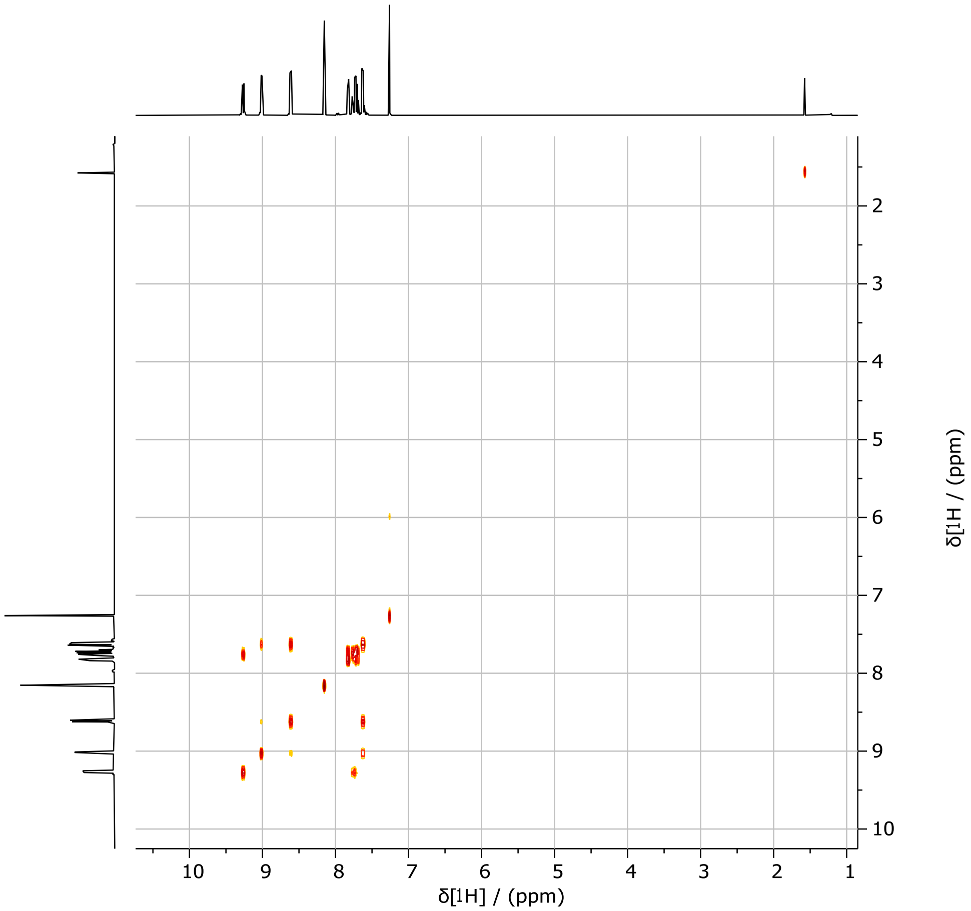

Figure SI-4-2: ^{1}H^{1}H COSY-NMR (400 MHz, 400 MHz) of compound bhq$^{5\text{-}Br}$H, **7.1**, in $CDCl_3$ measured at r.t.

4.1.2. NMR Spectra of bhq$^{5\text{-CHO}}$H, 7.2

For the synthetic procedures see subsection 2.1.2.

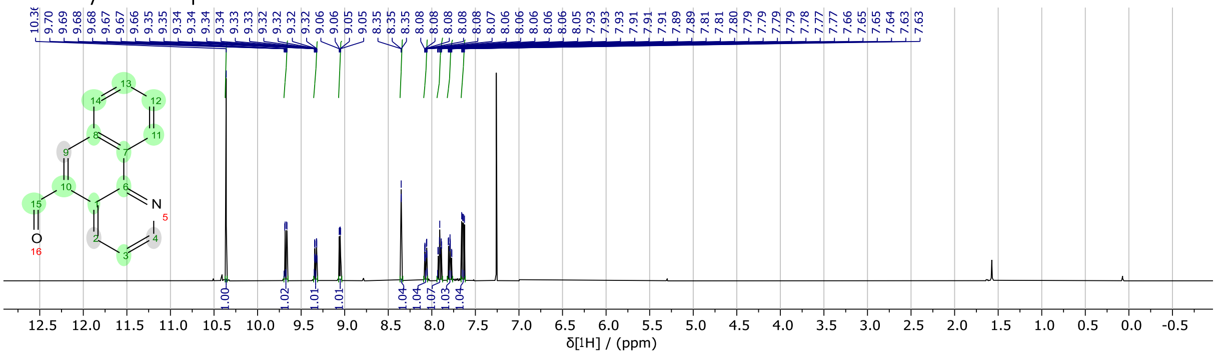

Figure SI-4-3: ^{1}H-NMR (400 MHz) spectrum of compound bhq$^{5\text{-CHO}}$H, **7.2** , in $CDCl_3$ measured at r.t. The spectrum was referenced to the residual solvent peak: ^{1}H-NMR ($CDCl_3$) δ = 7.26 ppm.

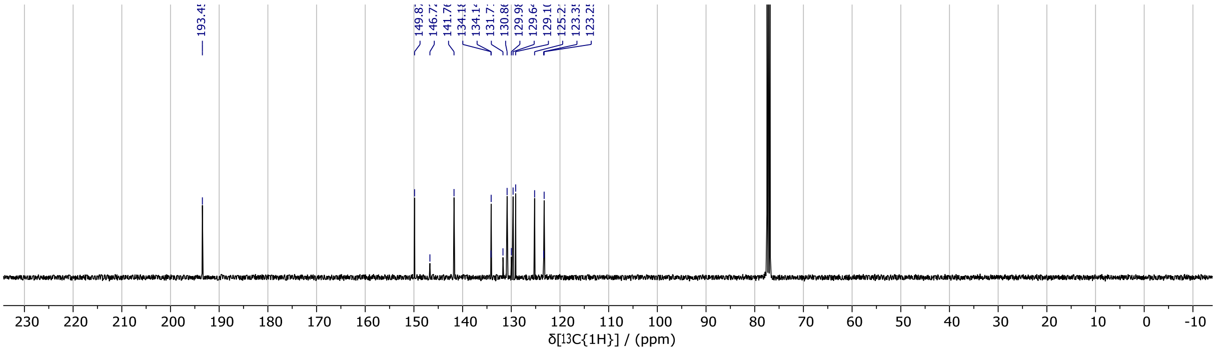

Figure SI-4-4: ^{13}C{^{1}H}-NMR (101 MHz) spectrum of compound bhq$^{5\text{-CHO}}$H, **7.2**, in $CDCl_3$ measured at r.t. The spectrum was referenced to the residual solvent peak: ^{13}C -NMR (**$CDCl_3$**) δ = 77.16 ppm.

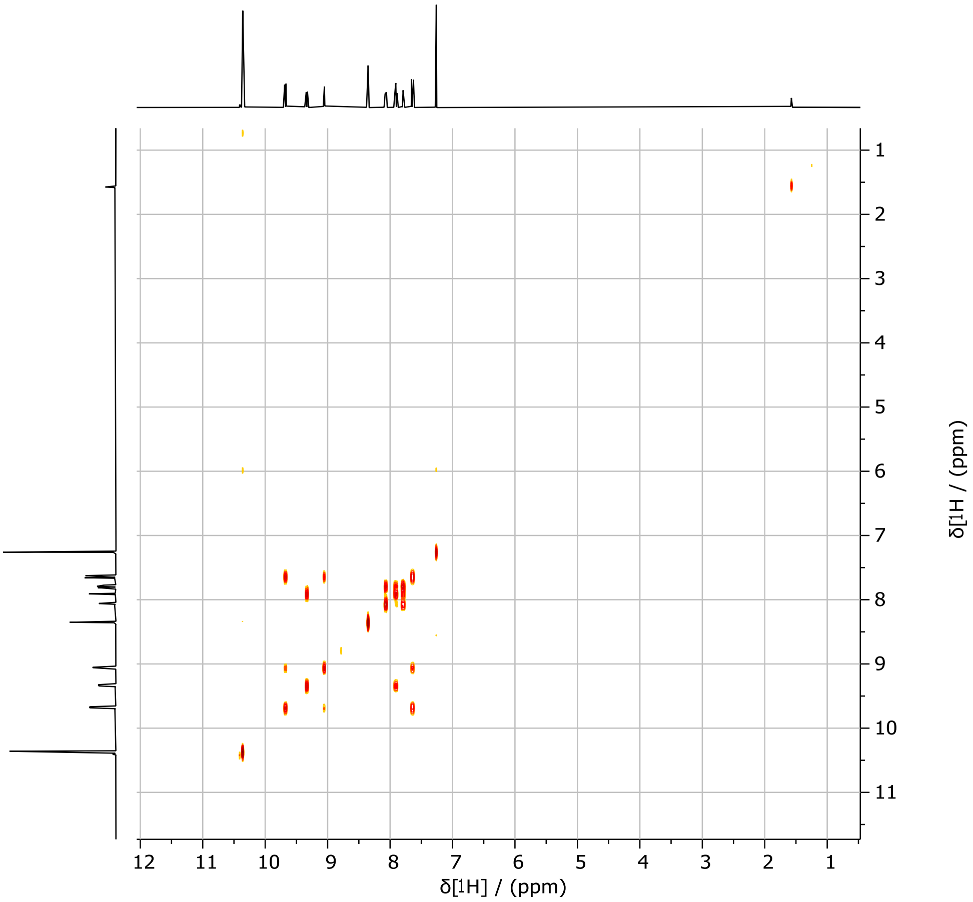

Figure SI-4-5: ^{1}H^{1}H COSY-NMR (400 MHz, 400 MHz) of compound bhq$^{5\text{-CHO}}$H, **7.2**, in $CDCl_3$ measured at r.t.

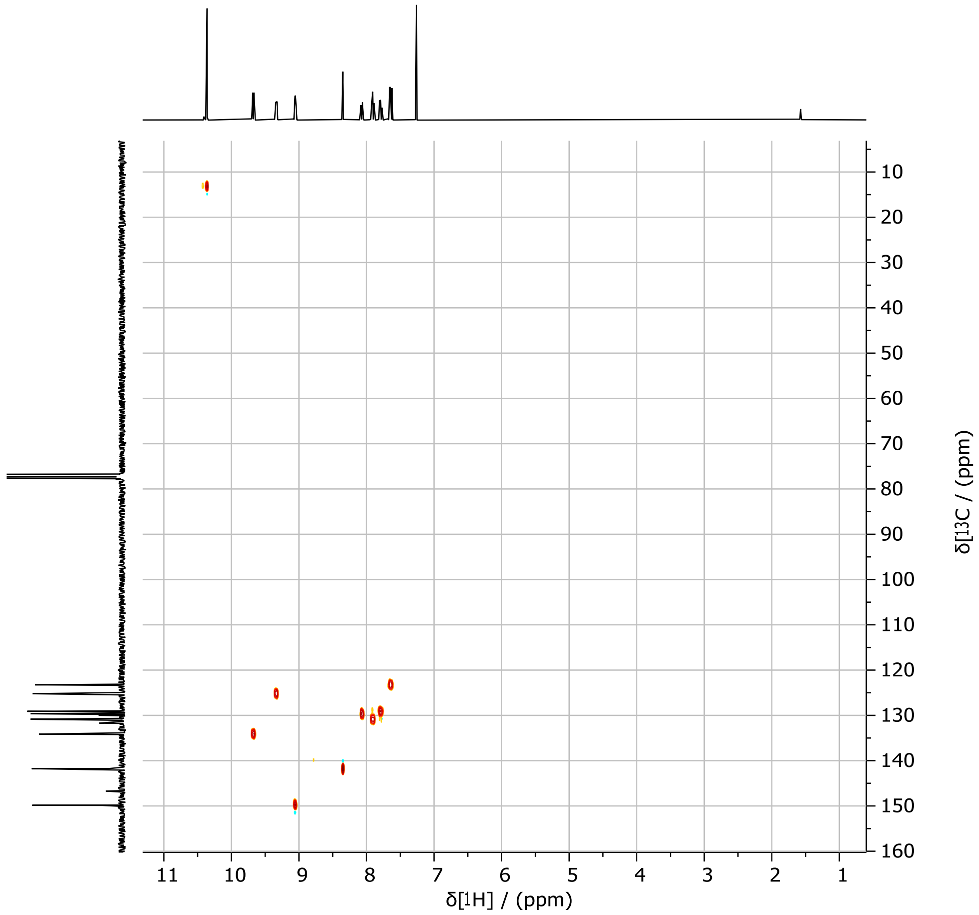

Figure SI-4-6: HSQC-NMR (400 MHz, 101 MHz) of compound bhq$^{5\text{-CHO}}$H, **7.2**, in $CDCl_3$ measured at r.t.

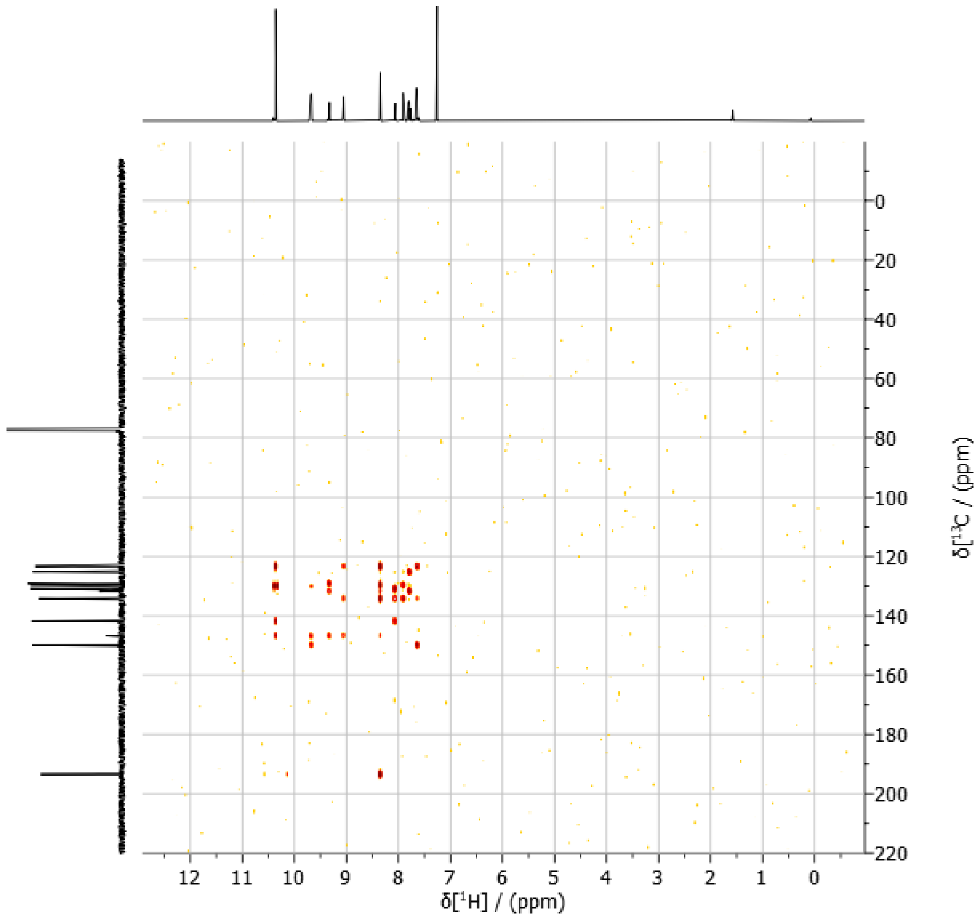

Figure SI-4-7: HMBC-NMR (400 MHz, 101 MHz) of compound bhq$^{5\text{-CHO}}$H, **7.2**, in $CDCl_3$ measured at r.t.

4.1.3. NMR Spectra of bhq$^{5\text{-}CH_2NMe_2}$H, 7.3

For the synthetic procedures see subsection 2.1.3.

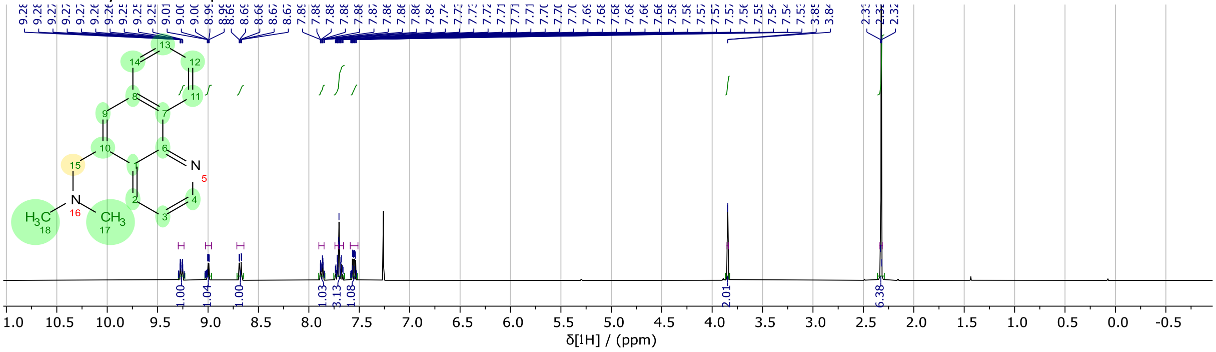

Figure SI-4-8: ^{1}H-NMR (400 MHz) spectrum of compound bhq$^{5\text{-}CH_2NMe_2}$H,**7.3** , in $CDCl_3$ measured at r.t. The spectrum was referenced to the residual solvent peak: ^{1}H-NMR ($CDCl_3$) δ = 7.26 ppm.

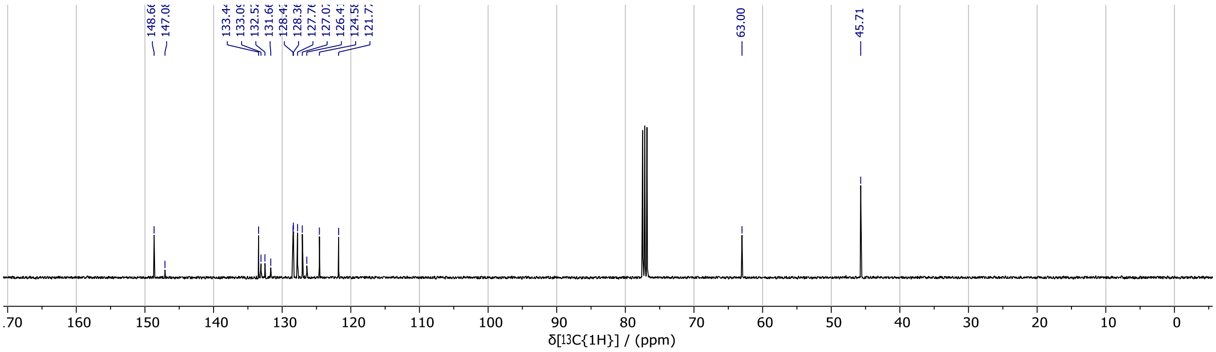

Figure SI-4-9: ^{13}C{^{1}H}-NMR (101 MHz) spectrum of compound bhq$^{5\text{-}CH_2NMe_2}$H, **7.3** , in $CDCl_3$measured at r.t. The spectrum was referenced to the residual solvent peak: ^{13}C -NMR ($CDCl_3$) δ = 77.16 ppm.

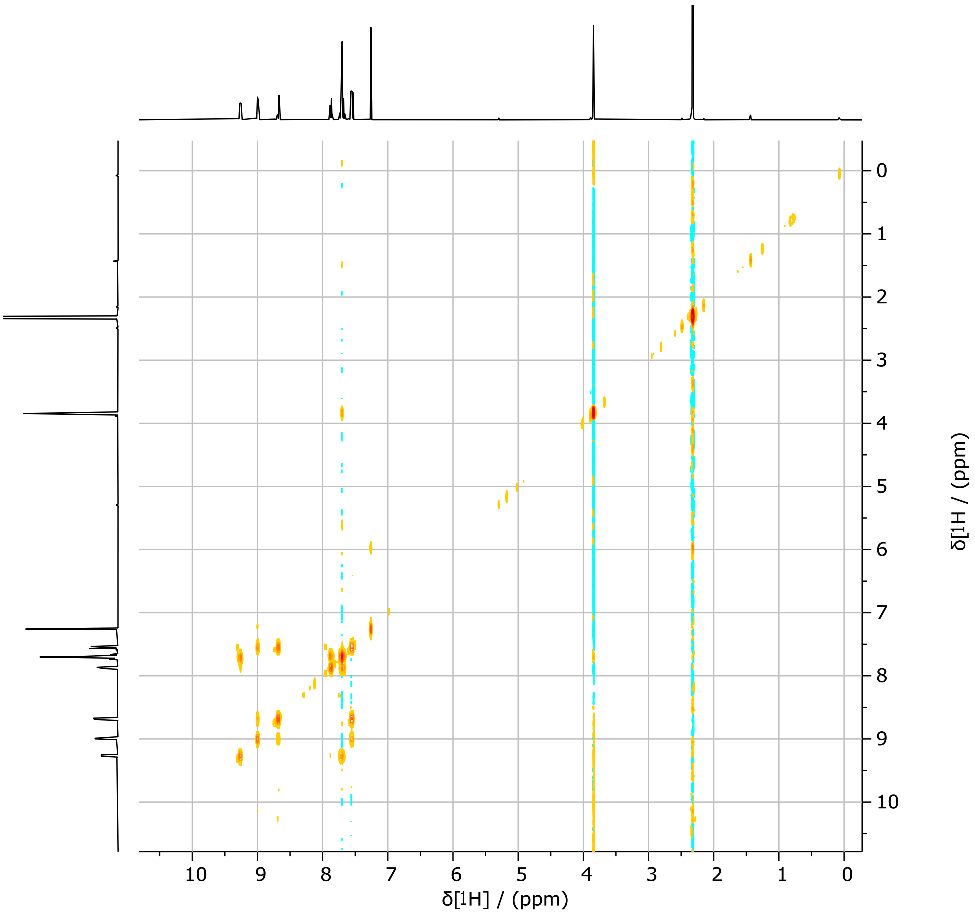

Figure SI-4-10: ^{1}H^{1}H COSY-NMR (400 MHz, 400 MHz) of compound bhq$^{5\text{-}CH_2NMe_2}$H, **7.3** , in $CDCl_3$ measured at r.t.

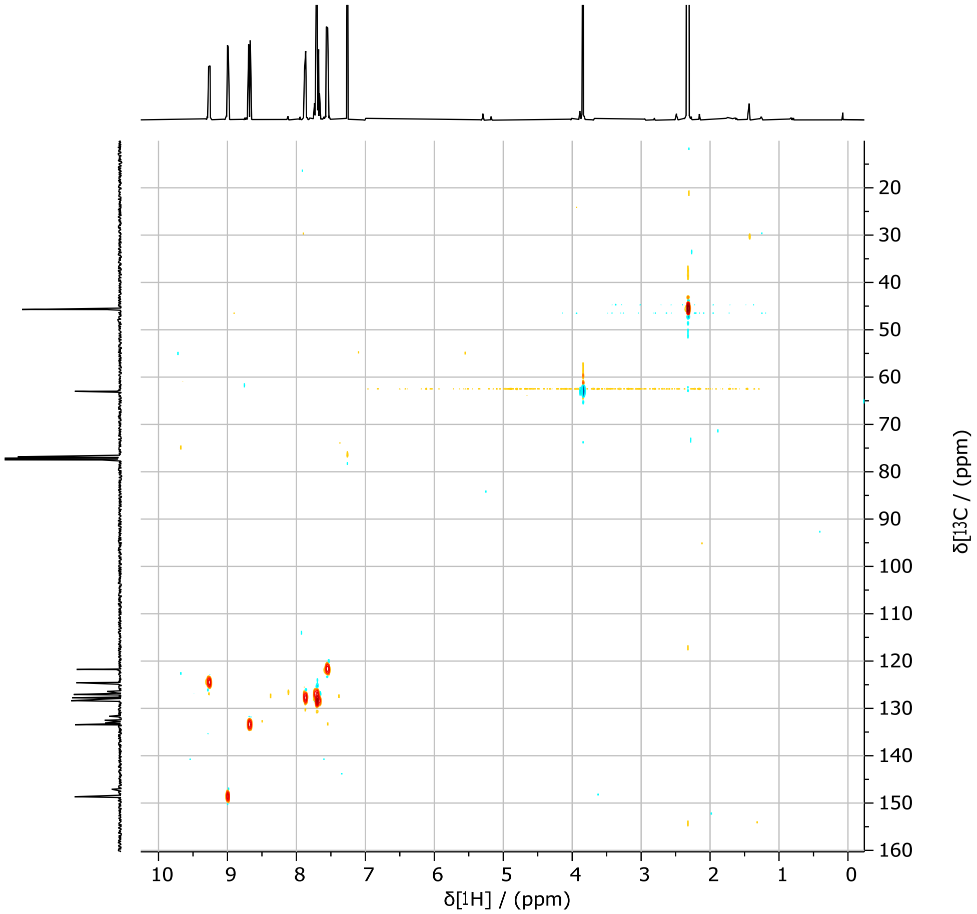

Figure SI-4-11: HSQC-NMR (400 MHz, 101 MHz) of compound bhq$^{5\text{-}CH_2NMe_2}$H, **7.3** , in $CDCl_3$ measured at r.t.

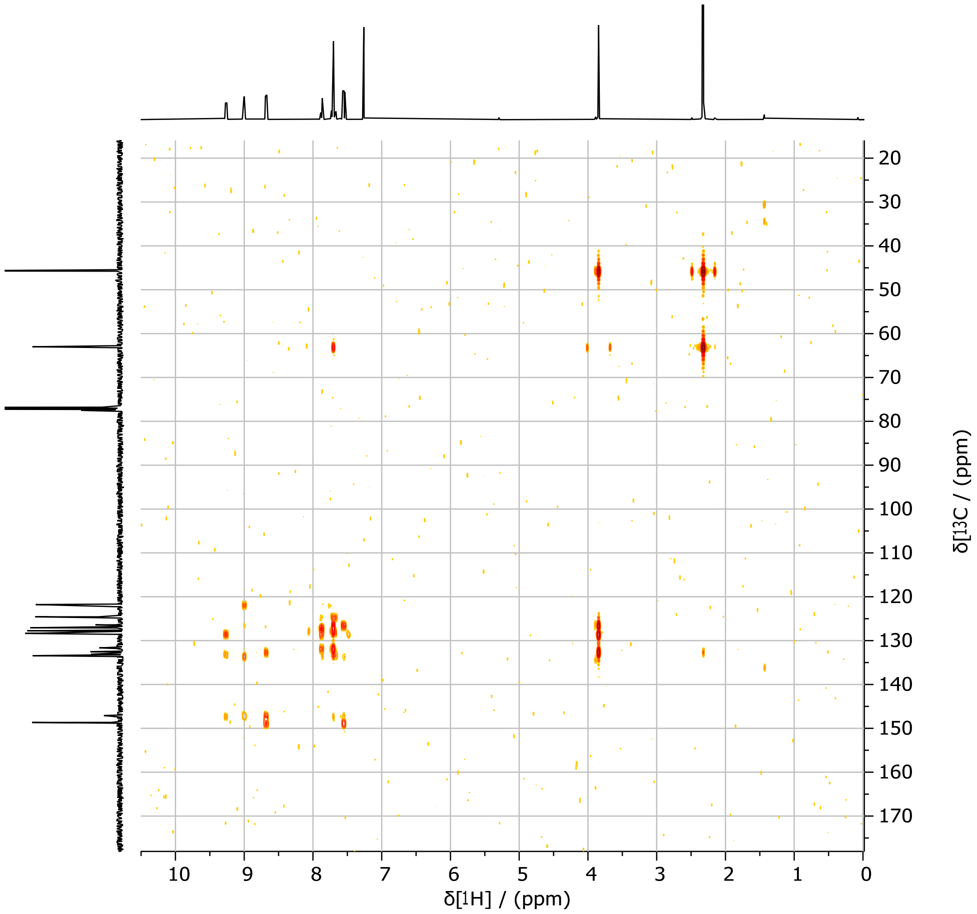

Figure SI-4-12: HMBC-NMR (400 MHz, 101 MHz) of compound bhq$^{5\text{-}CH_2NMe_2}$H, **7.3** , in $CDCl_3$ measured at r.t.

4.1.4. NMR Spectra of [bhq$^{5\text{-}CH_2NMe_3}$H$^+$]$^-$I, [7.4$^+$]$^-$I

For the synthetic procedures see subsection 2.1.4.

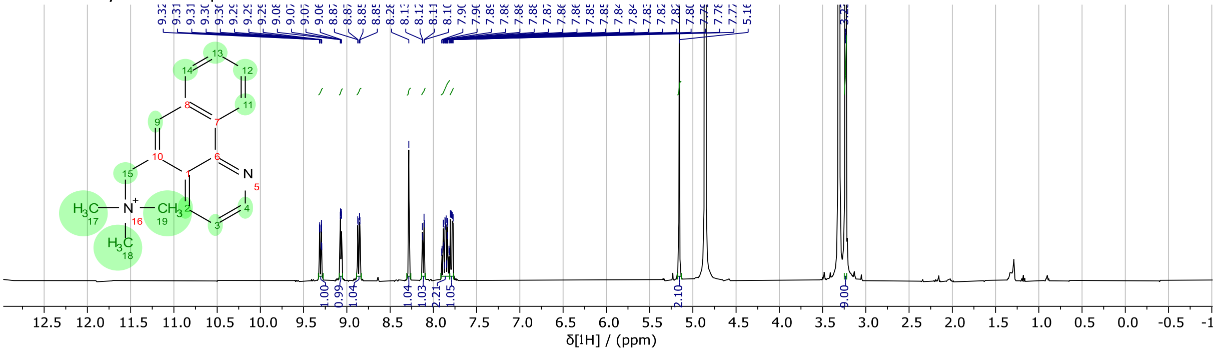

Figure SI-4-13: ^{1}H-NMR (400 MHz) spectrum of compound [bhq$^{5\text{-}CH2NMe3}$H$^+$]$^-$I, [**7.4$^+$**]$^-$I, in MeOD-d^4 measured at r.t. The spectrum was referenced to the residual solvent peak: ^{1}H-NMR ($CDCl_3$) δ = 3.31 ppm.

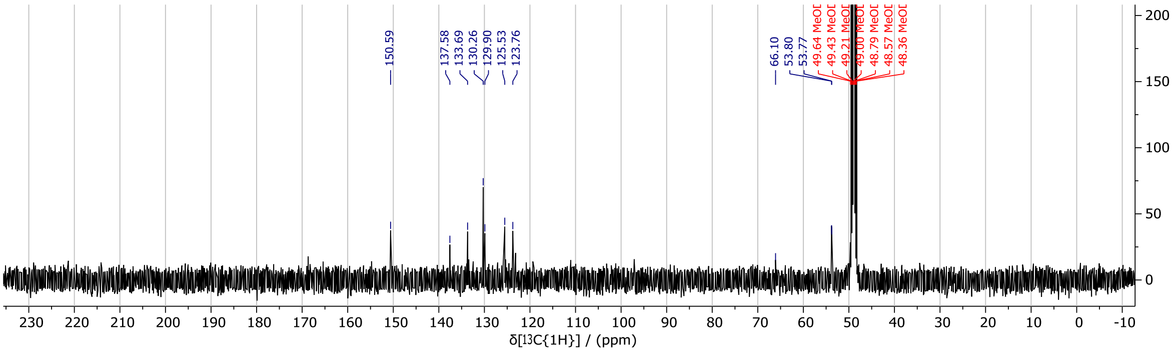

Figure SI-4-14: ^{13}C{^{1}H}-NMR (101 MHz) spectrum of compound [bhq$^{5\text{-}CH2NMe3}$H$^+$]$^-$I, [**7.4$^+$**]$^-$I, in MeOD-d^4 measured at r.t. The spectrum was referenced to the residual solvent peak: ^{13}C -NMR MeOD-d^4) δ = 49.00 ppm.

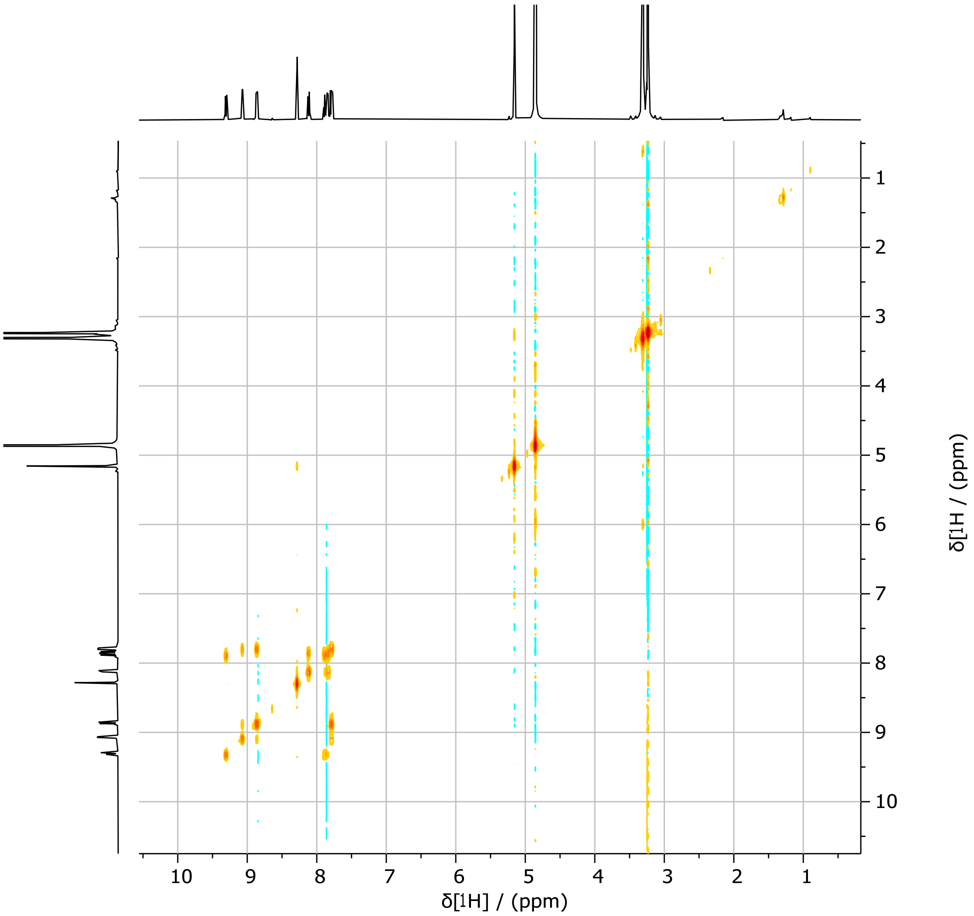

Figure SI-4-15: ^{1}H^{1}H COSY-NMR (400 MHz, 400 MHz) of compound [bhq$^{5\text{-}CH2NMe3}$H$^+$]$^-$I, [**7.4$^+$**]$^-$I, in MeOD-d^4 measured at r.t.

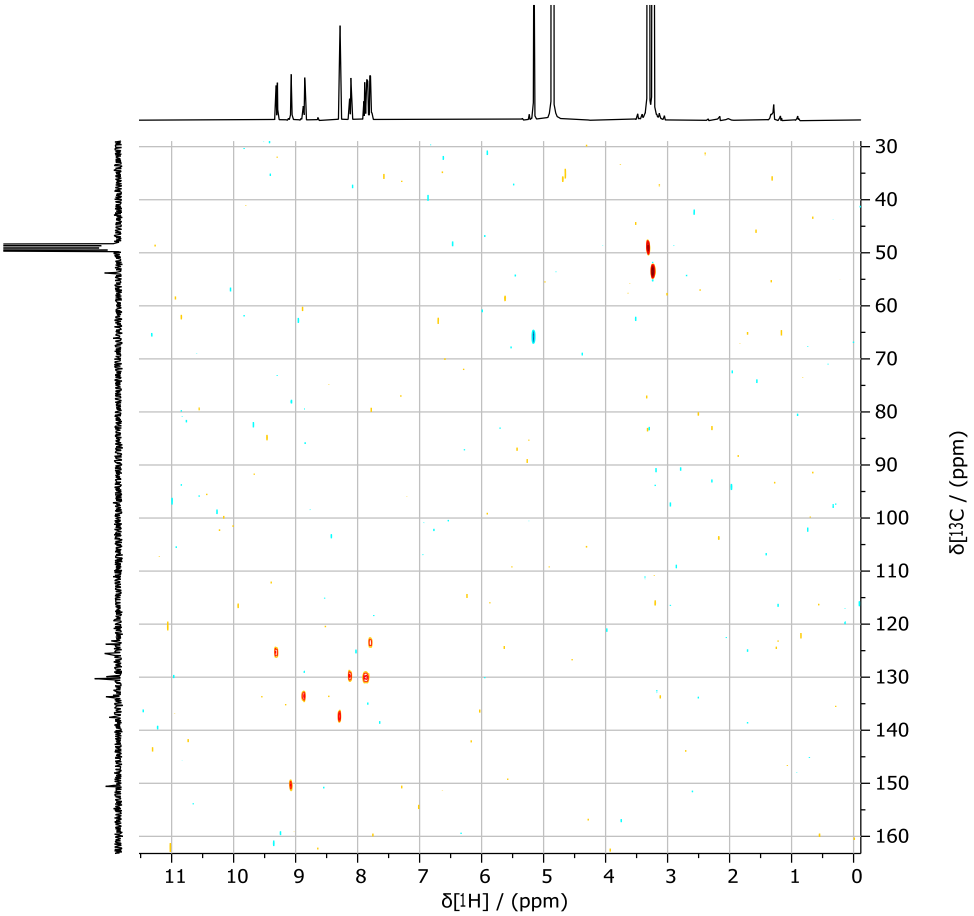

Figure SI-4-16: HSQC-NMR (400 MHz, 101 MHz) of compound [bhq$^{5\text{-}CH2NMe3}$H^{+}]$^{-}$I, [**7.4**$^{+}$]$^{-}$I, in MeOD-d^{4} measured at r.t.

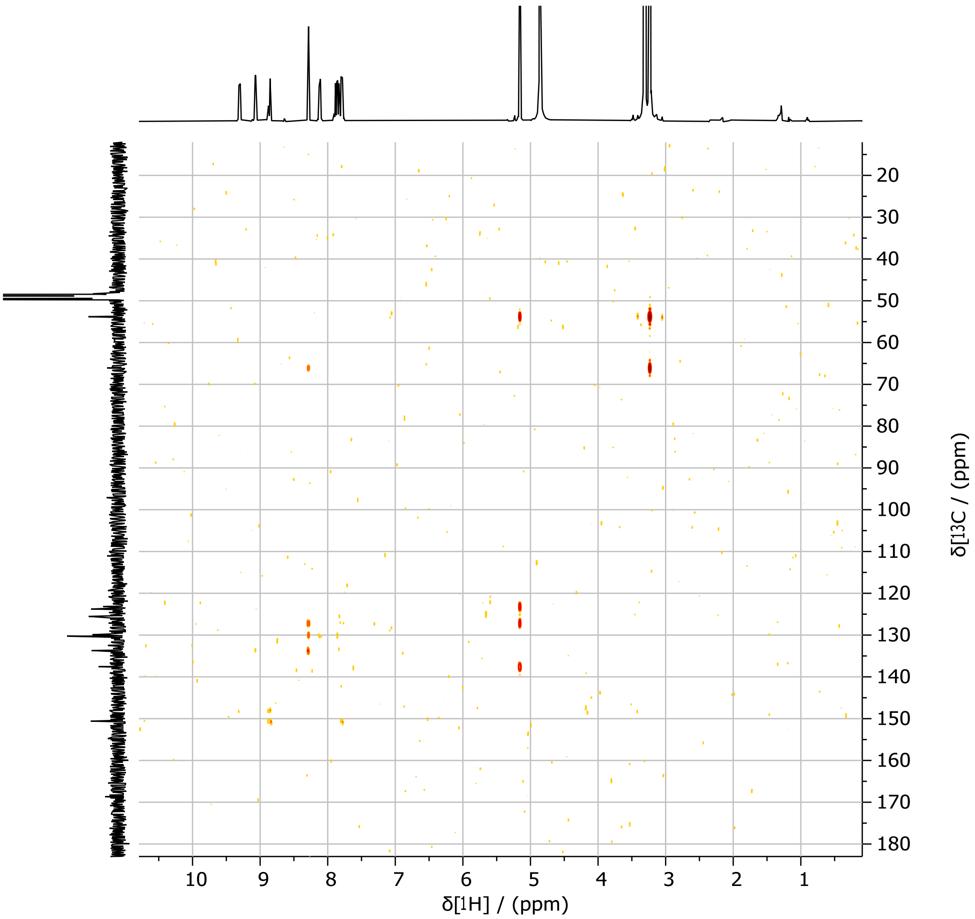

Figure SI-4-17: HMBC-NMR (400 MHz, 101 MHz) of compound [bhq$^{5\text{-}CH2NMe3}$H^{+}]$^{-}$I, [**7.4**$^{+}$]$^{-}$I, in MeOD-d^{4} measured at r.t.

4.1.5. NMR Spectra of [bhq$^{5\text{-}CH_2NMe_3}$H$^+$]$^-$BArF, [7.5$^+$]$^-$BArF

For the synthetic procedures see subsection 2.1.5.

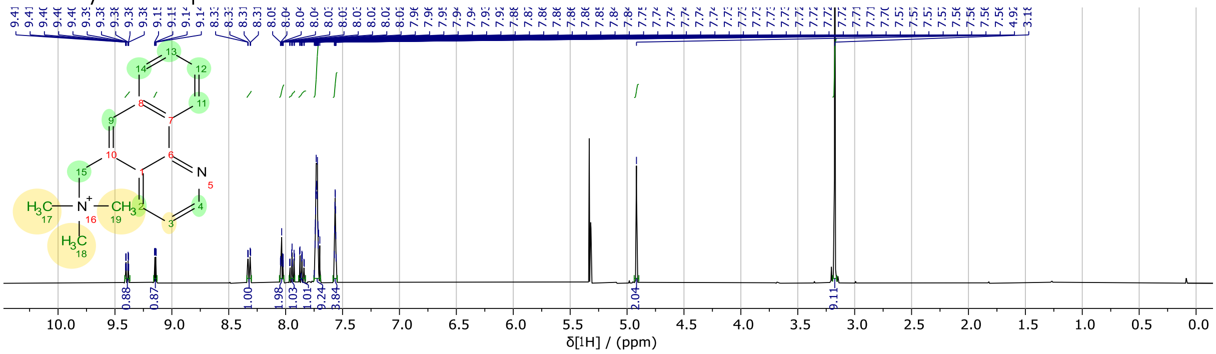

Figure SI-4-18: ^{1}H-NMR (400 MHz) spectrum of compound [bhq$^{5\text{-}CH2NMe3}$H$^+$]$^-$BArF, [**7.5**$^+$]$^-$BArF, in CD_2Cl_2 measured at r.t. The spectrum was referenced to the residual solvent peak: ^{1}H-NMR (CD_2Cl_2) δ = 5.32 ppm.

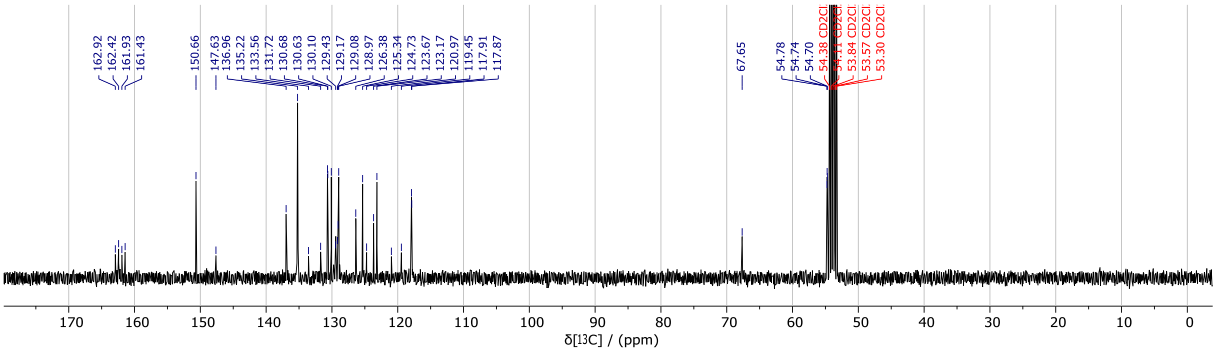

Figure SI-4-19: ^{13}C{^{1}H}-NMR (101 MHz) spectrum of compound [bhq$^{5\text{-}CH2NMe3}$H$^+$]$^-$BArF, [**7.5**$^+$]$^-$BArF, in CD_2Cl_2measured at r.t. The spectrum was referenced to the residual solvent peak: ^{13}C -NMR (CD_2Cl_2) δ = 53.84 ppm.

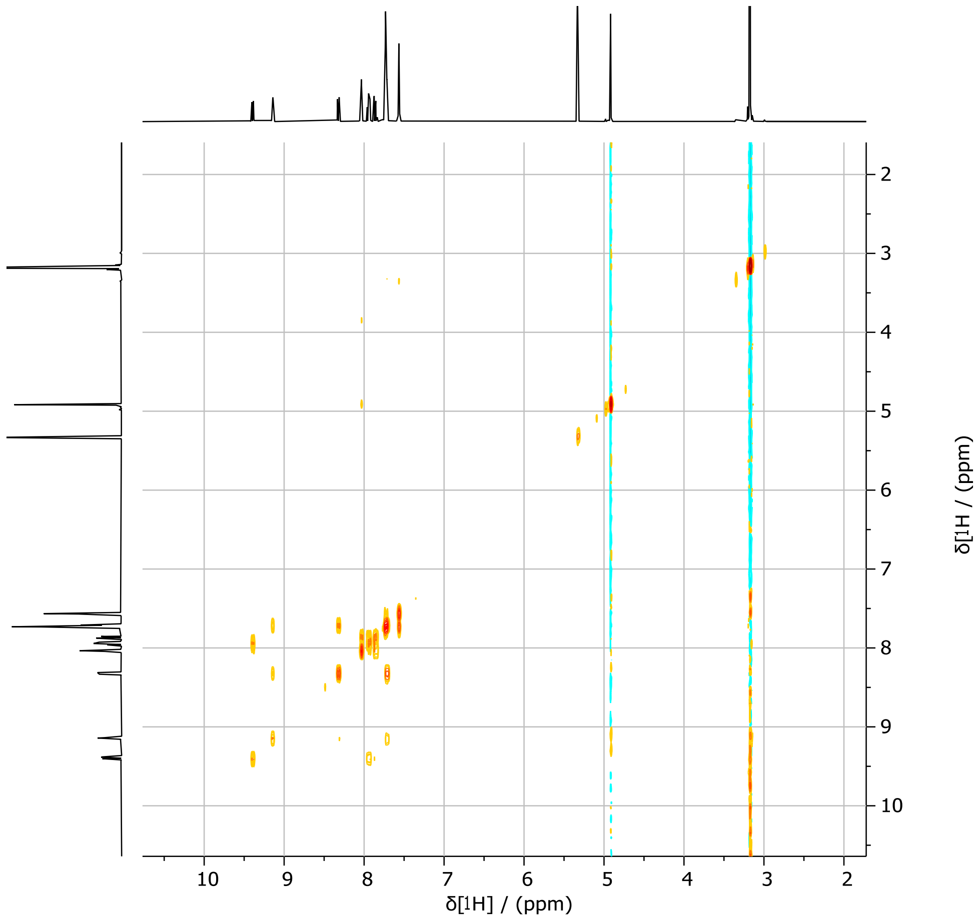

Figure SI-4-20: ^{1}H^{1}H COSY-NMR (400 MHz, 400 MHz) of compound [bhq$^{5\text{-}CH2NMe3}$H$^+$]$^-$BArF, [**7.5**$^+$]$^-$BArF**,** in CD_2Cl_2 measured at r.t.

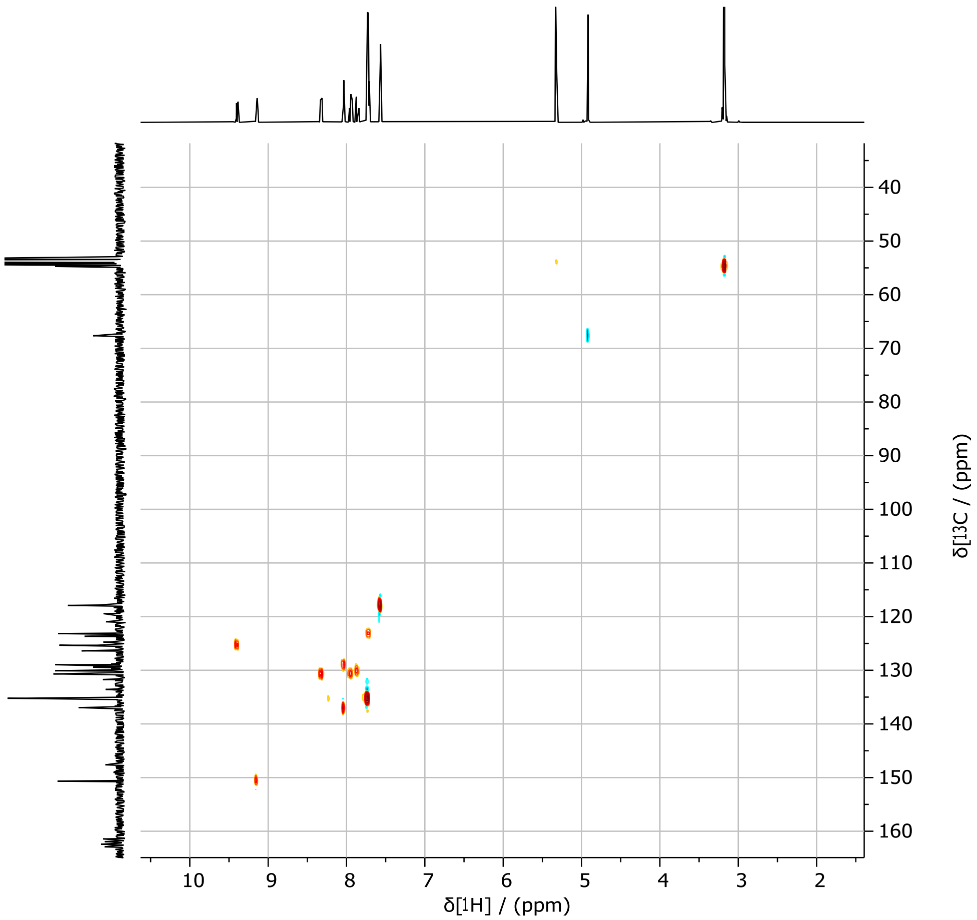

Figure SI-4-21: HSQC-NMR (400 MHz, 101 MHz) of compound [bhq$^{5\text{-}CH2NMe3}$H^{+}]$^{-}$BArF, [**7.5**$^{+}$]$^{-}$BArF, in CD_2Cl_2 measured at r.t.

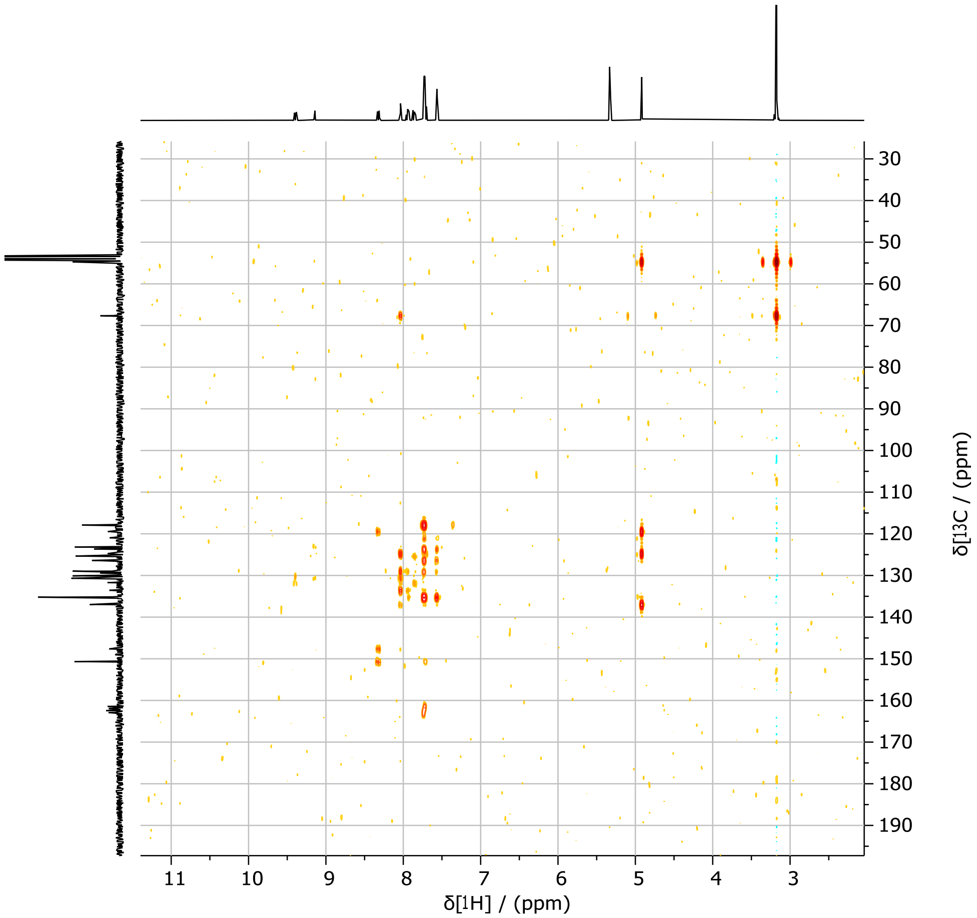

Figure SI-4-22: HMBC-NMR (400 MHz, 101 MHz) of compound [bhq$^{5\text{-}CH2NMe3}$H^{+}]$^{-}$BArF, [**7.5**$^{+}$]$^{-}$BArF, in CD_2Cl_2 measured at r.t.

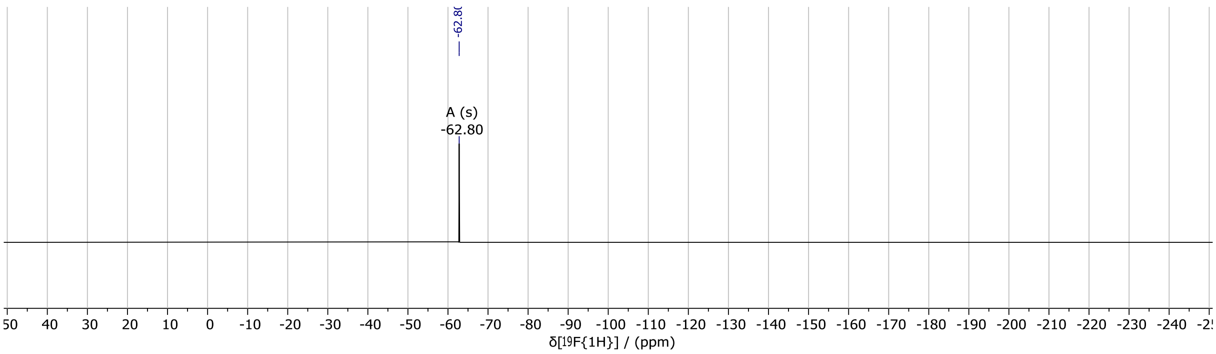

Figure SI-4-23: $^{19}F\{^{1}H\}$-NMR (377 MHz) spectrum of compound [bhq$^{5\text{-}CH2NMe3}$H^{+}]$^{-}$BArF, [**7.5**$^{+}$]$^{-}$BArF, in CD_2Cl_2 measured at r.t.

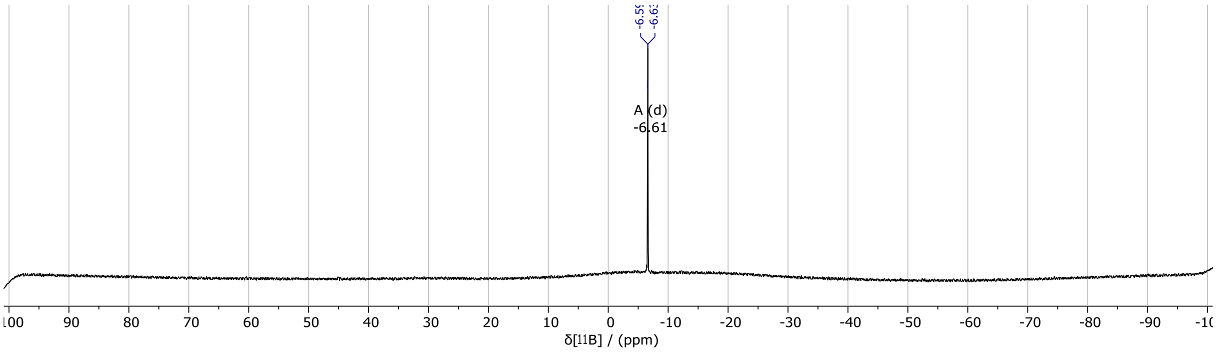

Figure SI-4-24: $^{11}B\{^{1}H\}$-NMR (128 MHz) spectrum of compound [bhq$^{5\text{-}CH2NMe3}$H^{+}]$^{-}$BArF, [**7.5**$^{+}$]$^{-}$BArF, in CD_2Cl_2 measured at r.t.

4.1.6. NMR Spectra of [Pd(bhq$^{5\text{-}CH_2NMe_3}$)(OAc)]$_n$ n BArF, [7.6^{n+}]n^{-}BArF

For the synthetic procedures see subsection 2.1.6.

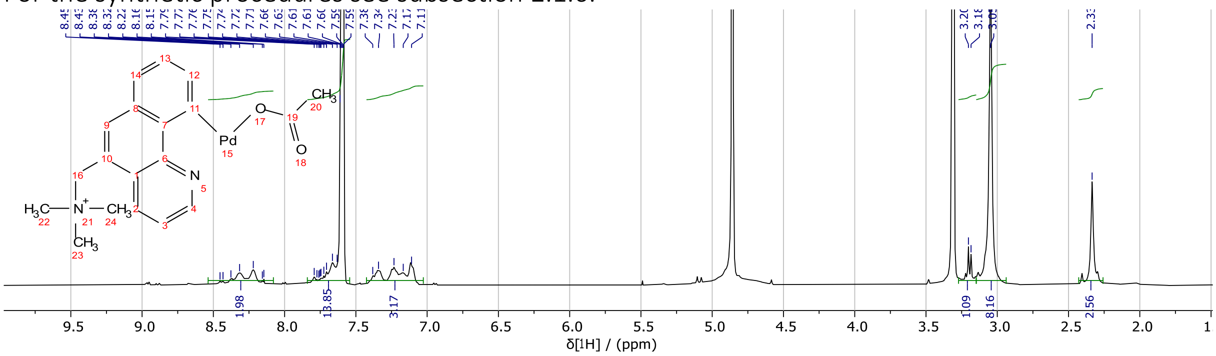

Figure SI-4-25: ^{1}H-NMR (400 MHz) spectrum of compound [Pd(bhq$^{5\text{-}CH2NMe3}$)(OAc)]$_n$ n BArF, [**7.6**$^{n+}$]n^{-}BArF, in MeOD-d^4 measured at r.t. The spectrum was referenced to the residual solvent peak: ^{1}H-NMR (MeOD-d^4) δ = 3.31 ppm.

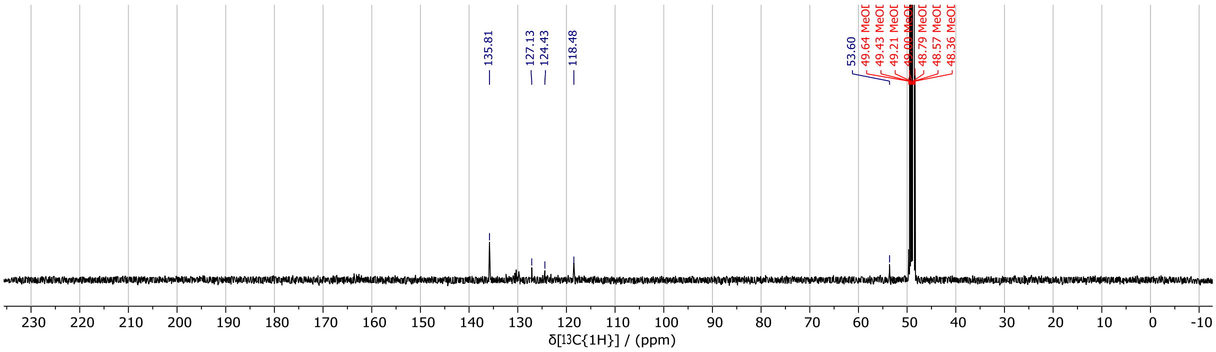

Figure SI-4-26: $^{13}C\{^{1}H\}$-NMR (101 MHz) spectrum of compound [Pd(bhq$^{5\text{-}CH2NMe3}$)(OAc)]$_n$ n BArF, [**7.6**$^{n+}$]n^{-}BArF, in MeOD-d^4 measured at r.t. The spectrum was referenced to the residual solvent peak: ^{13}C -NMR (MeOD-d^4) δ = 49.00 ppm.

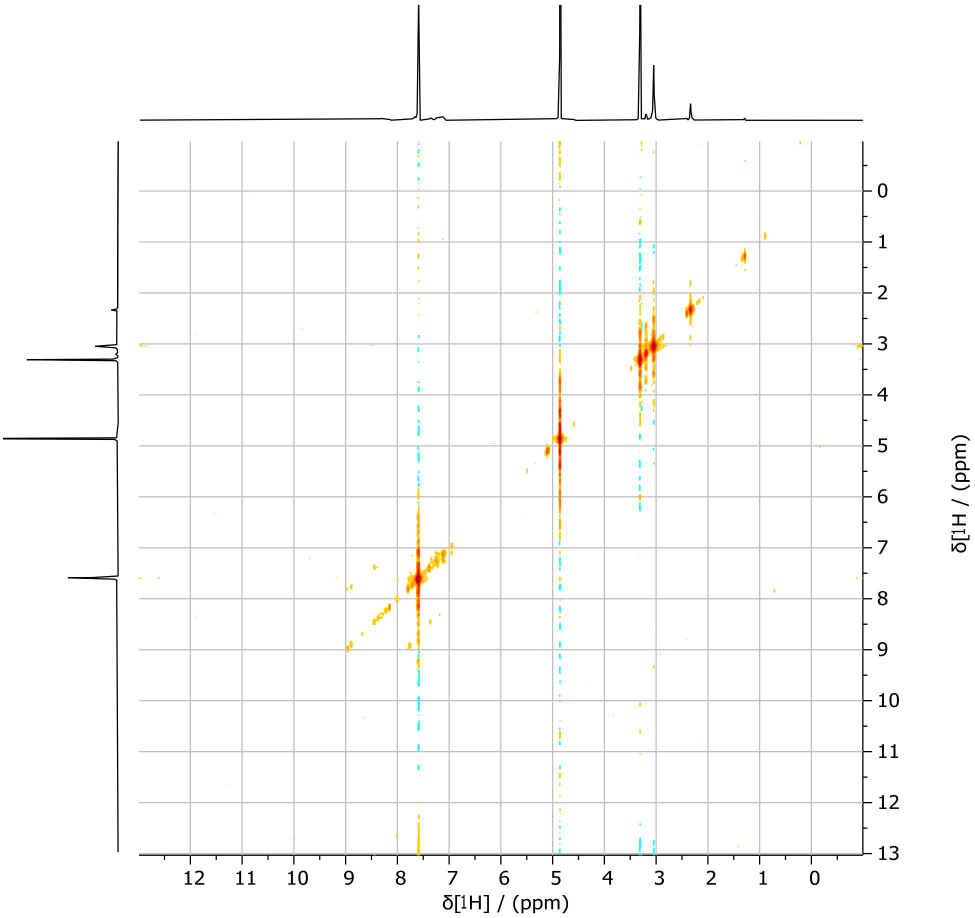

Figure SI-4-27: ^{1}H^{1}H COSY-NMR (400 MHz, 400 MHz) of compound [Pd(bhq$^{5\text{-}CH2NMe3}$)(OAc)]$_n$ n BArF, [**7.6**$^{n+}$]n$^-$BArF, in MeOD-d^4 measured at r.t.

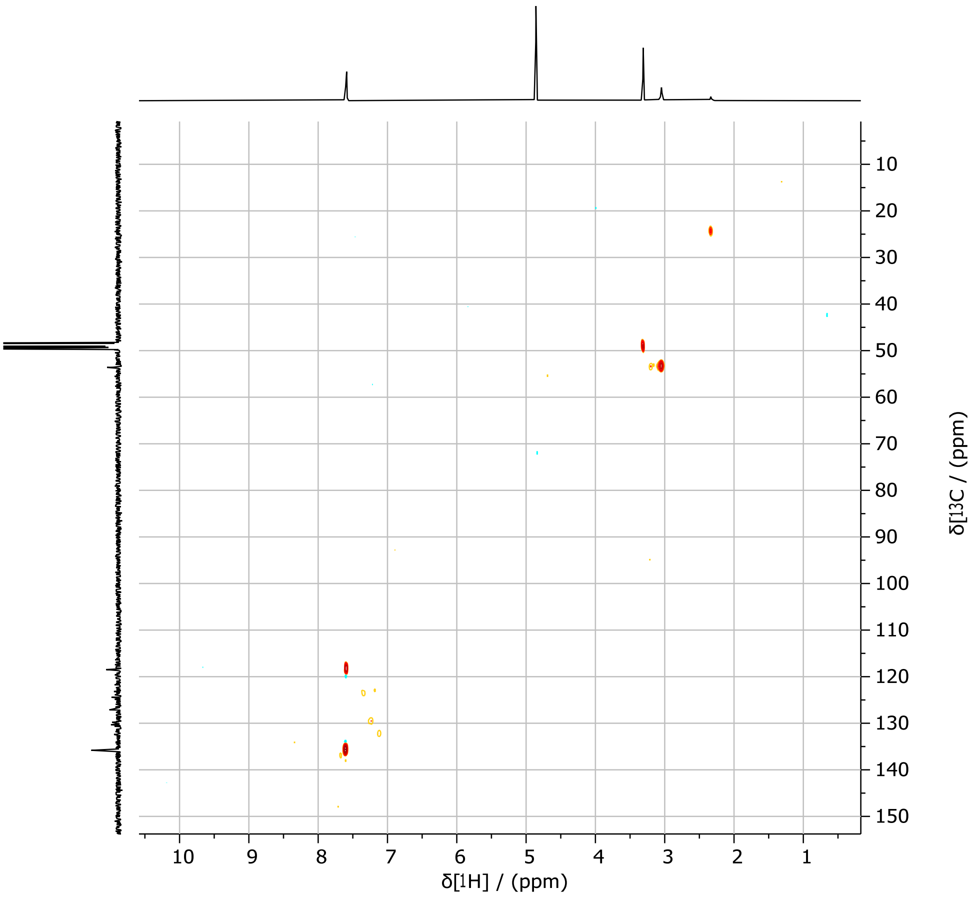

Figure SI-4-28: HSQC-NMR (400 MHz, 101 MHz) of compound $[Pd(bhq^{5\text{-}CH2NMe3})(OAc)]_n$ n BArF, [**7.6**$^{n+}$]n^{-}BArF, in MeOD-d^4 measured at r.t.

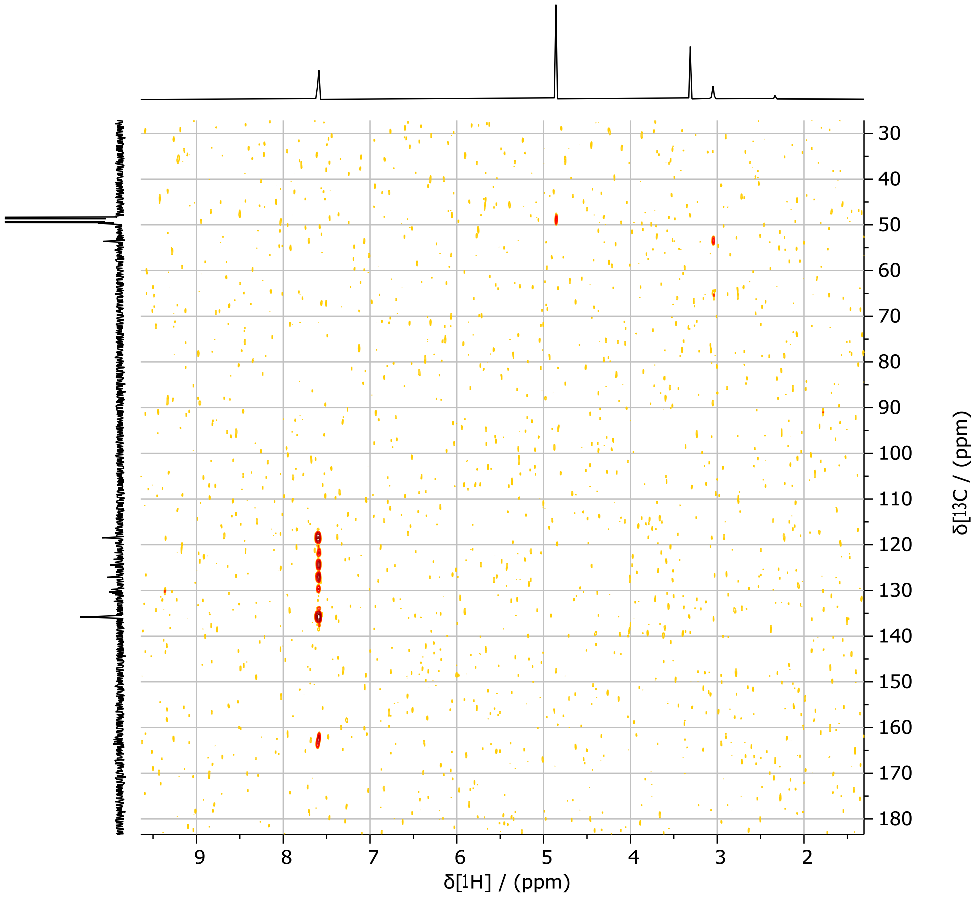

Figure SI-4-29: HMBC-NMR (400 MHz, 101 MHz) of compound $[Pd(bhq^{5\text{-}CH2NMe3})(OAc)]_n$ n BArF, [**7.6**$^{n+}$]n^{-}BArF, in MeOD-d^4 measured at r.t.

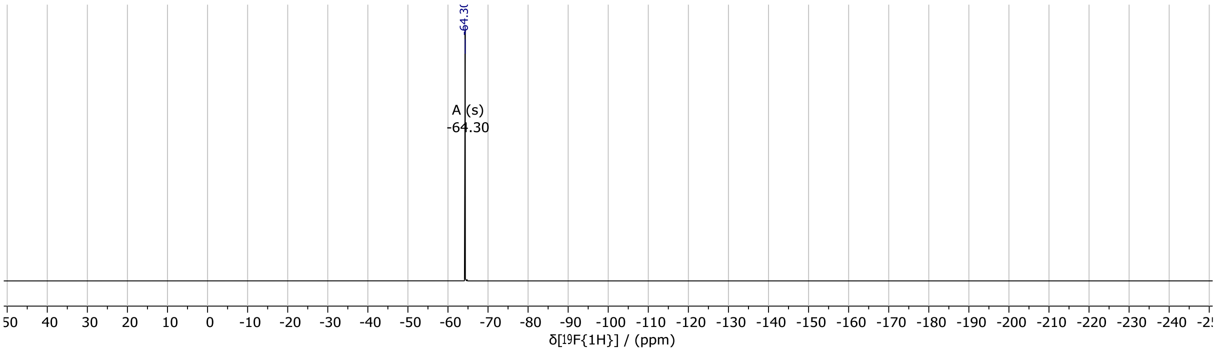

Figure SI-4-30: ^{19}F{^{1}H}-NMR (377 MHz) spectrum of compound $[Pd(bhq^{5\text{-}CH2NMe3})(OAc)]_n$ n BArF, [**7.6**$^{n+}$]n^{-}BArF, ***in*** MeOD-d^4 ***measured at r.t.***

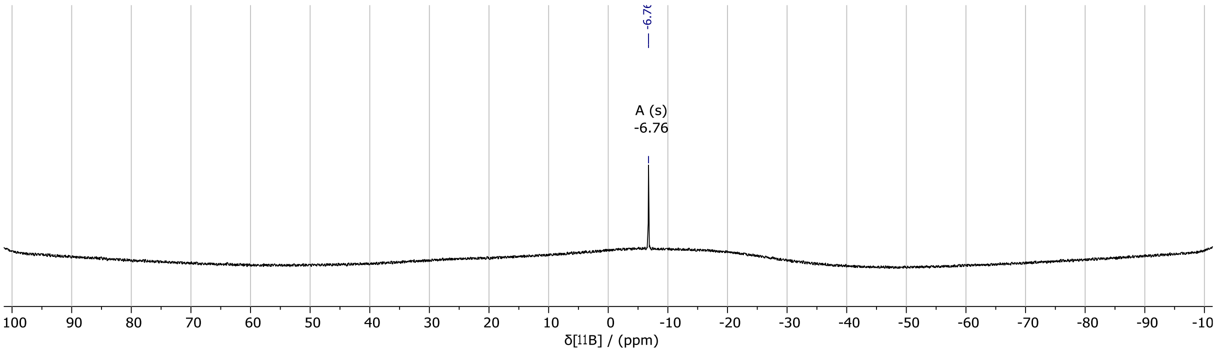

Figure SI-4-31: 11B{1H}-NMR (128 MHz) spectrum of compound $[Pd(bhq^{5\text{-}CH2NMe3})(OAc)]_n$ n BArF, [**7.6**$^{n+}$]n^{-}BArF, **in** MeOD-d4 **measured at r.t.**

4.1.7. NMR Spectra of [Pd(bhq$^{5\text{-}CH_2NMe_3}$)(Cl)(SEt_2)]BArF, [**7.7**$^+$]$^-$BArF

For the synthetic procedures see subsection 2.1.7.

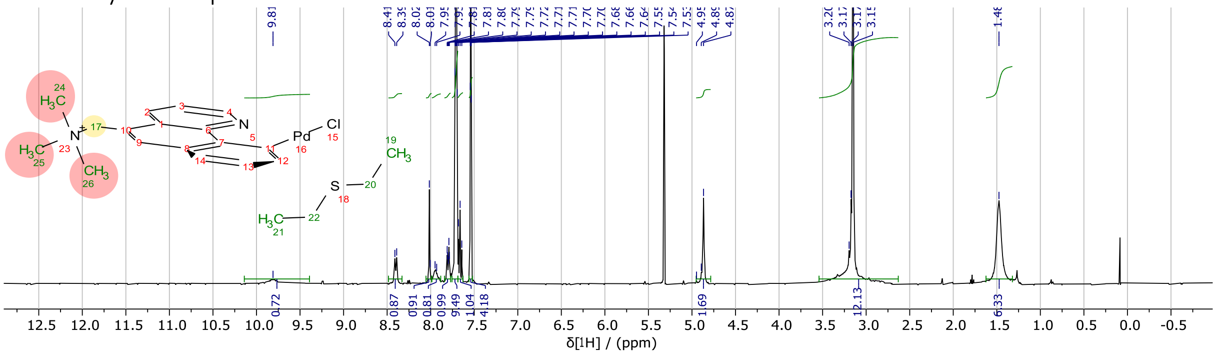

Figure SI-4-32: ^{1}H-NMR (400 MHz) spectrum of compound [Pd(bhq$^{5\text{-}CH_2NMe_3}$)(Cl)(SEt_2)]BArF, [**7.7**$^+$]$^-$BArF, in CD_2Cl_2 measured at r.t. The spectrum was referenced to the residual solvent peak: ^{1}H-NMR (CD_2Cl_2) δ = 5.32 ppm.

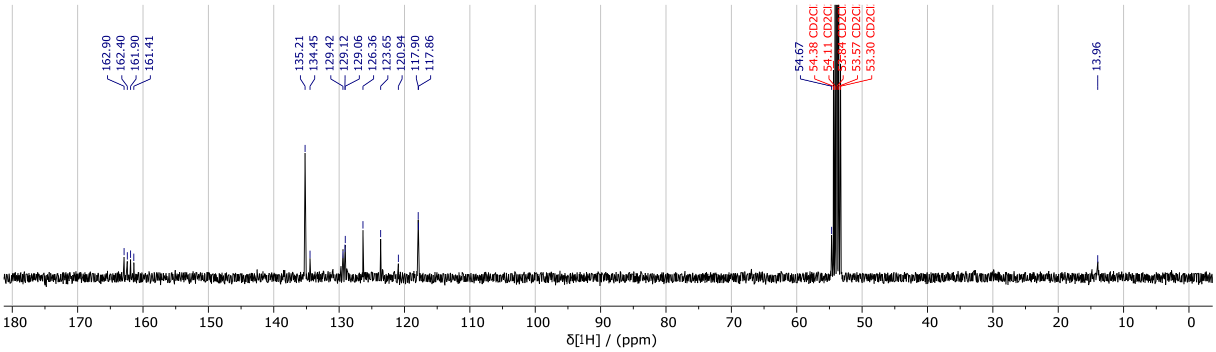

Figure SI-4-33: ^{13}C{^{1}H}-NMR (101 MHz) spectrum of compound [Pd(bhq$^{5\text{-}CH_2NMe_3}$)(Cl)(SEt_2)]BArF, [**7.7**$^+$]$^-$BArF, [9]BArF, in CD_2Cl_2measured at r.t. The spectrum was referenced to the residual solvent peak: ^{13}C -NMR (CD_2Cl_2) δ = 53.84 ppm.

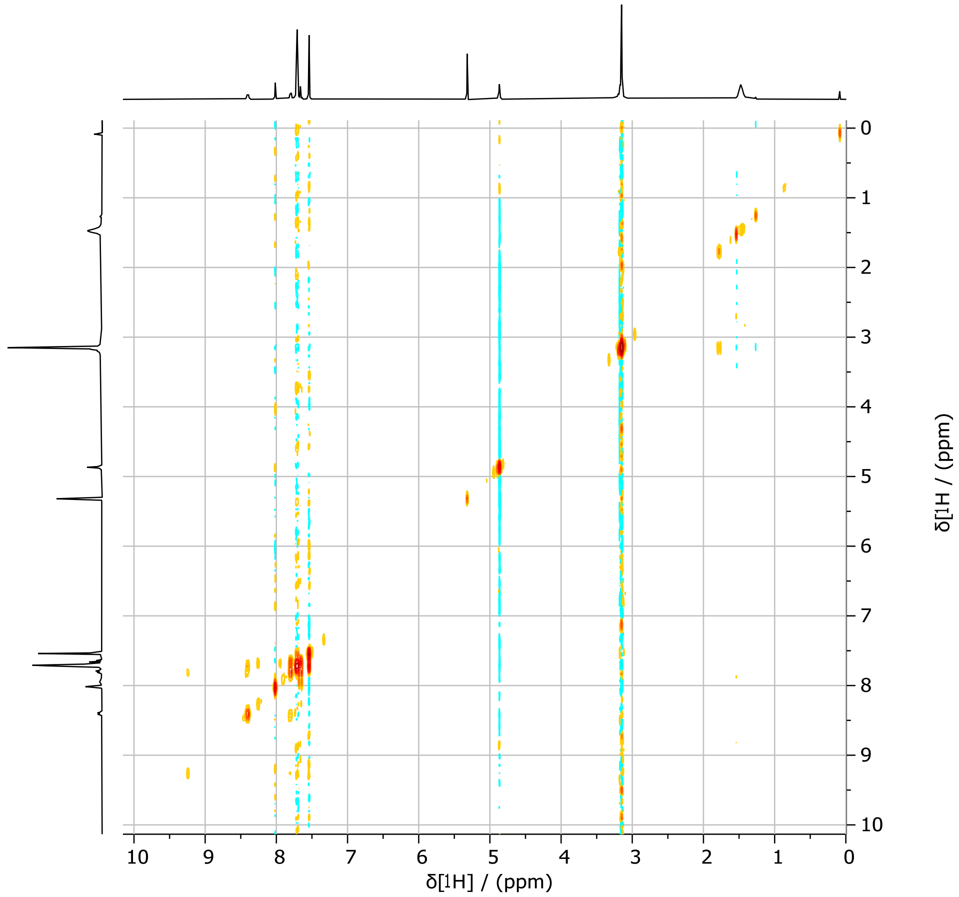

Figure SI-4-34: $^1H^1H$ COSY-NMR (400 MHz, 101 MHz) of compound $[Pd(bhq^{5\text{-}CH_2NMe_3})(Cl)(SEt_2)]BArF$, $[\mathbf{7.7}^{+}]^{-}BArF$, in CD_2Cl_2 measured at r.t.

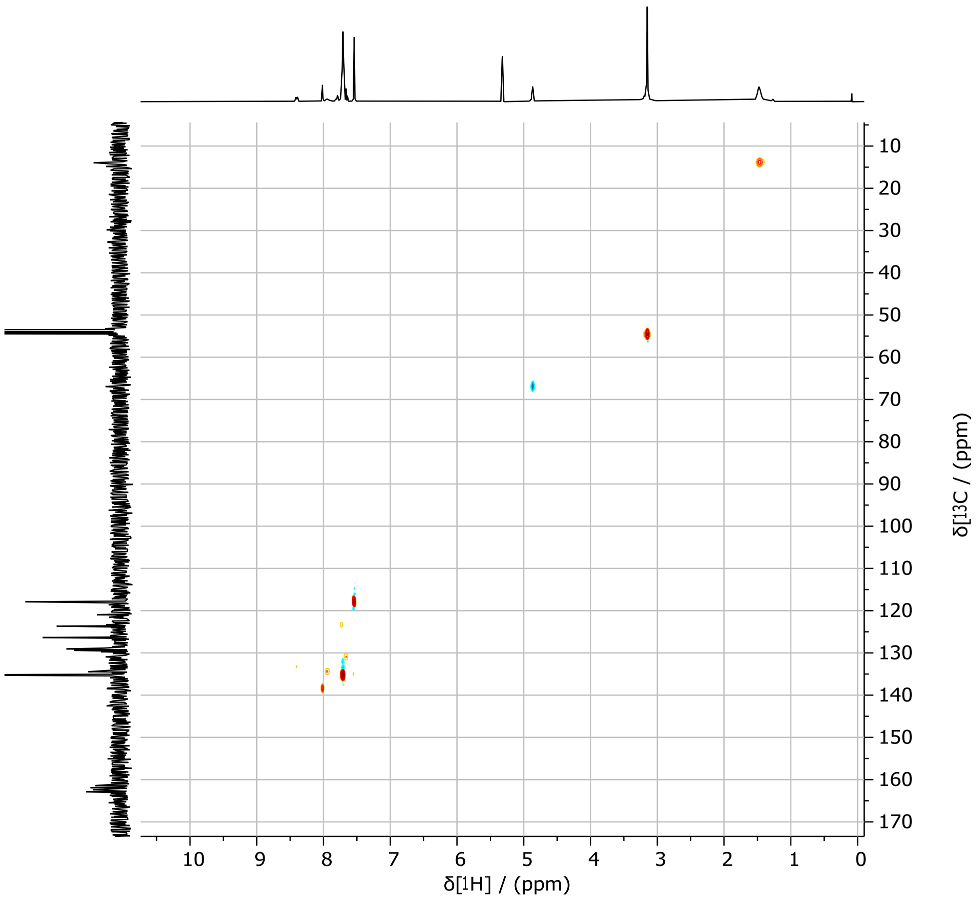

Figure SI-4-35: HSQC-NMR (400 MHz, 101 MHz) of compound $[Pd(bhq^{5\text{-}CH_2NMe_3})(Cl)(SEt_2)]BArF$, $[\mathbf{7.7}^{+}]^{-}BArF$, in CD_2Cl_2 measured at r.t.

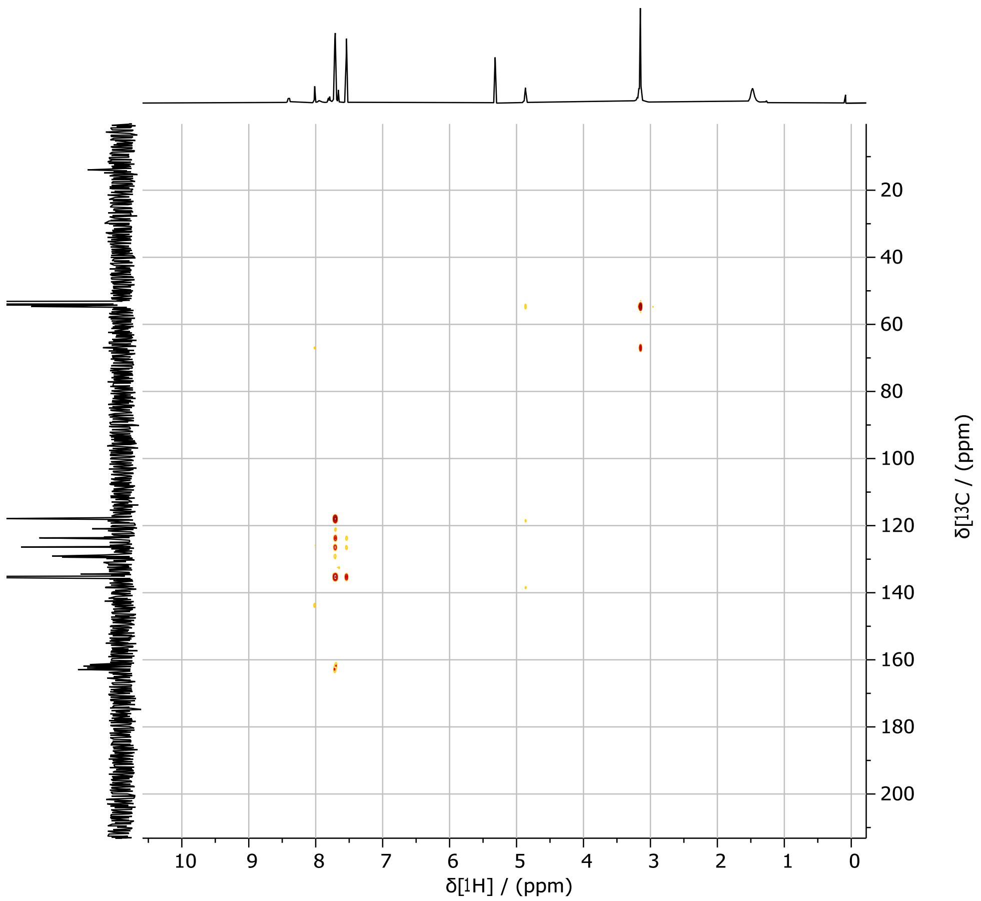

Figure SI-4-36: HMBC-NMR (400 MHz, 101 MHz) of compound [Pd(bhq$^{5\text{-}CH_2NMe_3}$)(Cl)(SEt$_2$)]BArF, [**7.7**$^+$]$^-$BArF, in CD_2Cl_2 measured at r.t.

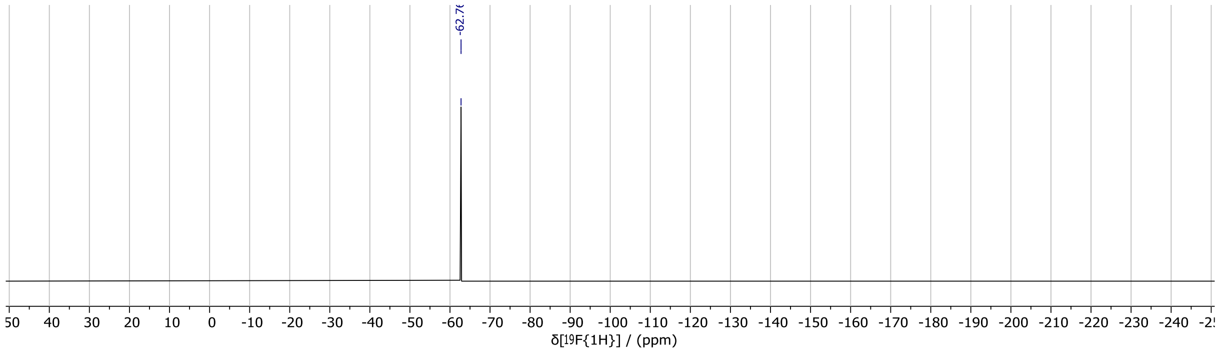

Figure SI-4-37: $^{19}F\{^1H\}$-NMR (377 MHz) spectrum of compound [Pd(bhq$^{5\text{-}CH_2NMe_3}$)(Cl)(SEt$_2$)]BArF, [**7.7**$^+$]$^-$BArF, in CD_2Cl_2 measured at r.t.

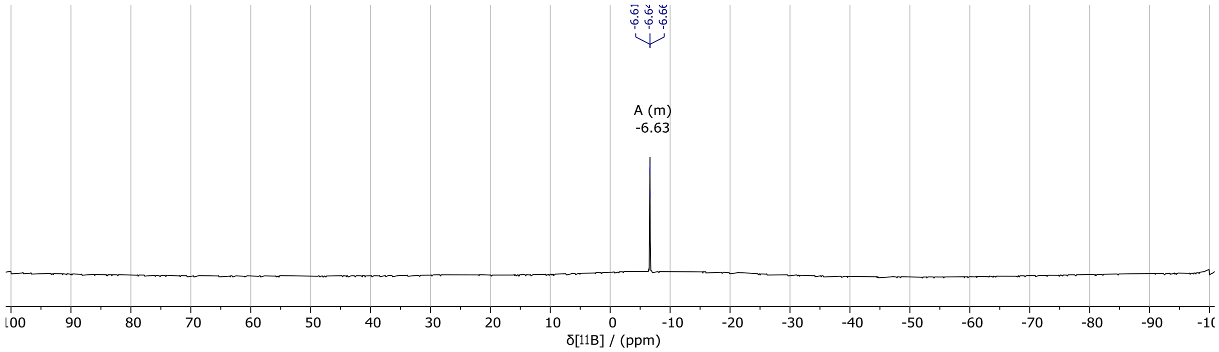

Figure SI-4-38: $^{11}B\{^1H\}$-NMR (128 MHz) spectrum of compound [Pd(bhq$^{5\text{-}CH_2NMe_3}$)(Cl)(SEt$_2$)]BArF, [**7.7**$^+$]$^-$BArF, in CD_2Cl_2 measured at r.t.

4.1.8. NMR Spectra of [Pd(bhq)(bhq$^{5\text{-}CH_2NMe_3}$)]BArF, [7$^+$]$^-$BArF

For the synthetic procedures see subsection 2.1.8.

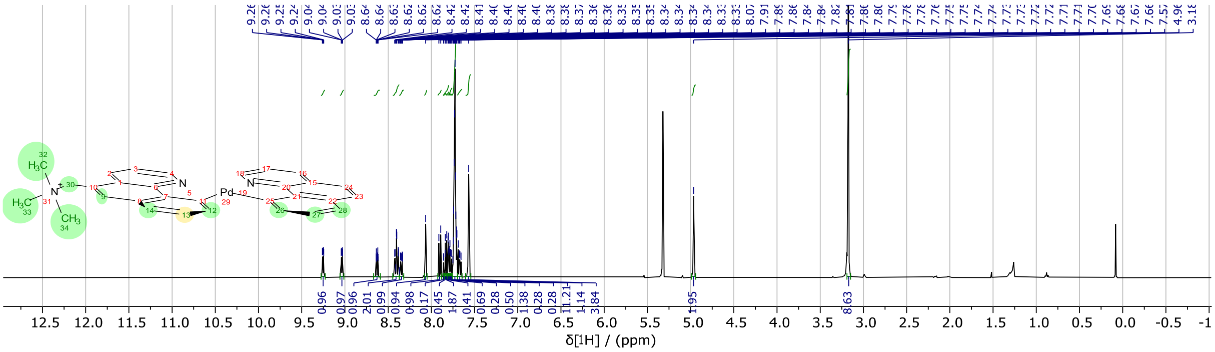

Figure SI-4-39: ^{1}H-NMR (400 MHz) spectrum of compound [Pd(bhq)(bhq$^{5\text{-}CH_2NMe_3}$)]BArF, [**7**$^{+}$]$^{-}$BArF, in CD_2Cl_2 measured at r.t. The spectrum was referenced to the residual solvent peak: ^{1}H-NMR (CD_2Cl_2) δ = 5.32 ppm.

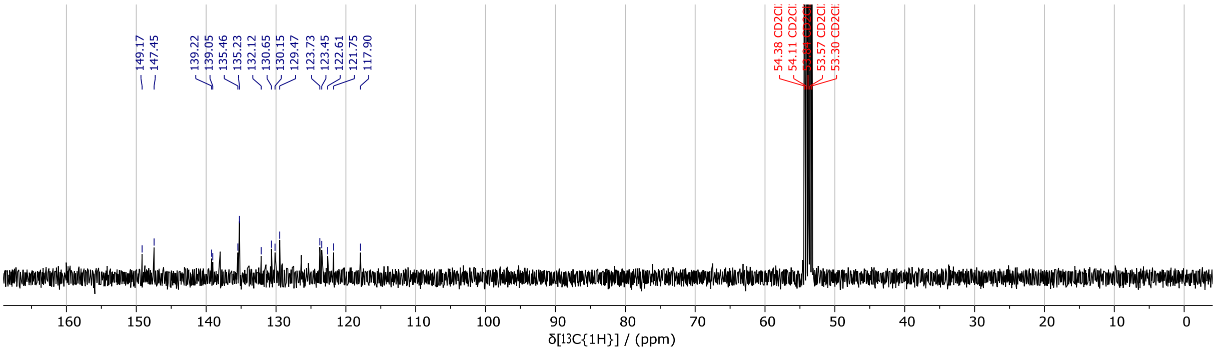

Figure SI-4-40: ^{13}C{^{1}H}-NMR (101 MHz) spectrum of compound [Pd(bhq)(bhq$^{5\text{-}CH_2NMe_3}$)]BArF, [**7**$^{+}$]$^{-}$BArF, in **CD_2Cl_2** measured at r.t. The spectrum was referenced to the residual solvent peak: ^{13}C -NMR (CD_2Cl_2) δ = 53.84 ppm.

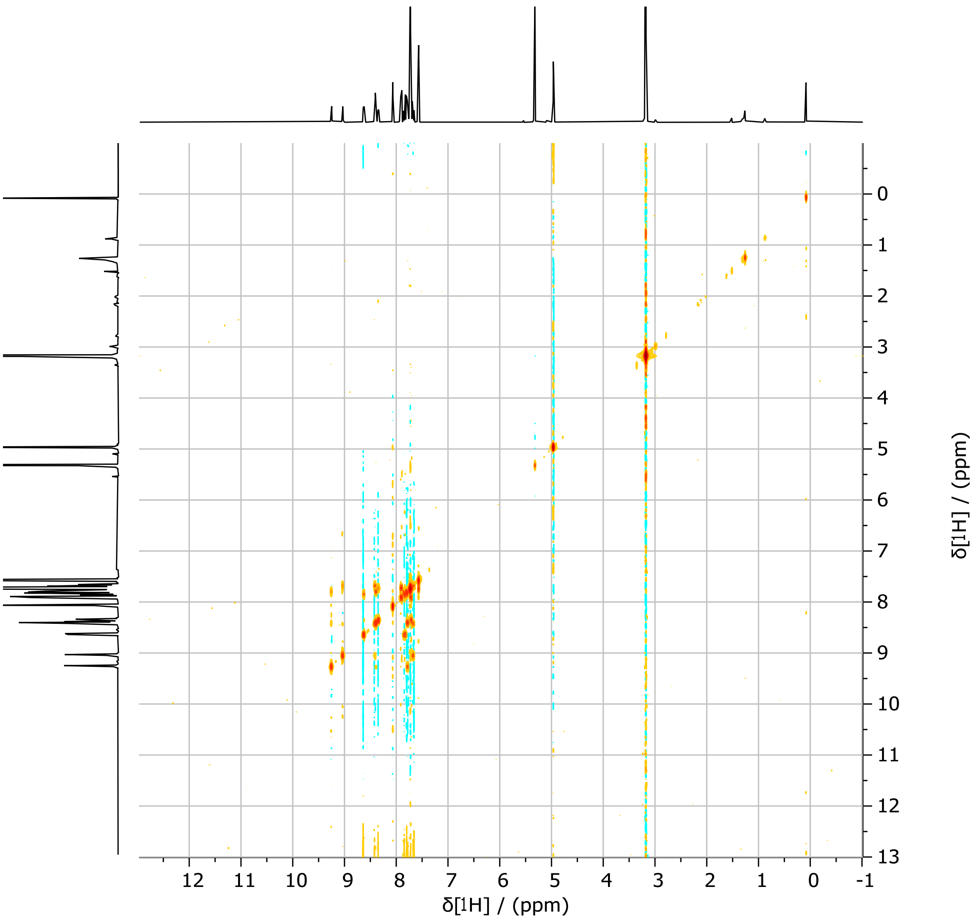

Figure SI-4-41: ^{1}H^{1}H COSY-NMR (400 MHz, 400 MHz) of compound [Pd(bhq)(bhq$^{5\text{-}CH_2NMe_3}$)]BArF, [**7**$^{+}$]$^{-}$BArF, in CD_2Cl_2 measured at r.t.

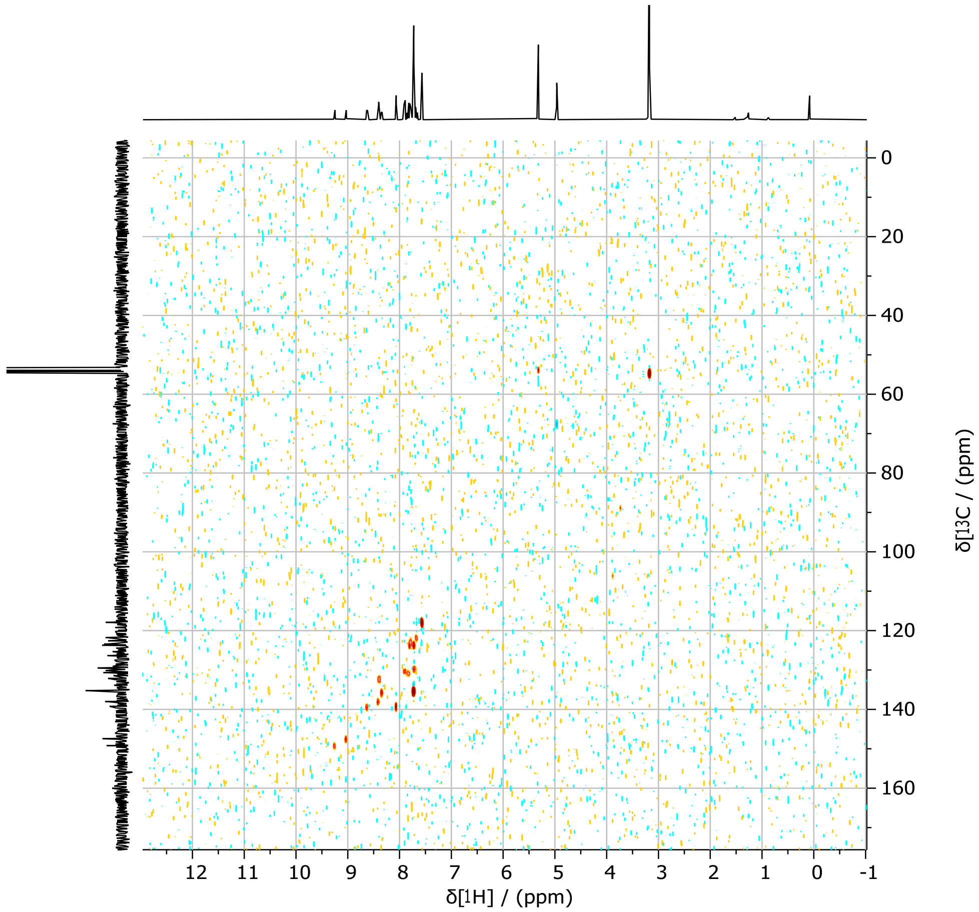

Figure SI-4-42: HSQC-NMR (400 MHz, 101 MHz) of compound [Pd(bhq)(bhq$^{5\text{-}CH2NMe3}$)]BArF, [**7**$^{+}$]$^{-}$BArF, in CD_2Cl_2 measured at r.t.

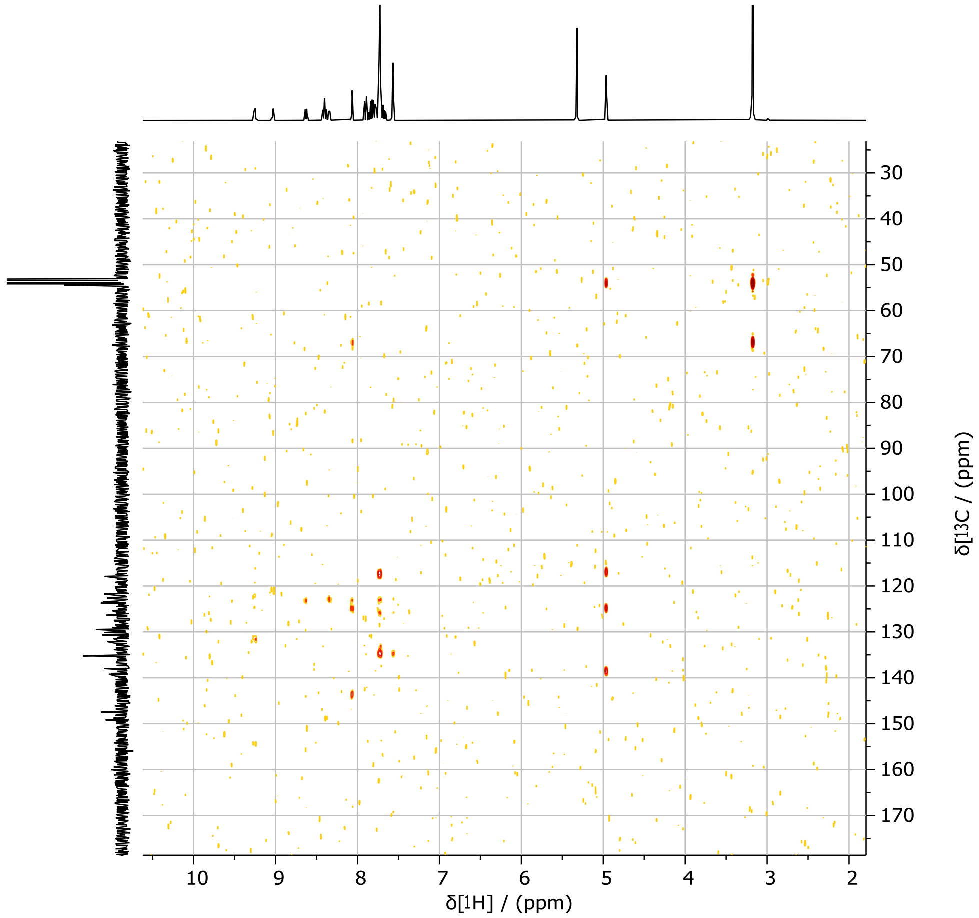

Figure SI-4-43: HMBC-NMR (400 MHz, 101 MHz) of compound [Pd(bhq)(bhq$^{5\text{-}CH2NMe3}$)]BArF, [**7**$^{+}$]$^{-}$BArF, in CD_2Cl_2 measured at r.t.

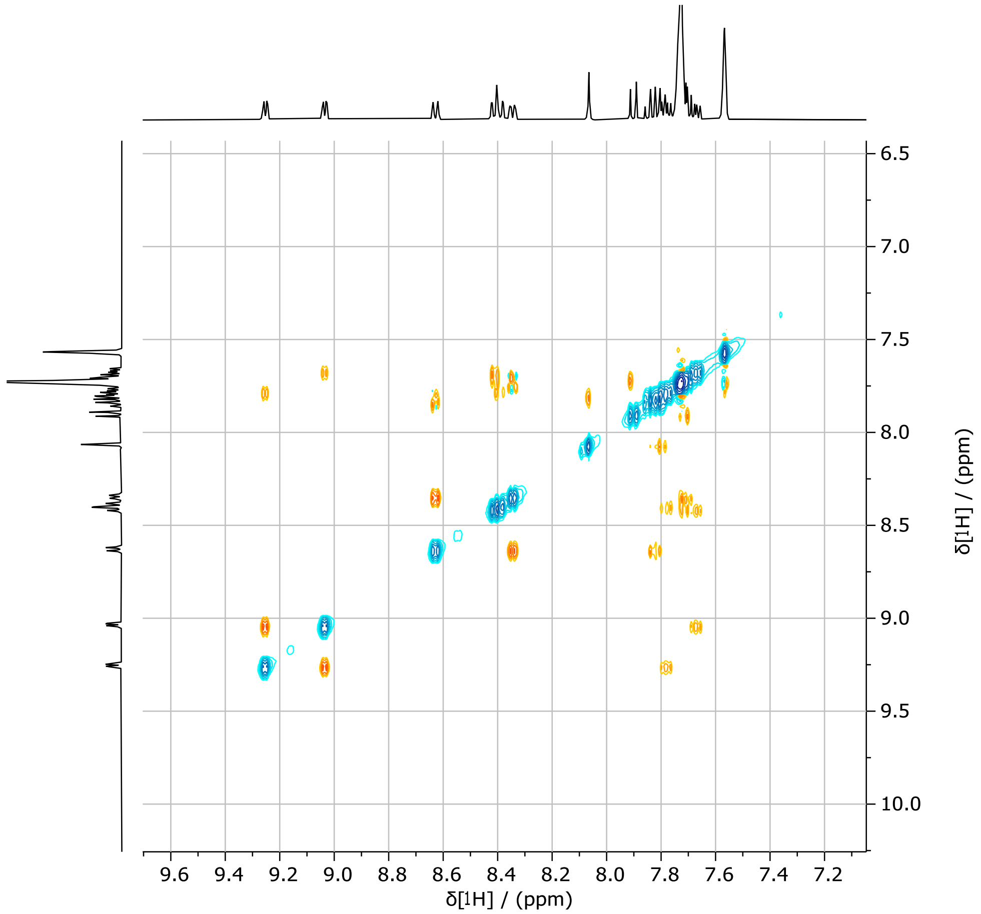

Figure SI-4-44: NOESY-NMR (400 MHz, 400 MHz) of compound [Pd(bhq)(bhq$^{5\text{-}CH2NMe3}$)]BArF, [7$^+$]$^-$BArF, in CD_2Cl_2 measured at r.t.

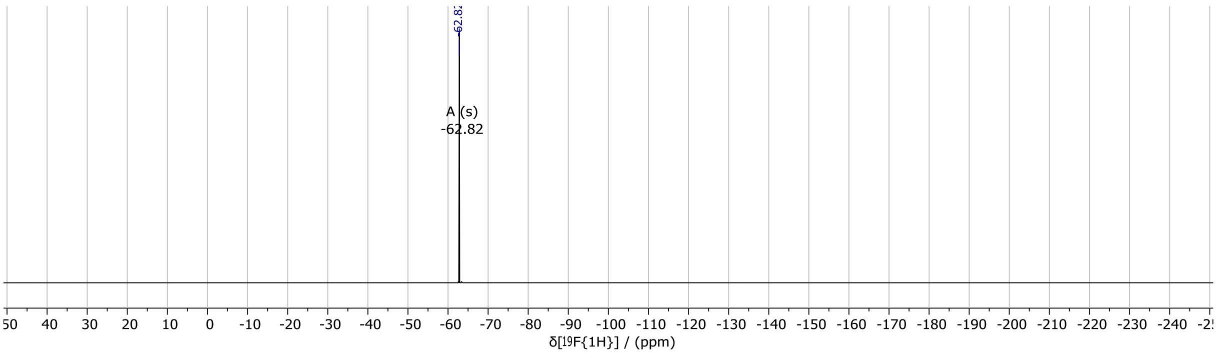

Figure SI-4-45: $^{19}F\{^1H\}$-NMR (377 MHz) spectrum of compound [Pd(bhq)(bhq$^{5\text{-}CH2NMe3}$)]BArF, [7$^+$]$^-$BArF, in CD_2Cl_2 measured at r.t.

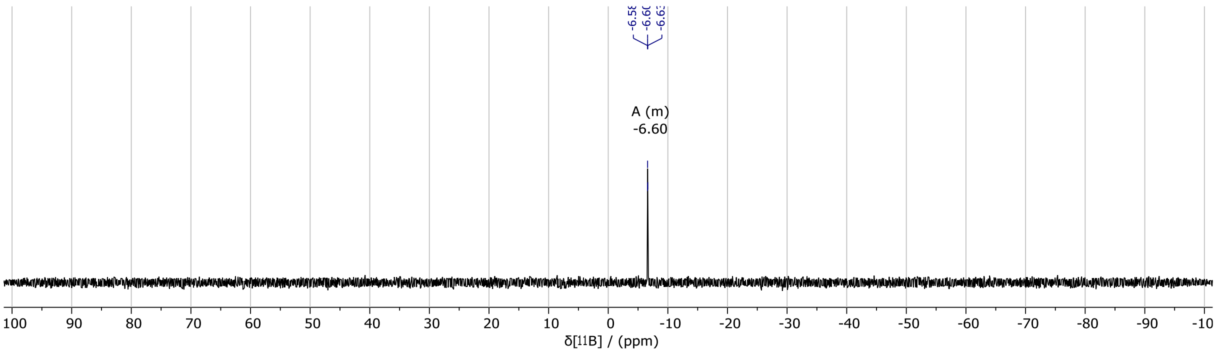

Figure SI-4-46: $^{11}B\{^1H\}$-NMR (128 MHz) spectrum of compound [Pd(bhq)(bhq$^{5\text{-}CH2NMe3}$)]BArF, [7$^+$]$^-$BArF, in CD_2Cl_2 measured at r.t.

4.2. NMR-Spectra of Organozinc Compounds

4.2.1. NMR Spectra of $Zn(Ar^{4F})_2$

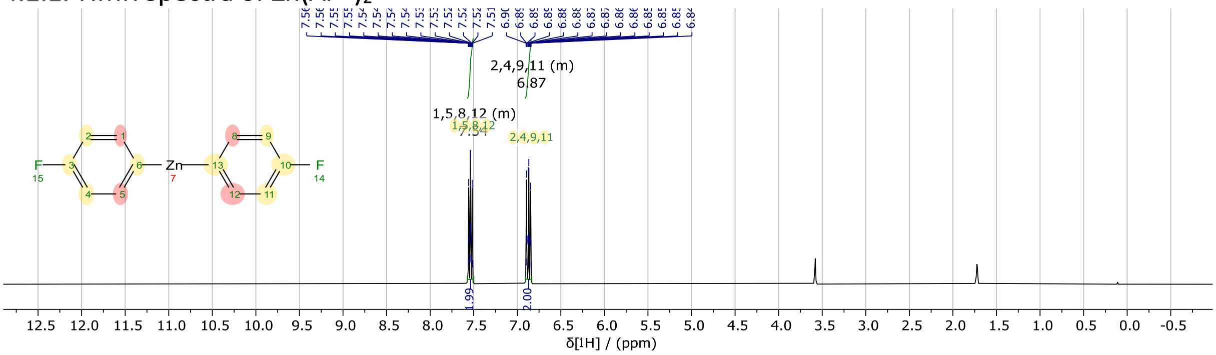

Figure SI-4-47: ^{1}H-NMR (400 MHz) spectrum of compound Bis(4-fluorophenyl)zinc, $Zn(Ar^{4F})_2$, in THF-d^8 measured at r.t. The spectrum was referenced to the residual solvent peak: ^{1}H-NMR (THF-d^8) δ = 3.58 ppm.

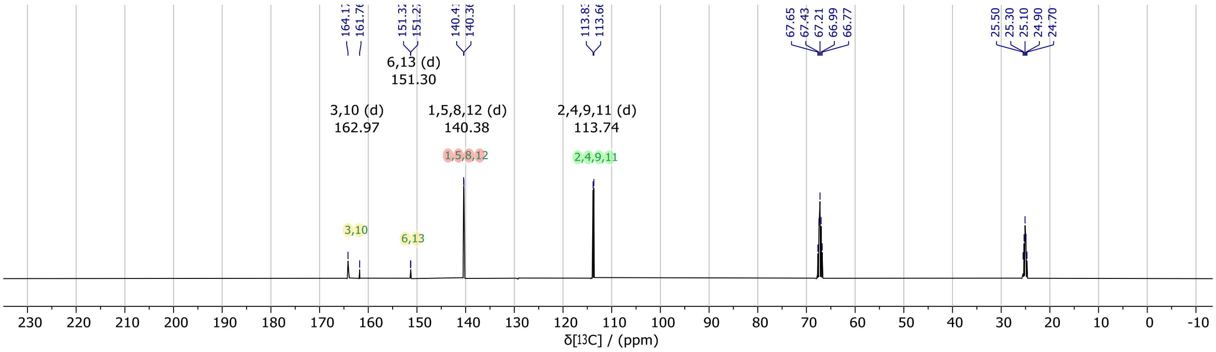

Figure SI-4-48: $^{13}C\{^{1}H\}$-NMR (101 MHz) spectrum of compound Bis(4-fluorophenyl)zinc, $Zn(Ar^{4F})_2$, in THF-**d**^{8}measured at r.t. The spectrum was referenced to the residual solvent peak: ^{13}C -NMR (THF-d^8) δ = 67.21 ppm.

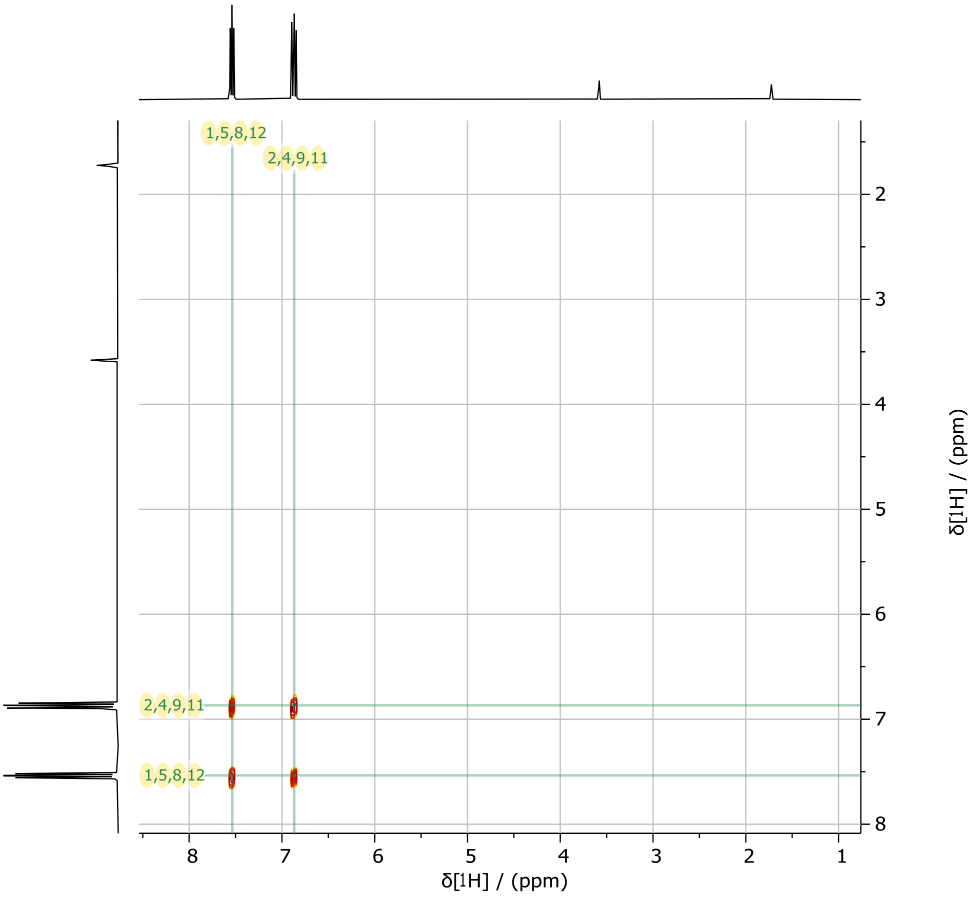

Figure SI-4-49: $^{1}H^{1}H$ COSY-NMR (400 MHz, 400 MHz) of compound Bis(4-fluorophenyl)zinc, $Zn(Ar^{4F})_2$, in THF-d^8 measured at r.t.

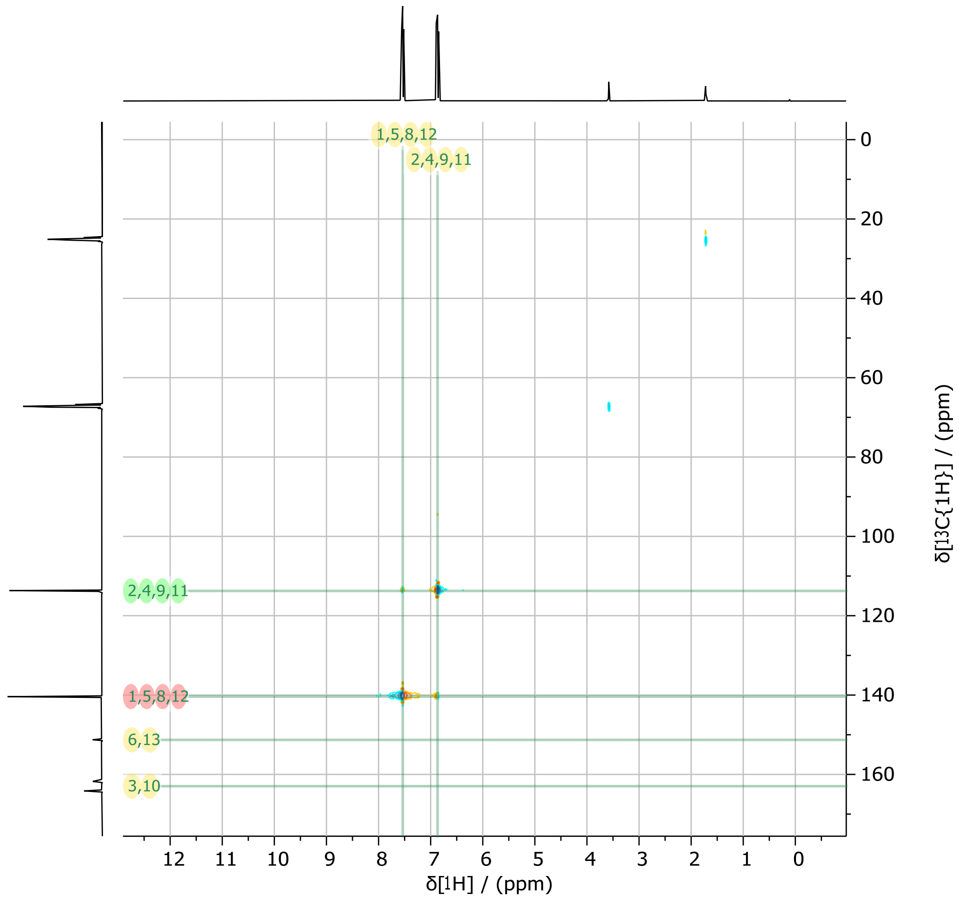

Figure SI-4-50: HSQC-NMR (400 MHz, 101 MHz) of compound Bis(4-fluorophenyl)zinc, Zn(Ar^{4F})$_2$, in THF-d[8] measured at r.t.

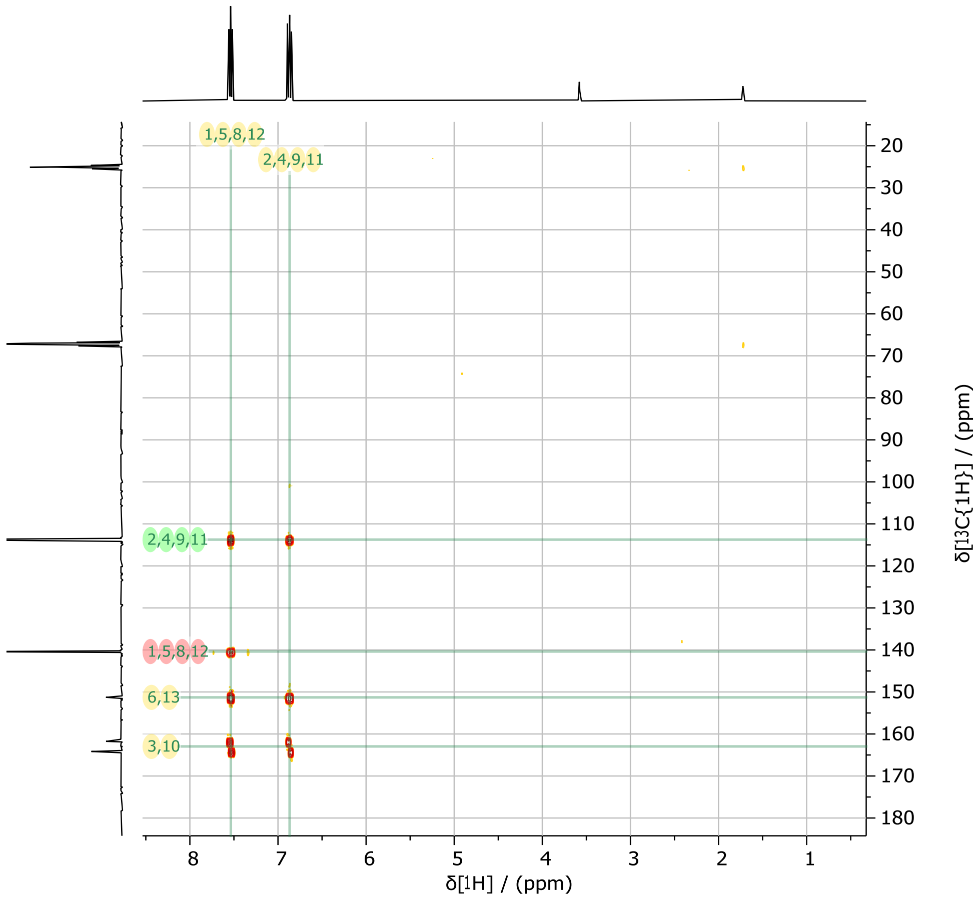

Figure SI-4-51: HMBC-NMR (400 MHz, 101 MHz) of compound Bis(4-fluorophenyl)zinc, Zn(Ar^{4F})$_2$, in THF-d[8] measured at r.t.

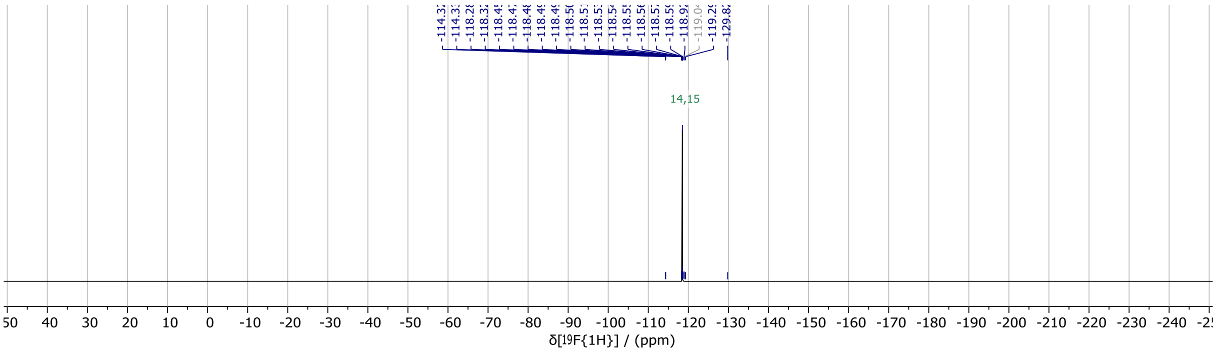

Figure SI-4-52: $^{19}F\{^1H\}$-NMR (377 MHz) spectrum of compound Bis(4-fluorophenyl)zinc, $Zn(Ar^{4F})_2$, in THF-d^8 measured at r.t.

4.2.2. NMR Spectra of $Zn(Ar^{2,6F})_2$

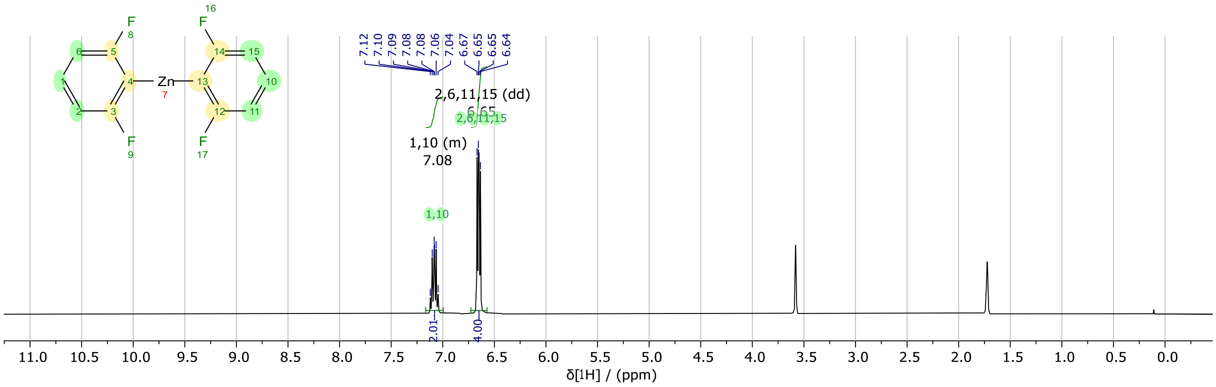

Figure SI-4-53: ^1H-NMR (400 MHz) spectrum of compound Bis(2,6-difluorophenyl)zinc, $Zn(Ar^{2,6F})_2$, in THF-d^8 measured at r.t. The spectrum was referenced to the residual solvent peak: ^1H-NMR (THF-d^8) δ = 3.58 ppm.

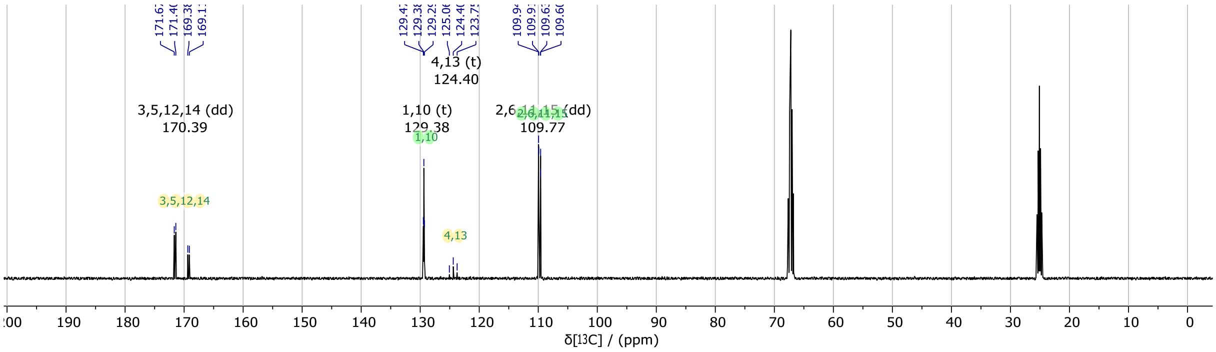

Figure SI-4-54: $^{13}C\{^1H\}$-NMR (101 MHz) spectrum of compound Bis(2,6-difluorophenyl)zinc, $Zn(Ar^{2,6F})_2$, in THF-d^8 measured at r.t. The spectrum was referenced to the residual solvent peak: ^{13}C -NMR (THF-d^8) δ = 67.21 ppm.

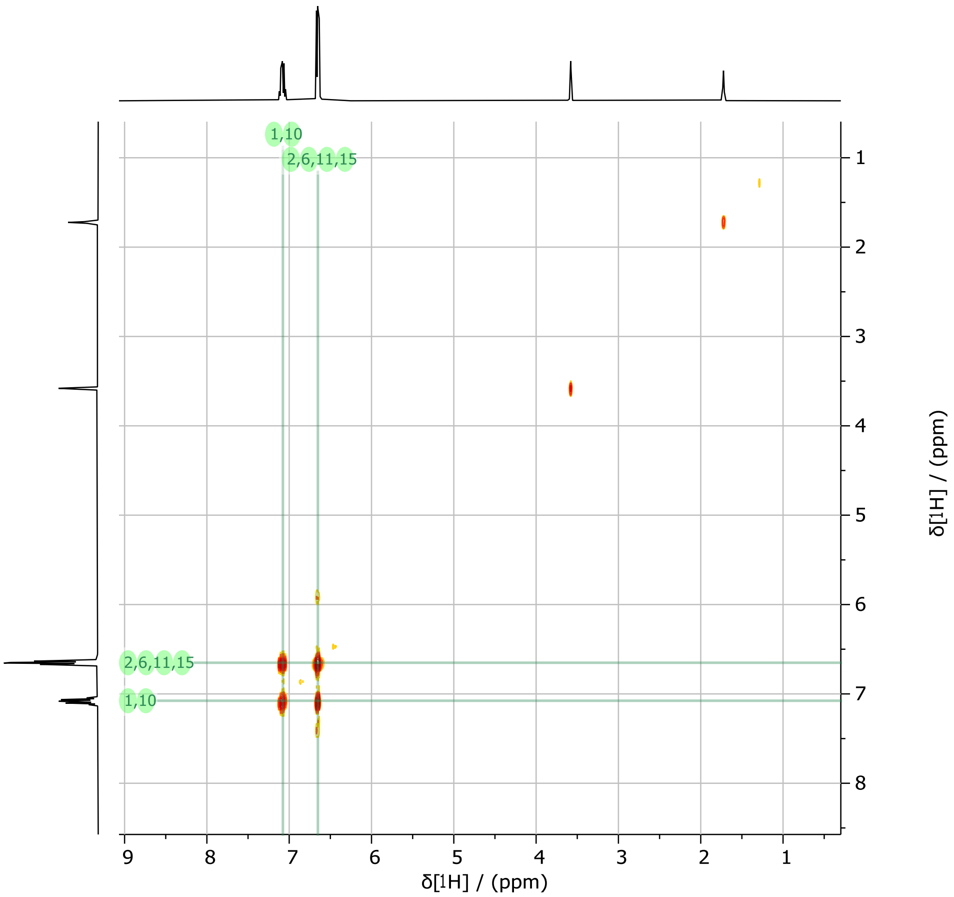

Figure SI-4-55: $^1H^1H$ COSY-NMR (400 MHz, 400 MHz) of compound Bis(2,6-difluorophenyl)zinc, Zn(Ar^{2,6F})$_2$, in THF-d^8 measured at r.t.

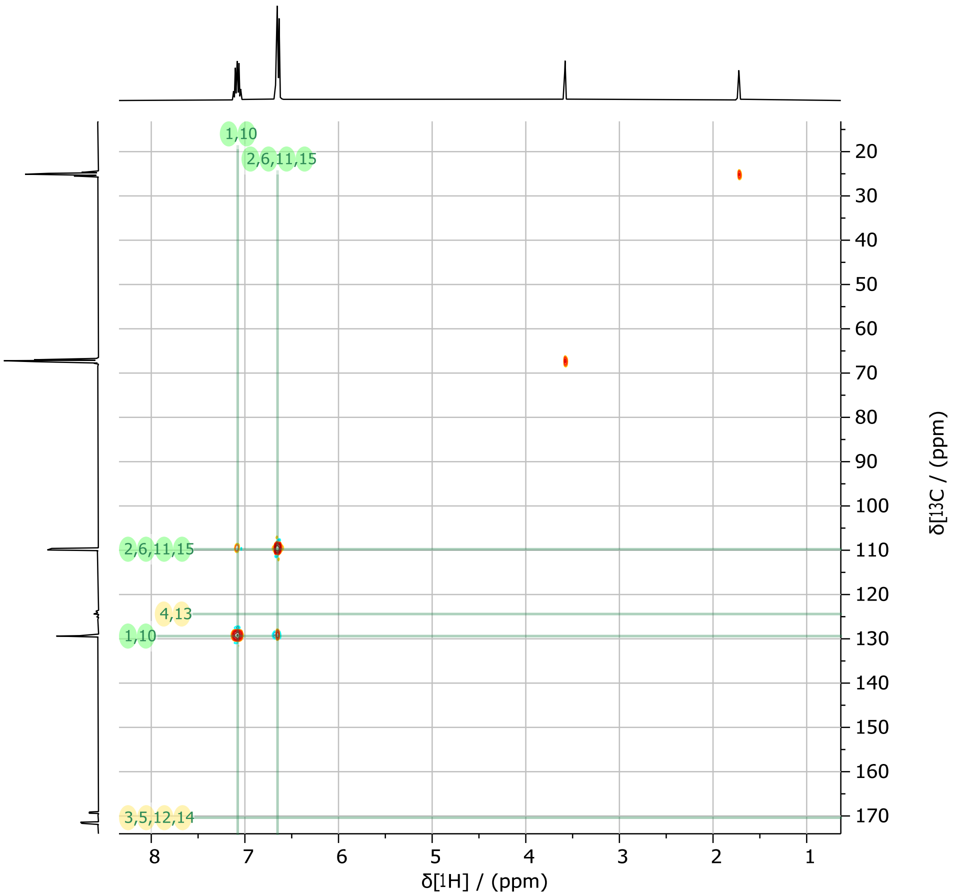

Figure SI-4-56: HSQC-NMR (400 MHz, 101 MHz) of compound Bis(2,6-difluorophenyl)zinc, Zn(Ar^{2,6F})$_2$, in THF-d^8 measured at r.t.

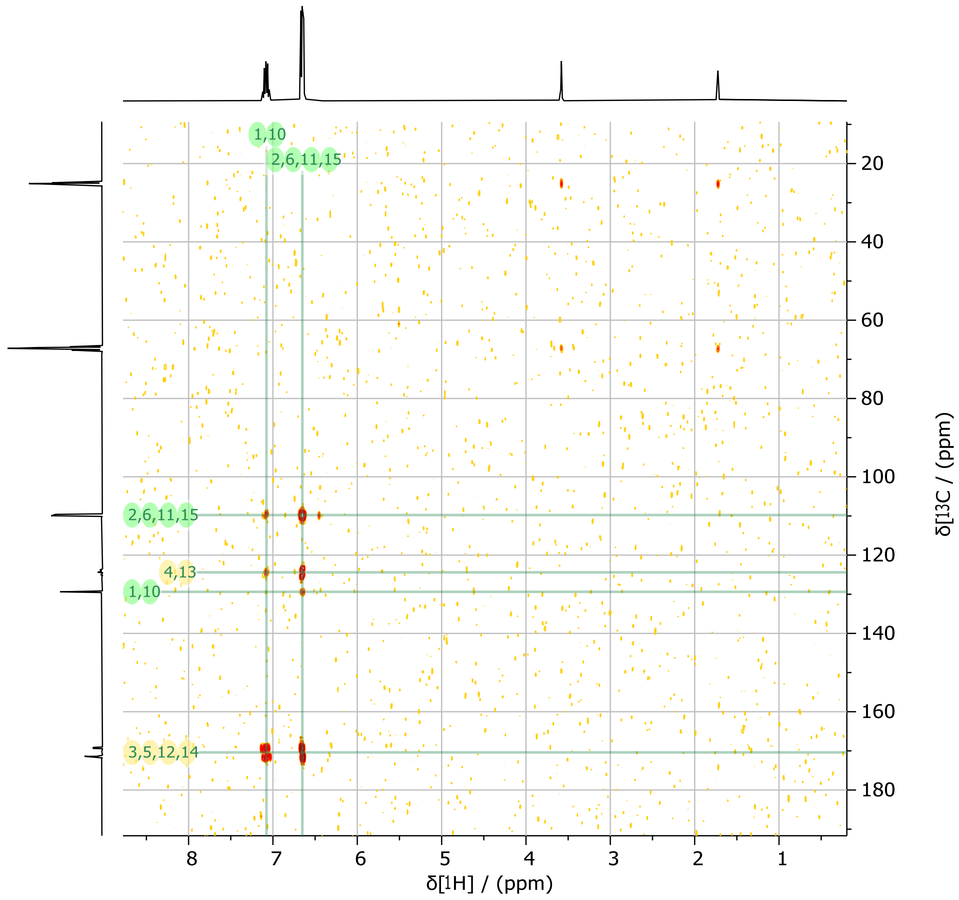

Figure SI-4-57: HMBC-NMR (400 MHz, 101 MHz) of compound Bis(2,6-difluorophenyl)zinc, Zn($Ar^{2,6F}$)$_2$, in THF-d^8 measured at r.t.

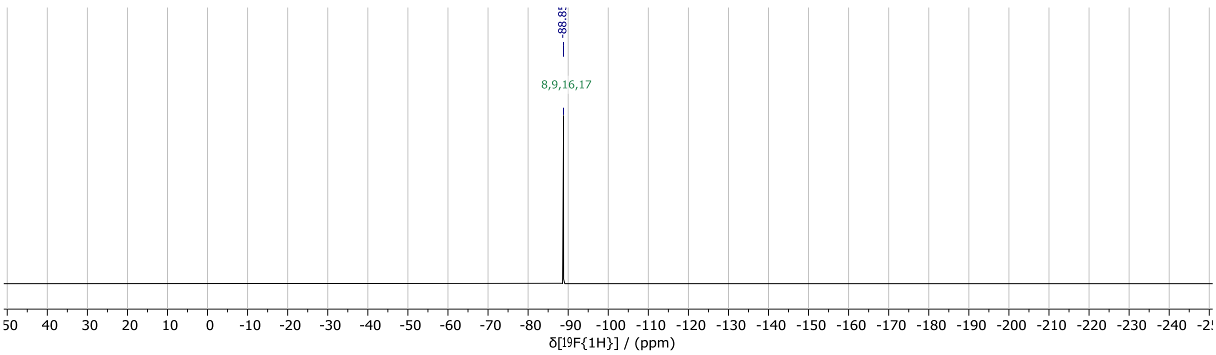

Figure SI-4-58: ^{19}F{^{1}H}-NMR (377 MHz) spectrum of compound Bis(2,6-difluorophenyl)zinc, Zn($Ar^{2,6F}$)$_2$, in THF-d^8 measured at r.t.

4.2.3. NMR Spectra of Zn($Ar^{2,4,6F}$)$_2$

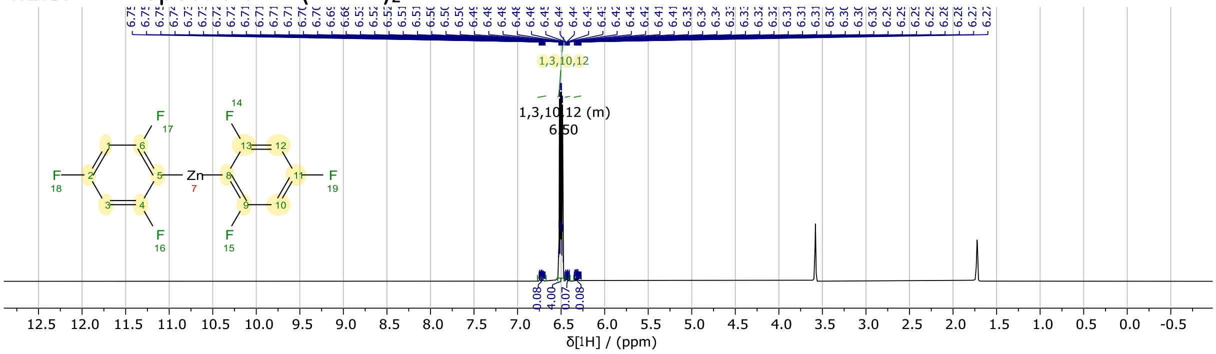

Figure SI-4-59: ^{1}H-NMR (400 MHz) spectrum of compound Bis(2,4,6-trifluorophenyl)zinc, Zn($Ar^{2,4,6F}$)$_2$, in THF-d^8 measured at r.t. The spectrum was referenced to the residual solvent peak: ^{1}H-NMR (THF-d^8) δ = 3.58 ppm.

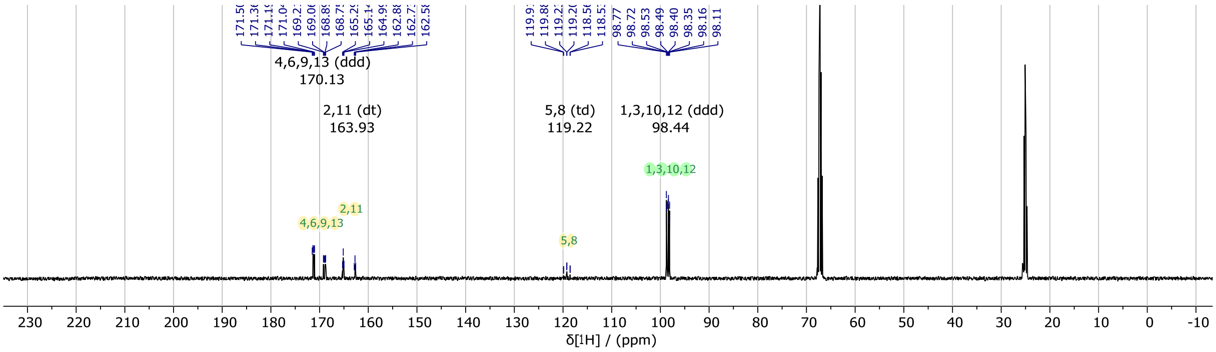

Figure SI-4-60: $^{13}C\{^{1}H\}$-NMR (101 MHz) spectrum of compound Bis(2,4,6-trifluorophenyl)zinc, $Zn(Ar^{2,4,6F})_2$, in THF-d8 measured at r.t. The spectrum was referenced to the residual solvent peak: ^{13}C -NMR (THF-d8) δ = 67.21 ppm.

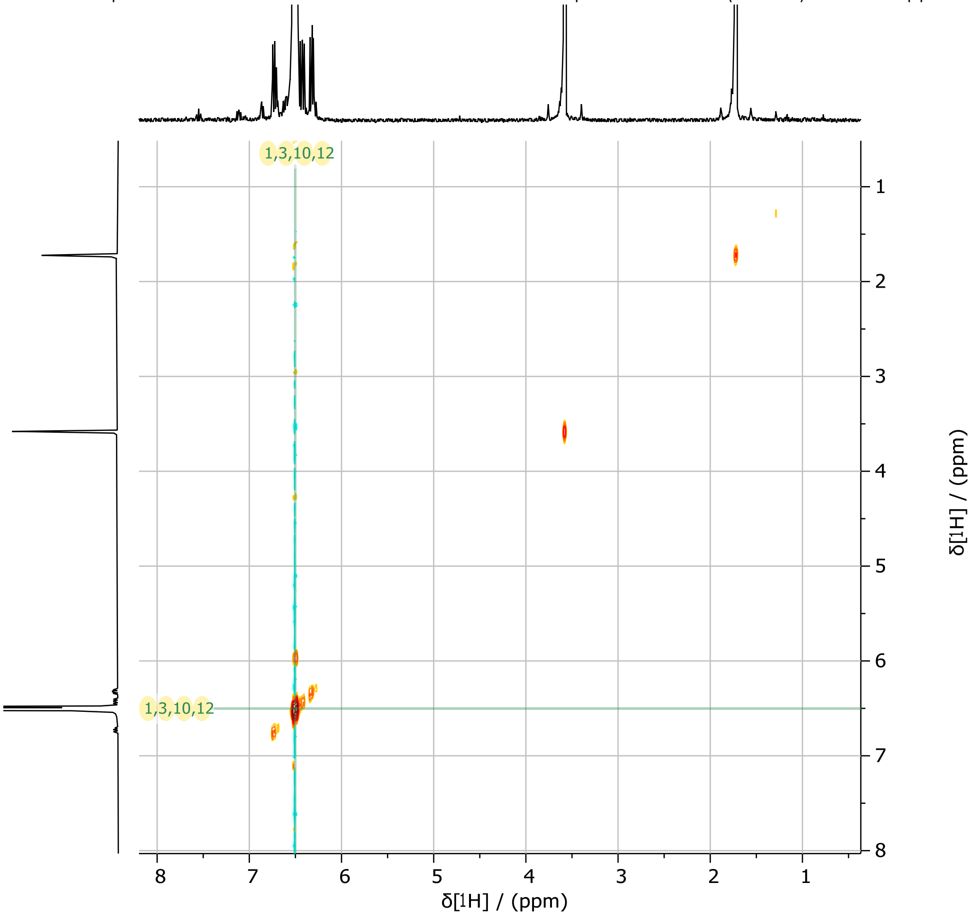

Figure SI-4-61: $^{1}H^{1}H$ COSY-NMR (400 MHz, 400 MHz) of compound Bis(2,4,6-trifluorophenyl)zinc, $Zn(Ar^{2,4,6F})_2$, in THF-d8 measured at r.t.

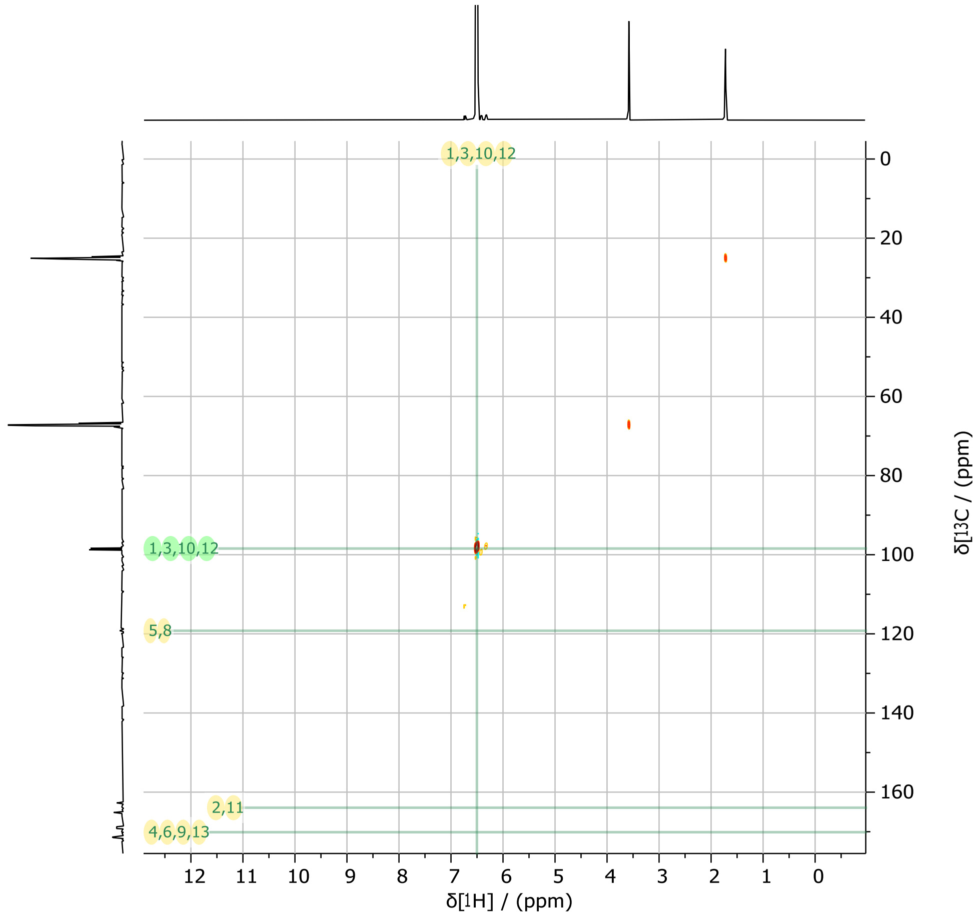

Figure SI-4-62: HSQC-NMR (400 MHz, 101 MHz) of compound Bis(2,4,6-trifluorophenyl)zinc, Zn($Ar^{2,4,6F}$)$_2$, in THF-d^8 measured at r.t.

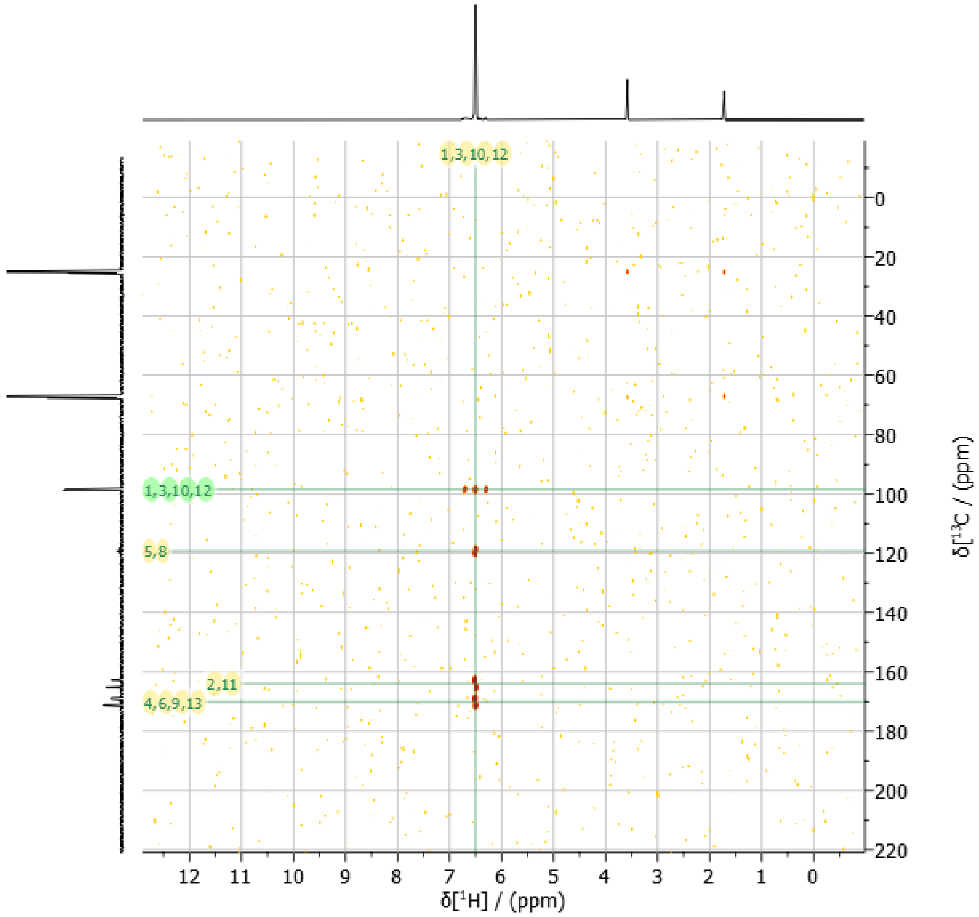

Figure SI-4-63: HMBC-NMR (400 MHz, 101 MHz) of compound Bis(2,4,6-trifluorophenyl)zinc, Zn($Ar^{2,4,6F}$)$_2$, in THF-d^8 measured at r.t.

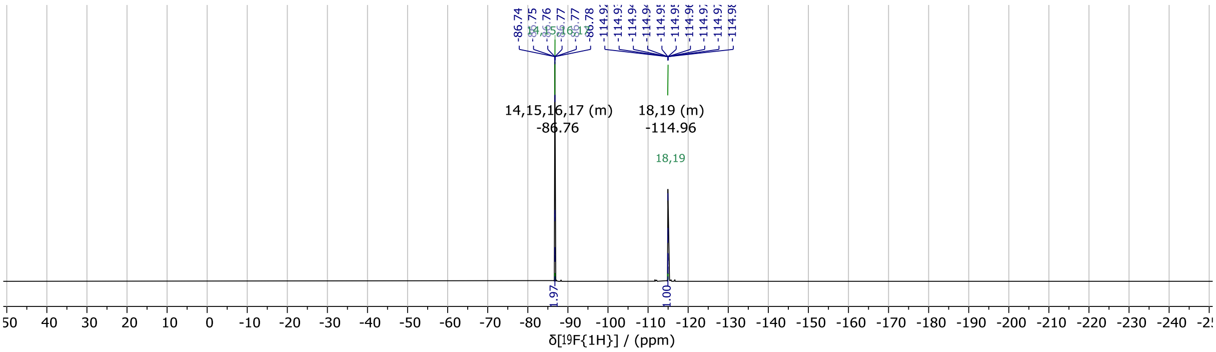

Figure SI-4-64: $^{19}F\{^{1}H\}$-NMR (377 MHz) spectrum of compound Bis(2,4,6-trifluorophenyl)zinc, $Zn(Ar^{2,4,6F})_2$, in THF-d[8] measured at r.t.

4.3. NMR-Spectra of Heterobimetallic Complexes

4.3.1. NMR Spectra of $[(bhq)_2Pd^{II}Ag^{I}(IPr)]BArF$, $[2^{+}]^{-}BArF$:

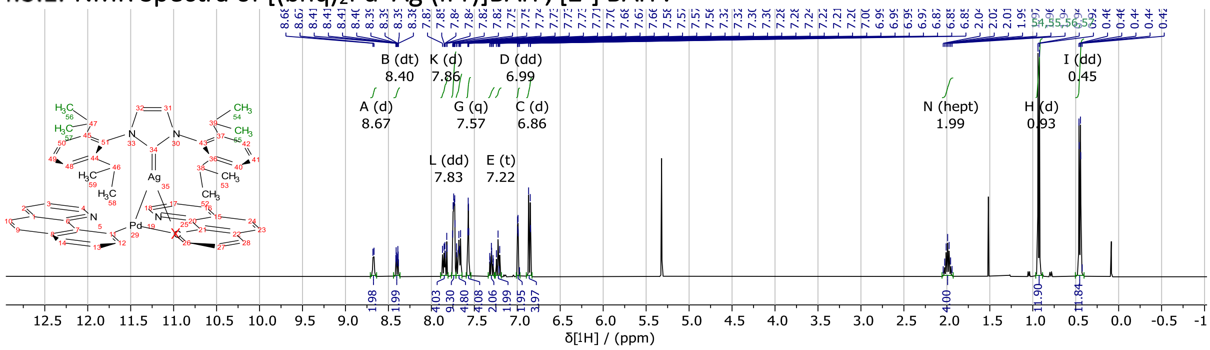

Figure SI-4-65: ^{1}H-NMR (400 MHz) spectrum of compound $[(bhq)_2Pd^{II}Ag^{I}(IPr)]BArF$, $[\mathbf{2}^{+}]^{-}BArF$, in CD_2Cl_2 measured at r.t. The spectrum was referenced to the residual solvent peak: ^{1}H-NMR (CD_2Cl_2) δ = 5.32 ppm.

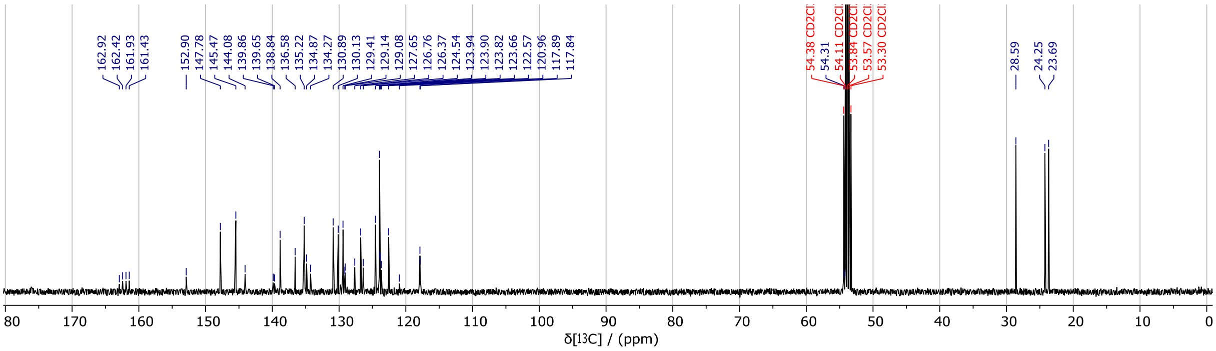

Figure SI-4-66: $^{13}C\{^{1}H\}$-NMR (101 MHz) spectrum of compound $[(bhq)_2Pd^{II}Ag^{I}(IPr)]BArF$, $[\mathbf{2}^{+}]^{-}BArF$, in CD_2Cl_2 measured at r.t. The spectrum was referenced to the residual solvent peak: ^{13}C -NMR (CD_2Cl_2) δ = 53.84 ppm.

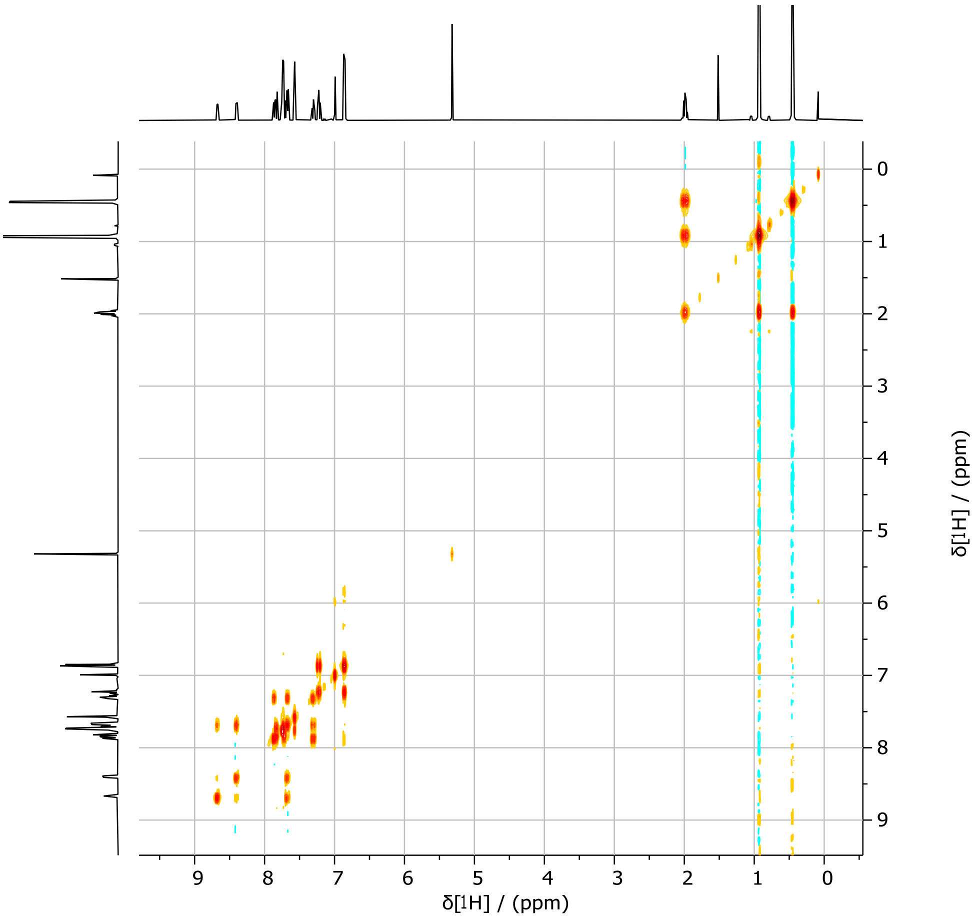

Figure SI-4-67: $^{1}H^{1}H$ COSY-NMR (400 MHz, 400 MHz) of compound [(bhq)$_2$Pd^{II}Ag^{I}(IPr)]BArF, [**2**$^{+}$]$^{-}$BArF, in CD_2Cl_2 measured at r.t.

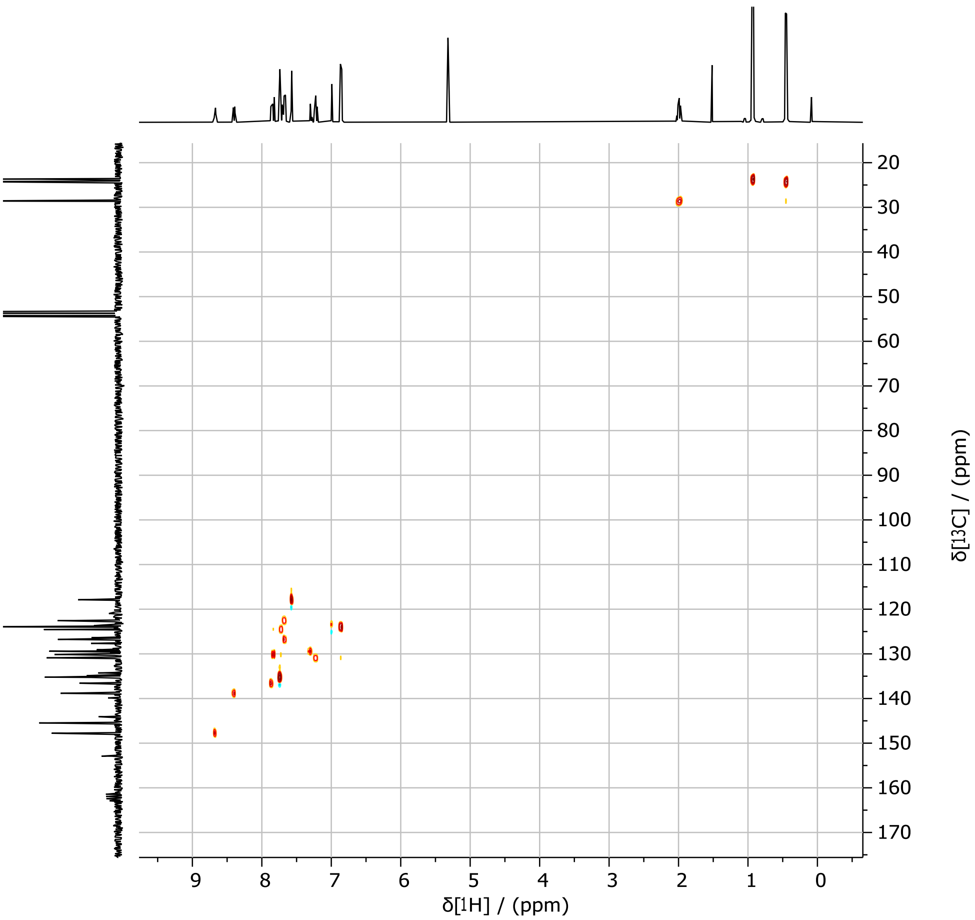

Figure SI-4-68: HSQC-NMR (400 MHz, 101 MHz) of compound [(bhq)$_2$Pd^{II}Ag^{I}(IPr)]BArF, [**2**$^{+}$]$^{-}$BArF, in CD_2Cl_2 measured at r.t.

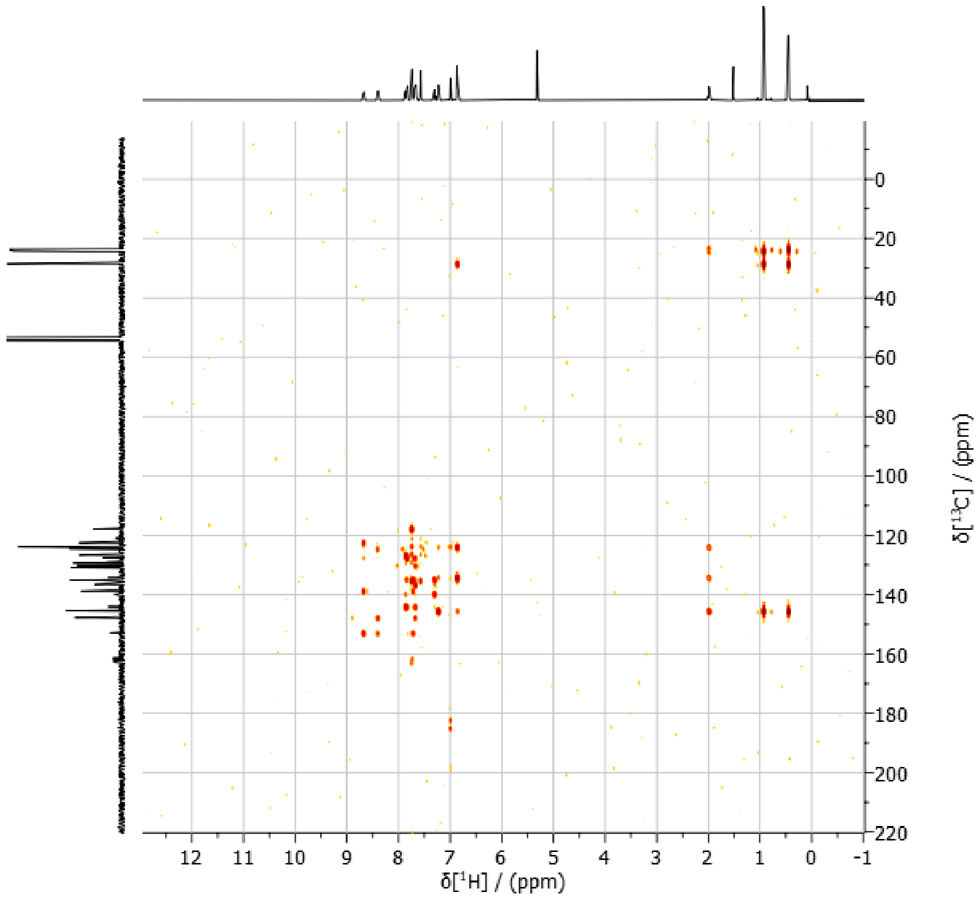

Figure SI-4-69: HMBC-NMR (400 MHz, 101 MHz) of compound [(bhq)$_2$Pd^{II}Ag^{I}(IPr)]BArF, [2$^+$]$^-$BArF, in CD_2Cl_2 measured at r.t.

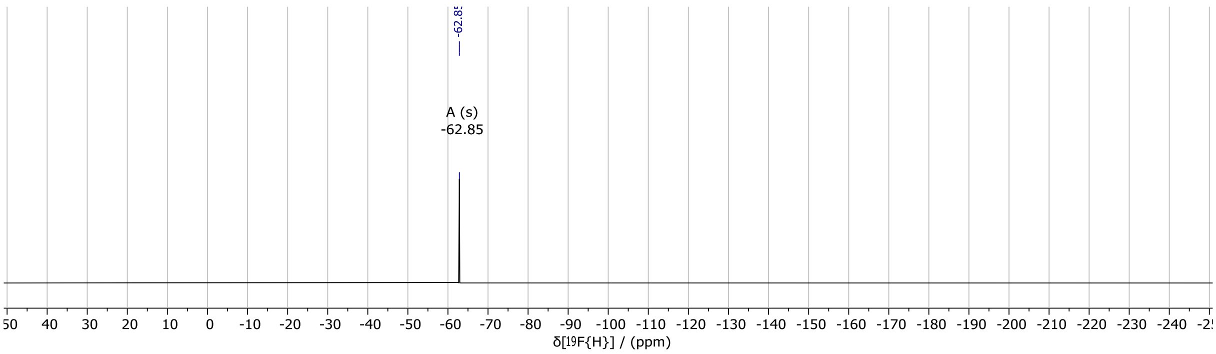

Figure SI-4-70: ^{19}F{^{1}H}-NMR (377 MHz) spectrum of compound [(bhq)$_2$Pd^{II}Ag^{I}(IPr)]BArF, [2$^+$]$^-$BArF, in CD_2Cl_2 measured at r.t.

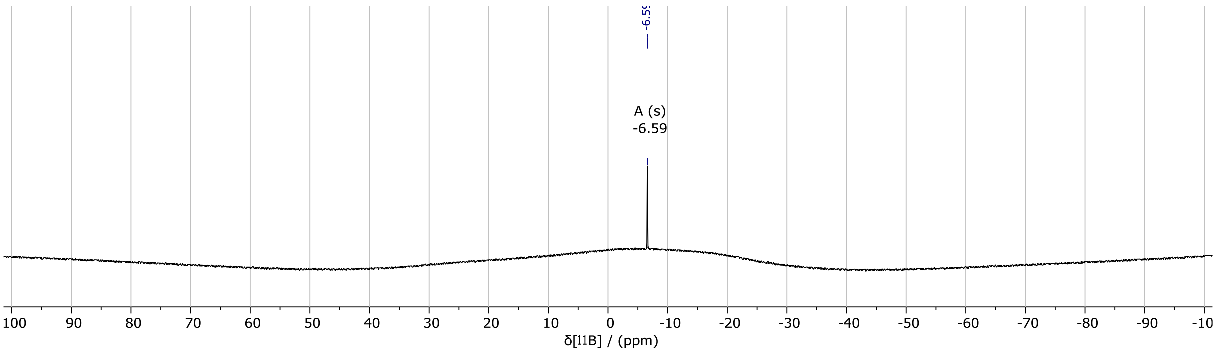

Figure SI-4-71: ^{11}B{^{1}H}-NMR (128 MHz) spectrum of compound [(bhq)$_2$Pd^{II}Ag^{I}(IPr)]BArF, [2$^+$]$^-$BArF, in CD_2Cl_2 measured at r.t.

4.3.2. NMR Spectra of [(bhq)$_2$Pd^{II}Au^{I}(IPr)]BArF, [3$^+$]$^-$BArF:

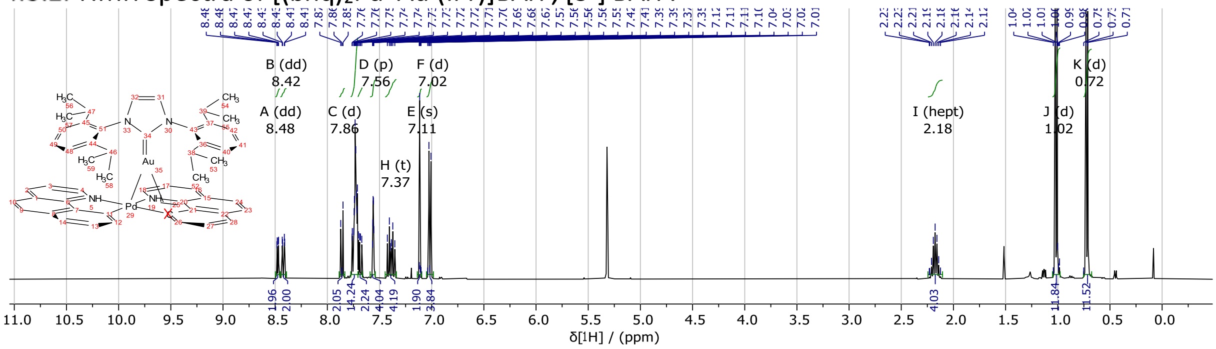

Figure SI-4-72: ^{1}H-NMR (400 MHz) spectrum of compound [(bhq)$_2$Pd^{II}Au^{I}(IPr)]BArF, [**3**$^+$]$^-$BArF, in CD_2Cl_2 measured at r.t. The spectrum was referenced to the residual solvent peak: ^{1}H-NMR (CD_2Cl_2) δ = 5.32 ppm.

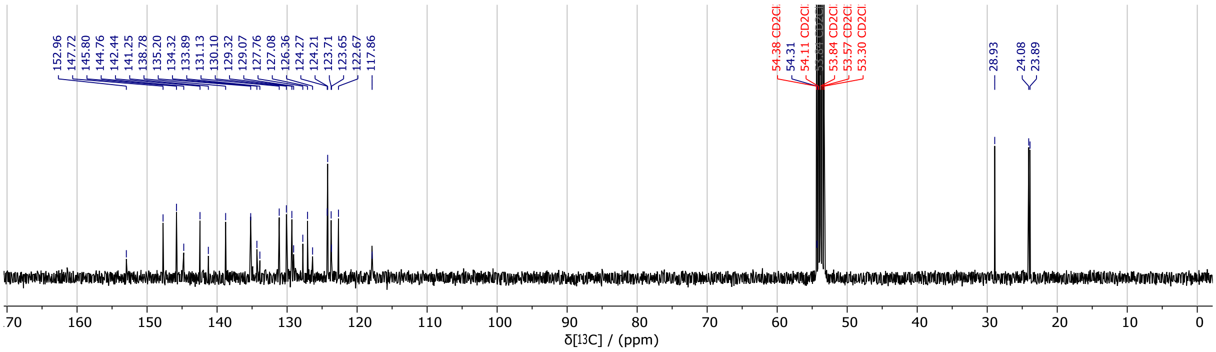

Figure SI-4-73: ^{13}C{^{1}H}-NMR (101 MHz) spectrum of compound [(bhq)$_2$Pd^{II}Au^{I}(IPr)]BArF, [**3**$^+$]$^-$BArF, in **CD_2Cl_2**measured at r.t. The spectrum was referenced to the residual solvent peak: ^{13}C -NMR (CD_2Cl_2) δ = 53.84 ppm.

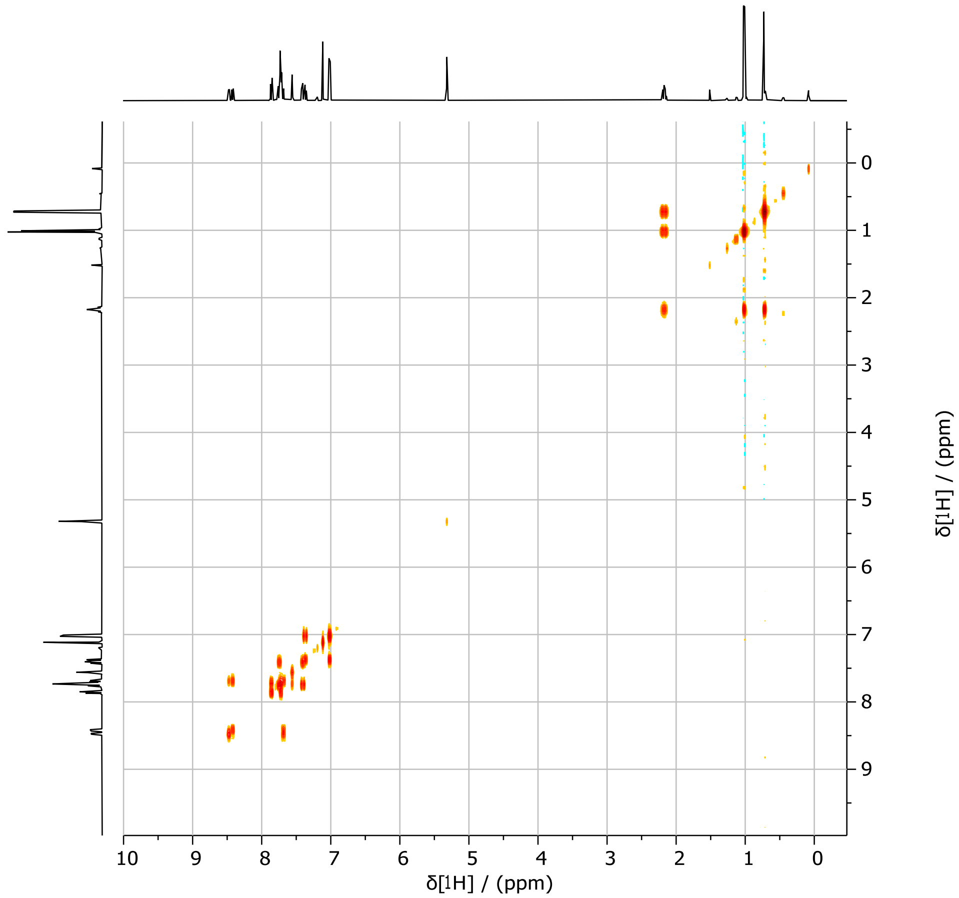

Figure SI-4-74: ^{1}H^{1}H COSY-NMR (400 MHz, 400 MHz) of compound [(bhq)$_2$Pd^{II}Au^{I}(IPr)]BArF, [**3**$^+$]$^-$BArF, in CD_2Cl_2 measured at r.t.

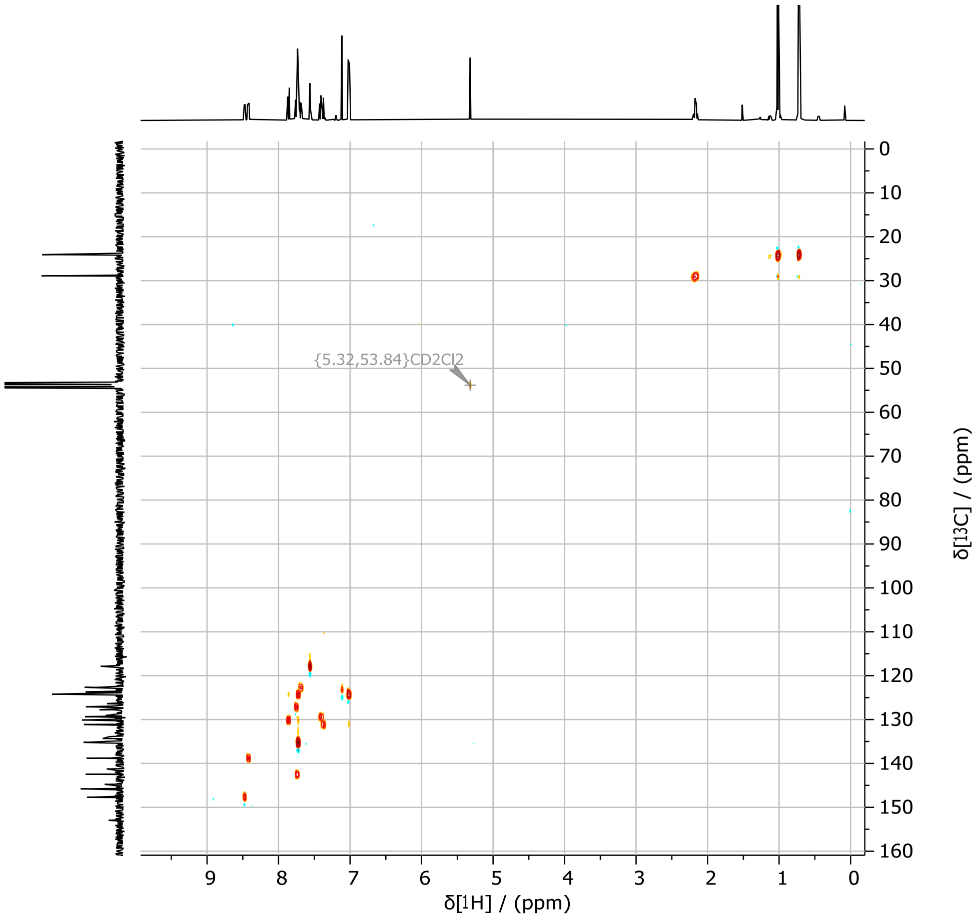

Figure SI-4-75: HSQC-NMR (400 MHz, 101 MHz) of compound [(bhq)$_2$Pd^{II}Au^{I}(IPr)]BArF, [**3**$^+$]$^-$BArF, in CD_2Cl_2 measured at r.t.

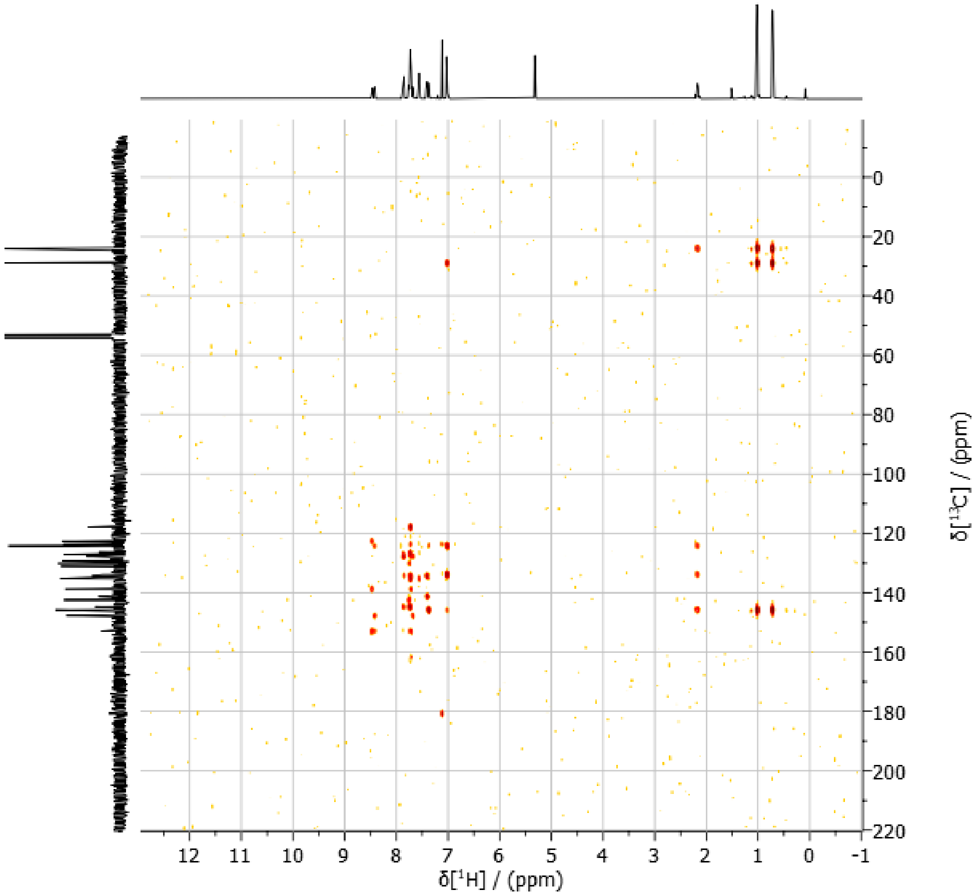

Figure SI-4-76: HMBC-NMR (400 MHz, 101 MHz) of compound [(bhq)$_2$Pd^{II}Au^{I}(IPr)]BArF, [**3**$^+$]$^-$BArF, in CD_2Cl_2 measured at r.t.

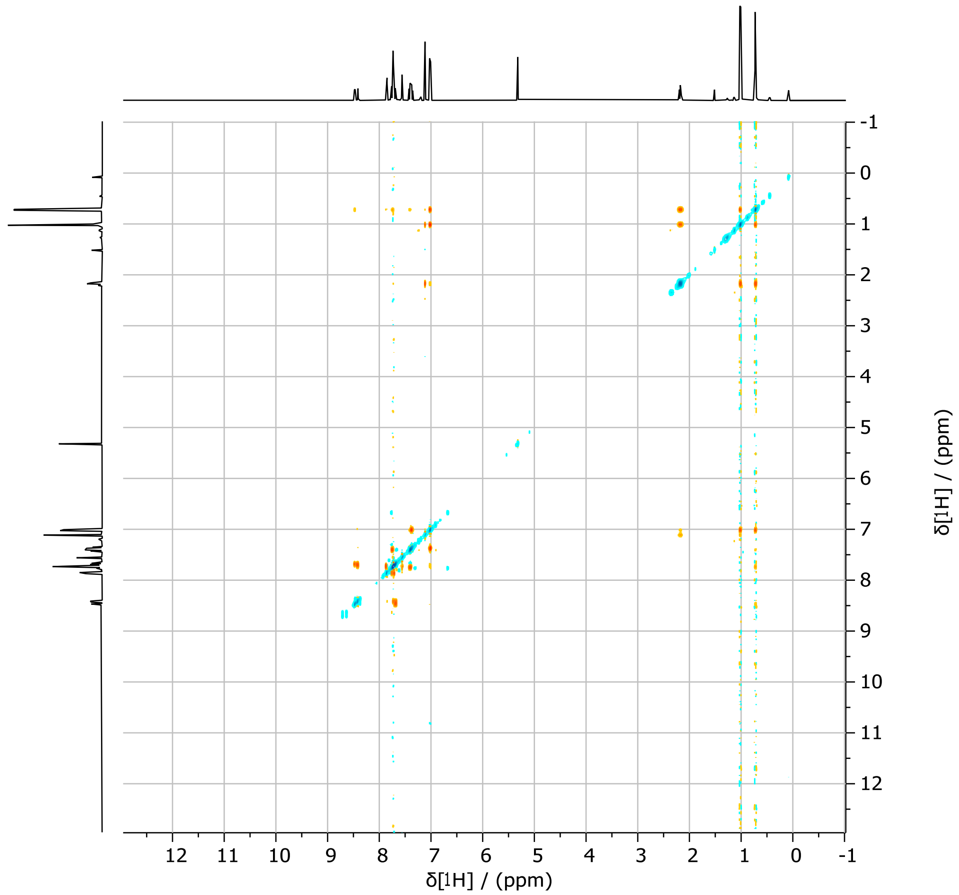

Figure SI-4-77: NOESY-NMR (400 MHz, 400 MHz) of compound [(bhq)$_2$Pd^{II}Au^{I}(IPr)]BArF, [**3**$^+$]$^-$BArF, in CD_2Cl_2 measured at r.t.

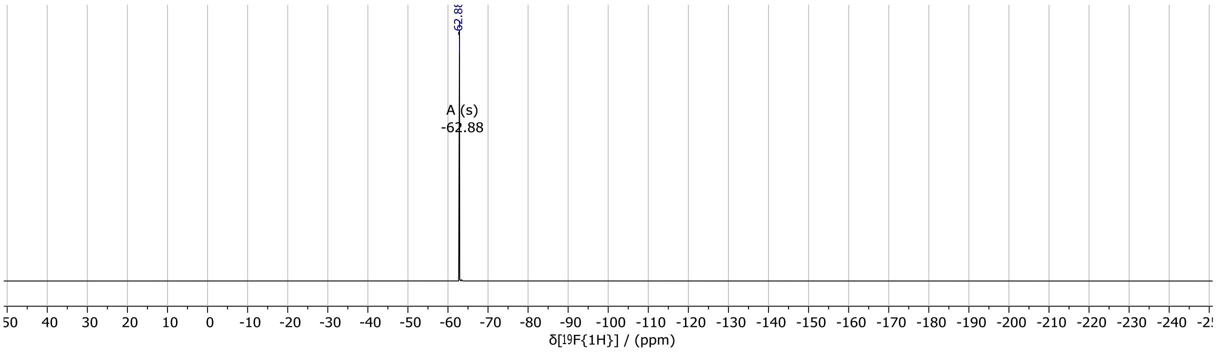

Figure SI-4-78: ^{19}F{^{1}H}-NMR (377 MHz) spectrum of compound [(bhq)$_2$Pd^{II}Au^{I}(IPr)]BArF, [**3**$^+$]$^-$BArF, in CD_2Cl_2 measured at r.t.

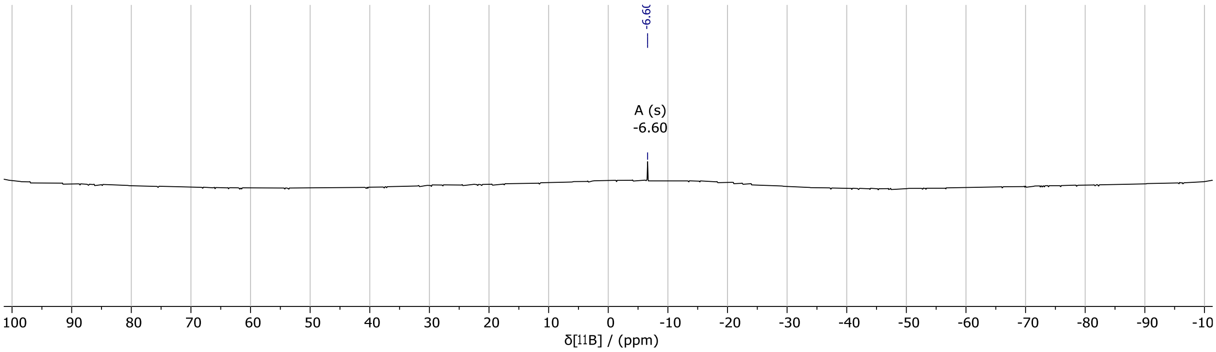

Figure SI-4-79: ^{11}B{^{1}H}-NMR (128 MHz) spectrum of compound [(bhq)$_2$Pd^{II}Au^{I}(IPr)]BArF, [**3**$^+$]$^-$BArF, in CD_2Cl_2 measured at r.t.

4.4. Processing ESI-MS/MS - T-CID / L-CID

For an overview see section 2.4. The following Figures show experimental characterization of the ion beam prior to the collision-induced dissociation. With regard to the kinetic energy distributions, specifically in the first panels of Figure SI-4-84 through SI-4-96, there are, in some instances, a second maximum at higher collision offsets (COFF). The Figures show the results from a retarding potential experiment in which the potential of the collision cell, in the absence of collision gas, is offset by a voltage, the COFF, which is then scanned to produce an ion transmission curve. The first derivative of the ion transmission curve should be the kinetic energy distribution. For an ideal ion guide, the ion transmission curve should be flat, followed by a sigmoidal rise to a flat plateau. The first derivative would then be a single Gaussian (or Gaussian-like) function. For a real ion guide, the ion transmission curves are more complicated. In constructing and optimizing the instrument, we experimented with different order multipoles, differently shaped multipoles, and multipoles of different dimension. Please refer to the dissertation of Marek Bot,[75] from which Figure SI-4-80 and SI-4-81 are taken. Moreover, we checked which adjustable parameters gave the best ion transmission curves. Below are sample ion transmission curves measured during that process, using the present octupole ion guide, with optimized operating parameters, compared to the curves for a (flat) quadrupole, starting with the same ions entering the multipole.

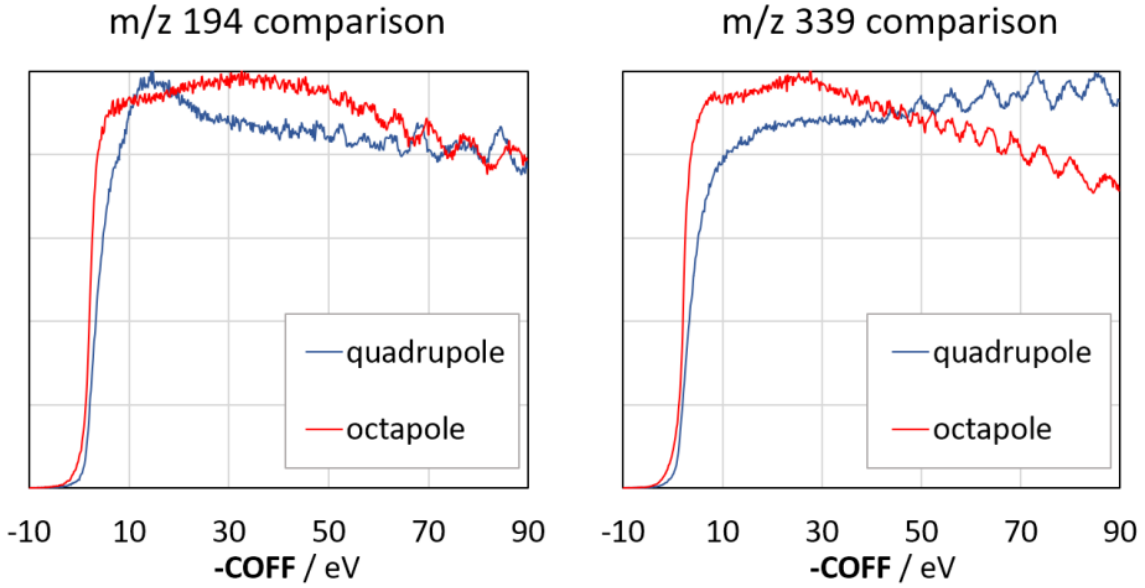

Figure SI-4-80: Representative ion transmission curves recorded for quadrupole (with flat poles) and octapole (with round poles) ion guides for a light test ion, m/z=194, and a heavier test ion, m/z=339 for ion guides with 2.75 mm inner diameter operating at 875 V rf amplitude at approximately 2.68 MHz (for the octapole). For further characterization of the effect of different geometric parameters, as well as different operating conditions, please refer to Marek Bot, Diss. ETH 26502, 2019.

The ion transmission curves show non-idealities, with the worst non-ideality being the so-called ion noding at higher COFF values, which are most easily explained with numerical simulations. Figure SI-4-81 shows a photograph of the actual octupole used in the present work, and a numerical simulation of a bundle of ion trajectories through the octupole, done with the program SIMION.

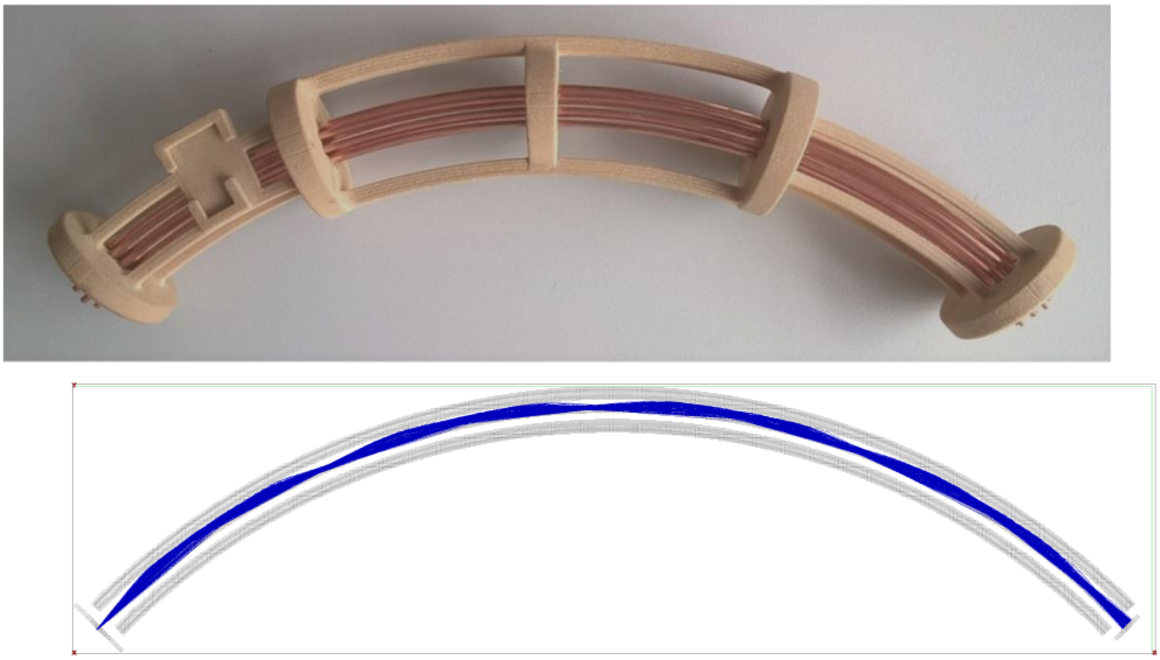

Figure SI-4-81: Photograph of the curved octupole used in the present work, and a numerical simulation of a packet of ion trajectories, at 75 eV kinetic energy, 875 V rf amplitude, and approximately 2.68 MHz. The bundle of ion trajectories all start at the left, at a single point, and exit the octupole at the orifice on the right. Further simulations may be found in the dissertation of Marek Bot, Diss. ETH 26502, 2019.

The simulation displays the result for a rather high COFF to show the effect clearly, i.e. a worst case. The ions fill a significant fraction of the volume in the octupole, and the bundle of trajectories shows nodes, which will move longitudinally in position as the total kinetic energy is varied. Looking to the right-hand side of the simulation, one sees that the radial extent of the bundle, which started at a point on the left-hand side, can converge to a point, or diverge to a bulge, depending on the kinetic energy. A finite orifice at the right-hand exit, or the poles themselves, can clip a part of the ion beam, giving rise to a periodic modulation of the ion transmission intensity as a function of COFF. The noding may be suppressed, or, at least, its onset may be pushed out to higher COFF, by increasing the RF amplitude. We find that the best results are always produced by setting the Q2 amplitude to the maximum, 825 V, that the RF generator can produce. Higher voltages also lead to breakdown, i.e. arcing, so this is a practical limit. Increasing the diameter also does not help, as the same electric field requires again a higher absolute RF amplitude. While not all non-idealities are related to noding, we show this example to indicate that the secondary bumps in the kinetic energy distribution are NOT due to differences in the actual, physical kinetic energy distribution of the ions, but rather limitations of the ion optics and electronics. Consider simply that the ions enter the quadupole and octupole, Figure SI-4-81, with the same kinetic energy distribution, but the non-idealities are different, so the derivatives of the ion transmission curves will show different bumps at higher COFF, which can then definitely be classified as artifacts.

Turning to the practical consequences of the non-ideal RF ion guides, we do point out that the diagnostics do give us independent measures of when the T-CID curves are negatively impacted by artifacts. We typically watch out for the artifacts, and we "cut" the T-CID curve at a point where they become a problem. A "cut" means that we fit fewer data points, but, first of all, we are fitting a very large data set, even with a "cut," and secondly, the value for the BDE delivered by L-CID is most sensitive to the part of the T-CID curve close to threshold. The part of the reactive cross-section above the "cut" simply matters less anyways. In Figure SI-4-82 through SI-4-96, "cuts" are already indicated by a red line in the lower, left-hand panel of each Figure. In each case, one may see that the non-idealities at higher COFF values are excluded from the data that will be fit for the BDE.

Lastly, we measure the kinetic energy distribution, KED, before and after each T-CID measurement. The kinetic energy distributions in the Figures are the summation of all of the measurements.

4.4.1. T-CID / L-CID data for the Measurements of [(bhq)$_2$Pd-Ag(IPr)]$^+$, [2$^+$]

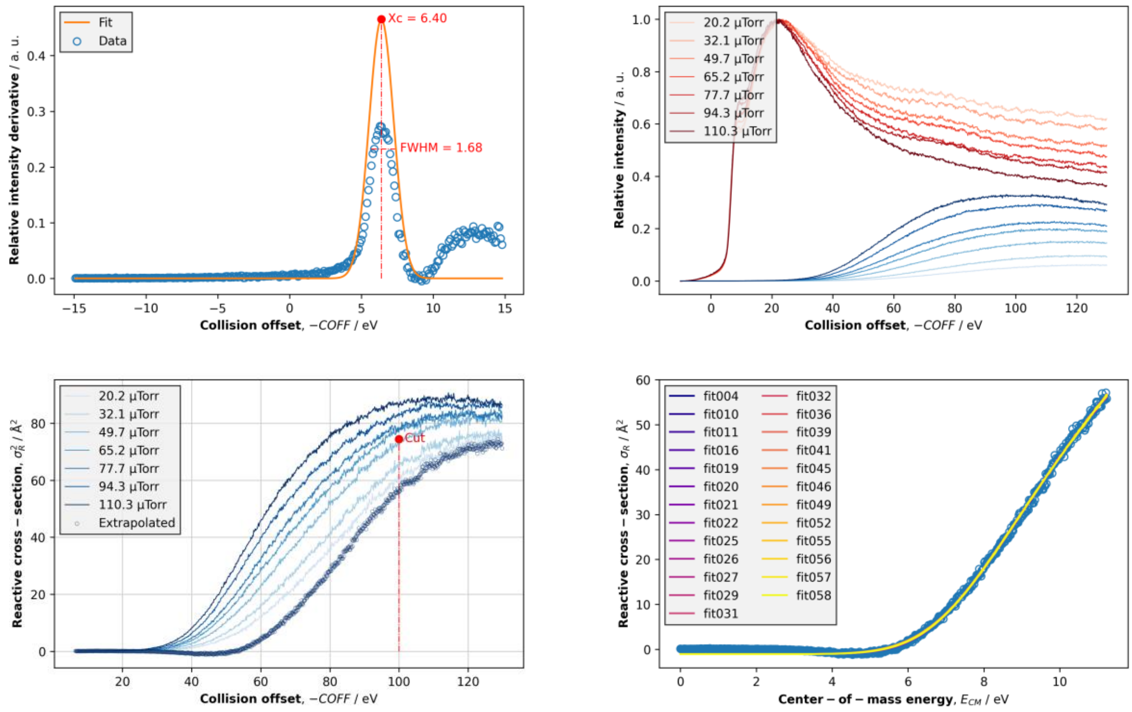

Figure SI-4-82: Experimental results (top-left) kinetic energy distribution, (top-right) intensity plot, (bottom-lef) zero collision extrapolation, (bottom-left) zero collision extrapolated and fits obtaind from L-CID.

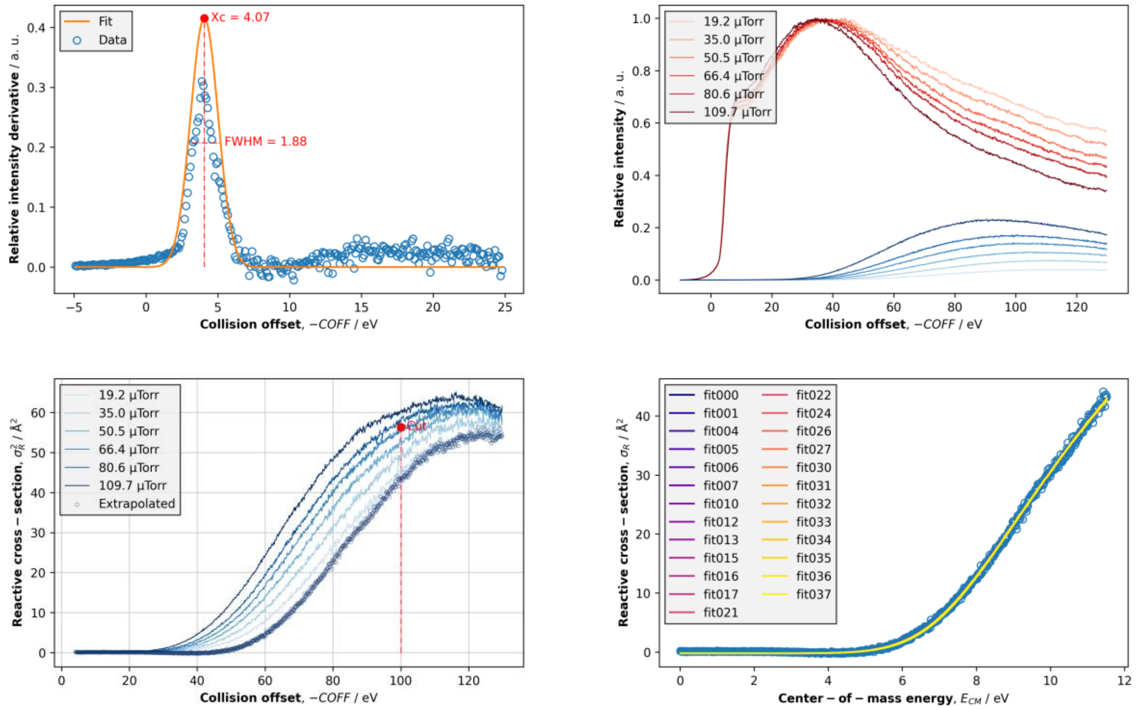

Figure SI-4-83: Experimental results (top-left) kinetic energy distribution, (top-right) intensity plot, (bottom-lef) zero collision extrapolation, (bottom-left) zero collision extrapolated and fits obtaind from L-CID.

4.4.2. T-CID / L-CID data for the Measurements of $[(bhq)_2Pd\text{-}Au(IPr)]^+$, $[3^+]$

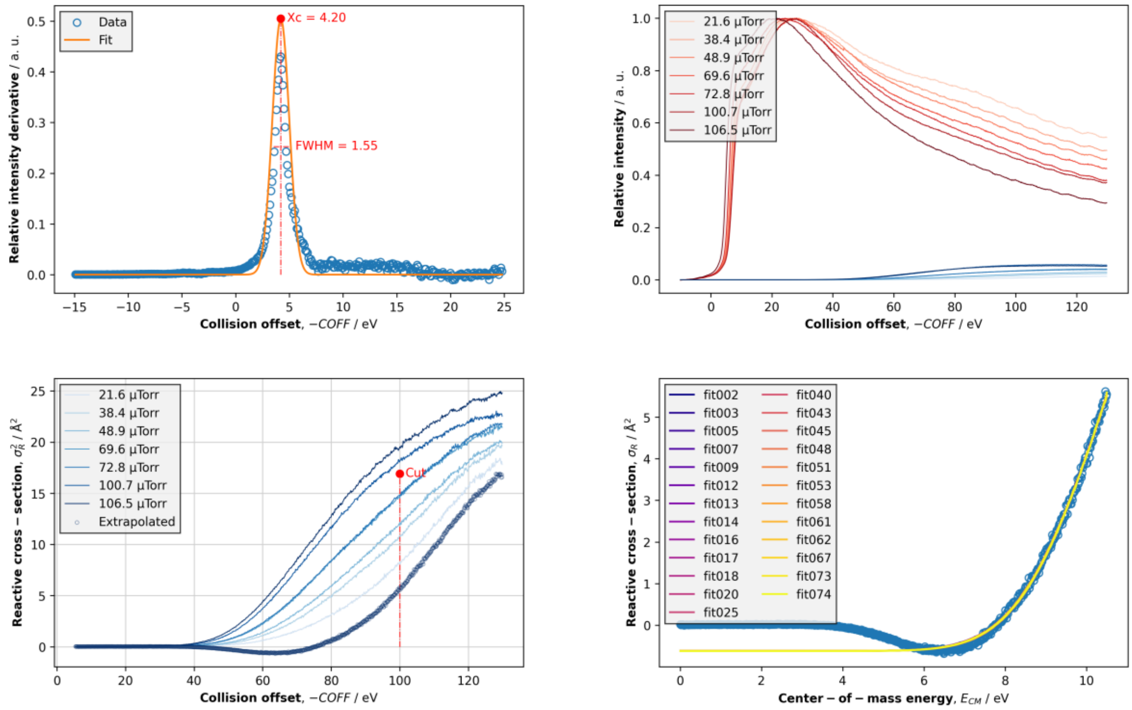

Figure SI-4-84: Experimental results (top-left) kinetic energy distribution, (top-right) intensity plot, (bottom-lef) zero collision extrapolation, (bottom-left) zero collision extrapolated and fits obtaind from L-CID.

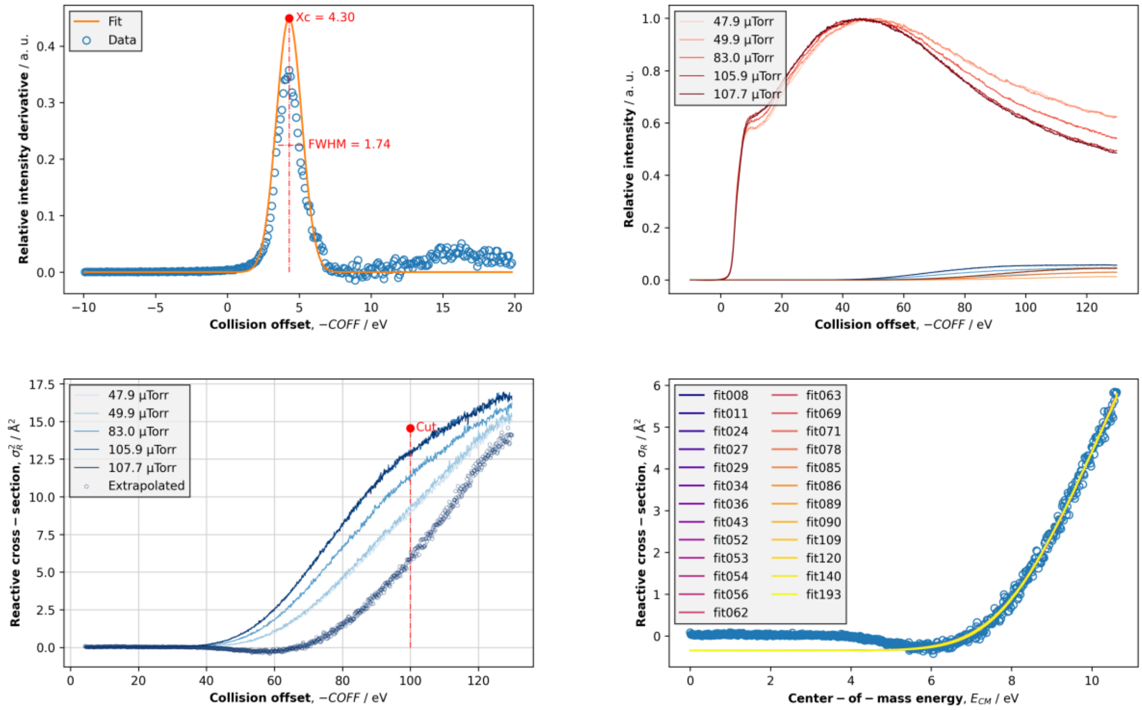

Figure SI-4-85: Experimental results (top-left) kinetic energy distribution, (top-right) intensity plot, (bottom-lef) zero collision extrapolation, (bottom-left) zero collision extrapolated and fits obtaind from L-CID.

4.4.3. T-CID / L-CID data for the Measurements of [(bhq)(bhq$^{5\text{-}CH_2NMe_3}$)Pd-Zn(C_6F_5)$_2$]$^+$, [4$^+$]

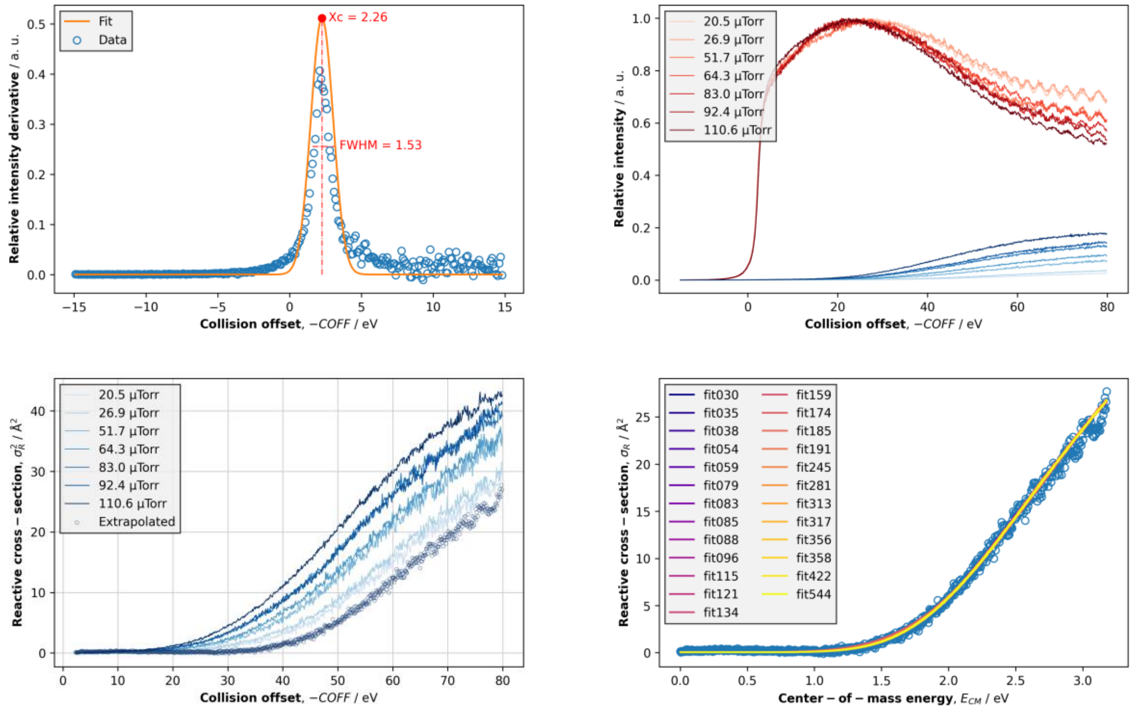

Figure SI-4-86: Experimental results (top-left) kinetic energy distribution, (top-right) intensity plot, (bottom-lef) zero collision extrapolation, (bottom-left) zero collision extrapolated and fits obtaind from L-CID.

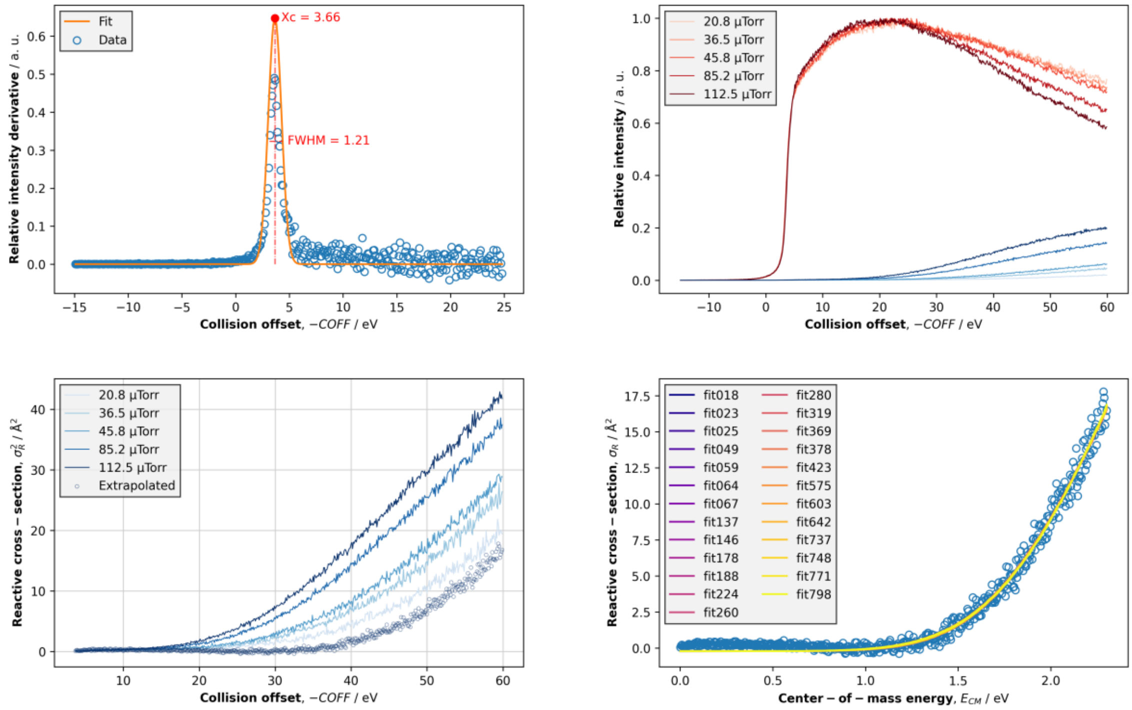

Figure SI-4-87: Experimental results (top-left) kinetic energy distribution, (top-right) intensity plot, (bottom-lef) zero collision extrapolation, (bottom-left) zero collision extrapolated and fits obtaind from L-CID.

4.4.4. T-CID / L-CID data for the Measurements of [(bhq)(bhq$^{5\text{-}CH_2NMe_3}$)Pd-Zn(Ar^{2,4,6F})$_2$]$^+$, [5$^+$]

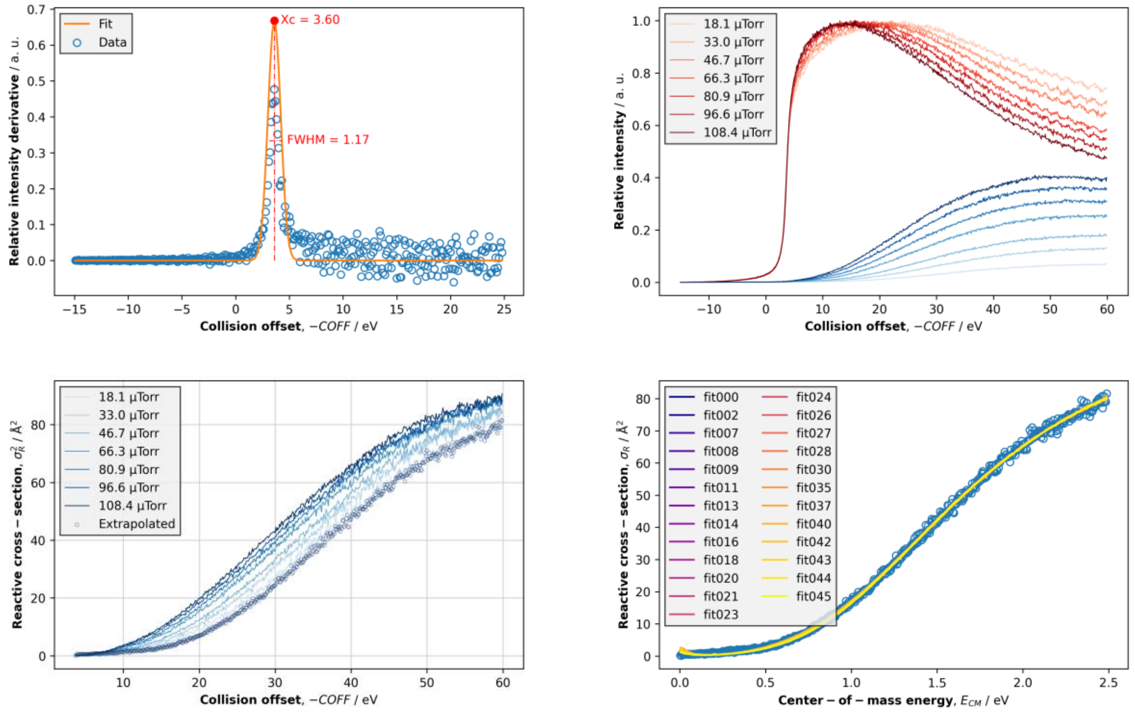

Figure SI-4-88: Experimental results (top-left) kinetic energy distribution, (top-right) intensity plot, (bottom-lef) zero collision extrapolation, (bottom-left) zero collision extrapolated and fits obtaind from L-CID.

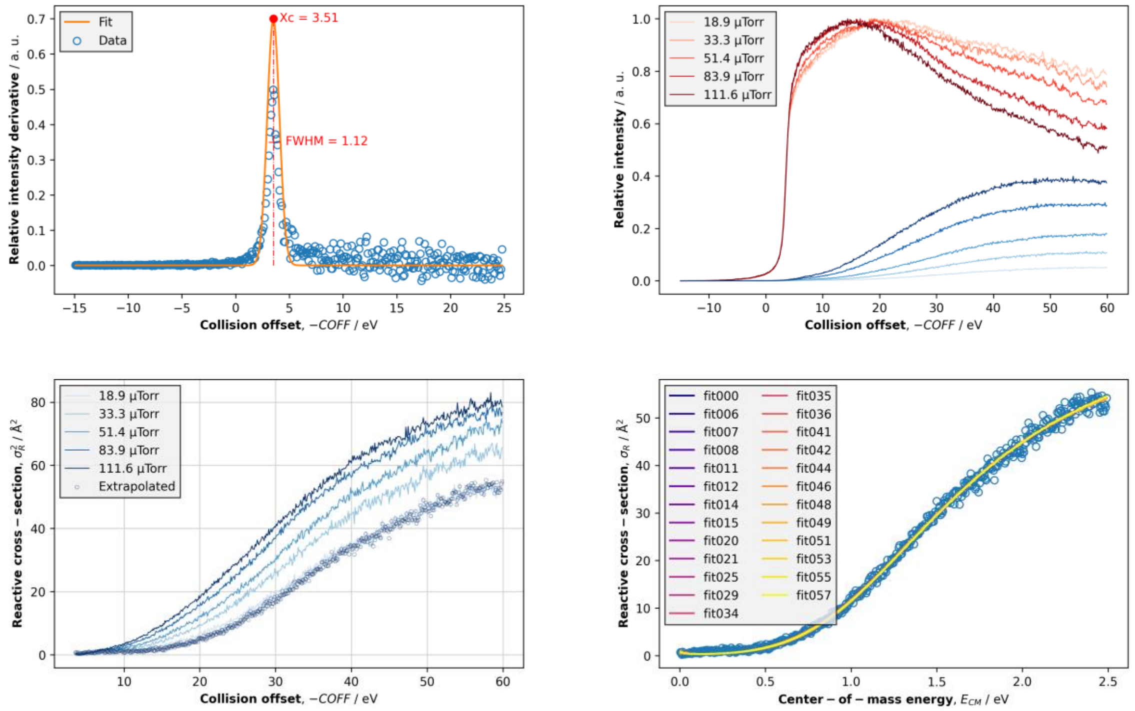

Figure SI-4-89: Experimental results (top-left) kinetic energy distribution, (top-right) intensity plot, (bottom-lef) zero collision extrapolation, (bottom-left) zero collision extrapolated and fits obtaind from L-CID.

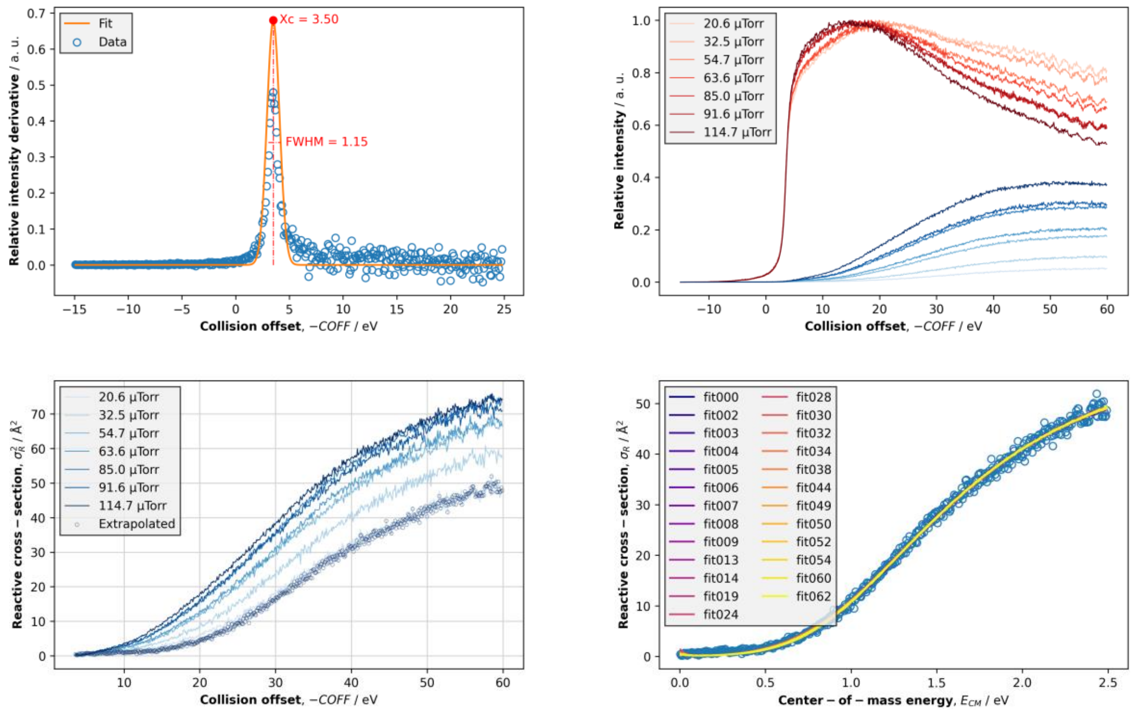

Figure SI-4-90: Experimental results (top-left) kinetic energy distribution, (top-right) intensity plot, (bottom-lef) zero collision extrapolation, (bottom-left) zero collision extrapolated and fits obtaind from L-CID.

4.4.5. T-CID / L-CID data for the Measurements of $[(bhq)(bhq^{5\text{-}CH_2NMe_3})Pd\text{-}Zn(Ar^{2,6F})_2]^+$, $[6^+]$

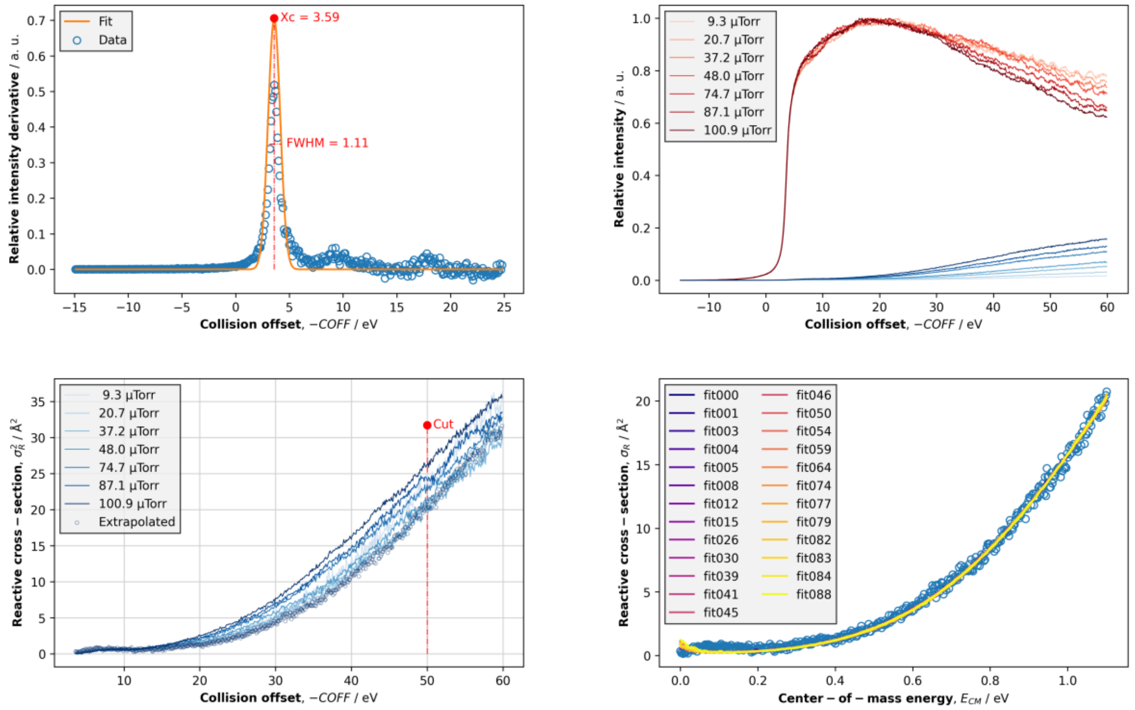

Figure SI-4-91: Experimental results (top-left) kinetic energy distribution, (top-right) intensity plot, (bottom-lef) zero collision extrapolation, (bottom-left) zero collision extrapolated and fits obtaind from L-CID.

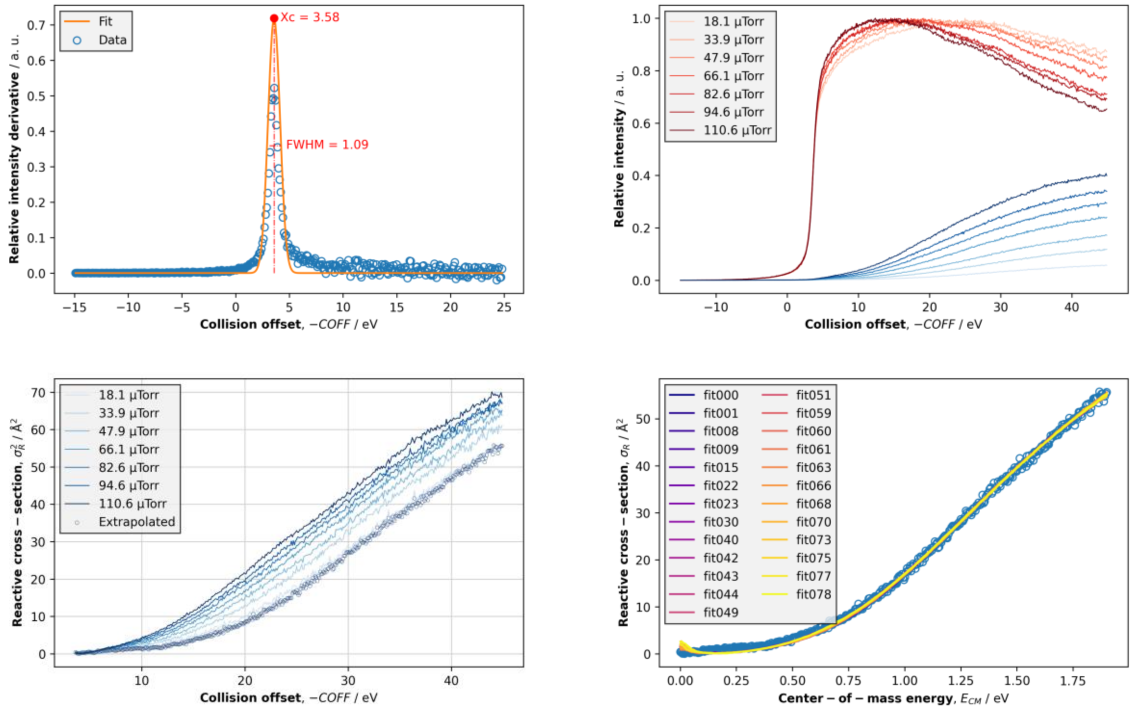

Figure SI-4-92: Experimental results (top-left) kinetic energy distribution, (top-right) intensity plot, (bottom-lef) zero collision extrapolation, (bottom-left) zero collision extrapolated and fits obtaind from L-CID.

4.4.6. T-CID / L-CID data for the Measurements of $[(bhq)_2Pd\text{-}Au(PPh_3)]^+$ [8^+]

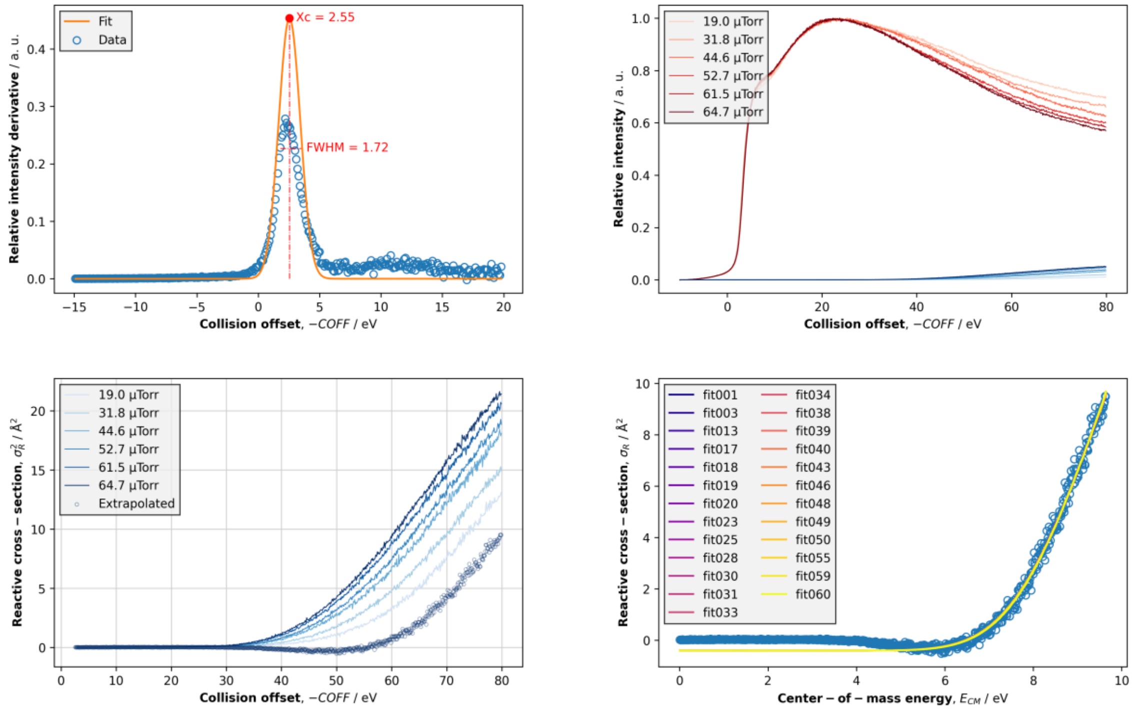

Figure SI-4-93: Experimental results (top-left) kinetic energy distribution, (top-right) intensity plot, (bottom-lef) zero collision extrapolation, (bottom-left) zero collision extrapolated and fits obtaind from L-CID.

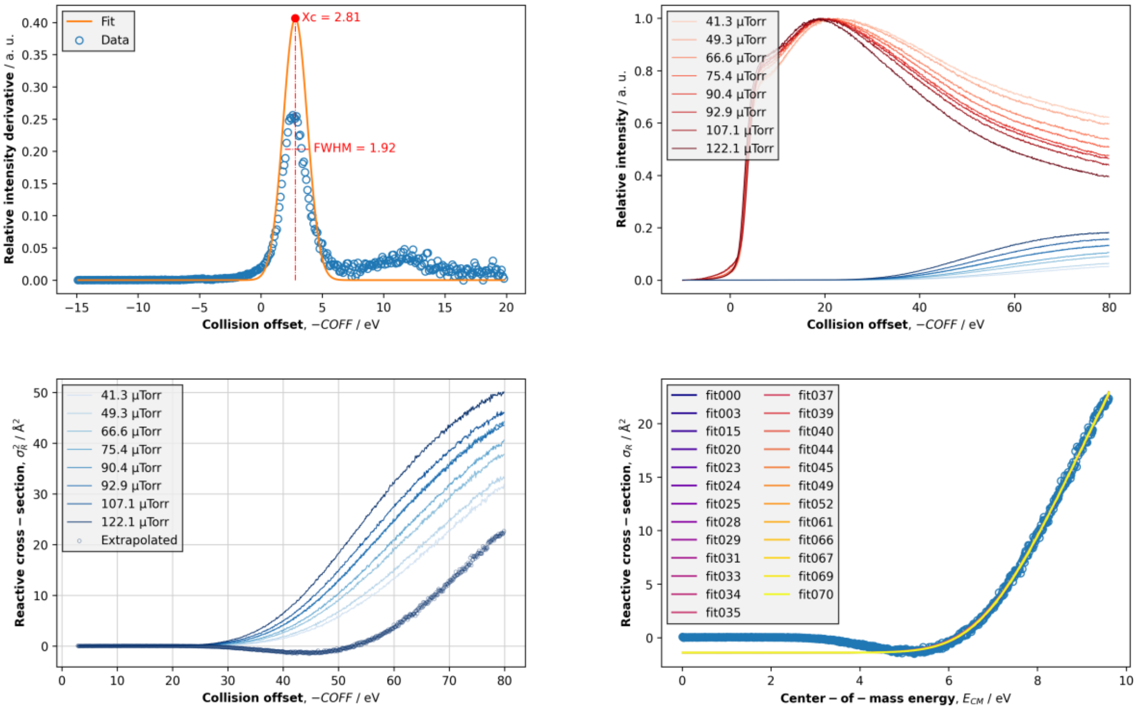

Figure SI-4-94: Experimental results (top-left) kinetic energy distribution, (top-right) intensity plot, (bottom-lef) zero collision extrapolation, (bottom-left) zero collision extrapolated and fits obtaind from L-CID.

4.4.7. T-CID / L-CID data for the Measurements of [Pd(bhq)(bhq$^{5\text{-}CH_2\text{-}NMe_3+}$)], [$7^+$]

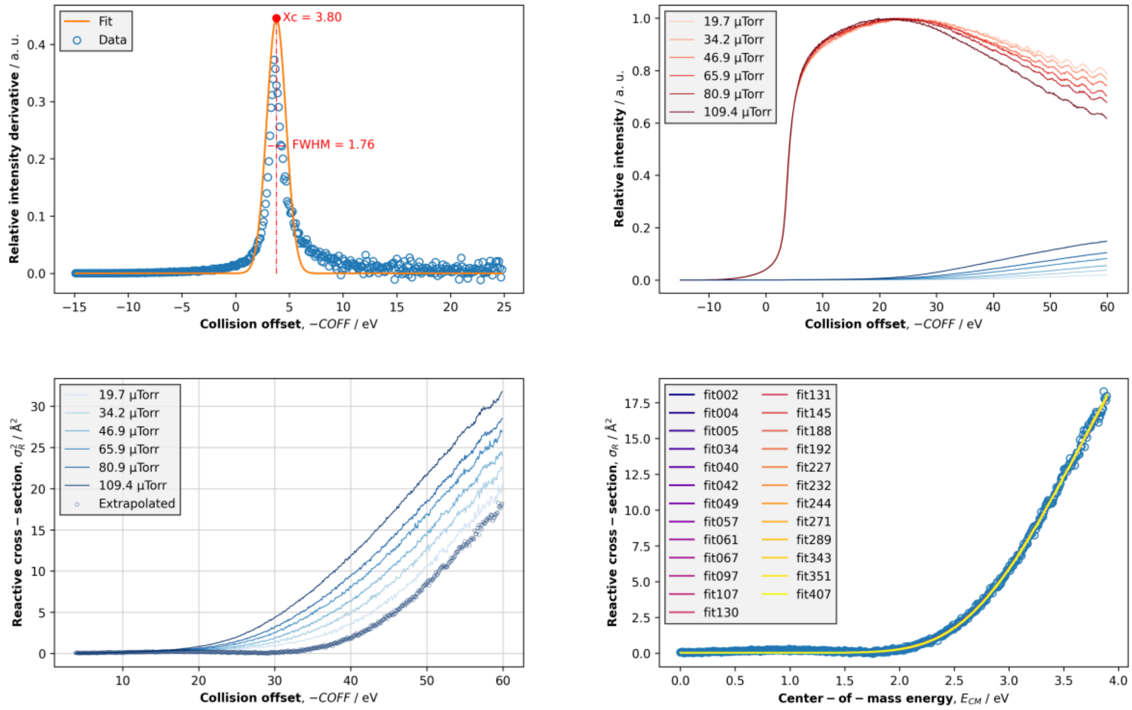

Figure SI-4-95: Experimental results (top-left) kinetic energy distribution, (top-right) intensity plot, (bottom-lef) zero collision extrapolation, (bottom-left) zero collision extrapolated and fits obtaind from L-CID.

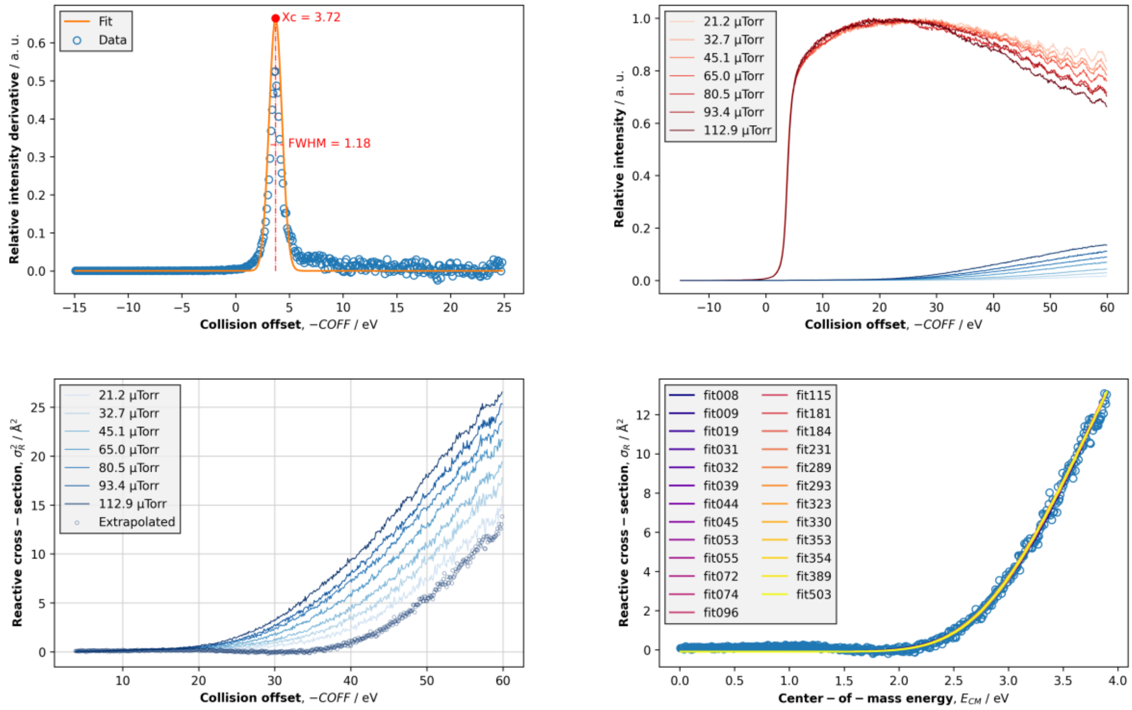

Figure SI-4-96: Experimental results (top-left) kinetic energy distribution, (top-right) intensity plot, (bottom-lef) zero collision extrapolation, (bottom-left) zero collision extrapolated and fits obtaind from L-CID.

4.5. Computational Input files and Scripts

General Information and Abbreviations

Keyword	Description:
<RUNTYPE>	Various runtypes were used: **sp** (single point calculation), **opt** (optimization using internal coordinates, w/o constraints), **copt** (optimization using cartesian coordinates), **freq** (analytical frequencies), **NumFreq** (numerical frequencies). For transition state optimizations **optts** was used.
<F>	Keyword for the choice of the electron-correlation-energy-functional, e.g. **pbe** (GGA functional) or **m06l** (meta-GGA).
<B>	Keyword for the choice of the basis set, e.g. **def2-SVP**, **def2-TZVP**, **def2-QZVP**
<Disp>	Grimme's D3 dispersion correction with Becke-Johnson damping) **d3bj** was employed together with PBE, whereas for M06-L **d3zero** dispersion correction was used.
<CHRG>	Charge value (integer value), necessary molecule specific input parameter.
<M>	Multiplicity value (integer value), necessary molecule specific input parameter.
<XYZ>	Filename of the xyz-coordinate file, e.g. the XMOL (Symbol X Y Z) format.
<OUT_FILENAME>	The filename for the calculation output.
<NP>	Number of processors requested for the calculation.
<MEM>	Memory requested for the calculation (per core).
<IDxN>	Index of the atom in the atomic-list, has to be provided for constrained calculations.
E^{Y}_{X}(Z)	represents the energy of fragment X at the optimized geometry of fragment Y with the basis set of Z.
Symbol X Y Z	Symbol refers to the element symbol, e.g. H, O. X Y Z refers to the coordinates of the specified atom.

4.5.1. CREST Calculations

The Conformer-Rotamer Ensemble Sampling Tool (CREST v.2.7.1) calculations were submitted according to the following command line along with the following additional file:

**Submission command**	sbatch --time=24:00:00 -n 8 --wrap="crest <XYZ> > <OUT_FILENAME>.out --chrg <CHRG>"
**Coordinates file (XMOL format)***:*<br>*<XYZ>*<br>*File form.:*<br>*"*.xyz"*	Number-of-Atoms<br>Header<br>Symbol X Y Z<br>.<br>.<br>.

Table SI-4-1: Input files and submission command, used for CREST calculations.

4.5.2. ORCA Input Templates

The following table is listing all input templates used for calculations with the program ORCA.

	**Coordinates file (XMOL format)***:*<br>*<XYZ>*<br>*File form.:*<br>*"*.xyz"*	Number-of-Atoms<br>Header<br>Symbol X Y Z<br>.<br>.<br>.
1	**ORCA Input Template**<br><br><br>Optimization-Frequency calculations<br><br><br>PBE / def2-TZVP (ZPE, D3BJ)	!RKS pbe d3bj def2-tzvp def2/J RI opt Freq TightSCF defgrid2 NoMOPrint Printbasis<br>%pal nprocs <NP> end<br>%maxcore <MEM><br>%base "<OUT_FILENAME_opt>"<br><br>%geom<br>TolE=1e-7<br>TolMaxG=5e-6<br>TolRMSG=3e-6

		TolMaxD=1e-4<br>TolRMSD=5e-5<br>end<br><br>*xyzfile <CHRG> <M> <XYZ>
2	**ORCA Input Template**<br><br>Single-point-calculations<br><br>PBE-D3(BJ) / def2-CBS	!RKS pbe d3bj Extrapolate(3/4,def2) def2-QZVPP/C sp TightSCF defgrid3 NoMOPrint Printbasis<br>%pal nprocs <NP> end<br>%maxcore <MEM><br>%base "<OUT_FILENAME_Sp>"<br><br>*xyzfile <CHRG> <M> <XYZ>
3	**ORCA Input Template**<br><br>Single-point-calculations<br><br>M06L-D30 / def2-CBS	!RKS m06l d3zero Extrapolate(3/4,def2) def2-QZVPP/C sp TightSCF defgrid3 NoMOPrint Printbasis<br>%pal nprocs <NP> end<br>%maxcore 6000<br>%base "<OUT_FILENAME_Sp>"<br><br>*xyzfile <CHRG> <M> <XYZ>
4	**ORCA Input Template**<br><br>Single-point-calculations<br><br>DKH2-PBE-D3(BJ) / DKH-def2-TZVP	!RKS pbe DKH2 DKH-def2-tzvp SARC/J AUTOAUX d3bj<br>%pal nprocs <NP> end<br>%maxcore <MEM><br>%base "<OUT_FILENAME_Sp>"<br><br>%basis<br>NewGTO Pd "SARC-DKH-TZVP" end<br>NewGTO Ag "SARC-DKH-TZVP" end<br>NewGTO Au "SARC-DKH-TZVP" end<br>end<br><br>*xyzfile <CHRG> <M> <XYZ>
5	**ORCA Input Template**<br><br>Single-point-calculations<br><br>ZORA-PBE-D3(BJ) / ZORA-def2-TZVP	!RKS pbe ZORA ZORA-def2-tzvp SARC/J AUTOAUX d3bj<br>%pal nprocs <NP> end<br>%maxcore <MEM><br>%base "<OUT_FILENAME_Sp>"<br><br>%basis<br>NewGTO Pd "SARC-ZORA-TZVP" end<br>NewGTO Ag "SARC-ZORA-TZVP" end<br>NewGTO Au "SARC-ZORA-TZVP" end<br>end<br><br>*xyzfile <CHRG> <M> <XYZ>
7	**ORCA Input Template**<br><br>Single-point-calculations<br><br>DKH2-M06L-D30 / DKH-def2-TZVP	!RKS m06l DKH2 DKH-def2-tzvp SARC/J AUTOAUX defgrid3 d3zero<br>%pal nprocs <NP> end<br>%maxcore <MEM><br>%base "<OUT_FILENAME_Sp>"<br><br>%basis<br>NewGTO Pd "SARC-DKH-TZVP" end<br>NewGTO Ag "SARC-DKH-TZVP" end<br>NewGTO Au "SARC-DKH-TZVP" end<br>end<br><br>*xyzfile <CHRG> <M> <XYZ>

8	**ORCA Input Template**<br><br>Single-point-calculations<br><br>ZORA-M06L-D30 / ZORA-def2-TZVP	!RKS m06l ZORA ZORA-def2-tzvp SARC/J AUTOAUX defgrid3 d3zero<br>%pal nprocs <NP> end<br>%maxcore <MEM><br>%base "<OUT_FILENAME_Sp>"<br><br>%basis<br>NewGTO Pd "SARC-ZORA-TZVP" end<br>NewGTO Ag "SARC-ZORA-TZVP" end<br>NewGTO Au "SARC-ZORA-TZVP" end<br>end<br><br>*xyzfile <CHRG> <M> <XYZ>
9	**ORCA Input Template**<br><br>Single-point-calculations<br><br>M06-D30 / def2-CBS	!RKS m06 d3zero Extrapolate(3/4,def2) def2-QZVPP/C sp AUTOAUX TightSCF defgrid3 NoMOPrint Printbasis<br>%pal nprocs <NP> end<br>%maxcore <MEM><br>%base "<OUT_FILENAME_Sp>"<br><br>*xyzfile <CHRG> <M> <XYZ>
10	**ORCA Input Template**<br><br>Single-point-calculations<br><br>DKH2-M06-D30 / DKH-def2-TZVP	!RKS m06 DKH2 DKH-def2-tzvp SARC/J AUTOAUX defgrid3 d3zero<br>%pal nprocs <NP> end<br>%maxcore <MEM><br>%base "<OUT_FILENAME_Sp>"<br><br>%scf MaxIter 1000 end<br><br>%basis<br>NewGTO Pd "SARC-DKH-TZVP" end<br>NewGTO Ag "SARC-DKH-TZVP" end<br>NewGTO Au "SARC-DKH-TZVP" end<br>end<br><br>*xyzfile <CHRG> <M> <XYZ>

Table SI-10: Input templates for computations using the ORCA program. The entries are numerated according to Tables SI-3.4.

4.5.3. ADF Input Templates

The following table is listing all input templates used for calculations with the program ADF.

	**Coordinate file**<br><XYZ><br>File form.: "*.xyz"<br>(not XMOL format)	Symbol X Y Z<br>.<br>.<br>.
6	**ADF Input Template**<br>Single-point-calculation<br><br>Calculation for PBE-D3(BJ) / ZORA-QZ4P	$ADFBIN/adf << eor<br>title <OUT_FILENAME><br><br>atoms<br>inline <XYZ><br><br>end<br><br>charge <CHRG><br><br>Basis<br>Type ZORA/QZ4P<br>end<br><br>Symmetry NOSYM<br><br>xc

		GGA PBE<br>Dispersion Grimme3 BJDAMP<br>end<br><br>relativistic ZORA<br><br>end input<br>eor<br>mv TAPE21 <OUT_FILENAME>.t21<br>mv logfile <OUT_FILENAME>.log
6a	ADF Input Template<br>Single-point-calculation / ETS-NOCV<br><br>Calculation for PBE-D3(BJ) / ZORA-QZ4P	$ADFBIN/adf << eor<br>title fragment 1<br><br>atoms<br>Symbol X Y Z<br>.<br>.<br>.<br>end<br><br>charge <CHRG_FragA><br><br>Basis<br>Type ZORA/QZ4P<br>end<br><br>Symmetry NOSYM<br><br>xc<br>GGA PBE<br>Dispersion Grimme3 BJDAMP<br>end<br><br>relativistic ZORA<br><br>end input<br>eor<br>mv TAPE21 t21.frag1<br><br>$ADFBIN/adf << eor<br>title fragment 2<br><br>atoms<br>Symbol X Y Z<br>.<br>.<br>.<br>end<br><br>charge <CHRG_FragB><br><br>Basis<br>Type ZORA/QZ4P<br>end<br><br>Symmetry NOSYM<br><br>xc

```
  GGA PBE
  Dispersion Grimme3 BJDAMP
end

relativistic ZORA

end input
eor
mv TAPE21 t21.frag2

$ADFBIN/adf << eor
title NAME ETS-NOCV

atoms
Symbol X Y Z f=a
.
Symbol X Y Z f=b
.
end

charge <CHRG_AB>

fragments
a t21.frag1
b t21.frag2
end

Basis
 Type ZORA/QZ4P
end

Symmetry NOSYM

xc
  GGA PBE
  Dispersion Grimme3 BJDAMP
end

relativistic ZORA
```

OUTPUT of NOCV’s

```
ETSNOCV
PRINT etslowdin
PRINT NOCVHirshfeld

end input
eor

$ADFBIN/densf << eor

INPUTFILE TAPE21

CUBOUTPUT NAME

GRID medium

NOCV
 N1
 SUM 1
 N2
```

		SUM 2<br>N3<br>SUM 3<br>N4<br>SUM 4<br>END<br><br>end input<br>eor
6b	**ADF Input Template**<br>Wavefunction analysis<br><br>Orbitals (*.cub) from wavefunction file TAPE21.<br><br>(PBE-D3(BJ) / ZORA-QZ4P)	$ADFBIN/densf << eor<br><br>INPUTFILE TAPE21<br><br>CUBOUTPUT NAME<br><br>GRID medium<br><br>Orbitals SCF<br>All HOMO-12 LUMO+10<br>END<br><br>end input<br>eor

Table SI-4-2: Input Templates for computations using the ADF program. The entries are numerated according to Tables SI-3.4. Entry 6 is a single point calculation, 6a is the input file for the ETS-NOCV calculation and 6b is the input file for analysis of the orbitals.

4.5.4. Single point Energies

				1		2	3	4	5	6	7	8	9	10
	**Name**			**PBE-D3BJ / Def2-TZVP**	**ZPE (PBE-D3BJ / Def2-TZVP)**	**PBE-D3bj / CBS(def2)**	**M06L-D30 / CBS(def2)**	**DKH2-PBE-D3BJ / DKH-Def2-TZVP**	**ZORA-PBE-D3BJ / ZORA-Def2-TZVP**	**PBE-D3bj / ZORA-QZ4P**	**DKH2-M06L-D30 / DKH-Def2-TZVP**	**ZORA-M06L-D30 / ZORA-Def2-TZVP**	**M06-D30 / def2-CBS**	**DKH2-M06-D30 / DKH-Def2-TZVP**
1	**$Pd(bhq)_2$**	C		-1237.01256	0.33529024	-1237.09945	-1238.32389	-6153.46497	-6213.40321	-11.5581851	-6154.86023	-6214.87455	-1237.57884	-6154.389858
2	**$(IPr)Cu^+$**	C		-2799.12505	0.55430702	-2799.26863	-2800.76926	-2813.83896	-2822.34589	-14.4139523	-2815.26295	-2823.80774	-2800.07234	-2814.638349
3	**$(IPr)Ag^+$**	D	**x**	-1305.84148	0.55452147	-1305.93844	-1307.3482	-6473.18692	-6538.98381	-14.3866296	-6474.74234	-6540.62311	-1306.57974	-6474.260635
4	**$(IPr)Au^+$**	D		-1294.6146	0.55497332	-1294.71127	-1296.0701	-20151.8365	-20835.3786	-14.3995619	-20152.322	-20836.8142	-1295.30032	-20152.52904
5	**$[(bhq)_2Pd\text{-}Cu(IPr)]^+$**	C		-4036.2545	0.89349493	-4036.48212	-4039.21431	-8967.42337	-9035.86941	-26.0910474	-8970.24929	-9038.80927	-4037.77884	-8969.162653
6	**$[(bhq)_2Pd\text{-}Ag(IPr)]^+$**	D		-2542.95771	0.89273951	-2543.14041	-2545.7761	-12626.7553	-12752.4911	-26.0488934	-12629.7089	-12755.6047	-2544.26948	-12628.76514
7	**$[(bhq)_2Pd\text{-}Au(IPr)]^+$**	D		-2531.74537	0.89293091	-2531.92716	-2534.50826	-26305.4223	-27048.9029	-26.0774237	-26307.3046	-27051.8114	-2533.00256	-26307.04977
8	**$Pd(bhq)(bhq^{5\text{-}CH2NMe3})^+$**	C		-1449.78243	0.47549784	-1449.88838	-1451.36139	-6366.32824	-6426.32736	-14.3439733	-6367.96119	-6428.0402	-1450.49115	-6367.36797
9	**$Pd(bhq)(bhq^{5\text{-}CH2})^+$**	D		-1275.4208	0.35140413	-1275.51012	-1276.78094							
10	**NMe_3**	N		-174.299979	0.11647487	-174.317164	-174.52546							
11	**$Zn(C_6F_5)_2$**	N	**x**	-3233.94155	0.09550557	-3234.08645	-3235.48869	-3251.47105	-3261.59954	-5.41003476	-3252.81225	-3262.98903	-3235.07932	-3252.496252
12	**$Zn(Ar^{2,4,6\text{-}F})_2$**	N	**x**	-2837.20601	0.12685411	-2837.32725	-2838.45497	-2854.37848	-2864.28564	-5.42027724	-2855.45005	-2865.39646	-2838.09938	-2855.179405
13	**$Zn(Ar^{2,6\text{-}F})_2$**	N	**x**	-2638.81146	0.14275112	-2638.92122	-2639.91339	-2655.80533	-2665.60182	-5.39880894	-2656.74383	-2666.57504	-2639.58378	-2656.494827
14	**$[(bhq)(bhq^{5\text{-}CH2NMe3})Pd\text{-}\ Zn(C_6F_5)_2]^+$**	C		-4683.78088	0.57290683	-4684.02758	-4686.90767	-9617.85407	-9687.98199	-19.8078817	-9620.83219	-9691.0883	-4685.63221	-9619.928357
15	**$[(bhq)(bhq^{5\text{-}CH2NMe3})Pd\text{-}\ Zn(Ar^{2,4,6\text{-}F})_2]^+$**	C		-4287.03803	0.60395595	-4287.2622	-4289.867	-9220.7545	-9290.66114	-19.8116057	-9223.46242	-9293.48819	-4288.64573	-9222.604175
16	**$[(bhq)(bhq^{5\text{-}CH2NMe3})Pd\text{-}\ Zn(Ar^{2,6\text{-}F})_2]^+$**	C		-4088.64163	0.61944213	-4088.85442	-4091.32427	-9022.17925	-9091.97522	-19.7879981	-9024.75488	-9094.66544	-4090.12796	-9023.916851
17	**$[(bhq)(bhq^{5\text{-}CH2})Pd\text{-}\ Zn(C_6F_5)_2]^+$**	D		-4509.41158	0.44848075	-4509.64161	-4512.31932							
18	**$[(bhq)(bhq^{5\text{-}CH2})Pd\text{-}\ Zn(Ar^{2,4,6\text{-}F})_2]^+$**	D		-4112.66943	0.47952255	-4112.87703	-4115.27974							
19	**$[(bhq)(bhq^{5\text{-}CH2})Pd\text{-}\ Zn(Ar^{2,6\text{-}F})_2]^+$**	D		-3914.27262	0.49525468	-3914.46906	-3916.73695							
20	**$(PPh_3)Au^+$**	N		-1171.14756	0.26784645	-1171.21718	-1172.17222	-20028.9894		-8.07052083	-20029.078	-20713.9286	-1171.65789	
21	**$[(bhq)_2Pd\text{-}Au(PPh^3)]^+$**	N		-2408.25744	0.60456645	-2408.41281	-2410.58416	-26182.5542		-19.7278004	-26184.0348	-26928.8999	-2409.3346	

Table SI-4-3: Obtained single point energies (in a.u.) for the named structures (see also additionally provided *.xyz file), at different levels of theory. Further notation is as follows: C: structures derived from CREST conformational search, D:Derived from analogous structures, N: No particular method and x which indicates structures with small imaginary frequencies. This table is continued in Table SI-4-4.

	Name	D3(BJ) (PBE)	D30(M06L)	d4 (PBE)	d4(M06L)
1	**Pd(bhq)$_2$**	-0.07624654	-0.00366285	-0.08361463	-0.01017876
2	**(IPr)Cu$^+$**	-0.10005562	-0.00784206	-0.10034375	-0.01423269
3	**(IPr)Ag$^+$**	-0.10106896	-0.00773727	-0.10146641	-0.01456473
4	**(IPr)Au$^+$**	-0.10144278	-0.00782837	-0.10193507	-0.01454296
5	**[(bhq)$_2$Pd-Cu(IPr)]$^+$**	-0.21414329	-0.02128698	-0.21904686	-0.03578565
6	**[(bhq)$_2$Pd-Ag(IPr)]$^+$**	-0.21259106	-0.02069850	-0.21780097	-0.03543945
7	**[(bhq)$_2$Pd-Au(IPr)]$^+$**	-0.21267175	-0.02064412	-0.21798116	-0.03517259
8	**Pd(bhq)(bhq$^{5\text{-}CH2NMe3}$)$^+$**	-0.09814958	-0.00550495	-0.10287816	-0.01312348
9	**Pd(bhq)(bhq$^{5\text{-}CH2}$)$^+$**	-0.07947578	-0.00391363	-0.08497990	-0.01040202
10	**NMe$_3$**	-0.00848909	-0.00016282	-0.00771463	-0.00072750
11	**Zn(C$_6$F$_5$)$_2$**	-0.02858551	-0.00103395	-0.03204642	-0.00345759
12	**Zn(Ar$^{2,4,6\text{-}F}$)$_2$**	-0.02769121	-0.00098033	-0.02974953	-0.00313639
13	**Zn(Ar$^{2,6\text{-}F}$)$_2$**	-0.02734590	-0.00094297	-0.02897979	-0.00302281
14	**[(bhq)(bhq$^{5\text{-}CH2NMe3}$)Pd- Zn(C$_6$F$_5$)$_2$]$^+$**	-0.16481909	-0.01325409	-0.17338372	-0.02617100
15	**[(bhq)(bhq$^{5\text{-}CH2NMe3}$)Pd- Zn(Ar$^{2,4,6\text{-}F}$)$_2$]$^+$**	-0.16198600	-0.01306231	-0.16864644	-0.02549734
16	**[(bhq)(bhq$^{5\text{-}CH2NMe3}$)Pd- Zn(Ar$^{2,6\text{-}F}$)$_2$]$^+$**	-0.15687470	-0.01309947	-0.16316362	-0.02472482
17	**[(bhq)(bhq$^{5\text{-}CH2}$)Pd- Zn(C$_6$F$_5$)$_2$]$^+$**	-0.14810281	-0.01094280	-0.15826821	-0.02316465
18	**[(bhq)(bhq$^{5\text{-}CH2}$)Pd- Zn(Ar$^{2,4,6\text{-}F}$)$_2$]$^+$**	-0.14391641	-0.01067936	-0.15228097	-0.02233577
19	**[(bhq)(bhq$^{5\text{-}CH2}$)Pd- Zn(Ar$^{2,6\text{-}F}$)$_2$]$^+$**	-0.13909500	-0.01047233	-0.14679646	-0.02154912
20	**(PPh$_3$)Au$^+$**	-0.05740140	-0.00272169	-0.05836339	-0.00693578
21	**[(bhq)$_2$Pd-Au(PPh^3)]$^+$**	-0.15845228	-0.01206458	-0.16567813	-0.02424994

Table SI-4-4: Obtained dispersion correction terms (in a.u.) for the named structures (see also additionally provided *.xyz file), at different levels of theory.

4.5.5. AIM-Analysis

AIM analysis was conducted based on calculations at the DKH2-M06-D3(0) / DKH-def2-TZVP // PBE-D3(BJ)/Def2-TZVP level of theory (Tables SI-3.4 and SI-3.5, entry 10). The *.wfn file was generated from the *.gbw file using the "orca_2aim *.gbw" command. The resulting *.wfn file was then processed and analyzed in MultiWFN using the provided script (Table SI-4-5) "Multiwfn *.wfn < Script_AIM.txt ".

**MultiWFN**	2 # Topology analysis
Wavefunction analysis	2 # Search CPs from nuclear positions
	3 # Search CPs from midpoint of atomic pairs
Script for AIM-Analysis	4 # Search CPs from triangle center of three atoms
from wavefunction file	8 # Generating the paths connecting (3,-3) and (3,-1) CPs
"*.wfn".	9 # Generating the paths connecting (3,+1) and (3,+3) CPs
	-10 # Return to main menu
	4 # Output and plot specific property in a plane
	3 # Laplacian of electron density
	2 # Contour line map
	200,200 # Gridpoints
	4 # Define by three atoms
➔ Manual Edit	1,24,46 # ATOM numbers (manual edit)
	-8 #Change length unit of the graph to Angstrom
	3 # Change setting of contour lines
	11 # Set color for positive contour lines, current: Black
	3 # Color
	13 # Set color for negative contour lines, current: Black
	1 # Color
	12 #Set line style and width for positive contour lines
	1,0 #
	2 # width positive conour lines
	14 # Set line style and width for negative contour lines
	10,15 #
	2 #
	1 # Save setting and return
	4 # Set details of plotting critical points and paths
	15 # Set color for CPs
	1 # (3,-3)
	5 # Black
	15 # Set color for CPs
	2 # (3,-1)
	3 # Blue
	15 # Set color for CPs
	3 # (3,+1)
	9 # Orange
	15 # Set color for CPs
	4 # (3,+3)
	10 # 10 Magenta
	0 # Return
	-3 # Change other plotting settings
	3 # Set pixel for weight and height of the exp. figure, current: 1500 1200
	3000 #
	2400 #
	8 # Set size of atomic labels, current: 50
	20 #
	0 #
	0

Table SI-4-5: MultiWFN script for AIM analysis.

4.5.6. AIM-IGMH-Analysis

AIM-IGMH analysis was conducted based on calculations at the DKH2-M06-D3(0) / DKH-def2-TZVP // PBE-D3(BJ)/Def2-TZVP level of theory (Tables SI-3.4 and SI-3.5, entry 10). The *.wfn file was generated from the *.gbw file using the “orca_2aim *.gbw” command. The resulting *.wfn file was then processed and analyzed in MultiWFN using the provided scripts (Table SI-4-6) “Multiwfn *.wfn < Scripts.txt ”.

	MultiWFN<br>1) Generate sign(λ_2)ρ<br><br><br><br><br><br>2) Output Cube files.<br>dg.cub,<br>dg_inter.cub,<br>dg_intra.cub,<br>sl2r.cub<br><br>3) Evaluating δG^{atom} and δG^{pair}	20 # Visual study of weak interaction<br>11 #IGM analysis based on Hirshfeld partition of molecular density (IGMH) (JCC, 43, 539)<br>2 # Define number of fragments, here default 2<br>ATOMS # ATOM numbers<---NEEDSMODIFICATIONS<br>c # complement the rest of the atoms to the other fragment<br>4 #Input the number of points or grid spacing in X,Y,Z, covering whole system<br>0.15 # Using 0.15 Bohr as Grid spacing<br>3 # Output cube files to current folder<br>6 # Evaluate contribution of atomic pairs and atoms to interfragment interaction<br>2 # High quality<br>y # fragments as atmdg.pdb<br>1 # Save the scatter graph to file<br>4 # 1 delta-g_inter vs. sign(lambda2)rho / 2 delta-g_intra vs. sign(lambda2)rho / 3 delta-g vs. sign(lambda2)rho / 4 delta-g_inter + delta-g_intra vs. sign(lambda2)rho<br>2 # Output scatter points to output.txt in current folder<br>3 #Output cube files to current folder<br>0<br>0<br>q
	Multiwfn<br>AIM for IGMH	2 #2 Topology analysis<br>2 #2 Search CPs from nuclear positions<br>3 #3 Search CPs from midpoint of atomic pairs<br>4 #4 Search CPs from triangle center of three atoms<br>5 #5 Search CPs from pyramid center of four atoms<br>8 #8 Generating the paths connecting (3,-3) and (3,-1) CPs<br>-4 #-4 Modify or export CPs (critical points)<br>6 # 6 Export CPs as CPs.pdb file in current folder<br>0 # 0 Return<br>-5 # -5 Modify or print detail or export paths, or plot property along a path<br>6 # 6 Export paths as paths.pdb file in current folder<br>0 # 0 Return<br>7 # 7 Show real space function values at specific CP or all CPs<br>-1 #<br>-10 # -10 Return to main menu<br>100 # 100 Other functions (Part 1)<br>2 # 2 Export various files (mwfn/pdb/xyz/wfn/wfx/molden/fch/47/mkl...) or generate input file of quantum chemistry programs<br>1 # 1 Output current structure to .pdb file<br>mol.pdb<br>0 # 0 Return<br>q # "q": Exit program gracefully
	VMD-Script<br>AIMIGMH.vmd<br><br>Run VMD and Load .vmd script as a visualization	proc vmdrestoremycolors {} {<br>color scale colors RWB {1.0 0.0 0.0} {1.0 1.0 1.0} {0.0 0.0 1.0}<br>color scale colors BWR {0.0 0.0 1.0} {1.0 1.0 1.0} {1.0 0.0 0.0}<br>color scale colors RGryB {1.0 0.0 0.0} {0.5 0.5 0.5} {0.0 0.0 1.0}<br>color scale colors BGryR {0.0 0.0 1.0} {0.5 0.5 0.5} {1.0 0.0 0.0}<br>color scale colors RGB {1.0 0.0 0.0} {0.0 1.0 0.0} {0.0 0.0 1.0}<br>color scale colors BGR {0.0 0.0 1.0} {0.0 1.0 0.0} {1.0 0.0 0.0}<br>color scale colors RWG {1.0 0.0 0.0} {1.0 1.0 1.0} {0.0 1.0 0.0}<br>color scale colors GWR {0.0 1.0 0.0} {1.0 1.0 1.0} {1.0 0.0 0.0}<br>color scale colors GWB {0.0 1.0 0.0} {1.0 1.0 1.0} {0.0 0.0 1.0}

```
color scale colors BWG {0.0 0.0 1.0} {1.0 1.0 1.0} {0.0 1.0 0.0}
color scale colors BlkW {0.0 0.0 0.0} {0.5 0.5 0.5} {1.0 1.0 1.0}
color scale colors WBlk {1.0 1.0 1.0} {0.5 0.5 0.5} {0.0 0.0 0.0}
color scale colors cividis {0.0 0.0 0.0} {0.0 0.0 0.0} {0.0 0.0 0.0}
color scale colors viridis {0.0 0.0 0.0} {0.0 0.0 0.0} {0.0 0.0 0.0}
color scale colors magma {0.0 0.0 0.0} {0.0 0.0 0.0} {0.0 0.0 0.0}
color scale colors plasma {0.0 0.0 0.0} {0.0 0.0 0.0} {0.0 0.0 0.0}
color scale colors inferno {0.0 0.0 0.0} {0.0 0.0 0.0} {0.0 0.0 0.0}
color scale colors CET_L3 {0.0 0.0 0.0} {0.0 0.0 0.0} {0.0 0.0 0.0}
color scale colors CET_L8 {0.0 0.0 0.0} {0.0 0.0 0.0} {0.0 0.0 0.0}
color scale colors CET_L9 {0.0 0.0 0.0} {0.0 0.0 0.0} {0.0 0.0 0.0}
color scale colors CET_L16 {0.0 0.0 0.0} {0.0 0.0 0.0} {0.0 0.0 0.0}
color scale colors CET_L17 {0.0 0.0 0.0} {0.0 0.0 0.0} {0.0 0.0 0.0}
color scale colors CET_L18 {0.0 0.0 0.0} {0.0 0.0 0.0} {0.0 0.0 0.0}
color scale colors CET_L19 {0.0 0.0 0.0} {0.0 0.0 0.0} {0.0 0.0 0.0}
color scale colors CET_L20 {0.0 0.0 0.0} {0.0 0.0 0.0} {0.0 0.0 0.0}
color scale colors CET_C2 {0.0 0.0 0.0} {0.0 0.0 0.0} {0.0 0.0 0.0}
color scale colors CET_C4 {0.0 0.0 0.0} {0.0 0.0 0.0} {0.0 0.0 0.0}
color scale colors CET_C6 {0.0 0.0 0.0} {0.0 0.0 0.0} {0.0 0.0 0.0}
color scale colors CET_C7 {0.0 0.0 0.0} {0.0 0.0 0.0} {0.0 0.0 0.0}
color scale colors CET_I1 {0.0 0.0 0.0} {0.0 0.0 0.0} {0.0 0.0 0.0}
color scale colors CET_I2 {0.0 0.0 0.0} {0.0 0.0 0.0} {0.0 0.0 0.0}
color scale colors CET_I3 {0.0 0.0 0.0} {0.0 0.0 0.0} {0.0 0.0 0.0}
color scale colors CET_D11 {0.0 0.0 0.0} {0.0 0.0 0.0} {0.0 0.0 0.0}
color scale colors CET_D12 {0.0 0.0 0.0} {0.0 0.0 0.0} {0.0 0.0 0.0}
color scale colors CET_R2 {0.0 0.0 0.0} {0.0 0.0 0.0} {0.0 0.0 0.0}
 color scale method BGR
 set colorcmds {
  {color Display {Background} white}
  {color Display {BackgroundTop} black}
  {color Display {BackgroundBot} blue2}
  {color Display {FPS} white}
  {color Name {LPA} green}
  {color Name {LPB} green}
  {color Name {F} pink}
  {color Type {LP} green}
  {color Type {DRUD} pink}
  {color Type {F} pink}
  {color Element {X} cyan}
  {color Element {Ac} ochre}
  {color Element {Ag} silver}
  {color Element {Al} ochre}
  {color Element {Am} ochre}
  {color Element {Ar} ochre}
  {color Element {As} ochre}
  {color Element {At} ochre}
  {color Element {Au} yellow}
  {color Element {B} ochre}
  {color Element {Ba} ochre}
  {color Element {Be} ochre}
  {color Element {Bh} ochre}
  {color Element {Bi} ochre}
  {color Element {Bk} ochre}
  {color Element {Br} ochre}
  {color Element {C} black}
  {color Element {Ca} ochre}
  {color Element {Cd} ochre}
  {color Element {Ce} ochre}
  {color Element {Cf} ochre}
```

		{color Element {Cl} ochre}<br>{color Element {Cm} ochre}<br>{color Element {Co} ochre}<br>{color Element {Cr} ochre}<br>{color Element {Cs} ochre}<br>{color Element {Cu} orange}<br>{color Element {Db} ochre}<br>{color Element {Ds} ochre}<br>{color Element {Dy} ochre}<br>{color Element {Er} ochre}<br>{color Element {Es} ochre}<br>{color Element {Eu} ochre}<br>{color Element {F} lime}<br>{color Element {Fe} ochre}<br>{color Element {Fm} ochre}<br>{color Element {Fr} ochre}<br>{color Element {Ga} ochre}<br>{color Element {Gd} ochre}<br>{color Element {Ge} ochre}<br>{color Element {He} ochre}<br>{color Element {Hf} ochre}<br>{color Element {Hg} ochre}<br>{color Element {Ho} ochre}<br>{color Element {Hs} ochre}<br>{color Element {I} ochre}<br>{color Element {In} ochre}<br>{color Element {Ir} ochre}<br>{color Element {K} ochre}<br>{color Element {Kr} ochre}<br>{color Element {La} ochre}<br>{color Element {Li} ochre}<br>{color Element {Lr} ochre}<br>{color Element {Lu} ochre}<br>{color Element {Md} ochre}<br>{color Element {Mg} ochre}<br>{color Element {Mn} ochre}<br>{color Element {Mo} ochre}<br>{color Element {Mt} ochre}<br>{color Element {Na} ochre}<br>{color Element {Nb} ochre}<br>{color Element {Nd} ochre}<br>{color Element {Ne} ochre}<br>{color Element {Ni} ochre}<br>{color Element {No} ochre}<br>{color Element {Np} ochre}<br>{color Element {Os} ochre}<br>{color Element {Pa} ochre}<br>{color Element {Pb} ochre}<br>{color Element {Pd} cyan}<br>{color Element {Pm} ochre}<br>{color Element {Po} ochre}<br>{color Element {Pr} ochre}<br>{color Element {Pt} ochre}<br>{color Element {Pu} ochre}<br>{color Element {Ra} ochre}<br>{color Element {Rb} ochre}<br>{color Element {Re} ochre}<br>{color Element {Rf} ochre}<br>{color Element {Rg} ochre}

```
  {color Element {Rh} ochre}
  {color Element {Rn} ochre}
  {color Element {Ru} ochre}
  {color Element {Sb} ochre}
  {color Element {Sc} ochre}
  {color Element {Se} ochre}
  {color Element {Sg} ochre}
  {color Element {Si} ochre}
  {color Element {Sm} ochre}
  {color Element {Sn} ochre}
  {color Element {Sr} ochre}
  {color Element {Ta} ochre}
  {color Element {Tb} ochre}
  {color Element {Tc} ochre}
  {color Element {Te} ochre}
  {color Element {Th} ochre}
  {color Element {Ti} ochre}
  {color Element {Tl} ochre}
  {color Element {Tm} ochre}
  {color Element {U} ochre}
  {color Element {V} ochre}
  {color Element {W} ochre}
  {color Element {Xe} ochre}
  {color Element {Y} ochre}
  {color Element {Yb} ochre}
  {color Element {Zr} ochre}
  {color Resname {CPS} silver}
  {color Resname {PTH} green}
  {color Resname {} white}
  {color Chain {A} blue}
  {color Chain {X} red}
  {color Segname {} blue}
  {color Conformation {all} blue}
  {color Molecule {0} blue}
  {color Molecule {1} red}
  {color Molecule {2} gray}
  {color Molecule {3} orange}
  {color Structure {3_10_Helix} blue}
  {color Surface {Grasp} gray}
  {color Labels {Springs} orange}
  {color Stage {Even} gray}
  {color Stage {Odd} silver}
 }
 foreach colcmd $colorcmds {
  set val [catch {eval $colcmd}]
 }
 color change rgb 0 0.0 0.0 1.0
 color change rgb 2 0.3499999940395355 0.3499999940395355
0.3499999940395355
 color change rgb 3 1.0 0.5 0.0
 color change rgb 4 1.0 1.0 0.0
 color change rgb 5 0.5 0.5 0.20000000298023224
 color change rgb 6 0.6000000238418579 0.6000000238418579
0.6000000238418579
 color change rgb 7 0.0 1.0 0.0
 color change rgb 9 1.0 0.6000000238418579 0.6000000238418579
 color change rgb 11 0.6499999761581421 0.0 0.6499999761581421
 color change rgb 12 0.5 0.8999999761581421 0.4000000059604645
```

```
 color change rgb 13 0.8999999761581421 0.4000000059604645
0.699999988079071
 color change rgb 14 0.5 0.30000001192092896 0.0
 color change rgb 15 0.5 0.5 0.75
 color change rgb 17 0.8799999952316284 0.9700000286102295
0.019999999552965164
 color change rgb 18 0.550000011920929 0.8999999761581421
0.019999999552965164
 color change rgb 19 0.0 0.8999999761581421 0.03999999910593033
 color change rgb 20 0.0 0.8999999761581421 0.5
 color change rgb 21 0.0 0.8799999952316284 1.0
 color change rgb 22 0.0 0.7599999904632568 1.0
 color change rgb 23 0.019999999552965164 0.3799999952316284
0.6700000166893005
 color change rgb 24 0.009999999776482582 0.03999999910593033
0.9300000071525574
 color change rgb 25 0.27000001072883606 0.0 0.9800000190734863
 color change rgb 26 0.44999998807907104 0.0 0.8999999761581421
 color change rgb 27 0.8999999761581421 0.0 0.8999999761581421
 color change rgb 28 1.0 0.0 0.6600000262260437
 color change rgb 29 0.9800000190734863 0.0 0.23000000417232513
 color change rgb 30 0.8100000023841858 0.0 0.0
 color change rgb 31 0.8899999856948853 0.3499999940395355 0.0
 color change rgb 32 0.9599999785423279 0.7200000286102295 0.0
}
vmdrestoremycolors
proc vmdrestoremymaterials {} {
 set mlist { Opaque Transparent BrushedMetal Diffuse Ghost Glass1 Glass2
Glass3 Glossy HardPlastic MetallicPastel Steel Translucent Edgy EdgyShiny
EdgyGlass Goodsell AOShiny AOChalky AOEdgy BlownGlass GlassBubble
RTChrome Material25 }
 set mymlist [material list]
 foreach mat $mlist {
  if { [lsearch $mymlist $mat] == -1 } {
   material add $mat
  }
 }
 material change ambient Opaque 0.000000
 material change diffuse Opaque 0.650000
 material change specular Opaque 0.800000
 material change shininess Opaque 0.534020
 material change mirror Opaque 0.000000
 material change opacity Opaque 1.000000
 material change outline Opaque 0.000000
 material change outlinewidth Opaque 0.000000
 material change transmode Opaque 0.000000
 material change ambient Transparent 0.000000
 material change diffuse Transparent 0.650000
 material change specular Transparent 0.500000
 material change shininess Transparent 0.534020
 material change mirror Transparent 0.000000
 material change opacity Transparent 0.300000
 material change outline Transparent 0.000000
 material change outlinewidth Transparent 0.000000
 material change transmode Transparent 0.000000
 material change ambient BrushedMetal 0.080000
 material change diffuse BrushedMetal 0.390000
 material change specular BrushedMetal 0.340000
 material change shininess BrushedMetal 0.150000
```

		material change mirror BrushedMetal 0.000000<br>material change opacity BrushedMetal 1.000000<br>material change outline BrushedMetal 0.000000<br>material change outlinewidth BrushedMetal 0.000000<br>material change transmode BrushedMetal 0.000000<br>material change ambient Diffuse 0.000000<br>material change diffuse Diffuse 0.620000<br>material change specular Diffuse 0.000000<br>material change shininess Diffuse 0.530000<br>material change mirror Diffuse 0.000000<br>material change opacity Diffuse 1.000000<br>material change outline Diffuse 0.000000<br>material change outlinewidth Diffuse 0.000000<br>material change transmode Diffuse 0.000000<br>material change ambient Ghost 0.000000<br>material change diffuse Ghost 0.000000<br>material change specular Ghost 1.000000<br>material change shininess Ghost 0.230000<br>material change mirror Ghost 0.000000<br>material change opacity Ghost 0.100000<br>material change outline Ghost 0.000000<br>material change outlinewidth Ghost 0.000000<br>material change transmode Ghost 0.000000<br>material change ambient Glass1 0.000000<br>material change diffuse Glass1 0.500000<br>material change specular Glass1 0.650000<br>material change shininess Glass1 0.530000<br>material change mirror Glass1 0.000000<br>material change opacity Glass1 0.150000<br>material change outline Glass1 0.000000<br>material change outlinewidth Glass1 0.000000<br>material change transmode Glass1 0.000000<br>material change ambient Glass2 0.520000<br>material change diffuse Glass2 0.760000<br>material change specular Glass2 0.220000<br>material change shininess Glass2 0.590000<br>material change mirror Glass2 0.000000<br>material change opacity Glass2 0.680000<br>material change outline Glass2 0.000000<br>material change outlinewidth Glass2 0.000000<br>material change transmode Glass2 0.000000<br>material change ambient Glass3 0.150000<br>material change diffuse Glass3 0.250000<br>material change specular Glass3 0.750000<br>material change shininess Glass3 0.800000<br>material change mirror Glass3 0.000000<br>material change opacity Glass3 0.500000<br>material change outline Glass3 0.000000<br>material change outlinewidth Glass3 0.000000<br>material change transmode Glass3 0.000000<br>material change ambient Glossy 0.000000<br>material change diffuse Glossy 0.650000<br>material change specular Glossy 1.000000<br>material change shininess Glossy 0.880000<br>material change mirror Glossy 0.000000<br>material change opacity Glossy 1.000000<br>material change outline Glossy 0.000000<br>material change outlinewidth Glossy 0.000000<br>material change transmode Glossy 0.000000

		material change ambient HardPlastic 0.000000<br>material change diffuse HardPlastic 0.560000<br>material change specular HardPlastic 0.280000<br>material change shininess HardPlastic 0.690000<br>material change mirror HardPlastic 0.000000<br>material change opacity HardPlastic 1.000000<br>material change outline HardPlastic 0.000000<br>material change outlinewidth HardPlastic 0.000000<br>material change transmode HardPlastic 0.000000<br>material change ambient MetallicPastel 0.000000<br>material change diffuse MetallicPastel 0.260000<br>material change specular MetallicPastel 0.550000<br>material change shininess MetallicPastel 0.190000<br>material change mirror MetallicPastel 0.000000<br>material change opacity MetallicPastel 1.000000<br>material change outline MetallicPastel 0.000000<br>material change outlinewidth MetallicPastel 0.000000<br>material change transmode MetallicPastel 0.000000<br>material change ambient Steel 0.250000<br>material change diffuse Steel 0.000000<br>material change specular Steel 0.380000<br>material change shininess Steel 0.320000<br>material change mirror Steel 0.000000<br>material change opacity Steel 1.000000<br>material change outline Steel 0.000000<br>material change outlinewidth Steel 0.000000<br>material change transmode Steel 0.000000<br>material change ambient Translucent 0.000000<br>material change diffuse Translucent 0.700000<br>material change specular Translucent 0.600000<br>material change shininess Translucent 0.300000<br>material change mirror Translucent 0.000000<br>material change opacity Translucent 0.800000<br>material change outline Translucent 0.000000<br>material change outlinewidth Translucent 0.000000<br>material change transmode Translucent 0.000000<br>material change ambient Edgy 0.000000<br>material change diffuse Edgy 0.660000<br>material change specular Edgy 0.000000<br>material change shininess Edgy 0.750000<br>material change mirror Edgy 0.000000<br>material change opacity Edgy 1.000000<br>material change outline Edgy 0.620000<br>material change outlinewidth Edgy 0.940000<br>material change transmode Edgy 0.000000<br>material change ambient EdgyShiny 0.000000<br>material change diffuse EdgyShiny 0.660000<br>material change specular EdgyShiny 0.960000<br>material change shininess EdgyShiny 0.750000<br>material change mirror EdgyShiny 0.000000<br>material change opacity EdgyShiny 1.000000<br>material change outline EdgyShiny 0.760000<br>material change outlinewidth EdgyShiny 0.940000<br>material change transmode EdgyShiny 0.000000<br>material change ambient EdgyGlass 0.000000<br>material change diffuse EdgyGlass 0.660000<br>material change specular EdgyGlass 0.500000<br>material change shininess EdgyGlass 0.750000<br>material change mirror EdgyGlass 0.000000

		material change opacity EdgyGlass 0.620000<br>material change outline EdgyGlass 0.620000<br>material change outlinewidth EdgyGlass 0.940000<br>material change transmode EdgyGlass 0.000000<br>material change ambient Goodsell 0.520000<br>material change diffuse Goodsell 1.000000<br>material change specular Goodsell 0.000000<br>material change shininess Goodsell 0.000000<br>material change mirror Goodsell 0.000000<br>material change opacity Goodsell 1.000000<br>material change outline Goodsell 4.000000<br>material change outlinewidth Goodsell 0.900000<br>material change transmode Goodsell 0.000000<br>material change ambient AOShiny 0.000000<br>material change diffuse AOShiny 0.850000<br>material change specular AOShiny 0.200000<br>material change shininess AOShiny 0.530000<br>material change mirror AOShiny 0.000000<br>material change opacity AOShiny 1.000000<br>material change outline AOShiny 0.000000<br>material change outlinewidth AOShiny 0.000000<br>material change transmode AOShiny 0.000000<br>material change ambient AOChalky 0.000000<br>material change diffuse AOChalky 0.850000<br>material change specular AOChalky 0.000000<br>material change shininess AOChalky 0.530000<br>material change mirror AOChalky 0.000000<br>material change opacity AOChalky 1.000000<br>material change outline AOChalky 0.000000<br>material change outlinewidth AOChalky 0.000000<br>material change transmode AOChalky 0.000000<br>material change ambient AOEdgy 0.000000<br>material change diffuse AOEdgy 0.900000<br>material change specular AOEdgy 0.200000<br>material change shininess AOEdgy 0.530000<br>material change mirror AOEdgy 0.000000<br>material change opacity AOEdgy 1.000000<br>material change outline AOEdgy 0.620000<br>material change outlinewidth AOEdgy 0.930000<br>material change transmode AOEdgy 0.000000<br>material change ambient BlownGlass 0.040000<br>material change diffuse BlownGlass 0.340000<br>material change specular BlownGlass 1.000000<br>material change shininess BlownGlass 1.000000<br>material change mirror BlownGlass 0.000000<br>material change opacity BlownGlass 0.100000<br>material change outline BlownGlass 0.000000<br>material change outlinewidth BlownGlass 0.000000<br>material change transmode BlownGlass 1.000000<br>material change ambient GlassBubble 0.250000<br>material change diffuse GlassBubble 0.340000<br>material change specular GlassBubble 1.000000<br>material change shininess GlassBubble 1.000000<br>material change mirror GlassBubble 0.000000<br>material change opacity GlassBubble 0.040000<br>material change outline GlassBubble 0.000000<br>material change outlinewidth GlassBubble 0.000000<br>material change transmode GlassBubble 1.000000<br>material change ambient RTChrome 0.000000

		material change diffuse RTChrome 0.650000<br>material change specular RTChrome 0.500000<br>material change shininess RTChrome 0.530000<br>material change mirror RTChrome 0.700000<br>material change opacity RTChrome 1.000000<br>material change outline RTChrome 0.000000<br>material change outlinewidth RTChrome 0.000000<br>material change transmode RTChrome 0.000000<br>material change ambient Material25 0.000000<br>material change diffuse Material25 0.760000<br>material change specular Material25 0.310000<br>material change shininess Material25 0.660000<br>material change mirror Material25 0.000000<br>material change opacity Material25 0.800000<br>material change outline Material25 0.000000<br>material change outlinewidth Material25 0.000000<br>material change transmode Material25 1.000000<br>}<br>vmdrestoremymaterials
	# The Main script	## AIM<br>color Display Background white<br>axes location Off<br>display depthcue off<br>display rendermode GLSL<br><br>set CPsize 0.05<br>set pathsize 0.005<br><br>mol new CPs.pdb<br>#Set color for (3,-3) # NUCLEAR POSITIONS<br>mol modselect 0 0 name C<br>mol modstyle 0 0 VDW $CPsize 22.0<br>mol modcolor 0 0 ColorID 16<br>#Load and set (3,-1) # BOND CIRTICAL POINTS<br>mol addrep 0<br>mol modselect 1 0 name N<br>mol modstyle 1 0 VDW $CPsize 22.0<br>mol modcolor 1 0 ColorID 11<br>##Load and set (3,+1) # RING CRITICAL POINTS CURRENTLY OFF<br>#mol addrep 0<br>#mol modselect 2 0 name O<br>#mol modstyle 2 0 VDW $CPsize 22.0<br>#mol modcolor 2 0 ColorID 3<br>##Load and set (3,+3) # CAGE CRITICAL POINTS CURRENTLY OFF<br>#mol addrep 0<br>#mol modselect 3 0 name F<br>#mol modstyle 3 0 VDW $CPsize 22.0<br>#mol modcolor 3 0 ColorID 11<br>#Load and set topology paths<br>mol new paths.pdb<br>mol modstyle 0 1 VDW $pathsize 22.0<br>mol modcolor 0 1 ColorID 32<br>#Load molecular structure<br>mol new mol.pdb<br>mol modstyle 0 2 CPK 0.7 0.3 22.0 22.0<br>mol off 2

```
### IGMH
mol new sl2r.cub
mol addfile dg_inter.cub
mol delrep 0 top
#mol representation CPK 1.0 0.3 18.0 16.0 # ORIGINAL
#mol representation CPK 0.7 0.3 22.0 22.0 # THE SAME AS IN AIM.txt
mol representation CPK 0.6 0.2 80.0 80.0
mol color Element
mol addrep top
mol representation Isosurface 0.00250 1 0 0 1 1
mol color Volume 0
mol material Material25
mol addrep top
mol scaleminmax top 1 -0.05 0.05
color scale method BGR
color Display Background white
axes location Off
display depthcue off
display rendermode GLSL
display projection   Orthographic
light 3 on
material change specular Opaque 0.300000




proc labcp {cptype {labsize 1.8} {offsetx -0.1} {offsety 0.0}} {
label delete Atoms all
if {$cptype=="no"} {return}
color Labels Atoms blue
label textthickness 2.000000
label textsize $labsize
set atmsel all
if {$cptype=="3n3"} {set atmsel "name C"}
if {$cptype=="3n1"} {set atmsel "name N"}
if {$cptype=="3p1"} {set atmsel "name O"}
if {$cptype=="3p3"} {set atmsel "name F"}
set sel [atomselect 0 $atmsel]
set k 0
foreach i [$sel list] {
label add Atoms 0/$i
label textformat Atoms $k { %1i }
label textoffset Atoms $k "$offsetx $offsety"
incr k
}
$sel delete
}

proc labcpidx {cpidx {labsize 1.8} {offsetx -0.1} {offsety 0.0}} {
label delete Atoms all
color Labels Atoms blue
label textthickness 2.000000
label textsize $labsize
set sel [atomselect 0 "serial $cpidx"]
set k 0
foreach i [$sel list] {
```

		label add Atoms 0/$i<br>label textformat Atoms $k { %1i }<br>label textoffset Atoms $k "$offsetx $offsety"<br>incr k<br>}<br>$sel delete<br>}

Table SI-4-6: MultiWFN script for IGMH and AIM analysis, which is visualized using VMD.

4.5.7. DFT-D3 Analysis

The dftd3 program,[9] was used with the following command:
dftd3 *.xyz -func pbe -bj -anal

	**Fragment file** External input file required to be in the same directory File form.: “fragment.txt”	FragmentA<br>.<br>FragmentB<br>.

Table SI-4-7: Fragment file used for dftd3 analysis.

4.5.8. Scripts

```
#### R-Script for analyzing structural characteristics between molecular fragments ####
# The following files must be present in the same directory as this script: *.xyz and fragment.
## -- The "*.xyz" file: XMOL-format coordinates ----------------------------------------------
# Header-Line-Number-of-Atoms
# Header Line 2: the name of the Structure (can be empty)
# A1 x y z      # Atom label for Atom A1 and the corresponding xyz coordinates
# A2 x y z
## -- The "fragment" file: Lines define a fragments using the index numbers of the atoms. -----
# A1
# A1,A2
# A1-A2

### Workspace Preparations -------------------------------------------------------------------
rm(list = ls())
library(rstudioapi)
library(stringr)
current_path = rstudioapi::getActiveDocumentContext()$path
setwd(dirname(current_path ))
path='./'#

### Main Script ------------------------------------------------------------------------------
## -- Read the "*.xyz" file
xyzFN <- Sys.glob("*.xyz")                          # list all files in directory with ending *.xyz
xyzFN <- xyzFN[!grepl("centroids\\.xyz$", xyzFN)] # exclude files that contain "centroids" in name
XYZ=read.table(file=paste(path,xyzFN, sep=''),skip=2,sep="") # read the "*.xyz" file
colnames(XYZ)=c('AtomType','x','y','z')
## -- Read "fragment" file
f=read.table(file=paste(path,"fragment", sep=''),skip=0,sep="\n",header=F,fill=T) # read "fragment" file

## --  Reformat Fragment Data to a Matrix: fmat with dim: the number of rows in XYZ and f
fmat=matrix(NA,length(XYZ[,1]),length(f[,1]))      # creates an empty matrix fmat
for (i in 1:length(fmat[1,])){                      # loop over fragments
  fi_Aidx=c()                                       # creates an empty list, index of atoms in fragment i
  f_csep=unlist(str_split(f[i,],","))               # unlist items using comma separators ","
  for (j in 1:length(f_csep)){                      # loop over all comma-separated elements
    f_hsep=unlist(str_split(f_csep[j],"-"))          # unlist comma-separated-element using line separators
"-"
    if (length(f_hsep)==1){fi_Aidx=c(fi_Aidx,f_csep[j]) # if no "-" separators are used in comma-separated-
element j
    } else{                fi_Aidx=c(fi_Aidx,f_hsep[1]:f_hsep[2])}
  }                                                 # loop-return j, loop over comma-separated elements
  fmat[as.numeric(fi_Aidx),i]=1                     # assigns atoms-indexes to fragment i
}                                                   # loop-return i, loop over all fragments

## -- Generate All Combinations of Fragments
fcomb=unique(t(apply(merge(1:length(fmat[1,]), 1:length(fmat[1,])), MARGIN = 1, FUN = sort)))
fcomb=fcomb[-which(fcomb[,1]==fcomb[,2]),]         # remove fragment combinations so that Frag1!=Frag2
```

[9]Obtained from https://www.chemie.uni-bonn.de/grimme/de/software/dft-d3/get_dft-d3 (last checked 18.09.24)

```
## -- Determination of Specific Structural Information
data_res=c()
cF=c()
for (i in 1:(length(fcomb)/2)){     # loop i: over fragment combinations
# --- Fragments
  if (length(fmat[1,])==2){         # if only 1 fragment combination
    F1_IDX=fcomb[1]                 # fragment 1
    F2_IDX=fcomb[2]                 # fragment 2
  } else {
    F1_IDX=fcomb[i,1]               # fragment 1
    F2_IDX=fcomb[i,2]               # fragment 2
  }
  F1=which(fmat[,F1_IDX]!="NA")     # indexes of atoms in fragment 1
  F2=which(fmat[,F2_IDX]!="NA")     # indexes of atoms in fragment 2
# --- Inter-Fragment Distances
  comb_f=expand.grid(F1,F2)         # generate atom-pairs between frag 1 and 2
  F1_xyz=XYZ[comb_f[,1],2:4]        # coordinates of atoms in Fragment 1
  F2_xyz=XYZ[comb_f[,2],2:4]        # coordinates of atoms in Fragment 2
  df=rowSums((F2_xyz-F1_xyz)^2)^0.5# distance-list between atom-pairs i,j
  df_min=cbind(min(df),comb_f[which.min(df),]) # minimal distance i,j
  df_avg=mean(df)                   # average distance between frag 1 and frag 2
  # --- Inter-Fragment Weighted Distances
  w=(df^-6/sum(df^-6)) # weighted average distance between frag 1 and frag 2
  df_w=sum(df*w)

# --- Centroids and Centroid-Distances
  cF1=colSums(XYZ[F1,2:4])/length(F1)    # centroid of fragment 1
  cF2=colSums(XYZ[F2,2:4])/length(F2)    # centroid of fragment 2
  dc = sum((cF1-cF2)^2)^0.5              # distances between the fragment centroids

## -- Write to Variable for later Analysis

data_res=rbind(data_res,cbind(F1_IDX,F2_IDX,round(df_avg,2),round(df_w,2),round(dc,2),round(df_min,2),len
gth(F1),length(F2),paste(F1,collapse="-"),paste(F2,collapse="-")))
  cF=rbind(cF,cF1,cF2)
}                                        # loop-back i: over all fragment-combinations

## -- Print Data_res Results ------
# --- Save Results Table to File
FN_resTable=paste(strsplit(xyzFN,".xyz"),"_resTable.txt",sep="") # FileName_ResultsTable
write.table(t(c("F1","F2","d_av","d_w","d_c","dmin","i_dmin","j_dmin","len_F1","len_F2","i_F1","j_F2")),F
N_resTable, append=FALSE, row.names = F, col.names=F, quote=FALSE,sep="\t")
write.table(data_res,FN_resTable, append=TRUE, row.names = F, col.names=F,sep="\t")

# --- Create a *.xyz File, Including the Centroid Coordinates
FN_XYZcentroid=paste(strsplit(xyzFN,".xyz"),"_centroids.xyz",sep="")
file.copy(xyzFN,FN_XYZcentroid,overwrite=TRUE)
ucF <- unique(cF)       # reduce to unique found centroids
for (i in 1:length(ucF[,1])){
  write(paste("X",ucF[i,1],ucF[i,2],ucF[i,3],collapse="\t"),file=FN_XYZcentroid,append=TRUE)
}
```

Table SI-4-8: R-Script for analyzing structural characteristics between molecular fragments.

5. References

1. Jolliet, P., Gianini, M., von Zelewsky, A., Bernardinelli, G. & Stoeckli-Evans, H. Cyclometalated Complexes of Palladium(II) and Platinum(II): cis -Configured Homoleptic and Heteroleptic Compounds with Aromatic C⌒N Ligands. *Inorg. Chem.* 35, 4883–4888 (**1996**).
2. Oeschger, R. J., Bissig, R. & Chen, P. Model Compounds for Intermediates and Transition States in Sonogashira and Negishi Coupling: d 8 – d 10 Bonds in Large Heterobimetallic Complexes Are Weaker than Computational Chemistry Predicts. *J. Am. Chem. Soc.* 144, 10330–10343 (**2022**).
3. Orwat, B., Oh, M. joon O., Kubicki, M. & Kownacki, I. Synthesis of 5-Substituted Benzo[h]quinoline Derivatives via Reactions Involving C(sp2)−Br Bond Activation. *Adv. Synth. Catal.* 360, 3331–3344 (**2018**).
4. Fulmer, G. R. *et al.* NMR chemical shifts of trace impurities: Common laboratory solvents, organics, and gases in deuterated solvents relevant to the organometallic chemist. *Organometallics* 29, 2176–2179 (**2010**).
5. Solar, M. & Trapp, N. µCHILL: a lightweight, modular system for handling crystalline samples at low temperatures under inert conditions. *J. Appl. Crystallogr.* 51, 541–548 (**2018**).
6. Dolomanov, O. V., Bourhis, L. J., Gildea, R. J., Howard, J. A. K. & Puschmann, H. OLEX2 : a complete structure solution, refinement and analysis program. *J. Appl. Crystallogr.* 42, 339–341 (**2009**).
7. Sheldrick, G. M. SHELXT - Integrated space-group and crystal-structure determination. *Acta Crystallogr. Sect. A Found. Crystallogr.* 71, 3–8 (**2015**).
8. Sheldrick, G. M. Crystal structure refinement with SHELXL. *Acta Crystallogr. Sect. C Struct. Chem.* 71, 3–8 (**2015**).
9. Sheldrick, G. M. A short history of SHELX. *Acta Crystallogr. Sect. A Found. Crystallogr.* 64, 112–122 (**2008**).
10. Narancic, S., Bach, A. & Chen, P. Simple fitting of energy-resolved reactive cross sections in threshold collision-induced dissociation (T-CID) experiments. *J. Phys. Chem. A* 111, 7006–7013 (**2007**).
11. Oeschger, R. J. & Chen, P. Structure and Gas-Phase Thermochemistry of a Pd/Cu Complex: Studies on a Model for Transmetalation Transition States. *J. Am. Chem. Soc.* 139, 1069–1072 (**2017**).
12. Oeschger, R. J. & Chen, P. A Heterobimetallic Pd–Zn Complex: Study of a d 8 –d 10 Bond in Solid State, in Solution, and in Silico. *Organometallics* 36, 1465–1468 (**2017**).
13. Jolliet, P., Gianini, M., Von Zelewsky, A., Bernardinelli, G. & Stoeckli-Evans, H. Cyclometalated Complexes of Palladium(II) and Platinum(II): Cis-Configured Homoleptic and Heteroleptic Compounds with Aromatic C N Ligands. *Inorg. Chem.* 35, 4883–4888 (**1996**).
14. Powers, D. C. & Ritter, T. Bimetallic Pd(III) complexes in palladium-catalysed carbon–heteroatom bond formation. *Nat. Chem.* 1, 302–309 (**2009**).
15. Furuya, T. *et al.* Mechanism of C-F reductive elimination from palladium(IV) fluorides. *J. Am. Chem. Soc.* 132, 3793–3807 (**2010**).
16. Cockburn, B. N., Howe, D. V, Keating, T., Johnson, B. F. G. & Lewis, J. Reactivity of co-ordinated ligands. Part XV. Formation of complexes containing Group V donor atoms and metal–carbon σ-bonds. *J. Chem. Soc., Dalt. Trans.* 404–410 (**1973**) doi:10.1039/DT9730000404.
17. Foster, R. E., Lipscomb, R. D., Thompson, T. J. & Hamilton, C. S. Some γ-Substituted Benzoquinoline Derivatives 1. *J. Am. Chem. Soc.* 68, 1327–1330 (**1946**).
18. Utermohlen, W. P. & Hamilton, C. S. Some Derivatives of Benzo [h] quinoline. *J. Am. Chem. Soc.* 63, 156–159 (**1941**).
19. Riesgo, E. C., Jin, X. & Thummel, R. P. Introduction of benzo[h]quinoline and 1,10-phenanthroline subunits by friedländer methodology. *J. Org. Chem.* 61, 3017–3022 (**1996**).
20. Collis, G. E. & Burrell, A. K. Studies into the generation and Diels–Alder reactions of 7,8-quinolyne with furan dienes. *Tetrahedron Lett.* 46, 3653–3656 (**2005**).
21. Mazaheripour, A. *et al.* An Aza-Diels–Alder Approach to Crowded Benzoquinolines. *Org. Lett.* 18, 156–159 (**2016**).
22. Kawazoe, Y., Wu, W. & Saeki, K. Substituent Effect of the Fluorine Atom on Electrophilic Deuteration of Benzo[h]quinoline. *Heterocycles* 43, 1937 (**1996**).
23. Dubarle Offner, J., Schnakenburg, G., Rose-Munch, F., Rose, E. & Dötz, K. H. Dibenzo[f, h]quinoline Cr(CO)3 complexes: Synthesis by chromium-templated benzannulation, cyclomanganation, and haptotropic chromium migration. *Organometallics* 29, 3308–3317 (**2010**).
24. Oeschger, R. J. Development of Model Complexes for the Study of the Transmetalation Step in Palladium Catalyzed Cross-Coupling Reactions. *PhD Thesis* (**2017**).
25. Yang, L., Lin, J., Kang, L., Zhou, W. & Ma, D. Y. Lewis Acid-Catalyzed Reductive Amination of Aldehydes and Ketones with N,N-Dimethylformamide as Dimethylamino Source, Reductant and Solvent. *Adv. Synth. Catal.* 360, 485–490 (**2018**).
26. Pollice, R., Bot, M., Kobylianskii, I. J., Shenderovich, I. & Chen, P. Attenuation of London Dispersion in Dichloromethane Solutions. *J. Am. Chem. Soc.* 139, 13126–13140 (**2017**).

27. Bot, M., Gorbachev, V., Tsybizova, A. & Chen, P. Bond Dissociation Energies in the Gas Phase for Large Molecular Ions by Threshold Collision-Induced Dissociation Experiments: Stretching the Limits. *J. Phys. Chem. A* 124, 8692–8707 (**2020**).
28. Stephens, E. R., Dumlao, M., Xiao, D., Zhang, D. & Donald, W. A. Benzylammonium Thermometer Ions: Internal Energies of Ions Formed by Low Temperature Plasma and Atmospheric Pressure Chemical Ionization. *J. Am. Soc. Mass Spectrom.* 26, 2081–2084 (**2015**).
29. Oeschger, R. J., Ringger, D. H. & Chen, P. Gas-Phase Investigations on the Transmetalation Step in Sonogashira Reactions. *Organometallics* 34, 3888–3892 (**2015**).
30. Paenurk, E., Gershoni-Poranne, R. & Chen, P. Trends in Metallophilic Bonding in Pd-Zn and Pd-Cu Complexes. *Organometallics* 36, 4854–4863 (**2017**).
31. Reiher, M. A Theoretical Challenge: Transition-Metal Compounds. *Chimia (Aarau).* 63, 140 (2009).
32. Grimme, S. *et al.* Fully Automated Quantum-Chemistry-Based Computation of Spin–Spin-Coupled Nuclear Magnetic Resonance Spectra. *Angew. Chemie Int. Ed.* 56, 14763–14769 (**2017**).
33. Grimme, S. Exploration of Chemical Compound, Conformer, and Reaction Space with Meta-Dynamics Simulations Based on Tight-Binding Quantum Chemical Calculations. *J. Chem. Theory Comput.* 15, 2847–2862 (**2019**).
34. Weigend, F. & Ahlrichs, R. Balanced basis sets of split valence, triple zeta valence and quadruple zeta valence quality for H to Rn: Design and assessment of accuracy. *Phys. Chem. Chem. Phys.* 7, 3297–3305 (**2005**).
35. Dolg, M., Stoll, H., Savin, A. & Preuss, H. Energy-adjusted pseudopotentials for the rare earth elements. *Theor. Chim. Acta* 75, 173–194 (**1989**).
36. Andrae, D., Häussermann, U., Dolg, M., Stoll, H. & Preuss, H. Energy-adjustedab initio pseudopotentials for the second and third row transition elements. *Theor. Chim. Acta* 77, 123–141 (**1990**).
37. Metz, B., Stoll, H. & Dolg, M. Hartree-Fock-adjusted pseudopotentials for post-d main group elements: Application to PbH and PbO. *J. Chem. Phys.* 113, 2563–2569 (**2000**).
38. Peterson, K. A., Figgen, D., Goll, E., Stoll, H. & Dolg, M. Systematically convergent basis sets with relativistic pseudopotentials. II. Small-core pseudopotentials and correlation consistent basis sets for the post- d group 16–18 elements. *J. Chem. Phys.* 119, 11113–11123 (**2003**).
39. Leininger, T. *et al.* The accuracy of the pseudopotential approximation: non-frozen-core effects for spectroscopic constants of alkali fluorides XF (X = K, Rb, Cs). *Chem. Phys. Lett.* 255, 274–280 (**1996**).
40. Kaupp, M., Schleyer, P. v. R., Stoll, H. & Preuss, H. Pseudopotential approaches to Ca, Sr, and Ba hydrides. Why are some alkaline earth MX 2 compounds bent? *J. Chem. Phys.* 94, 1360–1366 (**1991**).
41. Dolg, M., Stoll, H. & Preuss, H. Energy-adjusted a b i n i t i o pseudopotentials for the rare earth elements. *J. Chem. Phys.* 90, 1730–1734 (**1989**).
42. Cao, X. & Dolg, M. Valence basis sets for relativistic energy-consistent small-core lanthanide pseudopotentials. *J. Chem. Phys.* 115, 7348–7355 (**2001**).
43. Grimme, S., Antony, J., Ehrlich, S. & Krieg, H. A consistent and accurate ab initio parametrization of density functional dispersion correction (DFT-D) for the 94 elements H-Pu. *J. Chem. Phys.* 132, 154104 (**2010**).
44. Grimme, S., Ehrlich, S. & Goerigk, L. Effect of the damping function in dispersion corrected density functional theory. *J. Comput. Chem.* 32, 1456–1465 (**2011**).
45. Weigend, F. Accurate Coulomb-fitting basis sets for H to Rn. *Phys. Chem. Chem. Phys.* 8, 1057 (**2006**).
46. Zhao, Y. & Truhlar, D. G. A new local density functional for main-group thermochemistry, transition metal bonding, thermochemical kinetics, and noncovalent interactions. *J. Chem. Phys.* 125, (**2006**).
47. Ekström, U., Visscher, L., Bast, R., Thorvaldsen, A. J. & Ruud, K. Arbitrary-Order Density Functional Response Theory from Automatic Differentiation. *J. Chem. Theory Comput.* 6, 1971–1980 (**2010**).
48. Hellweg, A., Hättig, C., Höfener, S. & Klopper, W. Optimized accurate auxiliary basis sets for RI-MP2 and RI-CC2 calculations for the atoms Rb to Rn. *Theor. Chem. Acc.* 117, 587–597 (**2007**).
49. Chmela, J. & Harding, M. E. Optimized auxiliary basis sets for density fitted post-Hartree–Fock calculations of lanthanide containing molecules. *Mol. Phys.* 116, 1523–1538 (**2018**).
50. Weigend, F. Hartree–Fock exchange fitting basis sets for H to Rn †. *J. Comput. Chem.* 29, 167–175 (**2008**).
51. Caldeweyher, E. *et al.* A generally applicable atomic-charge dependent London dispersion correction. *J. Chem. Phys.* 150, 154122 (**2019**).
52. Caldeweyher, E., Bannwarth, C. & Grimme, S. Extension of the D3 dispersion coefficient model. *J. Chem. Phys.* 147, 034112 (**2017**).
53. Rolfes, J. D., Neese, F. & Pantazis, D. A. <scp>All-electron</scp> scalar relativistic basis sets for the elements Rb–Xe. *J. Comput. Chem.* 41, 1842–1849 (**2020**).
54. Pantazis, D. A. & Neese, F. All-Electron Scalar Relativistic Basis Sets for the Lanthanides. *J. Chem. Theory Comput.* 5, 2229–2238 (**2009**).
55. Pantazis, D. A., Chen, X.-Y., Landis, C. R. & Neese, F. All-Electron Scalar Relativistic Basis Sets for Third-Row

Transition Metal Atoms. *J. Chem. Theory Comput.* 4, 908–919 (**2008**).
56. Pantazis, D. A. & Neese, F. All-electron scalar relativistic basis sets for the 6p elements. *Theor. Chem. Acc.* 131, 1292 (**2012**).
57. Pantazis, D. A. & Neese, F. All-Electron Scalar Relativistic Basis Sets for the Actinides. *J. Chem. Theory Comput.* 7, 677–684 (**2011**).
58. Stoychev, G. L., Auer, A. A. & Neese, F. Automatic Generation of Auxiliary Basis Sets. *J. Chem. Theory Comput.* 13, 554–562 (**2017**).
59. te Velde, G. *et al.* Chemistry with ADF. *J. Comput. Chem.* 22, 931–967 (**2001**).
60. Bader, R. F. W. A bond path: A universal indicator of bonded interactions. *J. Phys. Chem. A* 102, 7314–7323 (**1998**).
61. Richard F. W. Bader. *Atoms in Molecules: A Quantum Theory.* (Oxford University Press, **1990**).
62. KUMAR, P. S. V., RAGHAVENDRA, V. & SUBRAMANIAN, V. Bader's Theory of Atoms in Molecules (AIM) and its Applications to Chemical Bonding. *J. Chem. Sci.* 128, 1527–1536 (**2016**).
63. Lu, T. & Chen, F. Multiwfn: A multifunctional wavefunction analyzer. *J. Comput. Chem.* 33, 580–592 (**2012**).
64. Mitoraj, M. & Michalak, A. Natural orbitals for chemical valence as descriptors of chemical bonding in transition metal complexes. *J. Mol. Model.* 13, 347–355 (**2007**).
65. Mitoraj, M. P., Michalak, A. & Ziegler, T. A combined charge and energy decomposition scheme for bond analysis. *J. Chem. Theory Comput.* 5, 962–975 (**2009**).
66. Bickelhaupt, F. M. & Baerends, E. J. Kohn-Sham Density Functional Theory: Predicting and Understanding Chemistry. in vol. 13 1–86 (**2000**).
67. Couzijn, E. P. A., Lai, Y.-Y., Limacher, A. & Chen, P. Intuitive Quantifiers of Charge Flows in Coordinate Bonding. *Organometallics* 36, 3205–3214 (**2017**).
68. Grimme, S., Hansen, A., Brandenburg, J. G. & Bannwarth, C. Dispersion-Corrected Mean-Field Electronic Structure Methods. *Chem. Rev.* 116, 5105–5154 (**2016**).
69. Lu, T. & Chen, Q. Independent gradient model based on Hirshfeld partition: A new method for visual study of interactions in chemical systems. *J. Comput. Chem.* 43, 539–555 (**2022**).
70. Ertl, P. A Web Tool for Calculating Substituent Descriptors Compatible with Hammett Sigma Constants**. *Chemistry–Methods* 2, (**2022**).
71. Hansch, C., Leo, A. & Taft, R. W. A survey of Hammett substituent constants and resonance and field parameters. *Chem. Rev.* 91, 165–195 (**1991**).
72. Crabtree, R. H. Deactivation in Homogeneous Transition Metal Catalysis: Causes, Avoidance, and Cure. *Chem. Rev.* 115, 127–150 (**2015**).
73. Tang, C. Y. *et al.* Sterically Encumbered Iridium Bis(N-heterocyclic carbene) Systems: Multiple C−H Activation Processes and Isomeric Normal/Abnormal Carbene Complexes. *Organometallics* 28, 3059–3066 (**2009**).
74. Graham, D. C., Cavell, K. J. & Yates, B. F. The influence of N-substitution on the reductive elimination behaviour of hydrocarbyl–palladium–carbene complexes—a DFT study. *Dalt. Trans.* 60, 1768 (**2006**).
75. Bot, M. Gas-phase Study of Dispersion-bound Complexes. Diss. ETH No. 26502 (**2019**).
